# Electrochemical Deoxygenation of a Ti^IV^–Oxo Complex to Enable Catalytic Reductive Oxygen-Atom Transfer from Organic Molecules

**DOI:** 10.1021/jacs.6c11967

**Published:** 2026-09-01

**Authors:** Ruohan Deng, Tianxiao Jiang, Haoran Chen, Ilia A. Guzei, Leah C. Garman, Xianyuan Zhao, Yang Yang, Shannon S. Stahl

**Affiliations:** Department of Chemistry, University of Wisconsin−Madison, 1101 University Avenue, Madison, Wisconsin 53706, United States

## Abstract

Well-defined (TTP)­Ti^II^ (TTP = 5,10,15,20-tetra­tolylporphyrin) complexes have been shown to promote stoichiometric deoxygenation of organic molecules, such as sulfoxides and epoxides, forming a stable (TTP)­Ti^IV^=O product. Here, we explore strategies to promote catalytic deoxygenation reactivity by regenerating (TTP)­Ti^II^ through chemical or electrochemical reduction. A survey of Lewis acids revealed the effectiveness of B­(OPh)_3_ in promoting activation and loss of the Ti-oxo group, enabling Ti^II^-mediated reductive oxygen-atom transfer (OAT) of diphenyl sulfoxide. Cyclic voltammetry, coulometry, EPR spectroscopy, and X-ray crystallography have been used to probe and characterize Ti^III^ and Ti^II^ species from the electroreductive deoxygenation of the Ti^IV^=O complex. The data implicate a Lewis-acid-coupled electron-transfer deoxygenation pathway for Ti^IV^=O turnover. These fundamental studies are complemented by electrochemical reactions demonstrating catalytic reductive oxygen-atom transfer from sulfoxides, pyridine *N*-oxides, and epoxides.

## Graphical abstract

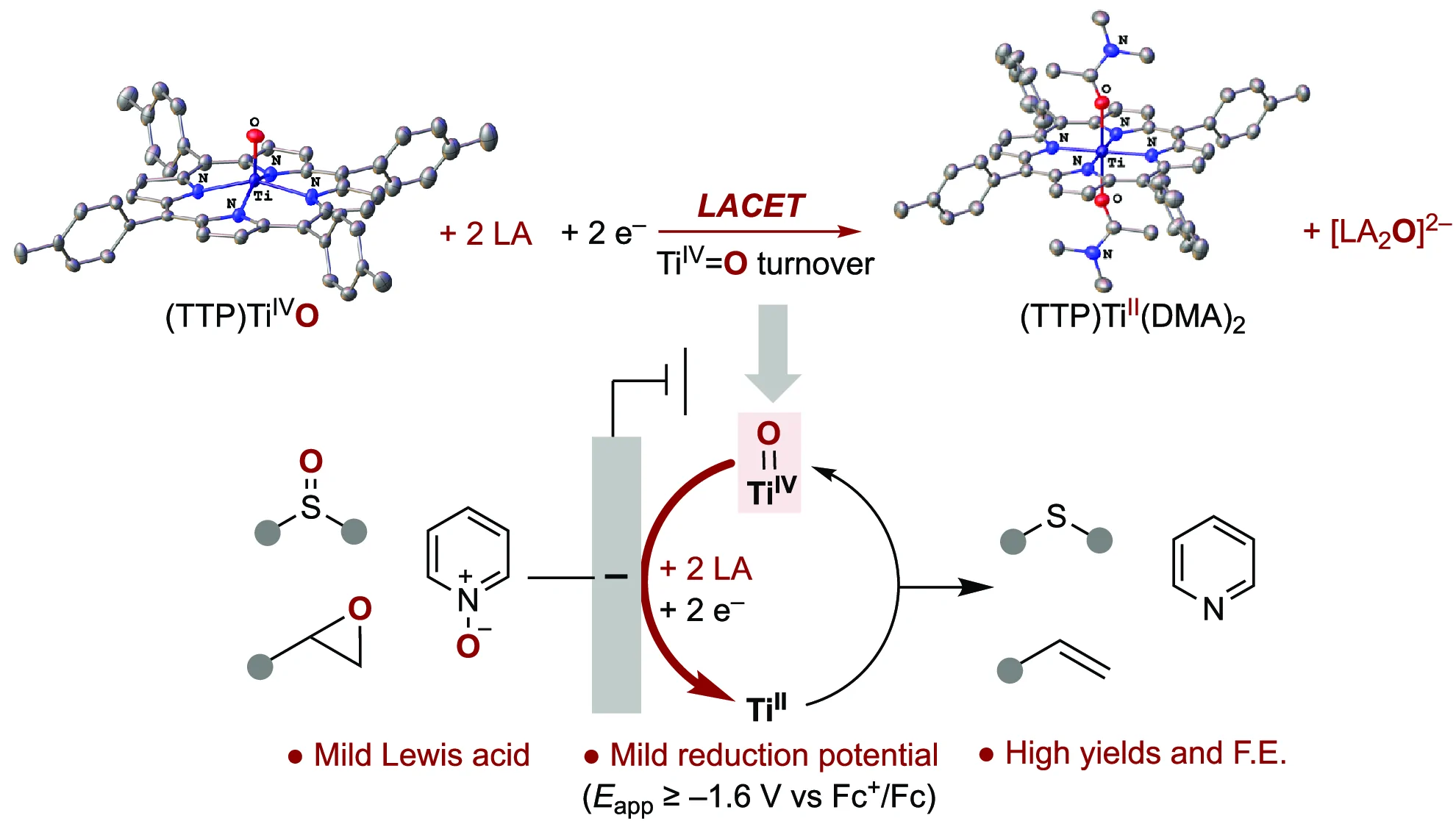

## Introduction

Deoxygenation reactions represent an important class of transformations featured in selective reduction and C–C coupling reactions with organic molecules.^1–5^ These reactions are often promoted by formation of stable oxygenated products, exemplified by formation of phosphine or titanium oxides in Wittig olefination^4^ or McMurry coupling reactions,^5^ respectively. Titanium is an oxophilic transition metal, and reduced Ti complexes can promote reductive oxygen-atom transfer (OAT) from many organic molecules.^6–11^ In the 1990s, Woo and co-workers reported the synthesis and characterization of (TTP)­Ti^II^ complexes^12^ and showed that they promote stoichiometric deoxygenation of sulfoxides and epoxides to form the corresponding thioether or alkene products together with a Ti^IV^=O species (Figure 1A).^9,13^ We postulated that (TTP)Ti complexes could support catalytic deoxygenation reactions through the development of methods for electrochemical regeneration of (TTP)­Ti^II^ from (TTP)­Ti^IV^O species.^14,15^ Studies by Kadish and co-workers, however, showed that reduction of (TPP)­Ti^IV^O (TPP = 5,10,15,20-tetraphenylporphin) leads to reduction of the porphyrin ligand, rather than titanium.^16^ This reactivity could potentially be altered in the presence of a Lewis acid, which could promote cleavage of the strong Ti=O bond upon reduction. If successful, we reasoned that such reactivity could provide the basis for electrocatalytic deoxygenation of organic molecules at comparatively mild redox potentials and with relatively mild Lewis acids (Figure 1B). This approach would complement other OAT reactions, such as those catalyzed by Mo, W, and Re species,^17–19^ which serve as deoxygenation catalysts in the presence of a stoichiometric oxygen acceptor, such as PPh_3_. Here, we report our investigation of electrochemical deoxygenation. Initial studies use chemical reductants (Zn and Mn powder) to validate the hypothesis that Lewis acids could support catalytic deoxygenation reactivity, using (TTP)­Ti^IV^O as a precatalyst. We then probe the redox behavior of (TTP)­Ti^IV^O in the absence and presence of Lewis acids, providing insights into Lewis acid-coupled electron-transfer (LACET) in the conversion of (TTP)­Ti^IV^O into (TTP)­Ti^III^ and (TTP)­Ti^II^ species. These results provide the basis for electrocatalytic deoxygenation of sulfoxides, pyridine *N*-oxides, and epoxides.

**Figure 1:**
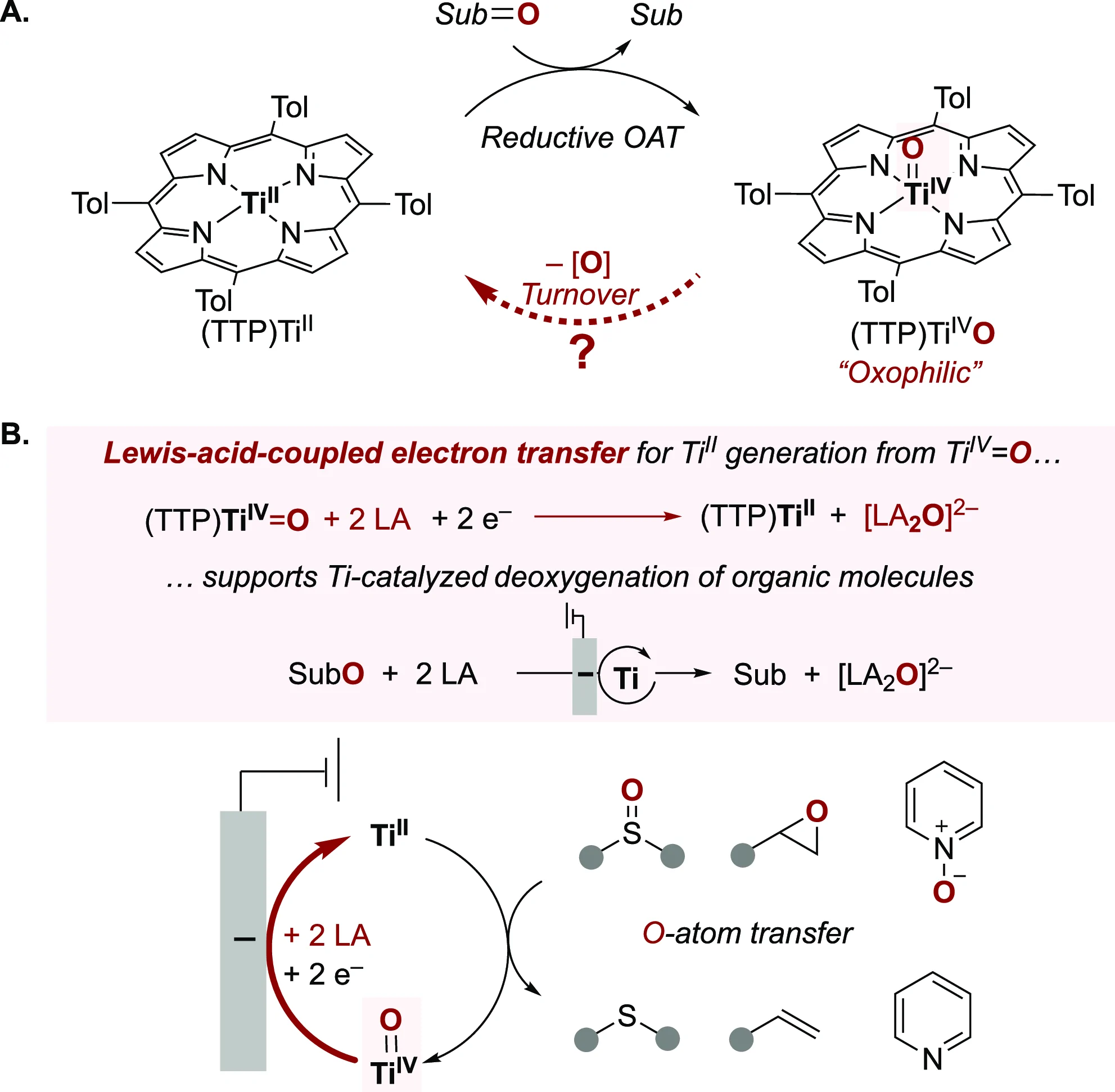
(A) Catalytic oxygen-atom transfer from Ti^IV^–oxo species could provide the basis for catalytic deoxygenation reactions. (B) Lewis acid-coupled electron transfer promotes reduction of Ti^IV^=O to Ti^II^, enabling catalytic deoxygenation reactions.

## Results and Discussion

### Development of Chemical and Electrochemical Ti-Catalyzed Deoxygenation

We initiated our study by using chemical reductants to probe the ability of Lewis acids to promote (TTP)­Ti-catalyzed conversion of diphenyl sulfoxide (**1a**) to diphenyl sulfide (**2a**) with Zn and Mn powders as the stoichiometric reductant in *N,N*-dimethylacetamide (DMA) as the solvent. (TTP)­Ti^IV^O (5 mol %) was used as the catalyst precursor. Negligible reactivity was observed in the absence of a Lewis acid with either reductant (Figure 2A). The presence of Lewis acidic metal salts, MgCl_2_ and AlCl_3_, contributed to the formation of **2a** in moderate yields. Similar outcomes were observed with silyl chlorides, such as TMSCl and TESCl (TMS = trimethylsilyl; TES = triethylsilyl), which have been used in other Ti-catalyzed reduction reactions.^20–22^ Borate esters^23^ proved to be the most effective, with B­(OPh)_3_ supporting a nearly quantitative yield of **2a** with both Zn and Mn.

**Figure 2:**
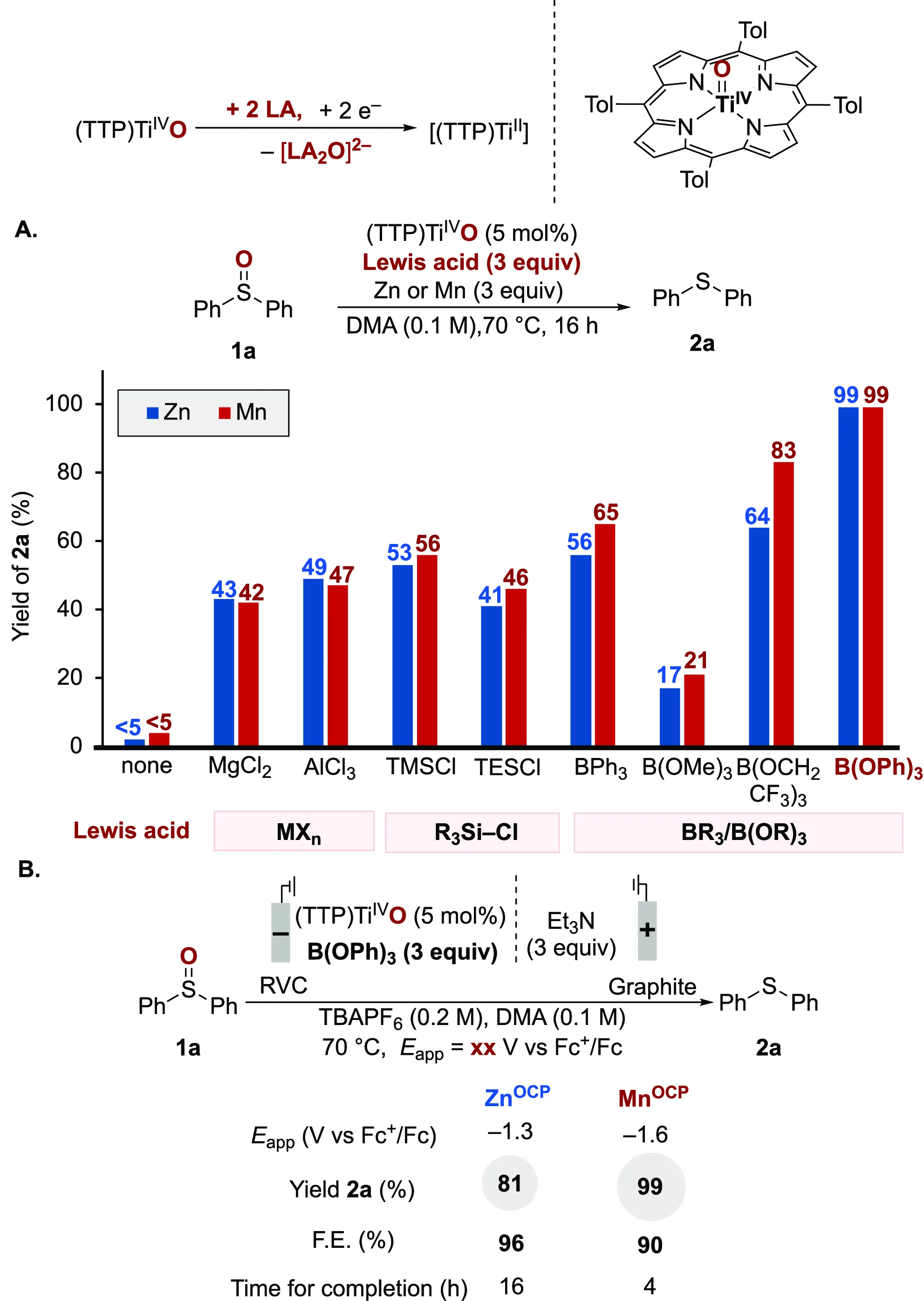
(A) Evaluation of various Lewis acids for Ti-catalyzed deoxygenation of **1a** with metal reductants (Zn or Mn). Yields determined by ^1^H NMR spectroscopy (int. std. = 1,3,5-trimethoxybenzene). (B) (TTP)­Ti-catalyzed deoxygenation of **1a** using constant potential electrolysis in a divided cell (Ag/AgNO_3_ reference electrode). TBA = *n*-tetrabutylammonium.

These promising results with chemical reductants prompted us to conduct Ti-catalyzed deoxygenation of **1a** under electrochemical conditions with B­(OPh)_3_ as the Lewis acid (Figure 2B). The reactions were performed in a divided cell using constant potential electrolysis in DMA. Two potentials were evaluated, one near the open circuit potential (OCP) of Zn and the other near the OCP of Mn:^24^ *E*_OCP(Zn)_∼ −1.36 V and *E*_OCP(Mn)_ = −1.56 V (all potentials reported versus ferrocenium/ferrocene, Fc^+^/Fc, as the reference, see Figure S2). Good yields of **2a** were observed at both potentials, but a nearly quantitative yield was accessed in only 4 h at the more reducing potential (Figure 2B).

### Mechanistic Studies of the Deoxygenation of (TTP)­Ti^IV^O

The results described above indicate that the Lewis acid plays a crucial role in promoting reduction of (TTP)­Ti^IV^O to the catalytically active low-valent Ti species. Efforts to interrogate this process further were initiated by analyzing the redox properties of (TTP)­Ti^IV^O by cyclic voltammetry (CV) (Figure 3A). Two well-defined redox features were observed with cathodic peak potentials of −1.45 V (*E*_c1_) and −2.00 V (*E*_c2_). The redox behavior of (TTP)­Ti^IV^O aligns with CV data for (TPP)­Ti^IV^O reported previously by Kadish and co-workers and is consistent with sequential electron-transfer without cleavage of the Ti^IV^=O bond.

**Figure 3:**
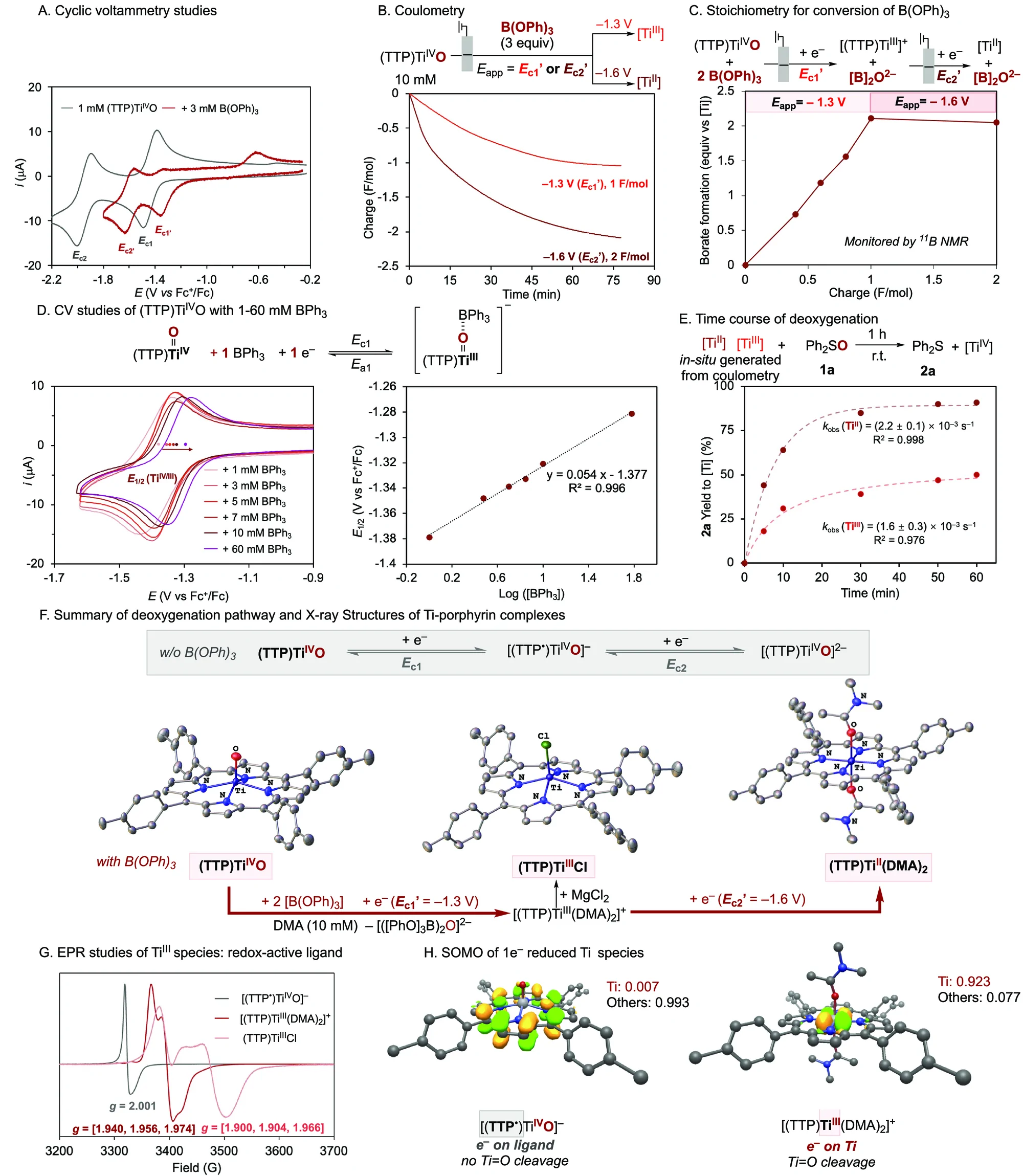
(A) Cyclic voltammetry (CV) analysis of 1 mM (TTP)­Ti^IV^O in the absence (gray) and presence (red) of 3 mM B­(OPh)_3_, 5 mL DMA, r.t. (B) Coulometric analysis of (TTP)­Ti^IV^O (10 mM) with B­(OPh)_3_ (30 mM) at *E*_c1_′ or *E*_c2_′ (for conditions, see Section S5.1 in the SI). (C) ^11^B NMR spectroscopic analysis showing formation of borate species during reduction of (TTP)­Ti^IV^O in the presence of B­(OPh)_3_. Conditions: Divided cell, cathodic chamber: 10 mM (TTP)­Ti^IV^O, 5 mL DMA, 4.5 equiv B­(OPh)_3_, 0.2 M TBAPF_6_; anodic chamber: 5 mL DMA, 0.2 M TBAPF_6_, 3 equiv Et_3_N. *E*_app_ = −1.3 V and then −1.6 V. (D) CV analysis of (TTP)­Ti^IV^O (1 mM) in the presence of BPh_3_ (1–60 mM) in DMA (0.2 M TBAPF_6_, scan rate 100 mV/s), r.t. (E) Reaction time course for Ph_2_SO deoxygenation mediated by electrochemically generated (TTP)­Ti^III^ and (TTP)­Ti^II^ species (Conditions: 10 mM (TTP)­Ti^III^ and (TTP)­Ti^II^ species, 100 mM Ph_2_SO, DMA, 1 h, r.t.). (F) (TTP)­Ti^IV^O deoxygenation sequence, forming [(TTP^•^)­Ti^IV^O]^−^ and [(TTP)­Ti^IV^O]^2–^ in the absence of B­(OPh)_3_, and formation oxo free species in the presence of B­(OPh)_3_, together with single-crystal X-ray structures of (TTP)­Ti^IV^O, (TTP)­Ti^III^Cl, and (TTP)­Ti^II^(DMA)_2_. Ellipsoids shown at 50% probability, all H–atoms omitted for clarity. (G) X-band EPR spectra of one-electron-reduced Ti species generated in the presence and absence of B­(OPh)_3_: [(TTP^•^)­Ti^IV^O]^−^ (gray), [(TTP)­Ti^III^]^+^ (dark red), and (TTP)­Ti^III^Cl (pink) (115 K, microwave frequency 9.303 GHz, microwave power 0.6325 mW). (H) SOMO images of [(TTP^•^)­Ti^IV^O]^−^ and [(TTP)­Ti^III^(DMA)_2_]^+^ derived from DFT calculations.

Upon addition of three equivalents of B­(OPh)_3_, both reduction peaks shift to more positive potentials (*E*_c1_′ = −1.33 V, *E*_c2_′ = −1.61 V). The former feature (*E*_c1_′) is distinctly irreversible. The less-well-behaved redox behavior of (TTP)­Ti^IV^O in the presence of B­(OPh)_3_ was probed further by coulometry studies. A solution of 10 mM (TTP)­Ti^IV^O with 30 mM B­(OPh)_3_ was electrolyzed at −1.3 V and at −1.6 V, and the data show passage of approximately 1 F/mol and 2 F/mol, respectively, at these two potentials (Figure 3B). The boron speciation in this process was analyzed by ^11^B NMR spectroscopy during the electrolysis of (TTP)­Ti^IV^O in the presence of 4.5 equiv B­(OPh)_3_. Constant potential electrolysis initiated at −1.3 V and continued until 1 F/mol of charge had passed resulted in the conversion of 2 equiv of B­(OPh)_3_ into borate species (Figure 3C). Passing an additional 1 F/mol at −1.6 V showed no additional changes in the boron speciation (see Section S5.3 of the Supporting Information for details). These data suggest that the reductive deoxygenation of (TTP)­Ti^IV^O involves 2 equiv of B­(OPh)_3_ but only one electron, as depicted in the chemical equations in Figure 3C. This result rationalizes the irreversible CV behavior associated with the Ti^IV/III^ in the presence of B­(OPh)_3_ (cf. Figure 3A), since reduction is paired with loss of the oxo ligand, contrasting the relatively reversible Ti^III/II^ feature.

Variation of the B­(OPh)_3_ concentration (3–9 mM) leads to systematic changes in the Ti^IV/III^ potential, with a plot of *E*_c_′ versus log­[B­(OPh)_3_] exhibiting a slope of 71 mV/decade (Figure S6b). The poor reversibility of the CV feature complicates this analysis and prompted us to analyze the same behavior with the somewhat weaker Lewis acid BPh_3_ ^25^ (Figure 3D).^26^ More reversible Ti^IV/III^ CV behavior was observed with BPh_3_, and variation of the BPh_3_ concentration (1–60 mM) revealed an approximately Nernstian anodic shift of the Ti^IV/III^ redox potential: *E*_1/2_ versus log­[BPh_3_] = 54 mV/decade. These data provide support for a Lewis acid-coupled electron-transfer (LACET) pathway for (TTP)­Ti^IV^O deoxygenation.

Deoxygenation (TTP)­Ti^IV^O at the Ti^IV/III^ redox potential rationalizes the ability of the (TTP)Ti catalyst to promote **1a** deoxygenation with Zn or at −1.3 V (cf. Figure 2B). To probe this reactivity further, we generated TTP-ligated Ti^III^ and Ti^II^ by performing constant potential electrolysis of (TTP)­Ti^IV^O in the presence of 3 equiv of B­(OPh)_3_ at −1.3 and −1.6 V, respectively. Time-course analysis of the stoichiometric reaction of these species with **1a** showed that both Ti^III^ and Ti^II^ promote conversion of **1a** to Ph_2_S in yields consistent with their one- and two-electron redox stoichiometries. The Ti^II^ species is more reactive, exhibiting a higher rate than that of Ti^III^ (Figure 3E). Fitting of these time-course data to a pseudo-first-order kinetic model (Ph_2_SO: Ti = 10) reveals rate constants of *k* = 2.2 × 10^–2^ M^–1^ s^–1^ for Ti^II^ and *k* = 1.6 × 10^–2^ M^–1^ s^–1^ for Ti^III^ in the deoxygenation of Ph_2_SO.

Structural, spectroscopic, and computational studies provided further insights into the Ti-based redox reactivity associated with the deoxygenation reactions. An X-ray crystal structure of (TTP)­Ti^IV^O reveals a terminal oxo ligand with a Ti–O bond length of 1.641(3) Å reflecting a “short” double bond or “long” triple bond, consistent with competition between the oxo and porphyrin ligands for the π-symmetry orbitals on Ti (Figure 3F).^27^

One-electron electrochemical reduction of (TTP)­Ti^IV^O in the absence of B­(OPh)_3_ afforded a new Ti species that exhibits a sharp, isotropic signal in the EPR spectrum at *g* = 2.001 (Figure 3G). In contrast, the product of one-electron reduction of (TTP)­Ti^IV^O in the presence of B­(OPh)_3_ exhibits a broad, anisotropic EPR signal (*g* = [1.940, 1.956, 1.974]) (Figure 3G). Efforts to obtain X-ray crystal structures of these complexes were unsuccessful; however, addition of MgCl_2_ to the latter reaction generated (TTP)­Ti^III^Cl, which was isolated and characterized by single-crystal X-ray diffraction (Figure 3F, see Section S2.5 in the Supporting Information). EPR spectroscopic analysis of this complex revealed an anisotropic feature with *g* = [1.900, 1.904, 1.966]. Further reduction of the [(TTP)­Ti^III^]^+^ species generated in the presence of B­(OPh_3_) afforded (TTP)­Ti^II^, isolated as the bis-DMA adduct, which was characterized by single-crystal X-ray diffraction (Figure 3F).

These experimental data were complemented by density functional theory (DFT) computational analysis^28^ of the one-electron reduced Ti complexes to gain insights into the origin of the different EPR data (Figure 3H). The *g* value at 2.001 is characteristic of an organic radical, implicating formation of a porphyrin-radical anion species, formally retaining a Ti^IV^=O fragment: [(TTP^•^)­Ti^IV^O]^−^.^16,29^ DFT analysis supports this conclusion, evident from a SOMO localized on the periphery of the porphyrin ligand (Figure 3H). The sequence of reversible one-electron reduction features evident in the gray trace of Figure 3A suggests that the Ti^IV^=O fragment remains intact even upon two-electron reduction, forming [(TTP)­Ti^IV^O]^2–^.^16^ This behavior contrasts the reduction reactions observed in the presence of B­(OPh)_3_, which results in loss of the oxo ligand. The EPR data observed following one-electron reduction implicate formation of *d* ^1^ Ti^III^ species in which the unpaired electron resides in a *d*_*xy*_-like Ti orbital according to DFT analysis (Figure 3H). This reactivity is supported by the X-ray structures of (TTP)­Ti^III^Cl and (TTP)­Ti^II^(*O*-DMA)_2_ (Figure 3F).

### (TTP)­Ti-Catalyzed Electrochemical Deoxygenation of Organic Substrates

The fundamental reactivity documented above was then translated to catalytic reactions with a series of prototypical substrates, including sulfoxides, epoxides, and pyridine *N*-oxides (Figure 4). Woo and co-workers previously showed stoichiometric deoxygenation of the former two substrate classes using a well-defined (TTP)­Ti^II^-alkyne complex,^9^ while *N*-oxides were not previously reported. Sulfoxide substrates tested in our catalytic reactions included dialkyl (**2b, 2c**), aryl/alkyl (**2d**), and diaryl (**2e–i**) derivatives, and the reactions proceeded in good yields (77–99% yield) and with high Faradaic efficiency (70–92%). No detectable dehalogenation was observed with **2g**–**i**, despite precedents for stoichiometric C­(sp^2^)–Cl cleavage with the same (TTP)­Ti^II^–alkyne complex.^9^ The catalytic conditions were also effective for catalytic deoxygenation of heteroarene *N*-oxides, including pyridine, pyrazine, and quinoline *N*-oxides, to afford **4a–e**. The reactions generally proceeded in excellent yield (83–99%), except for 4-cyanopyridine *N*-oxide (**4c**), which undergoes direct electrochemical reduction at −1.8 V (see Figure S38), a potential near the Ti^III/II^ potential.

**Figure 4:**
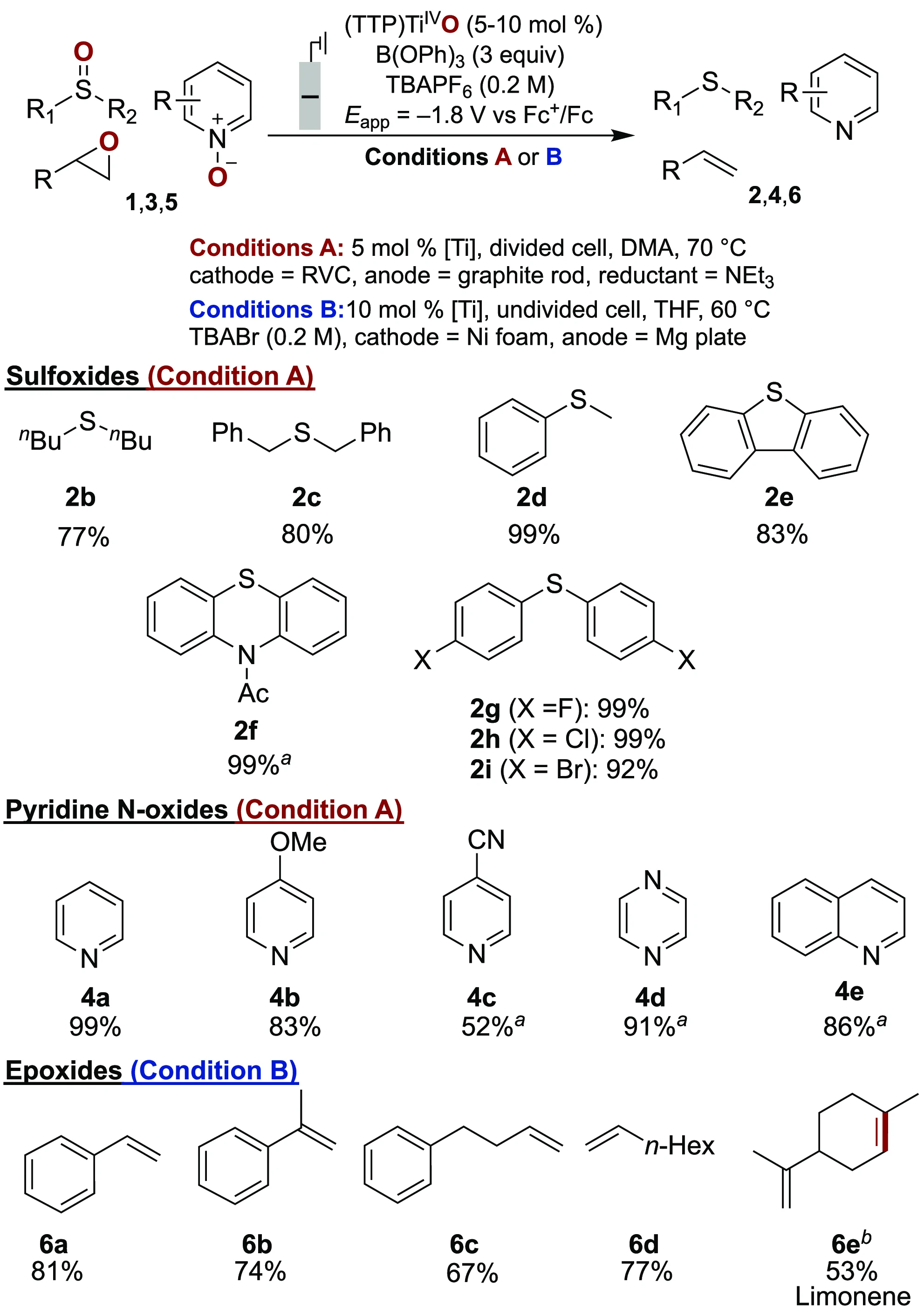
(TTP)­Ti-catalyzed electrochemical deoxygenation of organic substrates. Condition A: Divided cell, cathodic chamber: 0.5 mmol sulfoxides/N-oxides, 5 mol % (TTP)­Ti^IV^O, 5 mL DMA, 3 equiv B­(OPh)_3_, 0.2 M TBAPF_6_; anodic chamber: 5 mL DMA, 0.2 M TBAPF_6_, 3 equiv of Et_3_N. *E*_app_ = −1.8 V (Ag/AgNO_3_ as reference electrode). Condition B: Undivided cell, 0.5 mmol epoxides, 10 mol % (TTP)­Ti^IV^O, 5 mL THF, 3 equiv B­(OPh)_3_, 0.2 M TBAPF_6_, 0.2 M TBABr. *E*_app_ = −1.8 V (Ni foam as working electrode, Mg plate as counter electrode, Ag/AgNO_3_ as reference electrode). Yields determined by ^1^H NMR using 1,3,5-trimethoxybenzene as an internal standard. ^*a*^Isolated yield. ^*b*^Site for epoxide deoxygenation was marked red.

Deoxygenation of epoxides required modification of the conditions to achieve optimal results. For example, styrene oxide afforded styrene in only 11% yield under the standard conditions with DMA as the solvent. Switching the solvent from DMA to THF and empirical reoptimization of the electrochemical process (e.g., changing from divided to undivided cell and the identity of the electrodes) resulted in conversion of styrene oxide to styrene (**6a**) in 81% yield. The mechanistic basis for this change is not fully understood; however, we noted that the Ti^IV/III^ and Ti^III/II^ redox potentials of the catalyst shift to more negative values by approximately 120 mV each in THF. This observation suggests that solvent change may contribute to additional driving force that supports an improved synthetic outcome. The modified conditions also led to favorable outcomes with other epoxides, affording α-methylstyrene **6b** (74%), terminal alkyl olefins **6c** (67%) and **6d** (77%), and limonene **6e** (53%). This reactivity represents a catalytic Ti^II^-mediated process that complements early studies of epoxide deoxygenation with Cp_2_Ti^III^Cl.^30,31^

### Energetic Considerations of Ti-Mediated Deoxygenation Reactivity

The (TTP)­Ti-mediated deoxygenation reactivity may be compared with a direct reduction approach through electrochemical studies. Direct reduction of **1a** occurs at *E*′ ∼ −2.9 V, and the potential is similar in the presence of B­(OPh)_3_.^32^ Bulk electrolysis at this potential leads to deoxygenation of Ph_2_SO in 47% yield, based on ^1^H NMR analysis (Figure 5A, blue trace). (TTP)­Ti^IV^O undergoes reversible reduction at potentials of −1.45 (*E*_c1_) and −2.00 V (*E*_c2_), but retention of the oxo ligand (cf. Figure 3) prevents deoxygenation of the substrate. Loss of the oxo ligand is promoted by the presence of B­(OPh)_3_, which also contributes to reduction at less reducing potentials, −1.33 V (*E*_c1_′) and −1.61 V (*E*_c2_′). Electrolysis at these potentials leads to deoxygenation of Ph_2_SO in 81% and 99% yield, respectively (Figure 5A, red trace; cf. Figure 2B). These results show that the Ti-mediated process occurs at 1.3–1.6 V higher potential than the direct electrochemical reduction reaction, corresponding to a much lower overpotential for the mediated process.

**Figure 5:**
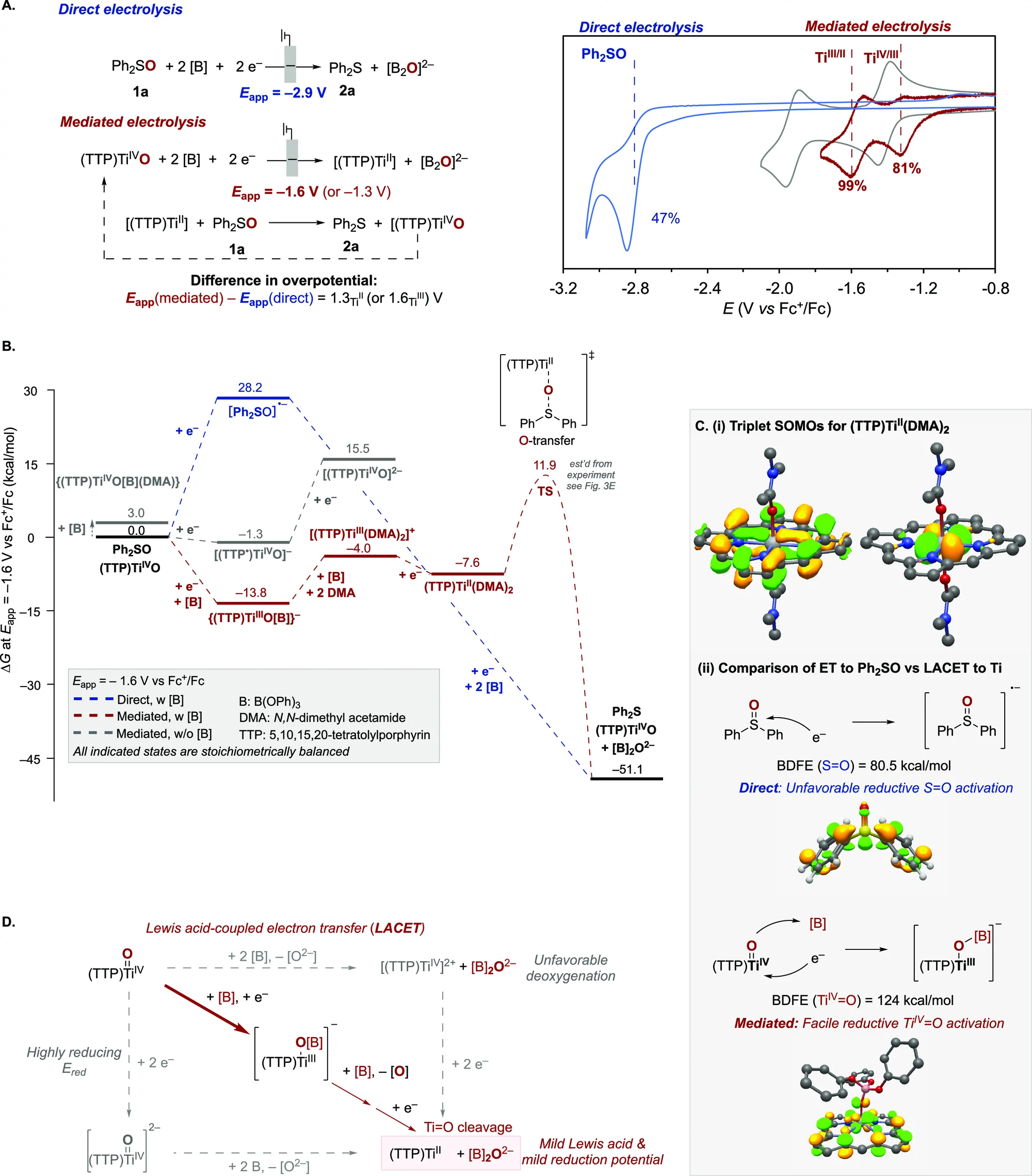
(A) Overpotential comparison of direct (blue trace: no catalyst) vs Ti-mediated (red trace: 5 mol % (TTP)­Ti^IV^O) electrochemical deoxygenation of **1a**. Conditions: Divided cell, cathodic chamber: 0.5 mmol **1a**, 5 mL DMA, 3 equiv B­(OPh)_3_, 0.2 M TBAPF_6_; anodic chamber: 5 mL DMA, 0.2 M TBAPF_6_, 3 equiv Et_3_N, *E*_app_ = −1.6 V or −1.3 V (mediated) or −2.9 V (direct). RVC as working electrode, graphite rod as counter electrode, Ag/AgNO_3_ as reference electrode. Yields determined by ^1^H NMR spectroscopy (int. std. = 1,3,5-trimethoxybenzene). Yield of **2a**% are listed separately for direct and mediated electrolysis. [B] = B­(OPh)_3_. (B) Energy diagram for comparison of three different pathways for electrochemical **1a** deoxygenation: direct, with [B] (blue); mediated, with [B] (red); mediated, without [B] (gray). Relative free energies were obtained from DFT calculations and corrected based on Δ*G* = −*n*F*E* where *E*_app_ = −1.6 V vs Fc^+^/Fc. (C) SOMO images of {(TTP)­Ti^III^O­[B]}^−^ (*p*-tolyl groups, which have no unpaired spin density, are omitted for clarity) and [Ph_2_SO]^•–^ species from DFT calculations. (D) Proposed Lewis acid-coupled electron-transfer (LACET) pathway for turnover of Ti^IV^=O to regenerate Ti^II^.

DFT calculations were performed to evaluate the energetics of the direct and mediated deoxygenation reactions (Figure 5B).^28^ An applied reduction potential of −1.6 V was used as the basis for these calculations, reflecting the potential that enables near-quantitative deoxygenation of Ph_2_SO (cf. Figure 2B; variation of this potential would lead to Nernstian shifts in the free energies of redox steps: Δ*G* = −nF*E*). The ground-state (TTP)­Ti^IV^O complex (together with the other stoichiometric species in the reaction) was defined as the reference energy of Δ*G* = 0 kcal/mol. B­(OPh)_3_ (abbreviated as [B]) does not form a stable adduct with (TTP)­Ti^IV^O, consistent with experimental findings. The Ti^IV^O–[B] adduct is calculated to be unfavorable by 3 kcal/mol relative to dissociated Ti^IV^O and [B] species. On the other hand, B­(OPh)_3_ leads to more favorable reduction of Ti^IV^O by forming a (Ti^III^O­[B])^−^ adduct, which is calculated to be 12.5 kcal/mol more favorable than the free [(TTP)­TiO]^−^/[B] species. Further 1 e^–^ reduction of (Ti^III^O­[B])^−^ is uphill, reflecting the more negative Ti^III/II^ redox potential, and forms (TTP)­Ti^II^(DMA)_2_ upon loss of the oxo ligand through formation of [(PhO)_3_B–O–B­(OPh)_3_]^2–^. The (TTP)­Ti^II^(DMA)_2_ species is calculated to be a ground-state triplet, with one of the unpaired electrons distributed in π-symmetry porphyrin-based orbitals (88%) mixed with a d_*yz*_-like orbital (12%), and the other localized in the in-plane d_*xy*_-like orbital (Figure 5C-*i*). Deoxygenation of Ph_2_SO will require intersystem crossing to access the singlet Ti^IV^O and Ph_2_S complex. Computational details of this pathway are the focus of ongoing investigation; however, a barrier of Δ*G* ^‡^ = 19.5 kcal/mol for this oxo-transfer reaction may be derived from the kinetics of the reaction of electrochemically generated (TTP)­Ti^II^ species with Ph_2_SO (cf. Figure 3E), adjusted for standard corrections to account for the nonstandard-state concentrations of the reagents (see Section S9 in the Supporting Information for details). Similar DFT computational analysis has been conducted with BPh_3_ as the Lewis acid, using a −1.8 V applied potential to reflect the Ti^III/II^ redox potential observed in the presence of BPh_3_ (see Figure S8 in the SI). Some minor differences are evident; however, the analysis reinforces the pathway in Figure 5B involving a Lewis acid-coupled electron-transfer mechanism for Ti^IV^O deoxygenation prior to Ti^II^-promoted OAT from Ph_2_SO.

The notable difference in redox potentials between Ph_2_SO and the titanium complexes is readily rationalized by the metal (or porphyrin)-based orbitals that are accessible in Ti^IV^O and Ti^IV^O–[B], which lie at lower energy than the antibonding orbital with mixed S–O and aryl π character in Ph_2_SO (Figure 5C-*ii*). Even though reduction of Ti^IV^ to Ti^II^ is much easier than Ph_2_SO, there is a 43.5 kcal/mol driving force for Ph_2_S-to-Ti OAT. This analysis highlights the benefit of a mediated strategy for deoxygenation of organic substrates, whereby the Ti-based mediator has relatively low-lying Ti-based orbitals that enable electron transfer at moderate redox potentials while leveraging the high Ti^IV^=O bond strength to promote OAT.

These results also highlight the beneficial effect of the Lewis acid-coupled electron-transfer (LACET) mechanism for reduction of the Ti^IV^=O species (Figure 5D). Direct deoxygenation of the (TTP)­Ti^IV^O by a Lewis acid is unfavorable, while direct reduction of (TTP)­Ti^IV^O leads to reduction of the porphyrin ligand, leaving the Ti=O bond intact. When paired, the Lewis acid and reducing equivalents provide an efficient pathway for cleavage of the Ti=O bond and formation of the reactive Ti^II^ species, which supports mediated electrochemical deoxygenation of the organic substrate.

## Conclusions

The results described herein introduce an electrochemical pathway for selective deoxygenation of organic substrates using a Ti-base mediator. Use of mild Lewis acid, B­(OPh)_3_, facilitates electrochemical reductive turnover of (TTP)­Ti^IV^O to (TTP)­Ti^II^ and subsequent OAT from the oxygenated organic substrates. Mechanistic studies, including electrochemical (CV, coulometry), EPR spectroscopic, and X-ray crystallographic analysis, have been used to identify and characterize the key low-valent Ti intermediates. Collectively, these data highlight a Lewis acid-coupled electron-transfer pathway for electrochemical generation of a reduced Ti mediator that can promote efficient OAT reactivity.

## Supplementary Material



## Notes

Experimental details, electrochemical data, compound characterization data with NMR spectra (PDF)
