# Supplementary material for: Electrochemical Deoxygenation of a TiIV–Oxo Complex to Enable Catalytic Reductive Oxygen-Atom Transfer from Organic Molecules
Source: J Am Chem Soc. 2026 Sep 1;148(36):39046–53. doi: 10.1021/jacs.6c11967 (PMC13596083; doi:10.1021/jacs.6c11967)
Supplement: Supplementary file 1 [file ja6c11967_si_001.pdf]

Electrochemical Deoxygenation of a Ti^{IV}-Oxo Complex to Enable Catalytic Reductive Oxygen-Atom Transfer from Organic Molecules

Ruohan Deng,[1‡] Tianxiao Jiang,[1‡] Haoran Chen,[1] Ilia A. Guzei,[1] Leah C. Garman,[1] Xianyuan Zhao,[1] Yang Yang,[1]* and Shannon S. Stahl[1]*

[1]Department of Chemistry, University of Wisconsin–Madison, 1101 University Avenue, Madison, Wisconsin 53706, United States.

*Email: yyang222@wisc.edu, stahl@chem.wisc.edu

Contents

1. General Considerations S3
2. Synthesis of Ti Complexes S4
2.1. (TTP)$Ti^{IV}Cl_2$ S4
2.2. (TTP)Ti^{IV}O S5
2.3. (TTP)$Ti^{IV}(OPh)_2$ S6
2.4. (TTP)$Ti^{II}(DMA)_2$ S6
2.5. (TTP)Ti^{III}Cl S8
3. Optimization of Ti-catalyzed deoxygenation of sulfoxides S9
3.1. Chemical deoxygenation of sulfoxides with metal reductants S9
3.2. Optimization of electrochemical deoxygenation of sulfoxides S10
3.3. Screening of Lewis acids for sulfoxides deoxygenation S11
3.4. Screening of applied potentials S12
3.5. Electrochemical sulfoxides deoxygenation S13
3.6. Time course of electrochemical deoxygenation of Ph_2SO S14
4. CV Studies S15
4.1. General CV considerations S15
4.2. (TTP)Ti^{IV}O in DMA S16
4.3. (TTP)Ti^{IV}O + $B(OPh)_3$ in DMA S16
4.4. (TTP)Ti^{IV}O + BPh_3 in DMA S18
4.5. (TTP)Ti^{IV}O + $B(OPh)_3$ in THF S19
5. Coulometry Studies: Reduction of (TTP)Ti^{IV}O S19
5.1 Reduction of (TTP)Ti^{IV}O in the absence and presence of $B(OPh)_3$ S19
5.2 Time course of stoichiometric oxo-transfer of Ph_2SO by Ti^{II} and Ti^{III} S21
5.3. EPR spectra of (TTP)Ti^{III} species S23
6. Quantitative ^{1}H and ^{11}B NMR spectroscopic studies of (TTP)Ti^{IV}O with $B(OPh)_3$ S25
6.1. Reactions of (TTP)Ti^{IV}O with $B(OPh)_3$ and BPh_3 analyzed by ^{1}H NMR spectroscopy S25
6.2. Analysis of reaction stoichiometry for reductive deoxygenation of Ph_2SO with $B(OPh)_3$ analyzed by ^{11}B NMR spectroscopy S28
6.3. Analysis of reaction stoichiometry for reductive deoxygenation of (TTP)Ti^{IV}O with $B(OPh)_3$ analyzed by ^{11}B NMR spectroscopy S32
7. Computational studies S36
7.1. Computational methods S36
7.2. Calculation of BDFE S37
7.3. Spin density results S38
7.4. Free Energy S44
7.5. Optimized Geometries S45
8. Reaction optimization of Ti-catalyzed deoxygenation of *N*-oxides and epoxides S99

8.1 Optimization of deoxygenation of N-oxides *S99*
8.2 Optimization of deoxygenation of epoxides *S100*
8.3 Bulk electrolysis data for Ti-mediated deoxygenation reactions *S102*
9. Comparison of mediated and direct electrochemical reductive deoxygenation of Ph_2SO S103
9.1 General procedures for direct and mediated electrolysis *S103*
9.2 Calculation of $\Delta G^{\ddagger}$ based on Figure 3E results *S104*
10. Product characterization data for (TTP)Ti-catalyzed deoxygenation reactions. S105
11. Crystallographic data for Ti-porphyrin complexes S112
11.1. (TTP)Ti^{IV}O *S112*
11.2. (TTP)$Ti^{IV}(OPh)_2$ *S116*
11.3. (TTP)Ti^{III}Cl *S123*
11.4. (TTP)$Ti^{II}(DMA)_2$ *S129*
12. References S133
13. NMR Spectra S137

1. General Considerations

Solvents and reagents:

Chemicals and solvents were purchased from commercially available sources and used without any additional purification. Sulfoxide, *N*-oxides and epoxide substrates were purchased from Oakwood, Combi-Blocks, TCI America, Ambeed, Sigma-Aldrich. Porphyrin ligands were purchased from Frontier Scientific Service. $TiCl_4$ was purchased from Sigma-Aldrich (1 M solution in toluene) and was stored in a nitrogen-filled glovebox. Anhydrous solvents (DMA, THF, toluene, pentane) were purchased from Sigma-Aldrich, dried through the solvent purification system and stored/used in a nitrogen-filled glovebox in the presence of 4Å molecular sieves. $CDCl_3$ and C_6D_6 were dried over 4Å molecular sieves and stored in the nitrogen-filled glovebox overnight.

NMR spectroscopy:

NMR spectra (^1H, ^{13}C, ^{11}B) were obtained with either a Bruker Avance III 400 MHz spectrometer or a Bruker Avance III 500 MHz spectrometer with reference to residual solvent peaks: $CDCl_3$ peaks at 7.26 ppm (^1H) and 77.16 ppm (^{13}C); C_6D_6 peaks at 7.16 ppm (^1H) and 128.06 ppm (^{13}C). Multiplicities are described using the following abbreviations: s = singlet, bs = broad singlet, d = doublet, dd = doublet of doublets, ddd = doublet of doublet of doublets, dt = doublet of triplets, ddt = doublet of doublet of triplets, t = triplet, td = triplet of doublets, q = quartet, m = multiplet. Chromatography was performed using an automated Biotage Isolera® with reusable 25 g Biotage® Silicycle cartridges or standard silica cartridges.

EPR spectroscopy:

All EPR spectra were recorded on Bruker EleXsys E500 spectrometer equipped with a Super Hi-Q resonator and a SuperX High Power Microwave Bridge. Temperature control was provided using a liquid nitrogen cryostat set-up (ER 4141VTC) that enabled collection of spectra in frozen solution at 115 K.

Electrodes and electrochemical experiments:

Voltammetric experiments and bulk electrolysis reactions were performed using either a CHI 600 potentiostat or a Pine WaveNow PGstat under either N_2-filled atmosphere or active N_2 flow on a Schlenk line. All electrode materials were purchased from commercial sources and used as received after cutting them to the appropriate size. Graphite rods were purchased (0.6 mm diameter) from GraphiteStore.[1] Reticulated vitreous carbon (RVC) foams (pore size 30 PPI) were purchased from Ultramet. Mg plates (AZ31B alloy) were purchased (1.0 mm thickness) from Yodaoke (Amazon).[2] Nickel foams were purchased (1.6 mm thickness) from MTI Corporation.[3] Glassy carbon working electrodes (1 mm diameter were purchased from EDAQ (ET074-1). The working electrode potentials were measured against a

Ag/$AgNO_3$ reference electrode purchased from BASi (internal solution, 0.2 M $TBAPF_6$ and 0.01 M $AgNO_3$ in DMF, part no. MF-2062).[4] The redox potential of ferrocenium/ferrocene (Fc^+/Fc) was measured (under the same experimental conditions) and used to provide an internal reference. The potential values were adjusted relative to Fc^+/Fc, and electrochemical studies in organic solvents were recorded accordingly.

2. Synthesis of Ti Complexes

2.1. (TTP)$Ti^{IV}Cl_2$

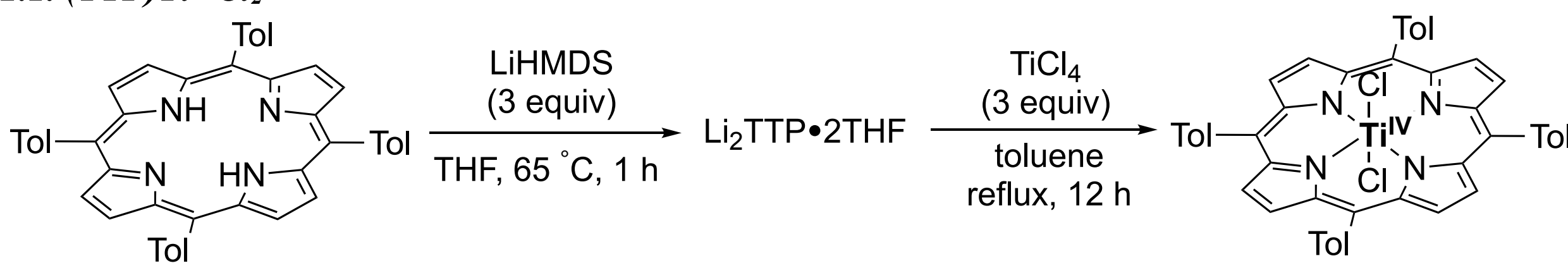

H2TTP 2.5 mmol | 99% | (TTP)$Ti^{IV}Cl_2$ 89%

Synthesis of Li_2TTP (TTP = dianion of tetratolylporphyrin). The compound was synthesized following the known literature procedure.[5] To a oven-dried two-neck round-bottom flask (equipped with a gas bubbler) containing H_2TTP (1.68 g, 2.5 mmol) in 30 mL of THF under an N_2 atmosphere on a Schlenk line was added 3 equiv of $LiN(SiMe_3)_2$ (LiHMDS), with a color change from purple to blue-green in 10 min. The solution was then heated at 65 °C under N_2 with a bubbler until all the THF had evaporated through the bubbler. The solids were immediately transferred into an N_2 filled glovebox and washed with 15 mL of pentane (5 mL × 3) to remove residual LiHMDS and $HN(SiMe_3)_2$. The dark blue precipitate was collected and dried under vacuum, which was confirmed by ^{1}H NMR spectroscopy. Yield: 2.05 g (2.48 mmol, 99%). ^{1}H NMR characterization matches with the literature values.[6]

^{1}H NMR (500 MHz, C_6D_6) δ 9.24 (s, 8H), 8.35 (d, J = 7.1 Hz, 8H), 7.43 (d, J = 7.5 Hz, 8H), 2.49 (s, 12H), 1.30 (s, br, 8H), 0.24 (s, br, 8H).

Synthesis of (TTP)$Ti^{IV}Cl_2$. The compound was synthesized using a modified literature procedure.[6] To a oven-dried 250-mL three-neck round bottom flask charged with a magnetic stir bar was added 3.8 mL of $TiCl_4$ solution (1 M in toluene, 3.75 mmol, 2.5 equiv) in the N_2-filled glovebox. The solution was then diluted with toluene to a volume of 10 mL. Li_2TPP (1.5 mmol) was dissolved in 50 mL of toluene and *slowly* added to the solution of $TiCl_4$ *dropwise* over 30–40 min, with the orange-red color of the Ti solution immediately turning dark green. The reaction was then heated to reflux under N_2 on the Schlenk line for 12 h, after which the solvent and remaining $TiCl_4$ were removed under vacuum. The solid residue was washed with pentane to afford a dark green solid (1.05 g, 89% yield), confirmed by ^{1}H NMR spectroscopy. It was stored under inert atmosphere in the glovebox. Characterization data match the literature values.[6]

Note: Slow, dropwise addition of the porphyrin solution to $TiCl_4$ is very crucial for the synthesis. Direct mixing or the reverse sequence of addition will lead to irreversible formation of $[H_4TPP]^{2+}$ as well as $(TTP)Ti^{IV}O$, which complicates purification. Anhydrous solvent is required throughout the synthesis.

^{1}H NMR (500 MHz, $CDCl_3$) δ 9.02 (s, 8H), 8.18 (d, J = 8.0 Hz, 8H), 7.60 (d, J = 7.6 Hz, 8H), 2.72 (s, 12H).

^{13}C NMR (126 MHz, $CDCl_3$): δ 146.0, 138.3, 138.0, 135.0, 131.6, 128.0, 124.2, 77.4, 21.7.

HRMS (ESI) m/z: $[M–Cl^-]$ ($C_{48}H_{38}ClN_4Ti^+$) Calcd: 753.2259; found: 753.2253.

2.2. (TTP)Ti^IV^O

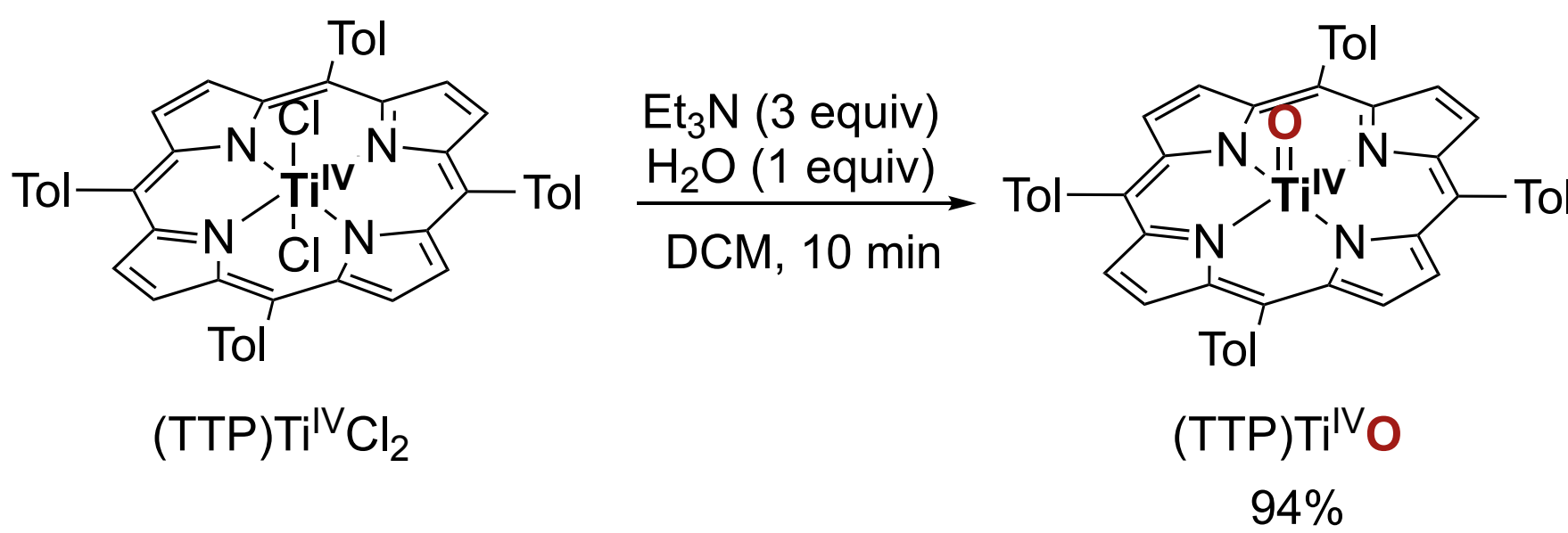

Synthesis of ***(****TTP)Ti^IV^O.* The compound was synthesized referring to a relevant literature procedure.[7] To a 250-mL round-bottom flask charged with a magnetic stir bar was added 1.05 g $(TTP)Ti^{IV}Cl_2$ (1.33 mmol) and 60 mL of CH_2Cl_2 under air. Water (24 µL, 1 equiv) was added, followed by *slow* addition of a solution of triethylamine (555 µL, 3 equiv) in 5 mL of CH_2Cl_2 with vigorous stirring. Fuming was observed immediately and the color of the solution turned from dark green to purple-pink within 5 min. After 10 min of stirring at room temperature, the solution was transferred into a 250-mL separatory funnel and extracted with water (50 mL × 3) to remove triethylammonium chloride. The organic phase was dried over anhydrous Na_2SO_4, and the solvent was removed on a rotary evaporator to obtain a pink-purple solid (0.92 g, 94% yield). Crystals suitable for X-ray diffraction were obtained via vapor diffusion of pentane into a saturated toluene solution in the freezer at −30 °C overnight (see section 11.1 for characterization details). ^{1}H NMR characterization data match the literature values.[8]

^{1}H NMR (500 MHz, $CDCl_3$): δ 9.14 (s, 8H), 8.36 (d, J = 7.6 Hz, 4H), 8.01 (d, J = 7.6 Hz, 4H), 7.65 (d, J = 7.6 Hz, 4H), 7.57 (d, J = 7.6 Hz, 4H), 2.74 (s, 12H).

^{13}C NMR (126 MHz, $CDCl_3$): δ 148.8, 139.0, 137.9, 135.0, 134.5, 132.6, 127.9, 127.7, 123.7, 21.7.

HRMS (ESI) m/z: $[M+H^+]$ ($C_{48}H_{39}N_4OTi^+$) Calcd: 735.2598; found: 735.2592.

2.3. (TTP)Ti^IV(OPh)_2

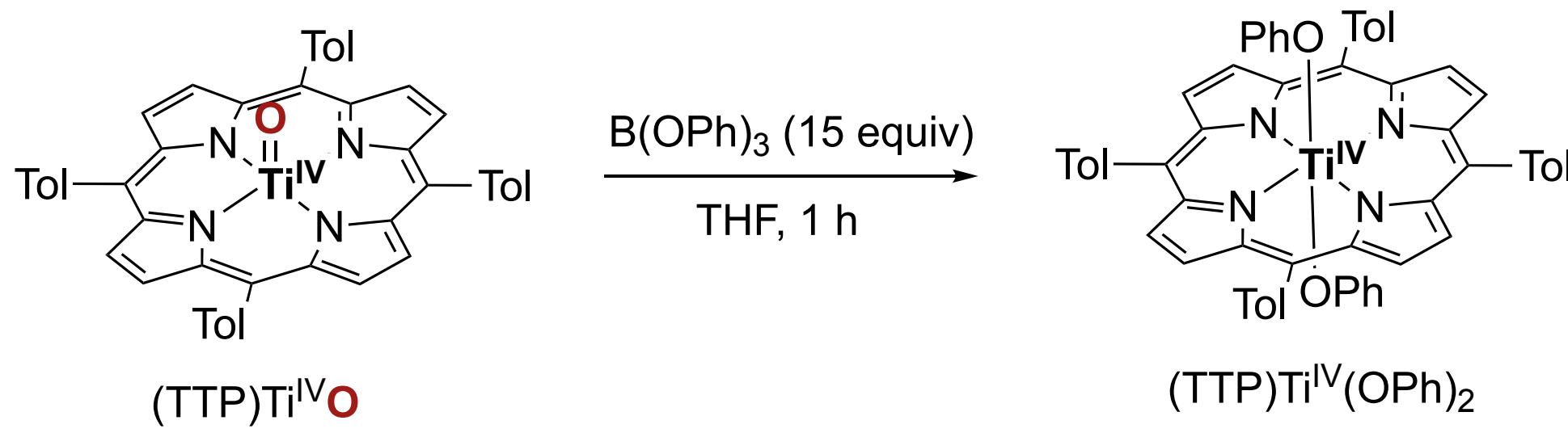

Synthesis of (TTP)Ti^IV(OPh)_2. To a 25-mL round-bottom flask charged with a magnetic stir bar was added 43.2 mg (TTP)Ti^IVO (0.06 mmol), $B(OPh)_3$ (0.9 mmol, 15 equiv, 261 mg), and THF (5 mL) in a N_2-filled glovebox. The solution immediately turned from purple to dark green, and the reaction was stirred for 1 h. THF was removed under reduced pressure, and the residue was washed with pentane (5 mL × 3). A small amount of toluene (~2 mL) was added to dissolve the residue, and the solution was filtered through a funnel and transferred to a dried 1-dram vial for crystallization (vapor diffusion with pentane) in a −30 °C freezer (see section 11.2 for characterization details). A dark blue-green crystalline solid formed after 3 d and was washed with pentane and dried under vacuum (31.4 mg, 58% yield). The ^{1}H NMR spectra data are consistent with the previous report.[9]

^{1}H NMR (500 MHz, C_6D_6): δ 9.01 (s, 8H), 8.01 (d, *J* = 7.9 Hz, 8H), 7.27 (d, *J* = 7.6 Hz, 8H), 5.89–5.75 (m, 6H), 2.72–2.61 (m, 4H), 2.40 (s, 12H).

^{13}C NMR (126 MHz, C_6D_6): δ 160.9, 146.3, 139.7, 137.5, 135.0, 131.6, 126.8, 123.7, 118.9, 115.2, 21.5.

HRMS (ESI) m/z: [M–OPh$^-$] ($C_{54}H_{43}N_4OTi^+$) Calcd: 811.2911; found: 811.2905.

2.4. (TTP)Ti^II(DMA)_2

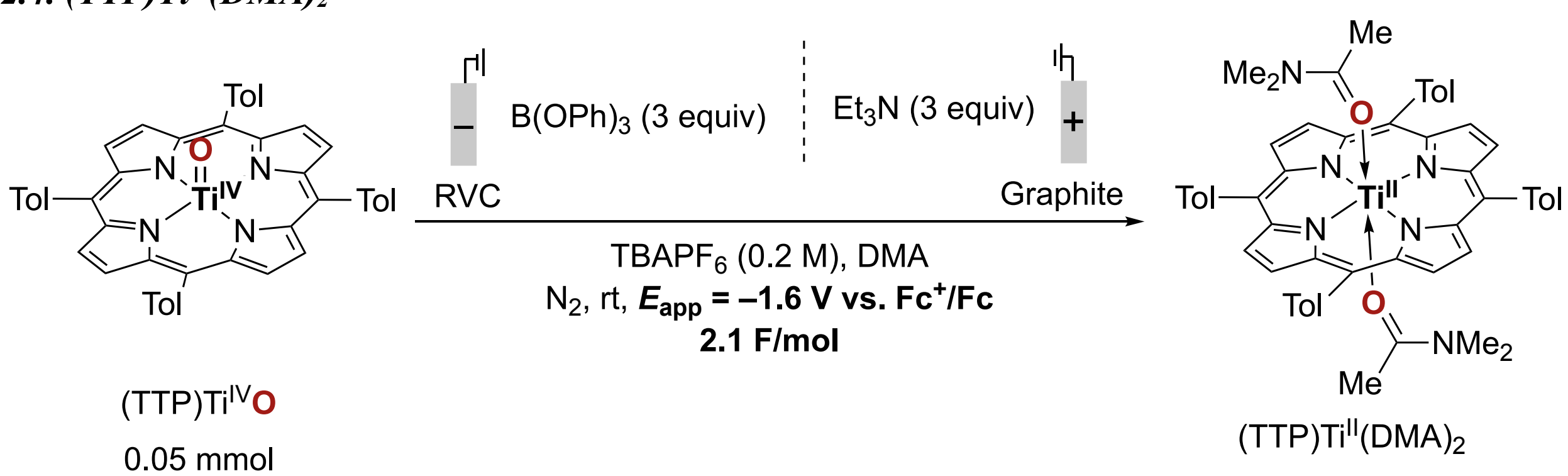

Synthesis of (TTP)Ti^II(DMA)_2

The reaction was carried out using an H-type divided cell (separated by a glass frit). In the cathodic chamber, (TTP)Ti^IVO (36.6 mg, 10 mM, 0.005 mmol), $B(OPh)_3$ (43.5 mg, 3 equiv, 0.015 mmol), and $TBAPF_6$ (387 mg, 0.2 M, 1 mmol) were dissolved in DMA (5 mL) and equipped with a reticulated vitreous carbon (RVC) electrode (30 PPI, 4.0 cm × 1.0 cm × 0.6 cm, ~3.0 cm was immersed in the solution) as the working electrode

and a Ag/AgNO$_3$ reference electrode. In the anodic chamber, TBAPF$_6$ (387 mg, 0.2 M, 1 mmol) and triethylamine (21 μL, 3 equiv, 0.15 mmol) were dissolved in DMA (5 mL) and equipped with a graphite rod anode. A constant potential of −1.6 V was applied at room temperature with magnetic stirring. The reactions were terminated when I was greater than −200 μA (i.e., $|I| < 200$ μA).

After the electrolysis, the black solution from the cathodic chamber was transferred to a round-bottom flask and the solvent was removed under reduced pressure. The residue was washed with pentane (3 × 1 mL), followed by the addition of toluene (5 mL). The resulting solution was filtered through a funnel. The filtrate was dried under reduced pressure, triturated with pentane, and dried under N$_2$ flow to obtain a black solid (23.4 mg, 54% yield).

Note: Keeping the sample under vacuum for long periods resulted in decomposition of (TTP)Ti^{II}(DMA)$_2$ even at room temperature. Do not keep the sample under vacuum for >10 min once most of the solvent is gone. Therefore, trace residue of DMA and hexane can still be observed in the ^{1}H NMR after numerous efforts of trituration with pentane. Single crystals suitable for X-ray diffractions were obtained via vapor diffusion of pentane into a saturated toluene solution in the freezer at −30 °C overnight (see section 11.4 for characterization details).

^{1}H NMR (500 MHz, C$_6$D$_6$): δ 11.87 (s, br, 6H), 10.97 (s, br, 6H), 6.70 (d, J = 7.9 Hz, 8H), 4.10 (d, J = 8.0 Hz, 8H), 2.09 (s, 12H), −9.23 (s, 8H).

^{13}C NMR (126 MHz, C$_6$D$_6$): δ 164.1, 139.0, 131.7, 18.7.

Alternatively, (TTP)Ti^{II}(DMA)$_2$ can be prepared through a stepwise pathway described below:

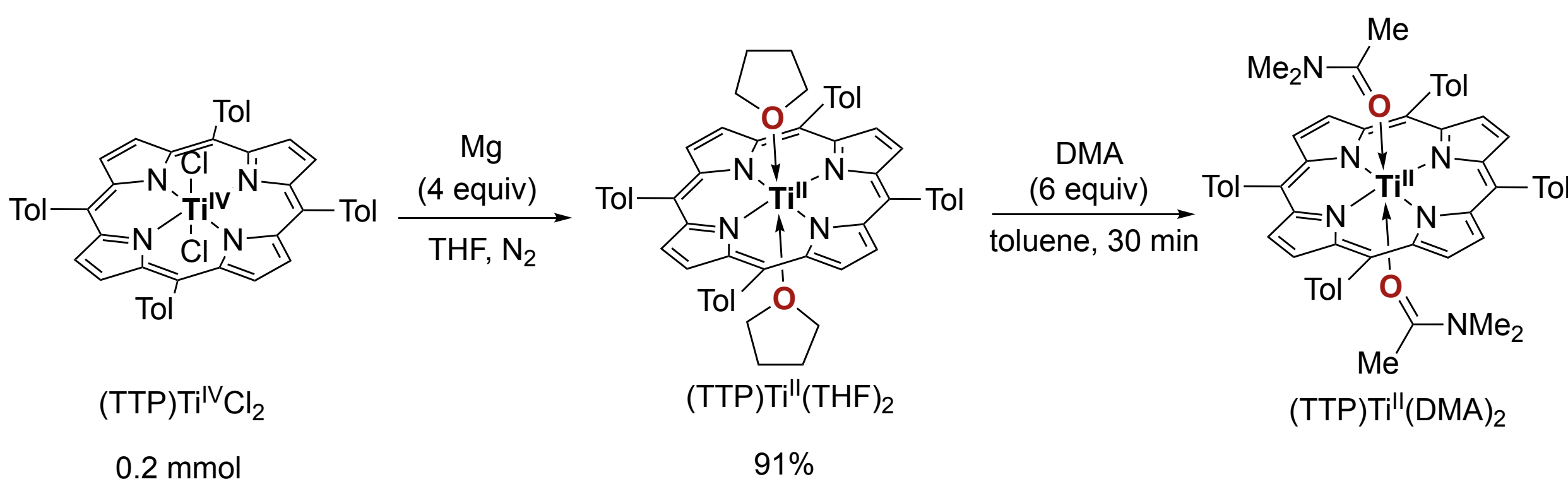

Synthesis of (TTP)Ti^{IV}(THF)$_2$. To a 250-mL round-bottom flask (dried in the oven) charged with a magnetic stir bar was added (TTP)Ti^{IV}Cl$_2$ (158 mg, 0.2 mmol), Mg powder (20 mg, 4 equiv), and THF (20 mL) in the N$_2$-filled glovebox. The solution was then stirred vigorously at room temperature for 50 min and the dark green solution turned black. The solvent was removed immediately under reduced pressure, and the remaining solid was dissolved in 6:1 toluene:THF (21 mL) and filtered through a glass-fritted funnel. The filtrate was collected, and the solvent removed under reduced pressure. The residue was washed with

hexane (5 mL × 3) to obtain (TTP)Ti^{II}(THF)$_2$ as black solid (157 mg, 91%) and stored in the freezer (−30 °C in the glovebox). *Note*: Leaving the solution after the reaction (over 8 h) will lead to complete decomposition of Ti^{II} observed by ^{1}H NMR spectroscopy, even stored in the glovebox. Immediate workup of the reaction is required. The instability of this Ti^{II} compound precluded characterization by techniques other than ^{1}H NMR spectroscopy, which matches the previous report.[10]

^{1}H NMR (500 MHz, C_6D_6) δ 17.70 (s, 8H, THF), 7.83 (s, 8H, THF), 6.28 (d, *J* = 7.9 Hz, 8H), 5.00 (d, *J* = 7.9 Hz, 8H), 1.61 (s, 12H), −5.22 (m, 8H).

Step 2: Synthesis of (TTP)Ti^{II}(DMA)$_2$: To a 25-mL vial (dried in the oven) charged with a magnetic stir bar was added (TTP)Ti^{IV}(THF)$_2$ (34.4 mg, 0.04 mmol), DMA (22.2 µL, 6 equiv), and toluene (4 mL) in the glovebox. The reaction progress was monitored by ^{1}H NMR spectroscopy and was finished within 30 min. The solution was filtered through a glass-fritted funnel, diluted with pentane (8 mL) and stored in the freezer at −30 °C for 3 d. The precipitate was separated from the supernatant and triturated with additional pentane to afford a black crystalline solid (30.0 mg, 87% yield).

2.5. (TTP)Ti^{III}Cl

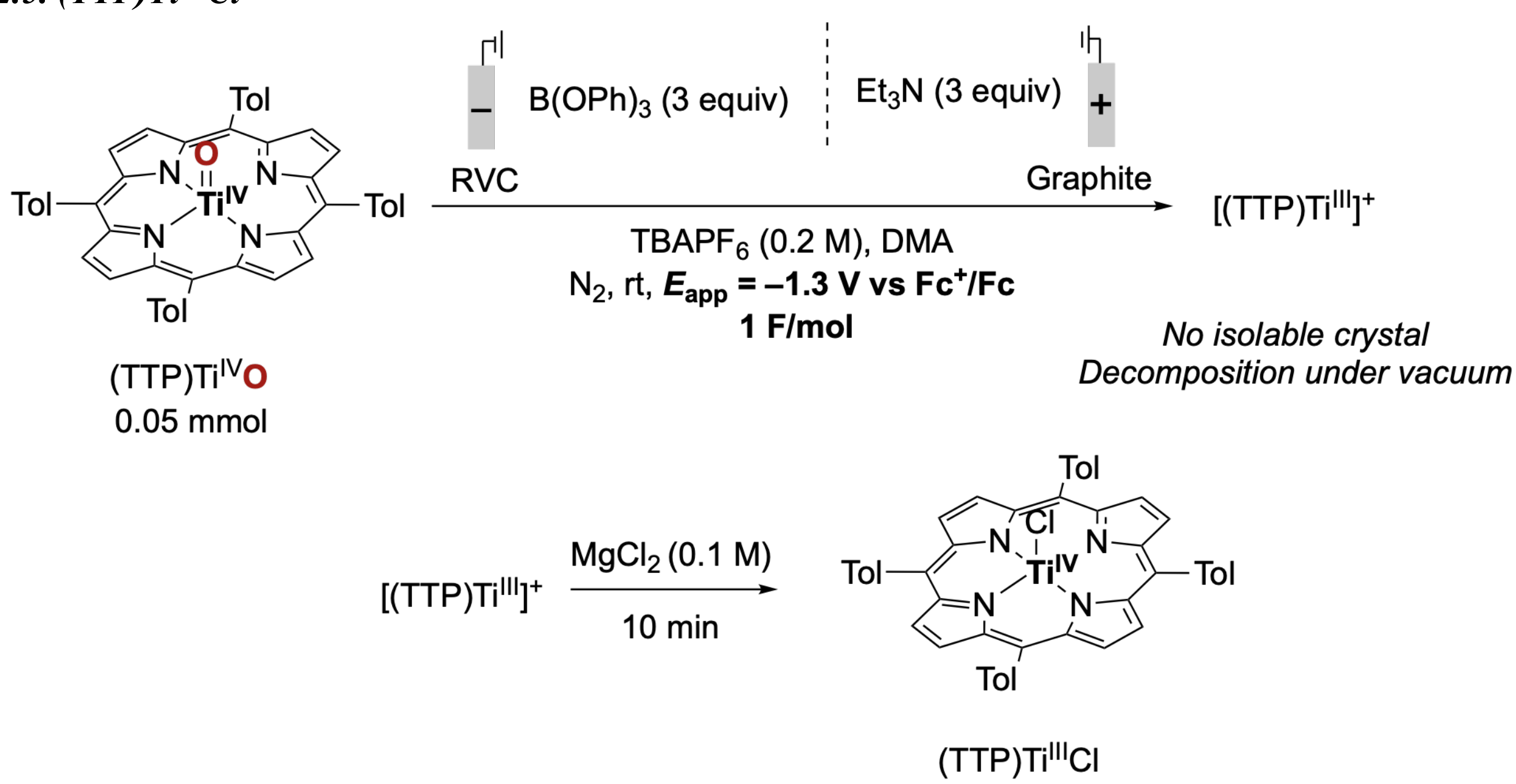

Synthesis of (TTP)Ti^{III}Cl:

The reaction was carried out using an H-type divided cell (separated by a glass frit). In the cathodic chamber, (TTP)Ti^{IV}O (36.6 mg, 10 mM, 0.005 mmol), B(OPh)$_3$ (43.5 mg, 3 equiv, 0.015 mmol), and TBAPF$_6$ (387 mg, 0.2 M, 1 mmol) were dissolved in DMA (5 mL) and equipped with a RVC electrode (30 PPI, 4.0 cm × 1.0 cm × 0.6 cm, ~3.0 cm was immersed in the solution) as the working electrode and a Ag/AgNO$_3$ reference electrode. In the anodic chamber, TBAPF$_6$ (387 mg, 0.2 M, 1 mmol) and triethylamine (21 µL, 3 equiv, 0.15 mmol) were dissolved in DMA (5 mL) and equipped with a graphite rod anode. A constant

potential of −1.3 V was applied at room temperature with magnetic stirring. Reactions were terminated when *I* was greater than −200 μA (i.e., |*I*| < 200 μA).

After the electrolysis, $MgCl_2$ (47.5 mg, 0.5 mmol, 0.1 M) was added to the solution from the cathodic chamber, and the reaction was stirred for additional 10 min, transferred to a round-bottom flask, and concentrated under reduced pressure to provide a crude residue. Pentane (1 × 3 mL) was added to wash the residue, followed by the addition of toluene (5 mL). The resulting solution was filtered. The filtrate was dried under reduced pressure, triturated with pentane, and dried under N_2 flow to obtain a purple solid (30.9 mg, 82% yield). Single crystals suitable for X-ray analysis were prepared by vapor diffusion of hexane into a saturated solution of (TTP)Ti^{III}Cl in toluene at −30 °C in the glovebox (see section 11.3 for characterization details).

HRMS (ESI) m/z: [M–Cl^-] ($C_{48}H_{38}N_4Ti^+$) Calcd: 718.2570; found: 718.2564.

3. Optimization of Ti-catalyzed deoxygenation of sulfoxides

3.1. Chemical deoxygenation of sulfoxides with metal reductants

Solvent screening under stoichiometric conditions (in-situ generation of Ti^{II})

To a 1-dram vial was added diphenyl sulfoxide (20.2 mg, 0.1 M, 0.1 mmol), (TTP)$Ti^{IV}Cl_2$ (79.6 mg, 0.1 mmol), magnesium powder (9.6 mg, 4 equiv), and solvent (1 mL) in the N_2-filled glovebox. Reactions were then removed from glovebox and stirred in a sand bath on a stir plate at 800 rpm and 70 °C overnight. 1,3,5-trimethoxybenzene was added as an internal standard (1 equiv, 0.1 mmol) for analysis by ^{1}H NMR spectroscopy.

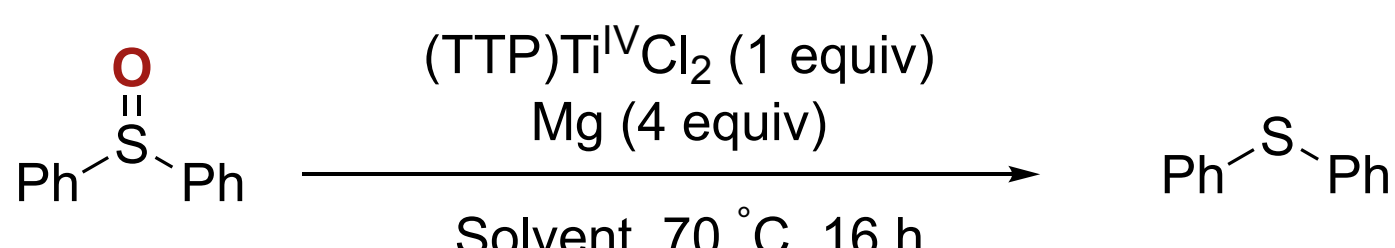

1a 0.1 mmol → **2a**

Table S1. Stoichiometric deoxygenation of **1a** with Ti^{IV} with metal reductant in various solvents.

Entry	Solvent	Conversion, 1a (%)	Yield, 2a (%)
1	MeCN	70	61
2	THF	51	54
3	DME	79	77
4	Toluene	0	0
**5**	**DMA**	**100**	**95**

3.2. Optimization of electrochemical deoxygenation of sulfoxides

General Procedure ***(Condition A)***: The reaction was carried out using an H-type divided cell separated by a glass frit. In the cathodic chamber, diphenyl sulfoxide (**1a,** 101 mg, 0.5 mmol), (TTP)$Ti^{IV}O$ (18.3 mg, 5 mol%, 0.025 mmol), triphenyl borate (435 mg, 3 equiv, 1.5 mmol), $TBAPF_6$ (387 mg, 0.2 M, 1 mmol) were dissolved in DMA (5 mL) and the chamber was equipped with an RVC (30 PPI, 4.0 cm × 1.0 cm × 0.6 cm, ~3.0 cm was immersed in the solution) as the working electrode and $Ag/AgNO_3$ as a reference electrode. In the anodic chamber, $TBAPF_6$ (387 mg, 0.2 M, 1 mmol) and triethylamine (209 µL, 3 equiv, 1.5 mmol) were dissolved in DMA (5 mL) and the chamber was equipped with a graphite rod (diameter: 0.6 cm; immersed height: ~1.5 cm) as an anode. Electrolysis at a constant potential of −1.8 V was carried out at 70 °C with magnetic stirring. The reactions were terminated when the magnitude of the current was less than 200 µA. The reaction mixture was analyzed by ^1H NMR spectroscopy using 1,3,5-trimethoxybenzene as an internal standard (1 equiv to substrate).

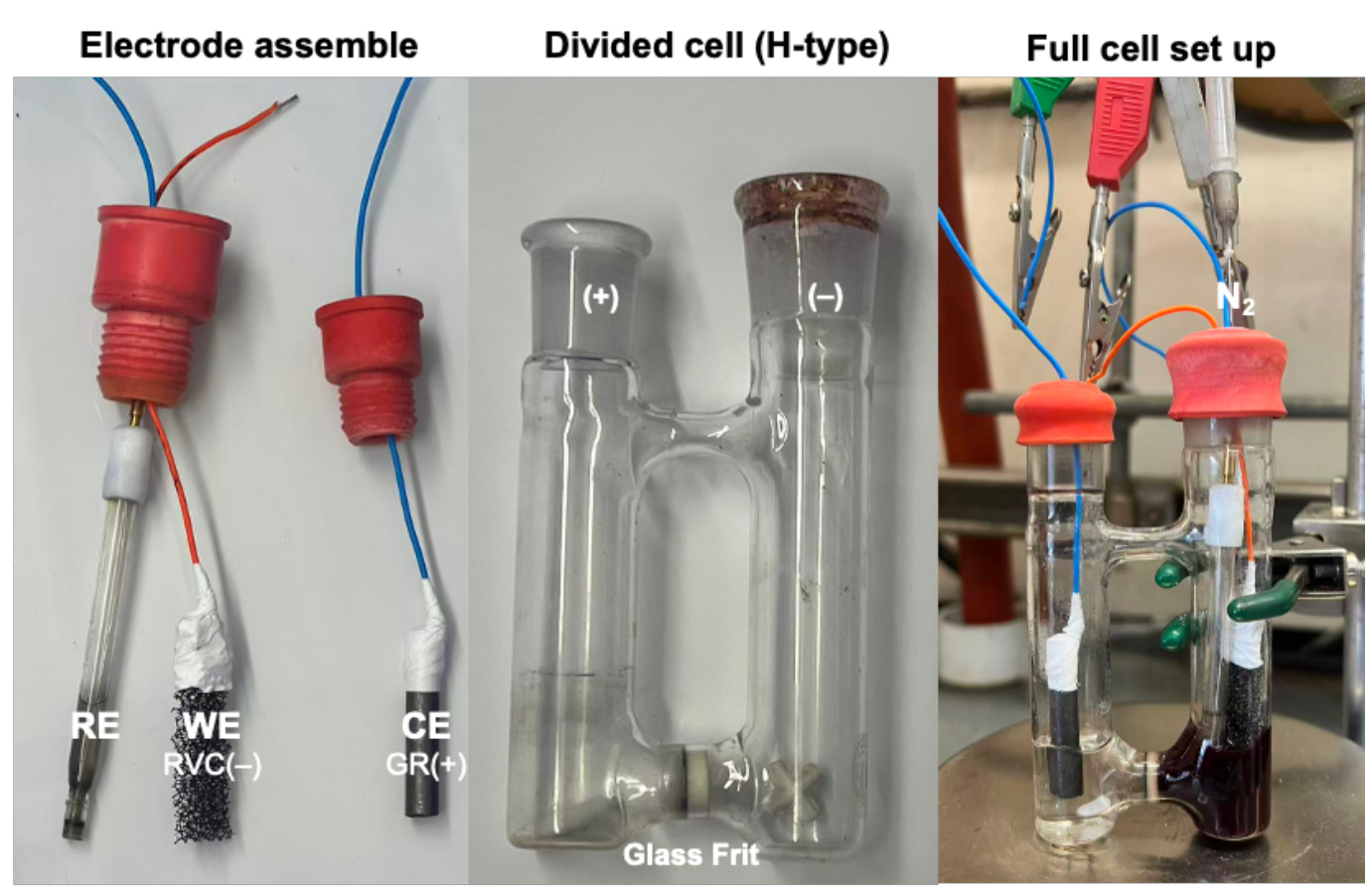

Figure S1. Electrochemical cell setup in divided cell (RE: reference electrode; WE: working electrode; CE: counter electrode).

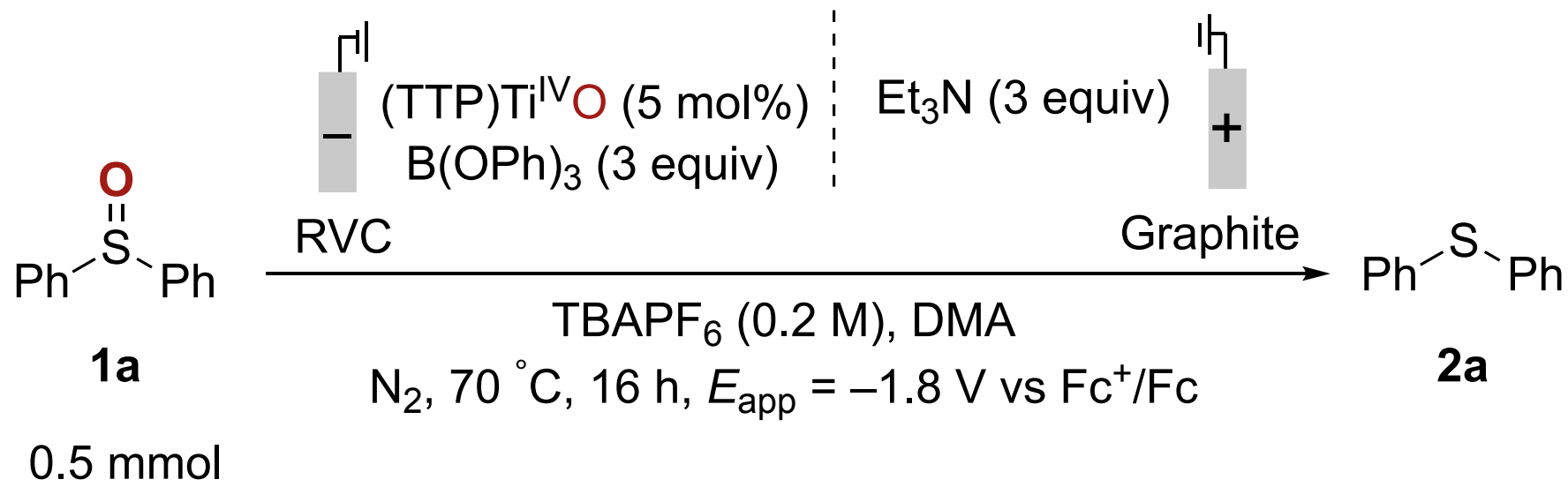

Table S2. Optimization of the electrochemical reaction conditions.

Entry	Deviation from standard conditions	Conversion, 1a (%)	Yield, 2a (%)
1	none	100	99
2	1 mol% (TTP)Ti^{IV}O	60	57
3	10 mol% (TTP)Ti^{IV}O	100	99
4	No (TTP)Ti^{IV}O	99	0
5	(TTP)Ti^{IV}Cl$_2$ instead of (TTP)Ti^{IV}O	100	99
6	Undivided cell	65	62
7	Undivided cell, Zn (+), no Et$_3$N	55	50
8	Graphite rod (–) instead of RVC (–)	67	66
9	Ni foam (–) instead of RVC (–)	100	95
10	Zn (+) instead of Graphite rod (+), no Et$_3$N	100	99
11	0.5 equiv of B(OPh)$_3$	10	10
12	2 equiv of B(OPh)$_3$	90	88

3.3. Screening of Lewis acids for *sulfoxides deoxygenation*

Chemical deoxygenation:

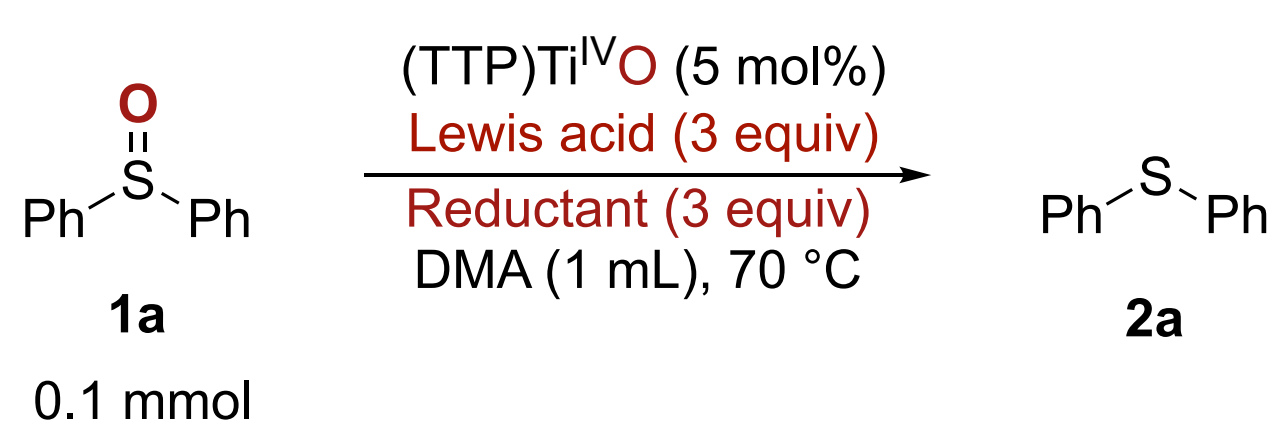

To a 1-dram vial was added diphenyl sulfoxide (20.2 mg, 0.1 M, 0.1 mmol), (TTP)Ti^{IV}O (3.7 mg, 5 mol%, 0.005 mmol), reductants (3 equiv, 0.3 mmol), Lewis acidic additives (3 equiv, 0.3 mmol) and DMA (1 mL) in the N$_2$-filled glovebox. Reactions were then removed from glovebox stirred in a sand bath on a stir plate at 800 rpm and 70 °C overnight. 1,3,5-trimethoxybenzene was added as an internal standard (1 equiv, 0.1 mmol) for analysis by ^{1}H NMR spectroscopy.

Table S3. Yields of **2a** under chemical catalytic deoxygenation of **1a** with various Lewis acids using Zn or Mn as reductant.

Lewis acid	Yield with Zn, 2a (%)	Yield with Mn, 2a (%)
none	2	4
$MgCl_2$	43	42
$AlCl_3$	49	47
Me_3SiCl	53	56
Et_3SiCl	41	46
BPh_3	56	65
$B(OMe)_3$	17	21
$B(OCH_2CF_3)_3$	64	83
**$B(OPh)_3$**	**99**	**99**

Notes: All three metal reductants lead to quantitative (TTP)Ti^{IV}O-catalyzed deoxygenation of diphenyl sulfoxide in the presence of 3 equiv of $B(OPh)_3$. The open circuit potential (OCP) of the metal reductants compared with the reduction features of (TTP)Ti^{IV}O + $B(OPh)_3$ support that efficient catalytic turnover can be achieved.[11]

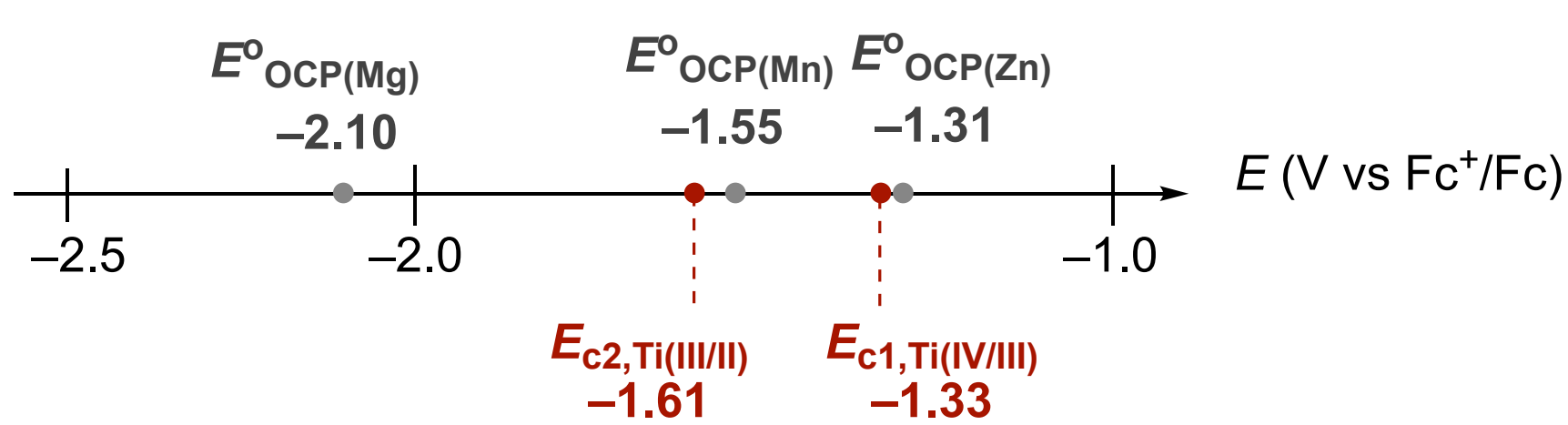

Figure S2. Comparison of the $Ti^{(IV/III)}$ and $Ti^{(III/II)}$ reduction potentials with reported open circuit potentials of metal reductants.

3.4. Screening of applied potentials

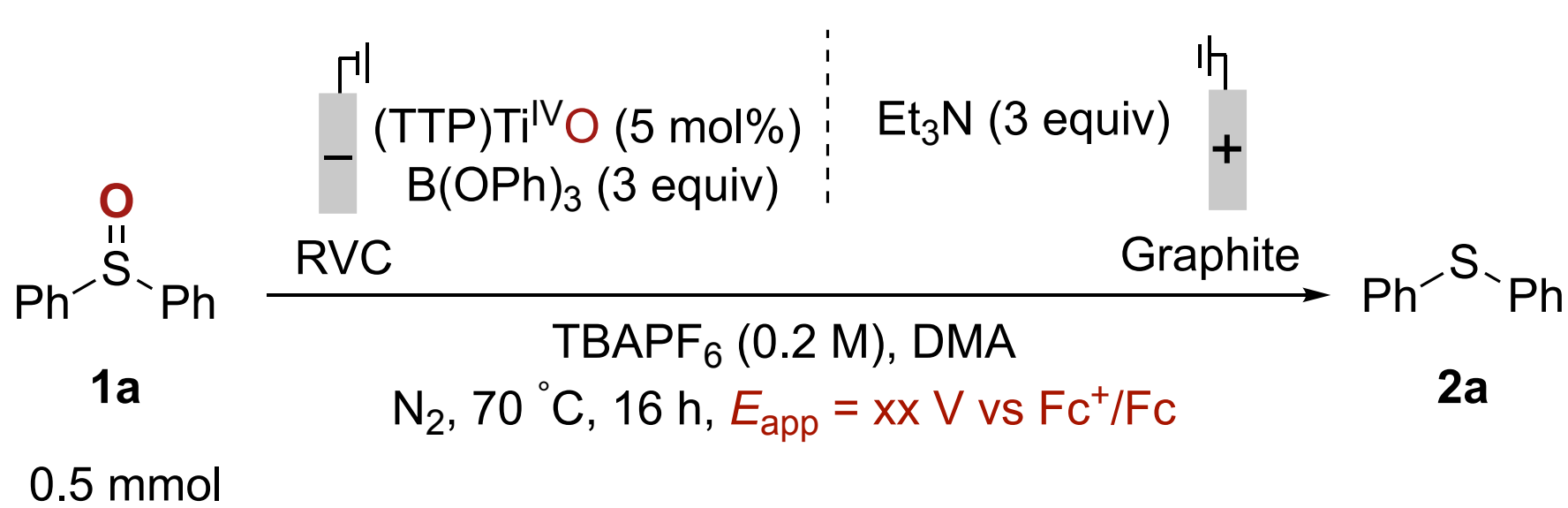

The reaction was carried out following the general procedure described in section 3.2. Conditions: Divided cell, cathodic chamber, 0.5 mmol **1a**, 5 mol % (TTP)Ti^{IV}O, 5 mL DMA, 3 equiv $B(OPh)_3$, 0.2 M $TBAPF_6$;

anodic chamber, 5 mL DMA, 0.2 M $TBAPF_6$, 3 equiv Et_3N. (RVC working electrode, graphite counter electrode, $Ag/AgNO_3$ reference electrode). Constant potential was applied at the following values when the magnitude of the current was less than 200 μA. The reaction mixture was analyzed by ^1H NMR spectroscopy using 1,3,5-trimethoxybenzene as an internal standard (0.33 equivalent of **1a**).

Table S4. Screening of reduction potentials under electrochemical conditions.

Entry	E_{app} (V vs Fc^+/Fc)	Conversion, 1a (%)	Yield, 2a (%)
1	−1.8	100	99
2	−1.6	100	99
3	−1.5	91	90
4	−1.3	80	81
5	−1.0	17	15

3.5. Electrochemical sulfoxides deoxygenation

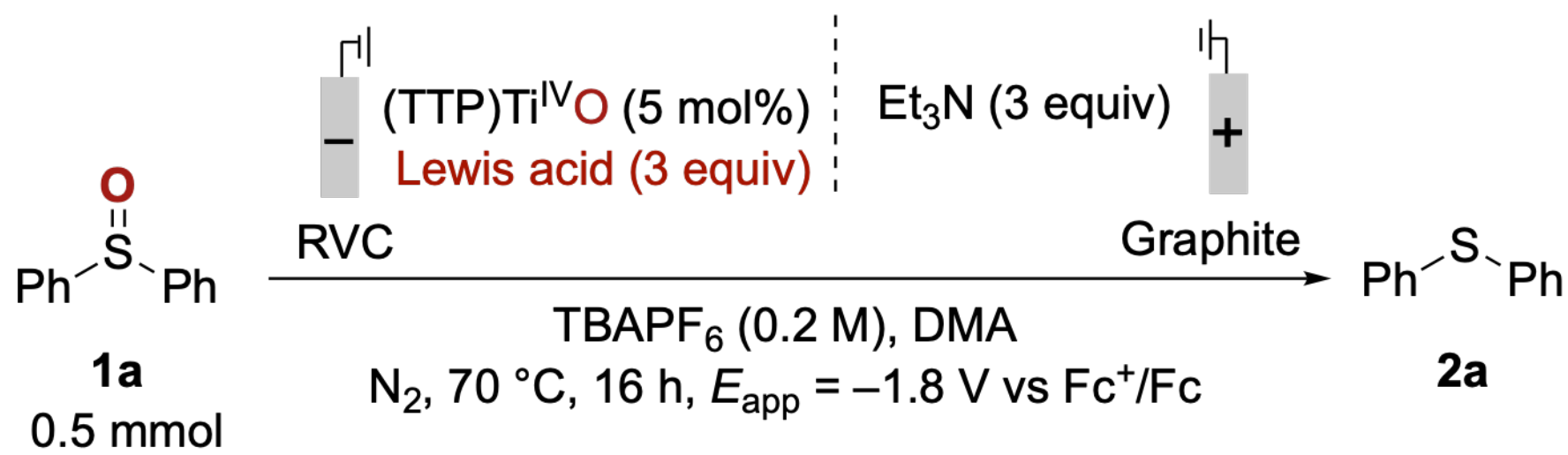

The reaction was carried out using an H-type divided cell separated by a glass frit. In the cathodic chamber, diphenyl sulfoxide (101 mg, 0.5 mmol), (TTP)Ti^{IV}O (18.3 mg, 5 mol%, 0.025 mmol), Lewis acidic additives (3 equiv, 1.5 mmol), $TBAPF_6$ (387 mg, 0.2 M, 1 mmol) were dissolved in DMA (5 mL) and the chamber was equipped with an RVC (30 PPI, 4.0 cm × 1.0 cm × 0.6 cm, ~3.0 cm was immersed in the solution) as the working electrode and $Ag/AgNO_3$ as a reference electrode. In the anodic chamber, $TBAPF_6$ (387 mg, 0.2 M, 1 mmol) and triethylamine (209 μL, 3 equiv, 1.5 mmol) were dissolved in DMA (5 mL) and the chamber was equipped with a graphite rod (diameter: 0.6 cm; immersed height: ~1.5 cm) as an anode. A constant potential electrolysis at −1.8 V was carried out at 70 °C with magnetic stirring. The reactions were terminated when the magnitude of the current was less than 200 μA. The reaction mixture was analyzed by ^1H NMR spectroscopy using 1,3,5-trimethoxybenzene as an internal standard (0.33 equivalent of **1a**).

Table S5. Screening of Lewis acids under electrochemical conditions.

Entry	Lewis acid	Conversion, 1a (%)	Yield, 2a (%)	Charge (F/mol)	F.E. (%)
1	none	5	4	0.38	20
2	$MgCl_2$	40	24	0.64	75
3	$AlCl_3$	29	27	1.55	35
4	Me_3SiCl	51	51	2.42	42
5	Et_3SiCl	50	46	2.56	36
6	BPh_3	83	83	2.26	74
7	$B(OMe)_3$	10	9	1.06	17
8	$B(OCH_2CF_3)_3$	55	60	2.21	54
**9**	**$B(OPh)_3$**	**100**	**>99**	2.23	90

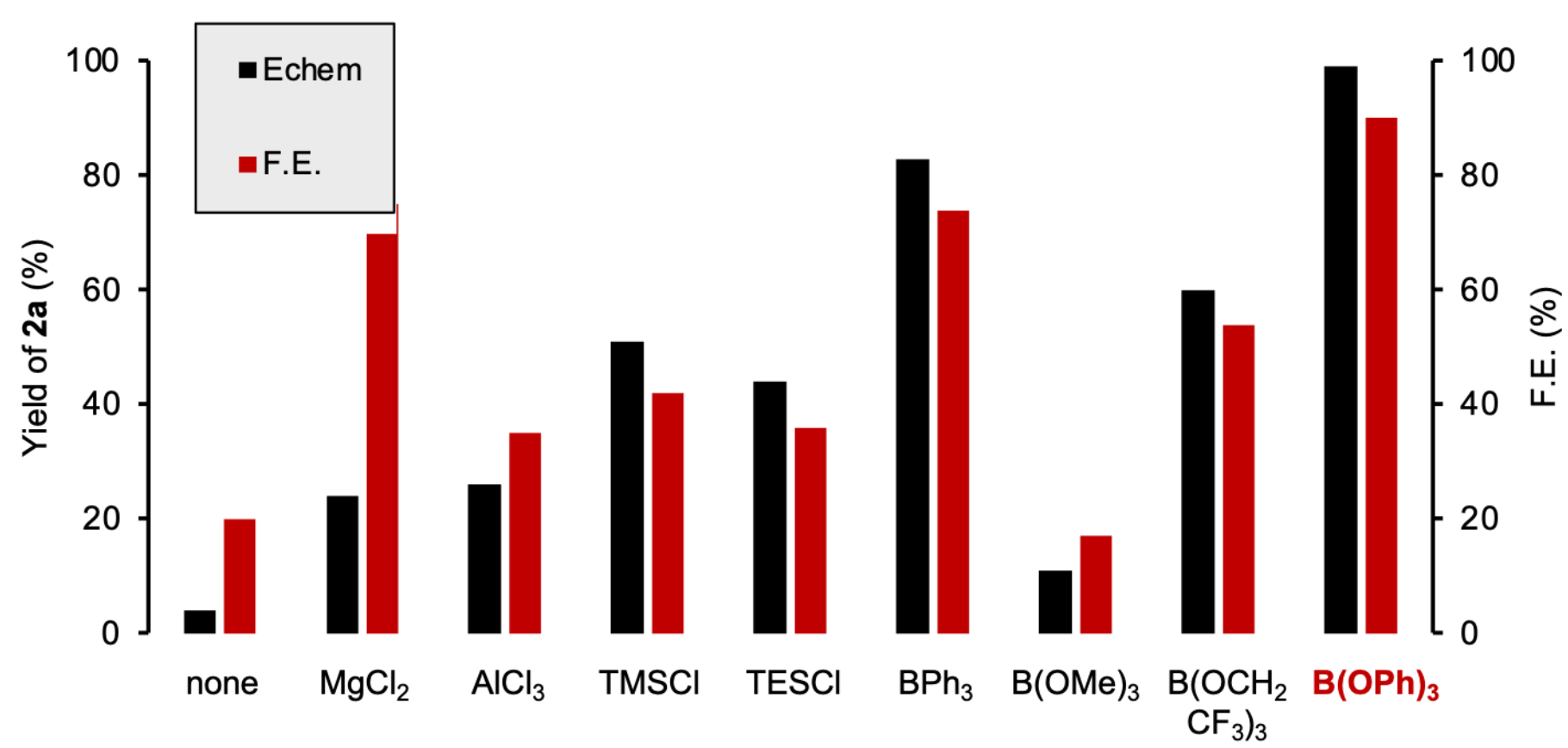

Figure S3. Yield of **2a** and calculated Faradaic efficiencies (F.E.) under electrochemical conditions using various Lewis acids.

3.6. Time course of electrochemical deoxygenation of Ph_2SO

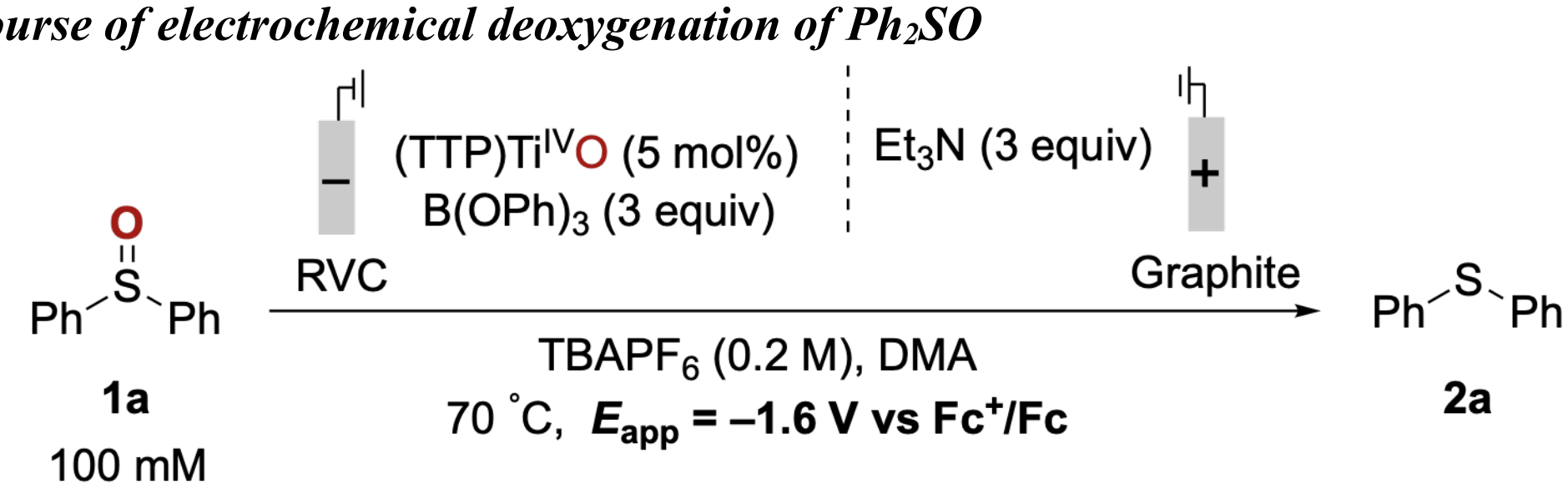

The reaction was carried out following the general procedure described in section 3.2 (with $E_{app} = -1.6$ V vs Fc^+/Fc). The electrolysis was set using a CHI 600 potentiostat where the charge passed can be monitored. An aliquot of 50 μL from the cathodic chamber was taken at a span of 0.2 F/mol (from 0 to 2.2 F/mol), which was diluted by $CDCl_3$ to 500 μL and transferred into an NMR tube for ^1H NMR spectroscopy analysis using 1,3,5-trimethoxybenzene as an internal standard (0.33 equivalent of **1a**) for each data points.

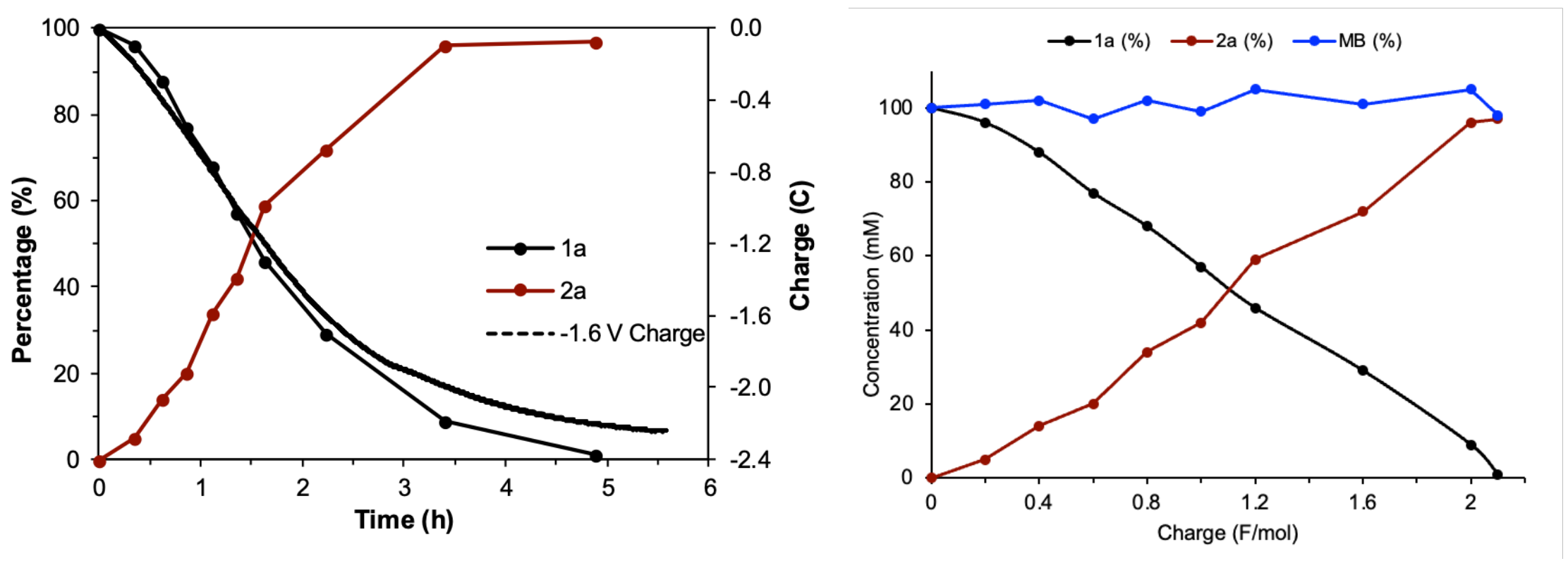

Figure S4. Deoxygenation of **1a** under standard reaction conditions. Left: Percent remaining of **1a** (solid black) and percent yield of **2a** (solid red) vs time. The dashed black line shows charge passed over time. Right: Percent remaining of **1a** (solid black) and percent yield of **2a** (solid red) vs charge passed. The mass balance is shown in blue. The amounts of **1a**/**2a** were determined via ^{1}H NMR spectroscopy with 1,3,5-trimethoxybenzene (0.167 mmol) as an internal standard.

4. CV Studies

4.1. General CV considerations

All cyclic voltammetry (CV) experiments were carried out using Pine WaveNow PGstat and CH Instrument 600. CV experiments were carried out using a three-electrode cell configuration with a glassy carbon (GC) working electrode (3 mm diameter, purchased from BASi, part no. MF-2012) and a custom-made platinum coil counter electrode (3 cm, 5–7 spiral coils, purchased from MilliporeSigma, 0.5 mm diameter, 99.99% metals basis). The working electrode potentials were measured against an Ag/$AgNO_3$ reference electrode purchased from BASi (internal solution, 0.2 M $TBAPF_6$ and 0.01 M $AgNO_3$ in DMF, part no. MF-2062). The redox potential of ferrocenium/ferrocene (Fc^+/Fc) was measured (under the same experimental conditions) and used to provide an internal reference. The potential values were then adjusted relative to Fc^+/Fc, and electrochemical studies in organic solvents were recorded accordingly. The GC working electrode was polished with alumina powder (5 µm) before and after each experiment. The volume of the CV solution was 5 mL with 0.2 M $TBAPF_6$ dissolved as a supporting electrolyte.

4.2. (TTP)Ti^{IV}O in DMA

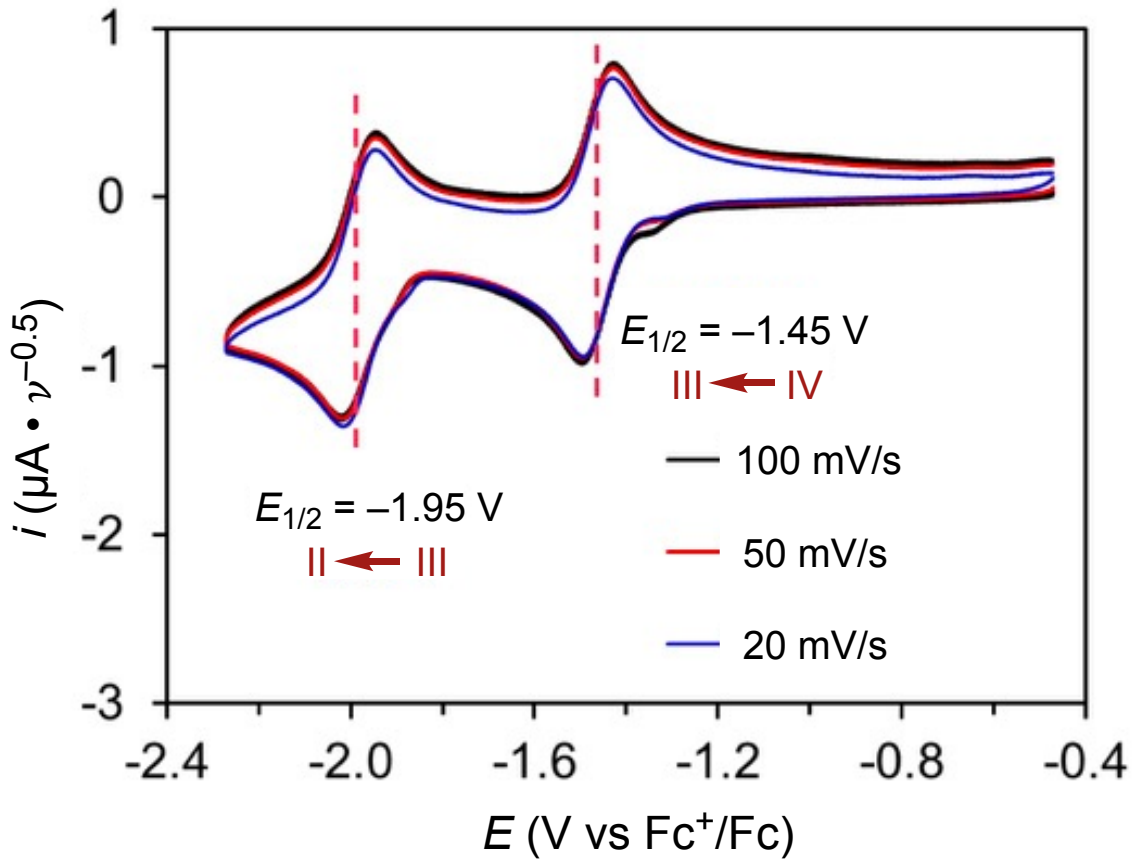

Figure S5. Scan rate normalized CVs of (TTP)Ti^{IV}O (1 mM in DMA) at varying scan rates: 100 mV/s (black); 50 mV/s (red); 20 mV/s (blue).

Notes: Two reversible peaks were observed at all scan rates, suggesting no structural change of (TTP)Ti^{IV}O upon 1 e$^-$ or 2 e$^-$ reduction.

4.3. (TTP)Ti^{IV}O + B(OPh)$_3$ in DMA

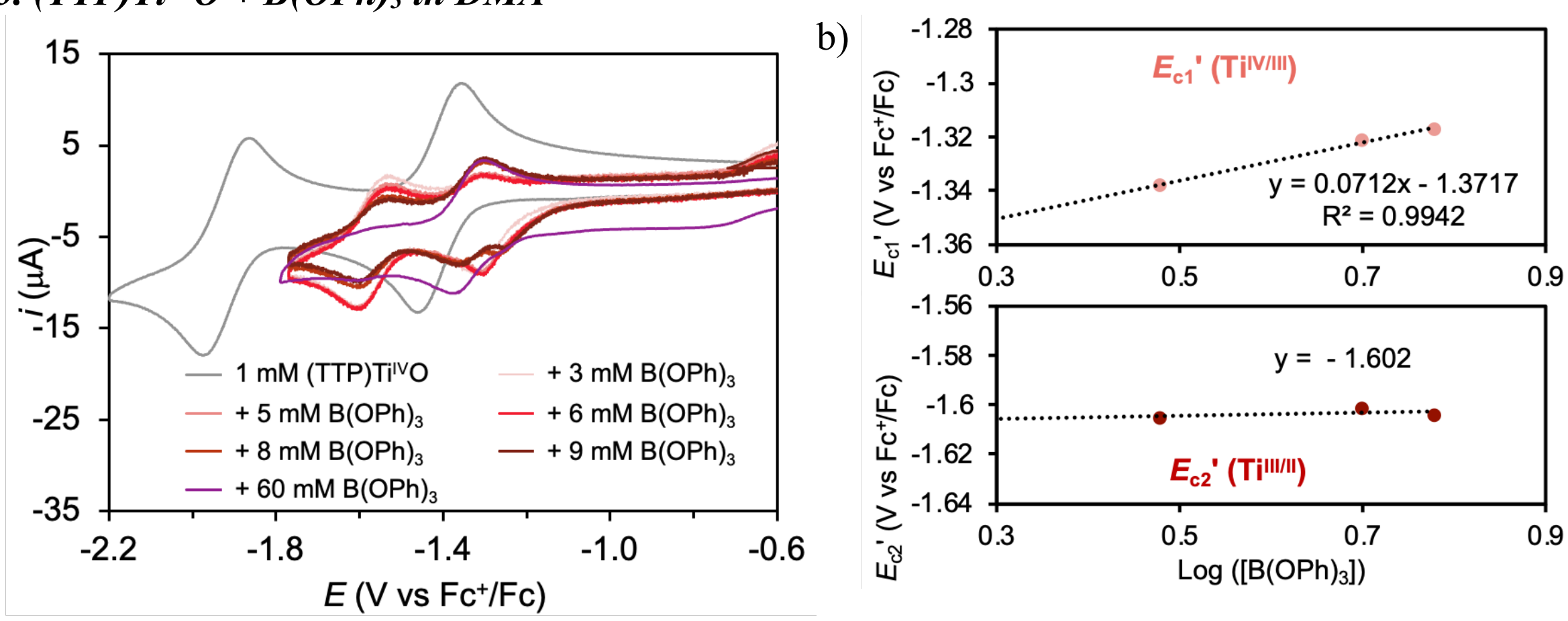

Figure S6. CV studies of (TTP)Ti^{IV}O (1 mM in DMA, grey) with B(OPh)$_3$: a) 3 mM, 5 mM, 6 mM, 8 mM, 9 mM (from light red to dark red), and 60 mM B(OPh)$_3$ (purple); b) a Nernstian dependence of the reduction potential (E_{c1}', top; and E_{c2}', bottom) on [B(OPh)$_3$]. Scan rate: 100 mV/s.

Notes: In **Figure S6a**, an anodic shift was observed for both reduction peaks (−1.95 V → −1.61 V, −1.45 V → −1.33 V). The first reduction peak became less reversible, suggesting that an irreversible reaction likely occurs for (TTP)Ti^{IV}O upon reduction in the presence of a Lewis acid (e.g. E_rC_i mechanism).[12]

In addition, the first reduction peak potential (E_{c1}') demonstrated a Nernstian anodic shift with increasing concentrations of B(OPh)$_3$ (up to 60 mM), suggesting a Lewis acid-coupled electron transfer process occurs at this reduction potential.[13] A slope of 71.2 mV/dec is shown in **Figure S6b**. Based on the Nernstian equation, the reduction potential (E_{c1}') is linearly dependent on the log[B(OPh)$_3$] as a Lewis acid. The number of B(OPh)$_3$ involved in the first reduction process per electron is calculated to be 1.20 (close to 1).

In contrast, the second reduction peak potential (E_{c2}') did not shift with increase of concentration of $B(OPh)_3$; therefore, it is likely that $B(OPh)_3$ is not involved in the second reduction step:

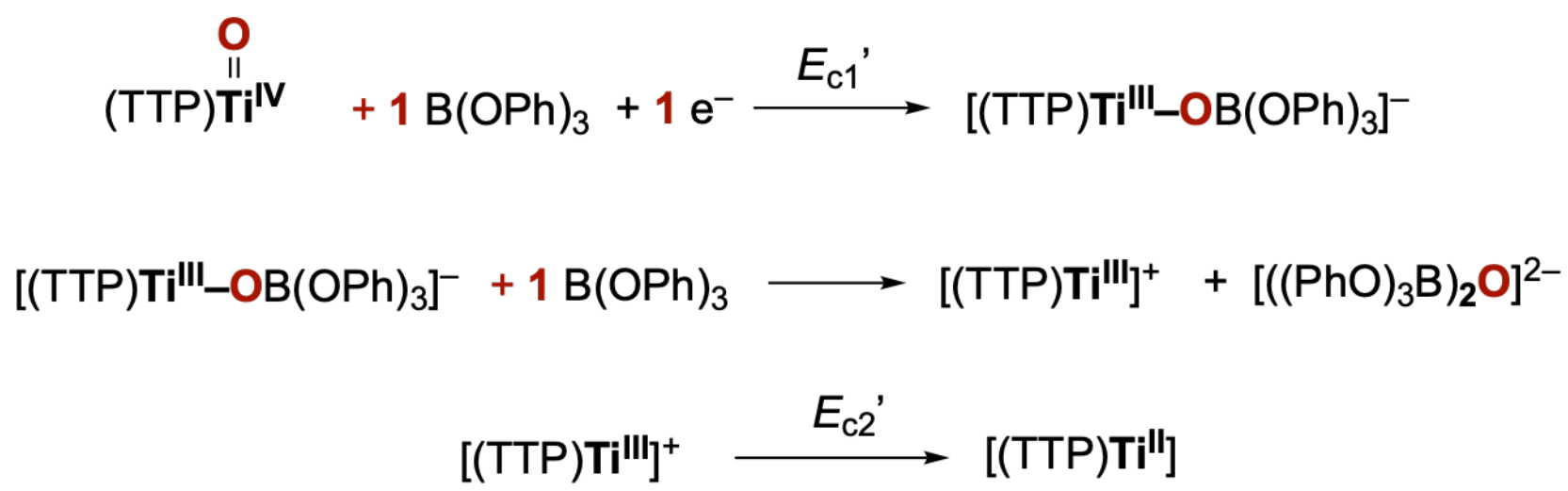

$$E\left(Ti^{(IV/III)}\right) = E^{\circ}\left(Ti^{(IV/III)}\right) + (71.2\ \mathrm{mV\,dec^{-1}})log([B(OPh)_3])$$

$$\frac{nF}{RT} = 59.2 \text{ when } n = 1$$

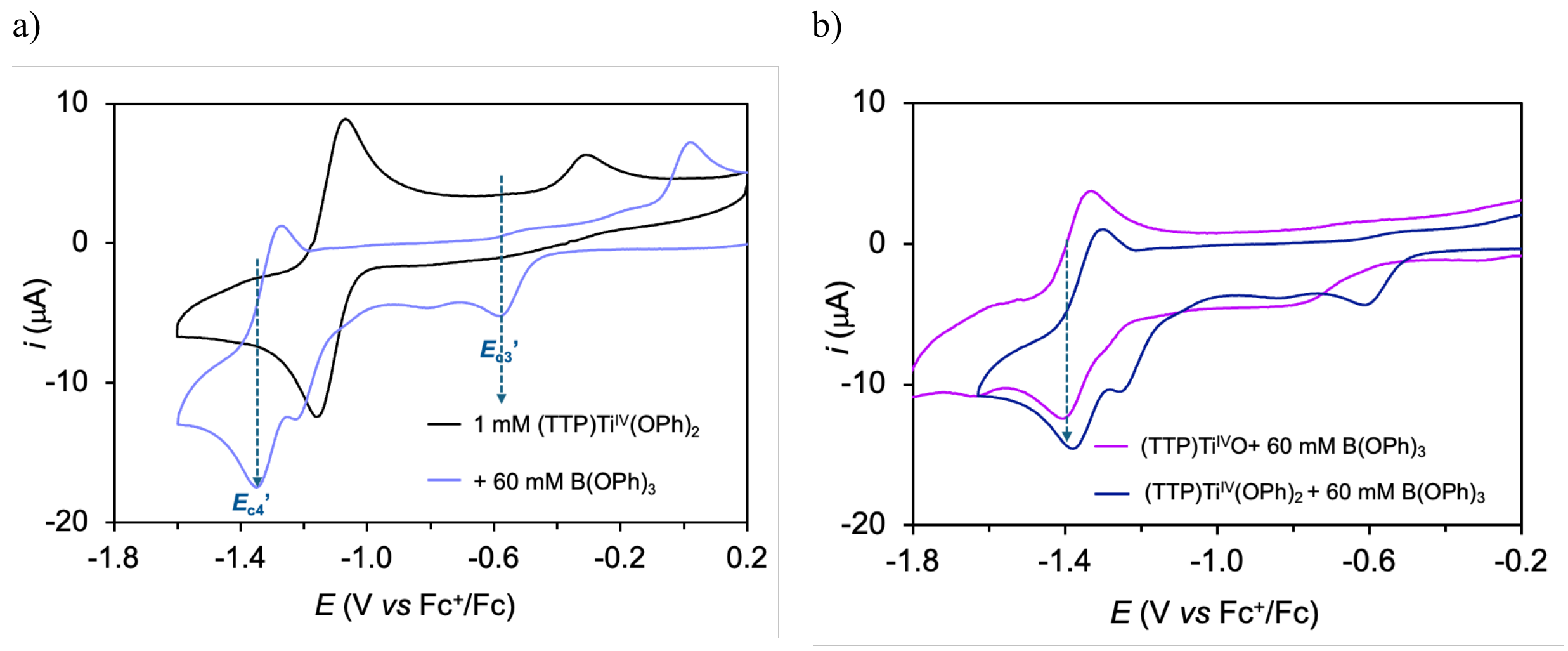

Figure S7. CV studies of $(TTP)Ti^{IV}(OPh)_2$ (1 mM in DMA, black) with $B(OPh)_3$: a) 3 mM, 15 mM, 30 mM, and 60 mM (from light blue to dark blue) $B(OPh)_3$; b) Stacked CV trace between $(TTP)Ti^{IV}O$ (1 mM, purple) and $(TTP)Ti^{IV}(OPh)_2$ (1 mM, dark blue) in the presence of $B(OPh)_3$ (60 mM). Scan rate: 100 mV/s.

Notes: We noticed that the peak at E_{c1}' is more complex at high concentrations of $B(OPh)_3$, with the reduction wave exhibiting two peaks (**Figure S6a,** 8-9 mM). When the concentration of $B(OPh)_3$ is further increased to 60 mM (60 equiv relative to $(TTP)Ti^{IV}O$, same as the ratio of $B(OPh)_3$ to [Ti] in the catalytic conditions), new reduction features at E_{c3} (−0.65 V) and E_{c4} (−1.4 V) appear (**Figure S7a**) with the peaks at E_{c1} and E_{c2} diminishing. Detailed information can be found in Section 6.1, which shows that $(TTP)Ti^{IV}(OPh)_2$ is the dominant species due to redox-neutral deoxygenation of $(TTP)Ti^{IV}O$ at high concentration of $B(OPh)_3$. In addition, The 2nd reduction peak potential of $(TTP)Ti^{IV}(OPh)_2$ with 60 equivalents of $B(OPh)_3$ is similar to the 2nd reduction peak of $(TTP)Ti^{IV}O$ with $B(OPh)_3$.

As for the reduction of (TTP)Ti^{IV}(OPh)$_2$, the 1st reduction peak is observed with a potential~ –1.2 V and a reversible feature was shown. In the presence of 60 mM of B(OPh)$_3$ (blue trace), the 1st reduction peak disappears, with a new reduction peak occurring at less reducing potential (~ –0.6 V) and an 2nd peak around –1.4 V, suggesting B(OPh)$_3$-promoted loss of OPh ligand at the reduction step:

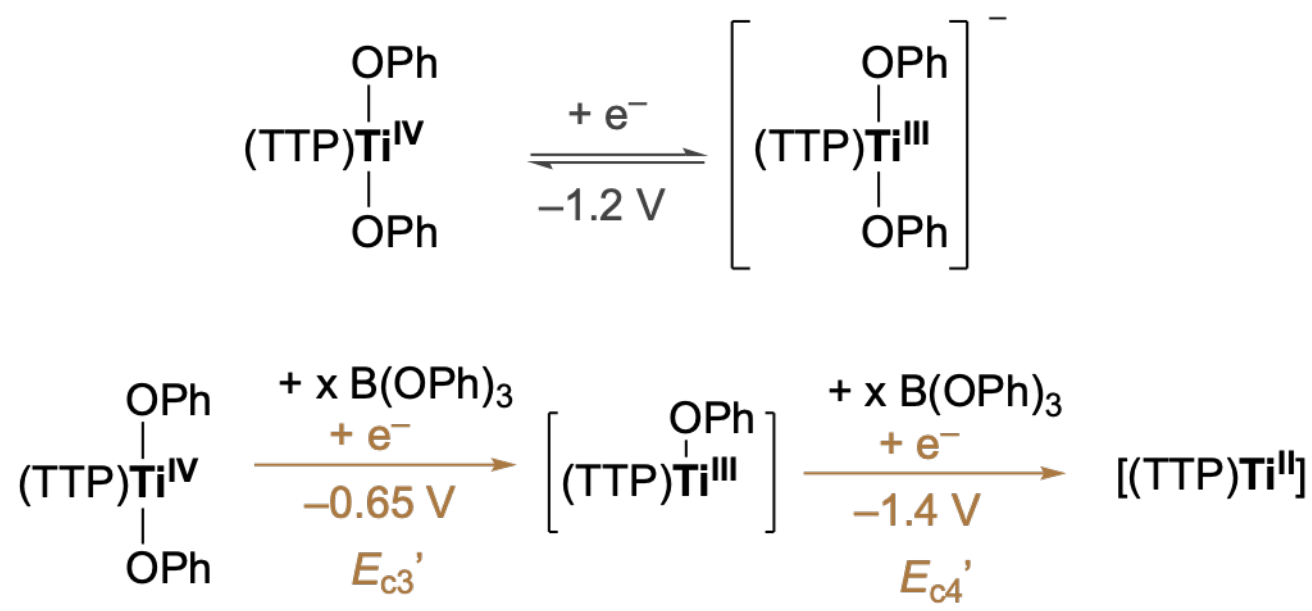

4.4. (TTP)Ti^{IV}O + BPh$_3$ in DMA

To avoid transmetalation between (TTP)Ti and B(OPh)$_3$, BPh$_3$ as an alternative Lewis acid that demonstrated deoxygenative turnover reactivity (**Figure 2A**) was used for CV studies. Given the less reactive B–C bond, we postulate that it might not undergo transmetalation that perplex the analysis.

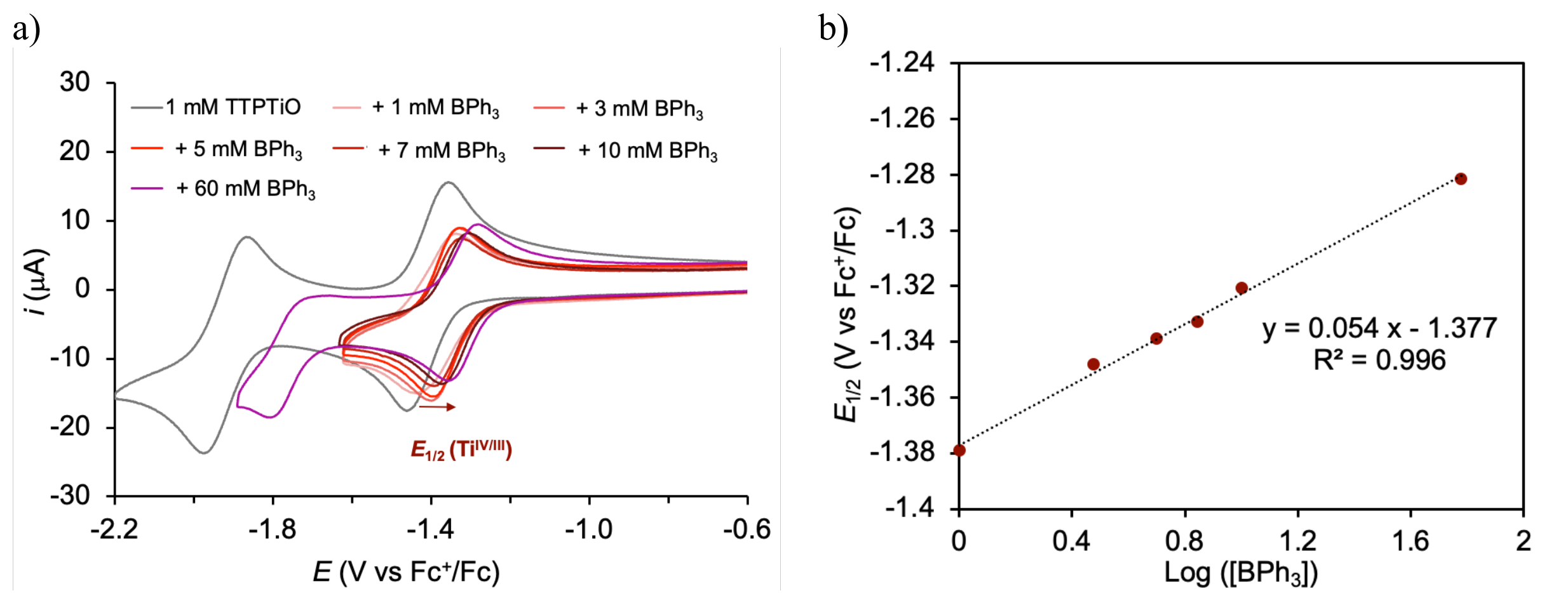

Figure S8. CV studies of (TTP)Ti^{IV}O (1 mM in DMA, grey) with BPh$_3$: a) 1 mM, 3 mM, 5 mM, 7 mM, and 10 mM (from light red to dark red) and 60 mM BPh$_3$ (purple); b) a Nernstian dependence of the $E_{1/2}$ on log[BPh$_3$]. Scan rate: 100 mV/s.

Conclusion: Addition of BPh$_3$ as the Lewis acid (1 to 60 equiv) to (TTP)Ti^{IV}O shows an improved correlation of $E_{1/2}$ (obtained from the average of reversible E_{c1} and E_{a1}) against log[BPh$_3$] on the 1st reduction feature of CV trace in **Figure S8**. The slope of is 54 mV/dec, which is characteristic of 1-Lewis acid, 1-electron (59.2 mV/dec theoretically), supporting a Nernstian shift that consistent with Lewis acid-coupled electron-transfer pathway:

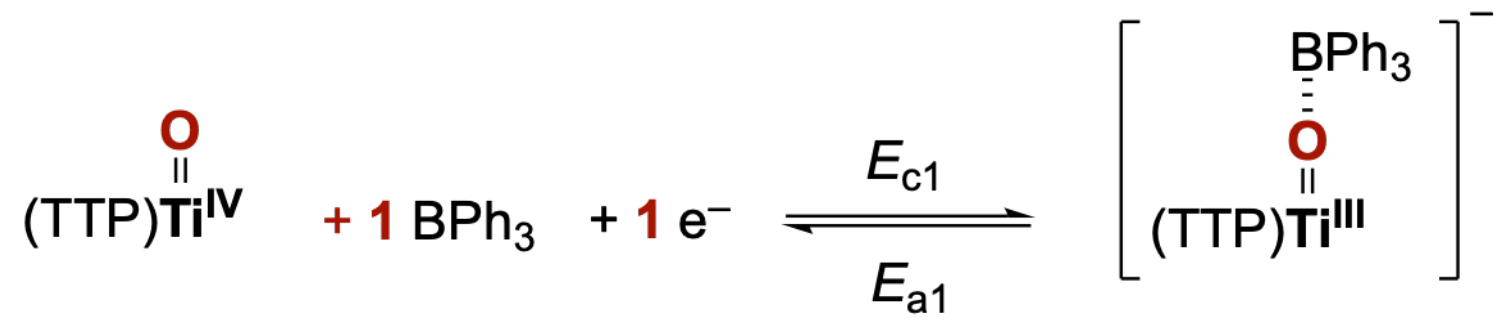

$$E\left(Ti^{(IV/III)}\right) = E^{\circ}\left(Ti^{(IV/III)}\right) + (54.4\ \mathrm{mV\ dec^{-1}})log([BPh_3])$$

$$\frac{nF}{RT} = 59.2 \text{ when } n = 1$$

At the 2nd reduction feature with BPh_3 (Ti^{III} to Ti^{II}), a quasi-reversible peak was shown. Electrolysis results (Table S5, entry 6) suggest that the Ti^{II} species generated at 2nd reduction feature can promote catalytic deoxygenation of Ph_2SO.

4.5. (TTP)Ti^{IV}O + B(OPh)$_3$ in THF

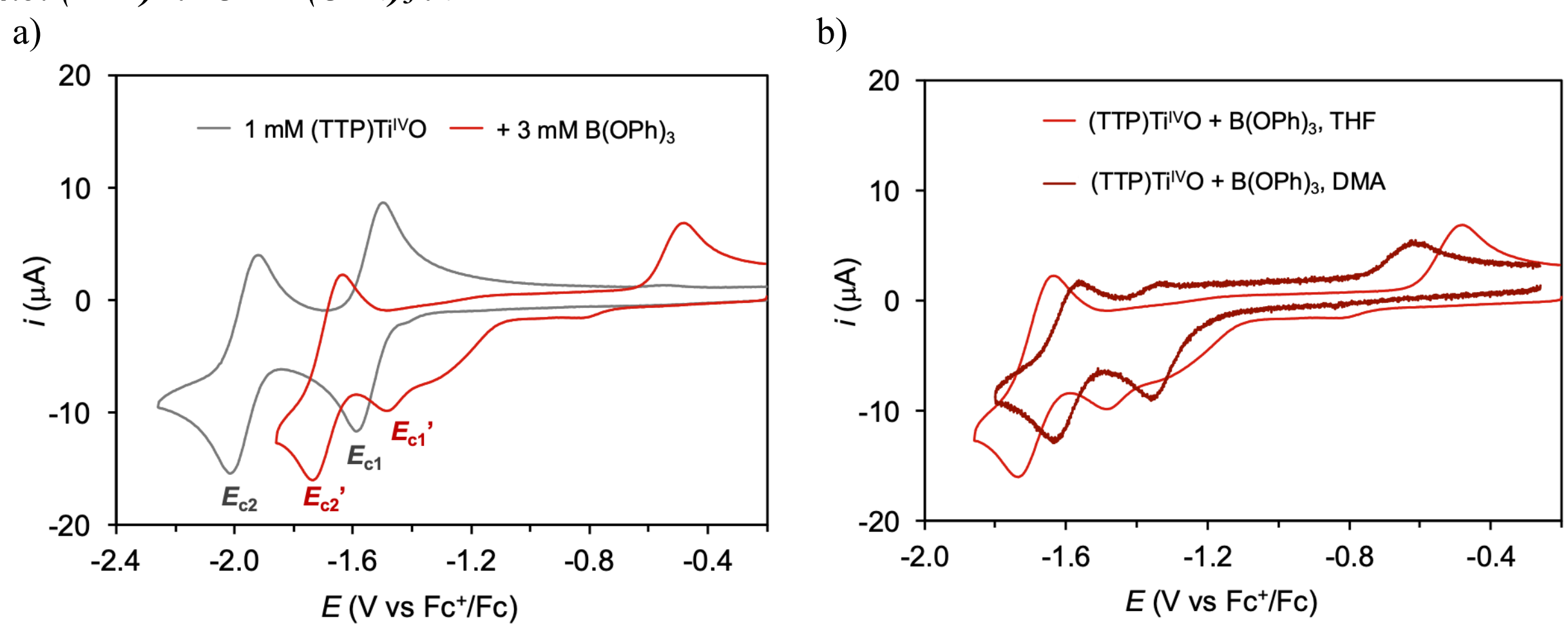

Figure S9. a) CV studies of (TTP)Ti^{IV}O (1 mM, grey) and with B(OPh)$_3$ (3 mM, red) in THF; b) Comparison between (TTP)Ti^{IV}O (1 mM) with B(OPh)$_3$ (3 mM) in THF (red) and DMA (dark red).

Notes: CV of (TTP)Ti^{IV}O in the absence and presence of B(OPh)$_3$ in THF showed similar reduction features compared with that in DMA (**Figure S6a**). However, the reduction peak potential E_{c1}' (–1.45 V vs Fc^+/Fc) and E_{c2}' (–1.73 V vs Fc^+/Fc) in THF were more reducing than E_{c1}' and E_{c2}' in DMA as shown in **Figure S8b**. This can be attributed to the fact that low-valent Ti species (especially Ti^{II}) with THF coordinated might be more reducing than that with DMA coordinated.

5. Coulometry Studies: Reduction of (TTP)Ti^{IV}O

5.1 Reduction of (TTP)Ti^{IV}O in the absence and presence of B(OPh)$_3$

Procedure: The reaction was carried out using an H-type divided cell (separated by a glass frit). In the cathodic chamber, (TTP)Ti^{IV}O (36.6 mg, 10 mM, 0.005 mmol), B(OPh)$_3$ (43.5 mg, 3.00 equiv, 0.015 mmol), and $TBAPF_6$ (387 mg, 0.2 M, 1 mmol) were dissolved in DMA (5 mL) and equipped with a RVC (30 PPI, 4.0 cm × 1.0 cm × 0.6 cm, ~3.0 cm was immersed in the solution) as the working electrode and a Ag/$AgNO_3$

reference electrode. In the anodic chamber, $TBAPF_6$ (387 mg, 0.2 M, 1 mmol) and triethylamine (21 µL, 3.00 equiv, 0.15 mmol) were dissolved in DMA (5 mL) and equipped with a graphite rod anode. A constant potential was applied at room temperature with magnetic stirring. The reactions were terminated when I was greater than −200 µA (i.e., $|I| < 200$ µA). The chronopotentiometry and coulometry data were recorded, and the cathodic solutions were kept for isolation of low-valent Ti complexes as described in Section 2 above.

Analysis: In the CVs of $(TTP)Ti^{IV}O$ in the absence and presence of 3 equiv $B(OPh)_3$ (**Figure 3A**), two sequential reduction peaks were observed. To verify the e^- stoichiometry, constant potential electrolysis were carried out for solutions of $(TTP)Ti^{IV}O$ in the absence and presence of $B(OPh)_3$.The charge passed was converted from C to F/mol of $(TTP)Ti^{IV}O$ to determine the number of the electrons involved in the reduction at a given applied potential (1 F/mol = −4.82 C in this context).

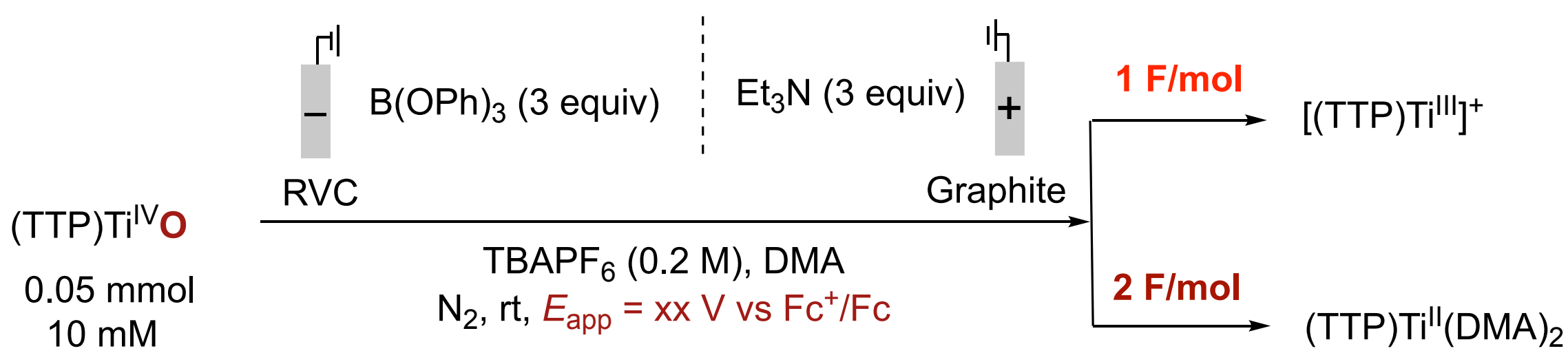

Notes: 1 F/mol and 2 F/mol were passed in the presence of $B(OPh)_3$ at −1.3 V and −1.6 V, respectively. Therefore, these two conditions were selected for further characterization and isolation of low-valent Ti species. The applied potentials that converged on integer F/mol values are consistent with the CVs in **Figure 3A**, supporting the reduction assignments of $Ti^{IV/III}$ at −1.33 V and $Ti^{III/II}$ at −1.61 V.

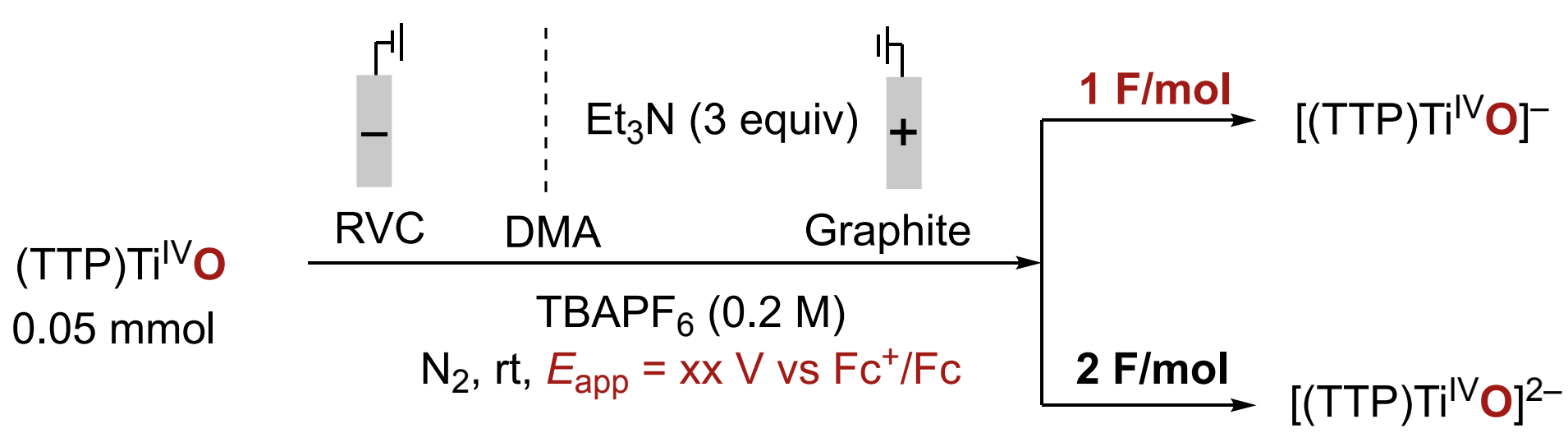

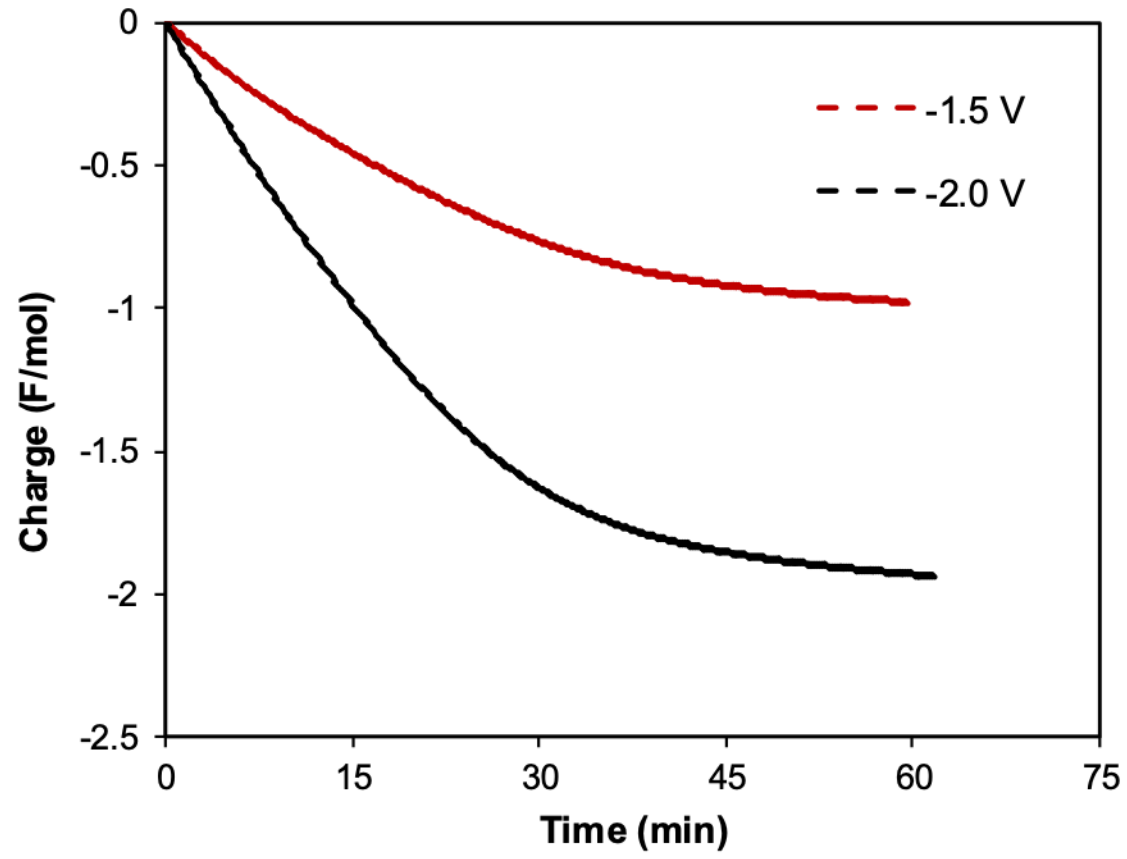

Figure S10. Coulometry using constant potential electrolysis of (TTP)Ti^{IV}O at various applied potentials in the absence of $B(OPh)_3$.

Notes: The Kadish group previously reported characterization of reduced (TPP)Ti^{IV}O species and concluded that Titanium remains in the Ti^{IV} oxidation state with a radical anion or dianion located on the porphyrin ligand.[14]

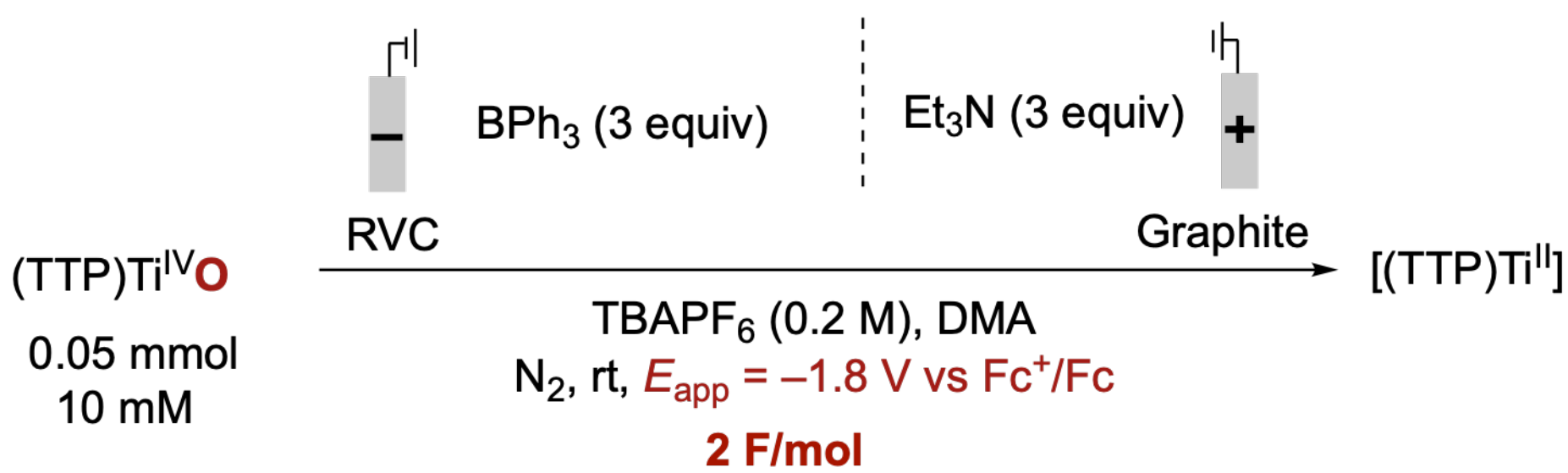

Notes: Reduction of (TTP)Ti^{IV}O with BPh_3 at –1.8 V vs Fc^+/Fc (according to the CV studies shown in **Figure S8a**) also shows a charge passed ~2 F/mol, consistent with our hypothesis that Ti^{II} species can be formed at the 2nd reduction peak as discussed in section 4.4.

5.2 Time course of stoichiometric oxo-transfer of Ph_2SO by Ti^{II} and Ti^{III}

To determine if the electrochemically generated low-valent Ti species described above (Section 5.1) can engage in oxygen atom transfer (OAT) from organic substrates and if the stoichiometry is consistent with the assignments of Ti^{III} and Ti^{II} from coulometry, we added 10 equiv **1a** (Ph_2SO) to each solution after electrolysis and monitored the reaction over 1 h of reaction at room temperature (see **Figure 3D** for details). Aliquots (50 µL) were taken from the reaction solution at t = 5 min, 10 min, 30 min, 50 min, and 60 min and were immediately quenched with 50 µL of DMSO to kill any remaining low-valent Ti^{II} or Ti^{III} species in the samples. Each aliquot was diluted to 500 µL with $CDCl_3$ and analyzed by ^{1}H NMR spectroscopy with 1,3,5-trimethoxybenzene as an internal standard (1 equivalent, added after electrolysis and before the time course started).

Notes: Both Ti^{II} and Ti^{III} species generated electrochemically can engage in OAT from **1a**. Additionally, the stoichiometry of the reactions (5.1 mM of Ph_2S generated respect to "Ti^{III}"; 9.4 mM of Ph_2S generated respect to "Ti^{II}") is consistent with the e^- stoichiometry observed in coulometry.

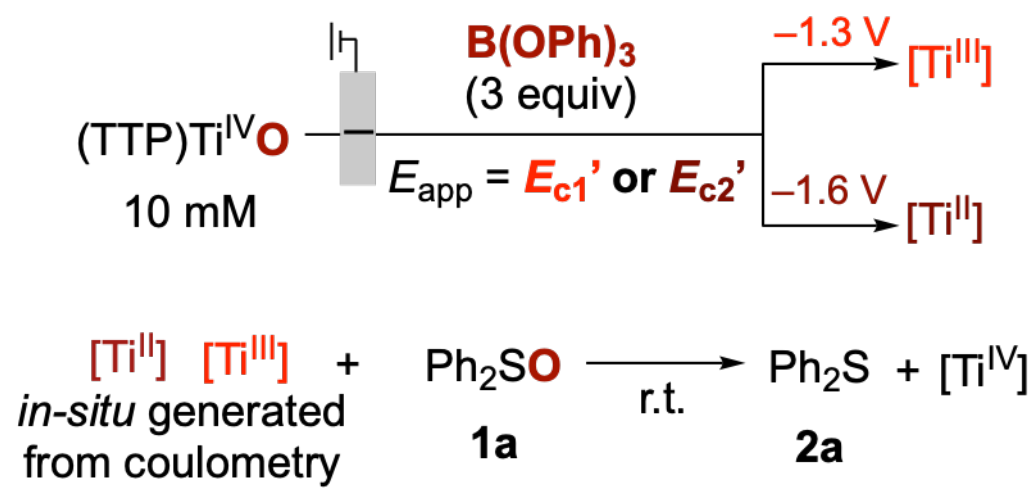

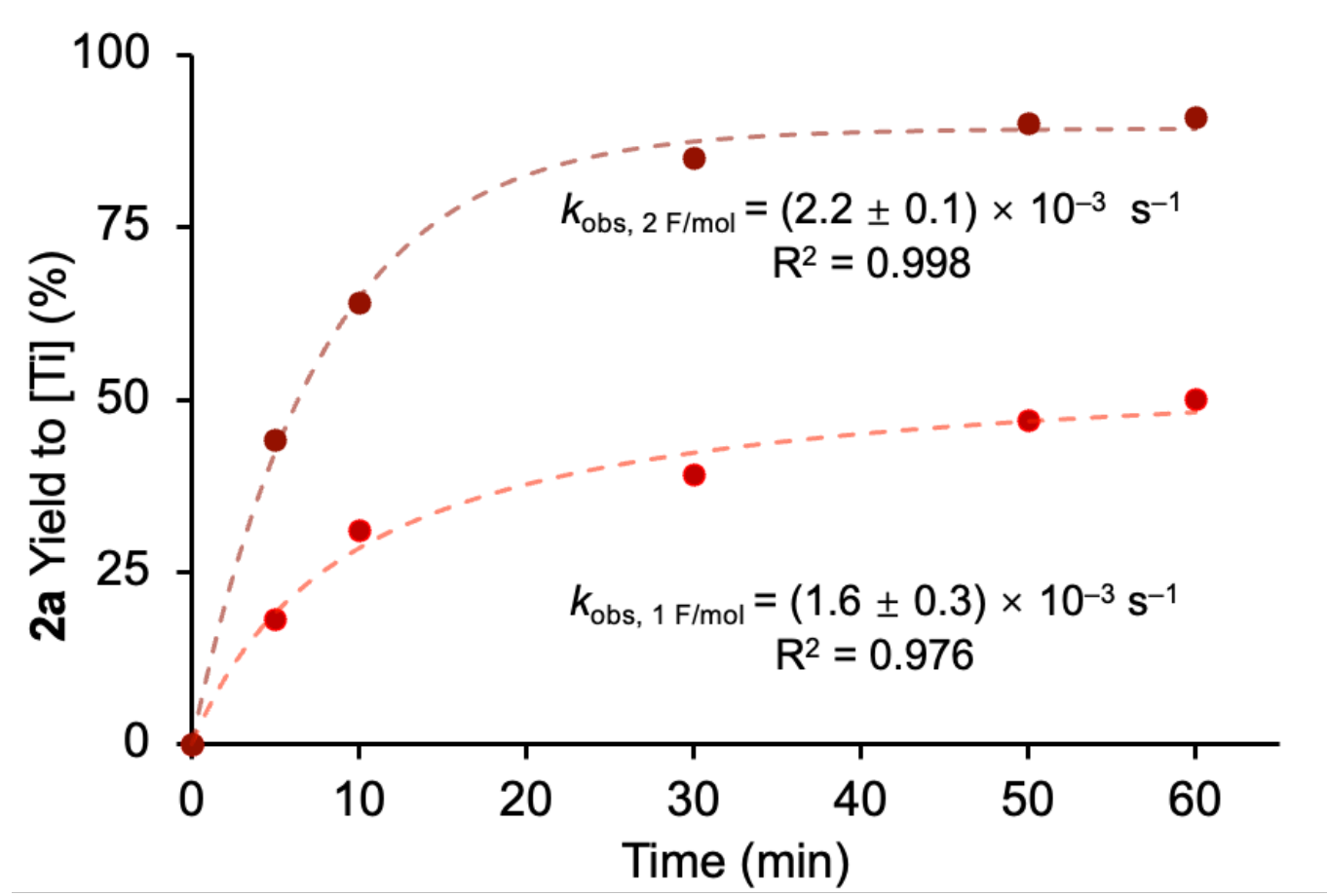

Figure S11. Reaction time course (dots) and first-order kinetic fitting plots (dashed traces) for Ph_2SO deoxygenation mediated by electrochemically generated (TTP)Ti^{III} (light red trace) and (TTP)Ti^{II} species (dark red trace). Conditions: 10 mM (TTP)Ti^{III} and (TTP)Ti^{II} species, 100 mM Ph_2SO, DMA, 1 h, r.t.

For Ti^{II} and Ti^{III}-mediated deoxygenation, the OAT reaction kinetics were assumed to be first-order in [Ph_2SO] and [Ti]. Since [Ph_2SO] >> [Ti], assuming [Ph_2SO] ≈ 0.1 M (constant) and the reaction can be approximated as pseudo-first-order in [Ti]:

$$rate_{OAT} = k[Ph_2SO][Ti^{II}] \approx k_{obs}[Ti^{II}]$$

Separately, we have also conducted similar time course studies from the cathodic solution of coulometry studies with 2 F/mol reduction of (TTP)Ti^{IV}O and BPh_3 (described in section 5.1). A smaller k_{obs} was observed based on first-order kinetic fitting:

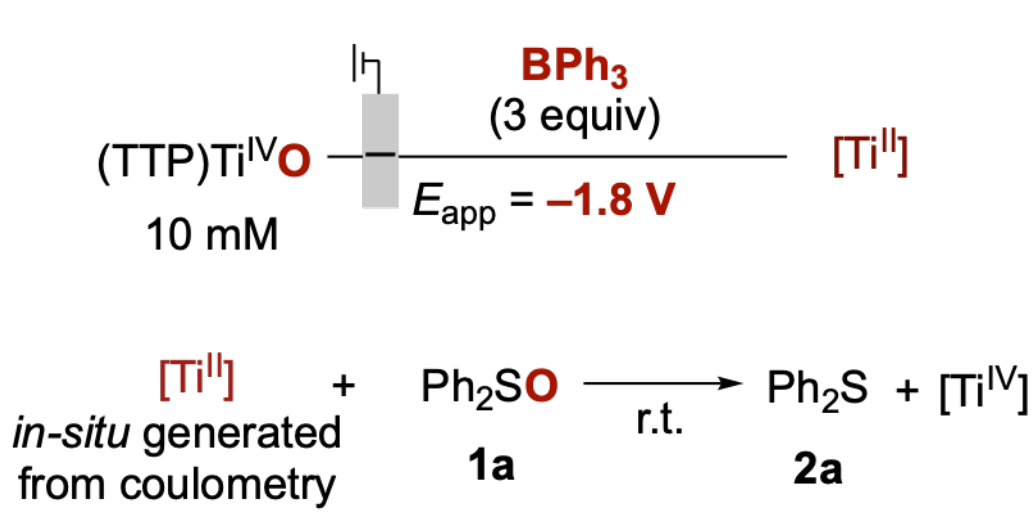

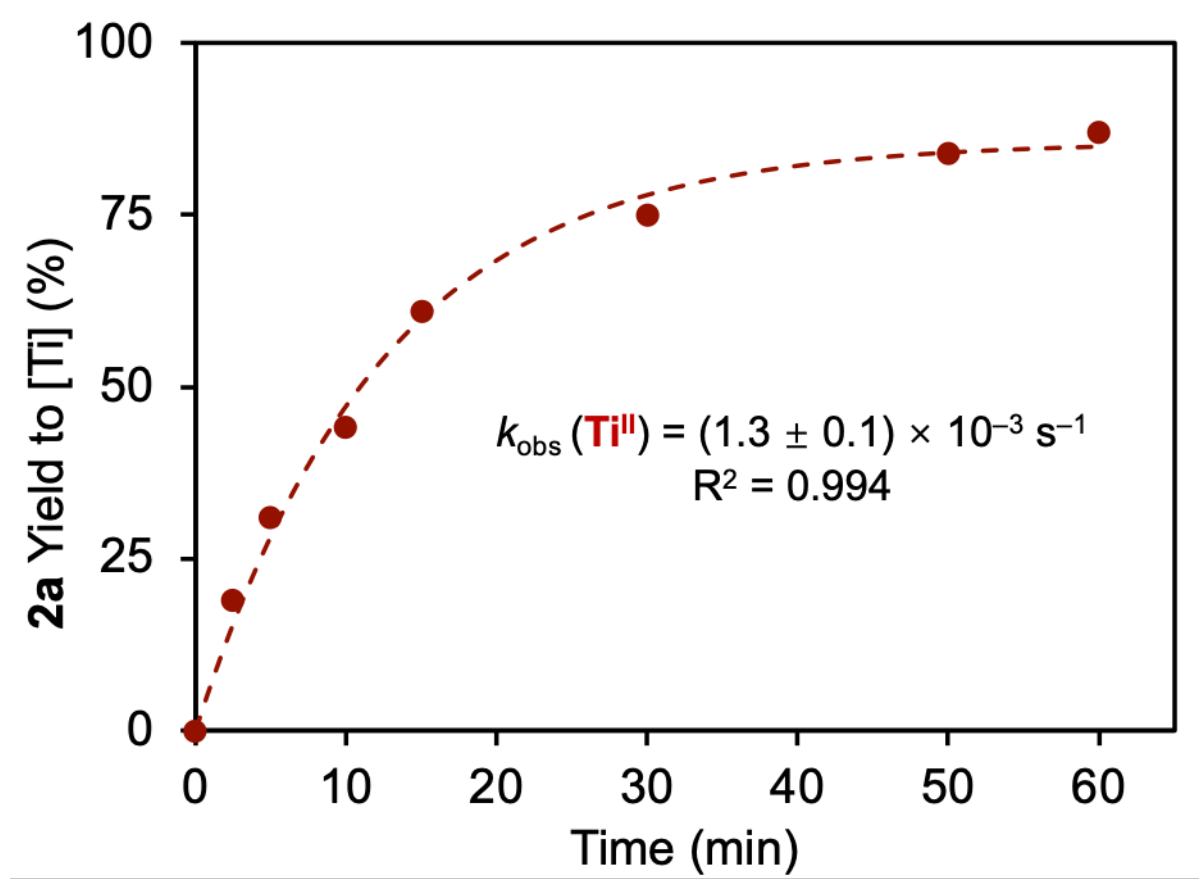

Figure S12. Reaction time course (dots) and first-order kinetic fitting plots (dashed traces) for Ph_2SO deoxygenation mediated by electrochemically generated (TTP)Ti^{II} species with BPh_3. Conditions: 10 mM (TTP)Ti^{II} species, 100 mM Ph_2SO, DMA, 1 h, r.t.

5.3. EPR spectra of (TTP)Ti^{III} species

All operations were carried out in a nitrogen-filled glovebox prior to the analysis. Solutions of **[Ti^{III}]**$^+$ (coulometric studies: reduction of (TTP)Ti^{IV}O in the presence of 3 equiv $B(OPh)_3$) and [(TTP$^•$)Ti^{IV}O]$^-$ (coulometric studies: reduction of (TTP)Ti^{IV}O in the absence of $B(OPh)_3$) were prepared as described in Section 5.1. After the electrolysis was finished, 30 µL of each solution was taken from the cathodic chamber and diluted to 150 µL with DMA to give 2 mM solutions of [Ti]. These two solutions were then immediately transferred to two oven-dried quartz EPR tubes (4 mm diameter, 250 mm long) and capped with a rubber septum in the N_2-filled glovebox. The septum was covered in parafilm to delay air exposure. After removal from the glovebox, the samples were promptly frozen in liquid nitrogen. Additionally, a 2 mM sample of (TTP)Ti^{III}Cl in DMA (synthesized as described in Section 2.5) was analyzed by EPR spectroscopy. X-band EPR spectroscopy was carried out at 115 K using a Bruker ELEXSYS E500 EPR spectrometer with a 5.24 s sweep time, 0.0 modulation phase, 100 kHz modulation frequency, 0.6325 µW microwave power, at 9.303 GHz.

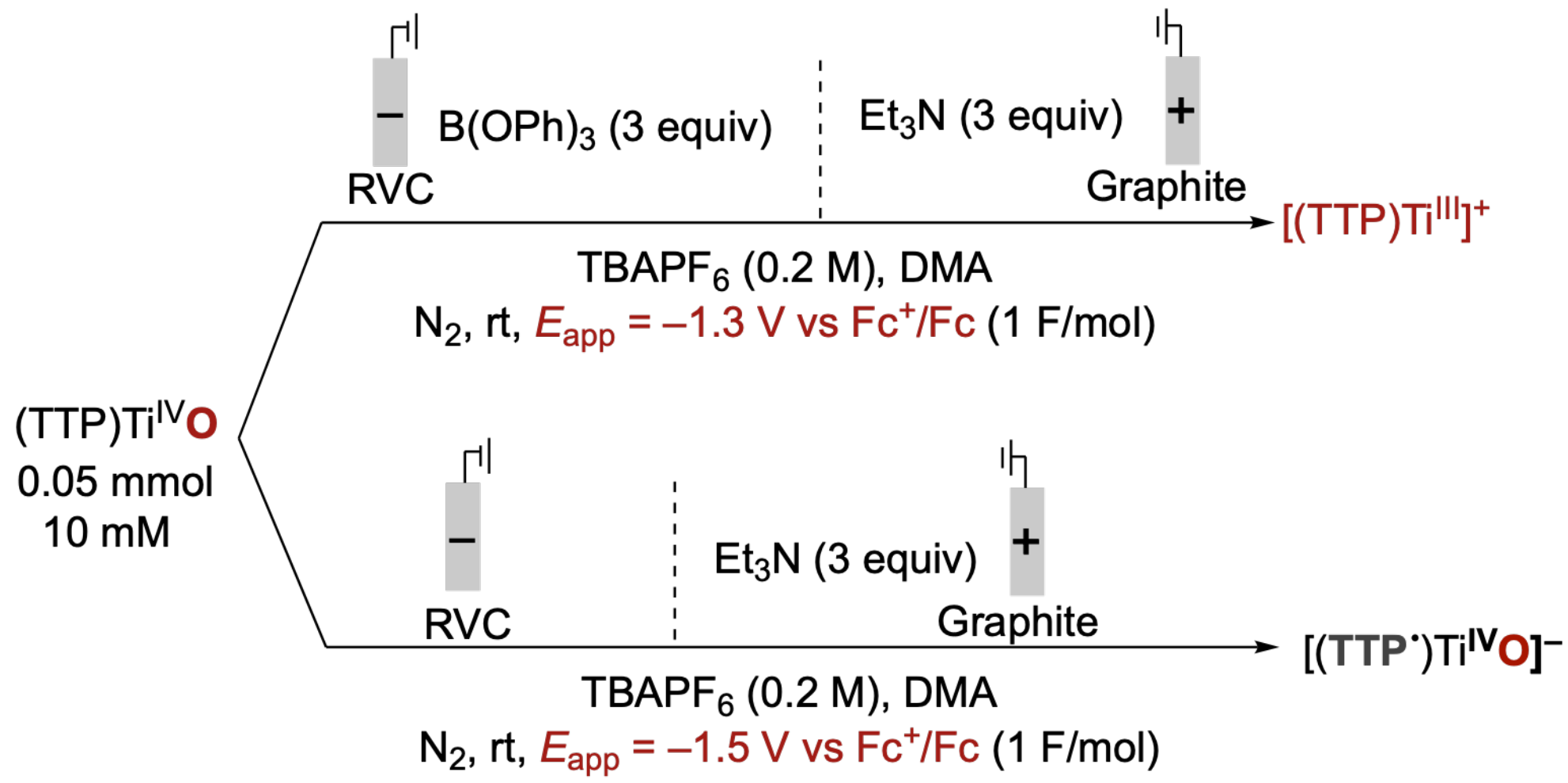

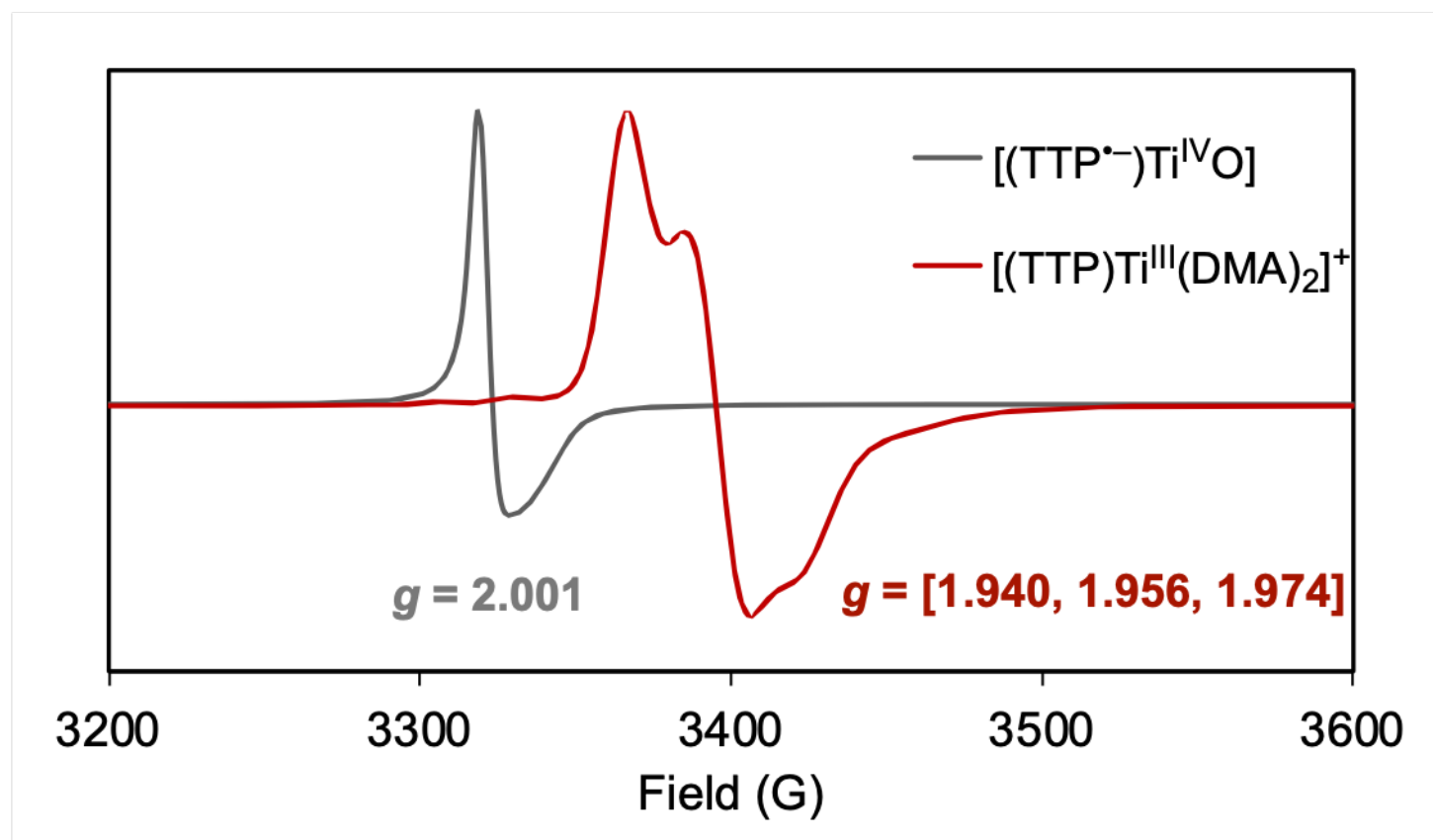

Figure S13. EPR spectra of 1 e⁻ reduced (TTP)Ti^{IV}O in the presence (dark red trace) and absence (gray trace) of 3 equiv $B(OPh)_3$.

Note: A signal observed at $g = 2.001$ is consistent with an organic radical (e.g., ligand-based reduction),[15] while $g = [1.940, 1.956, 1.974]$ is indicative of a metal-centered spin (e.g., Ti-based reduction). The results are consistent with previous studies by Kadish et al., who performed similar studies with the (TPP)Ti complex.[14]

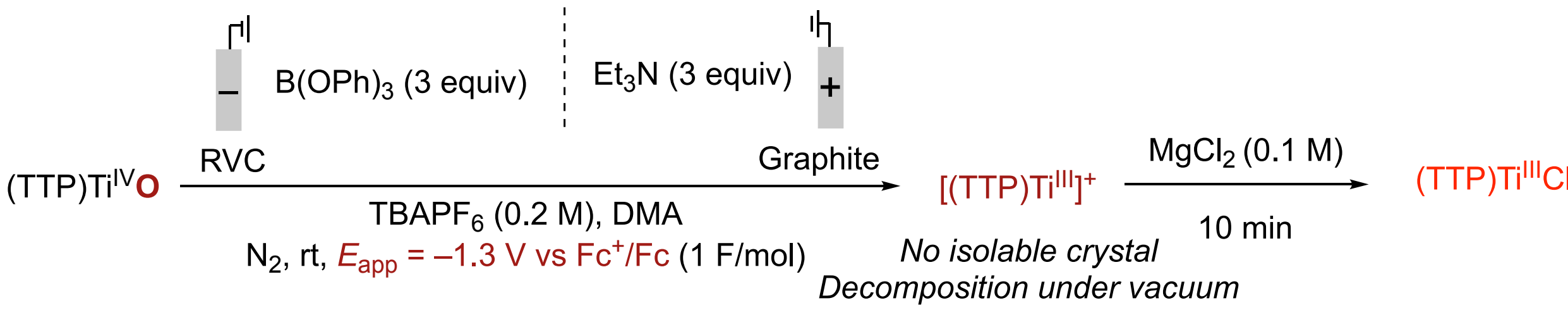

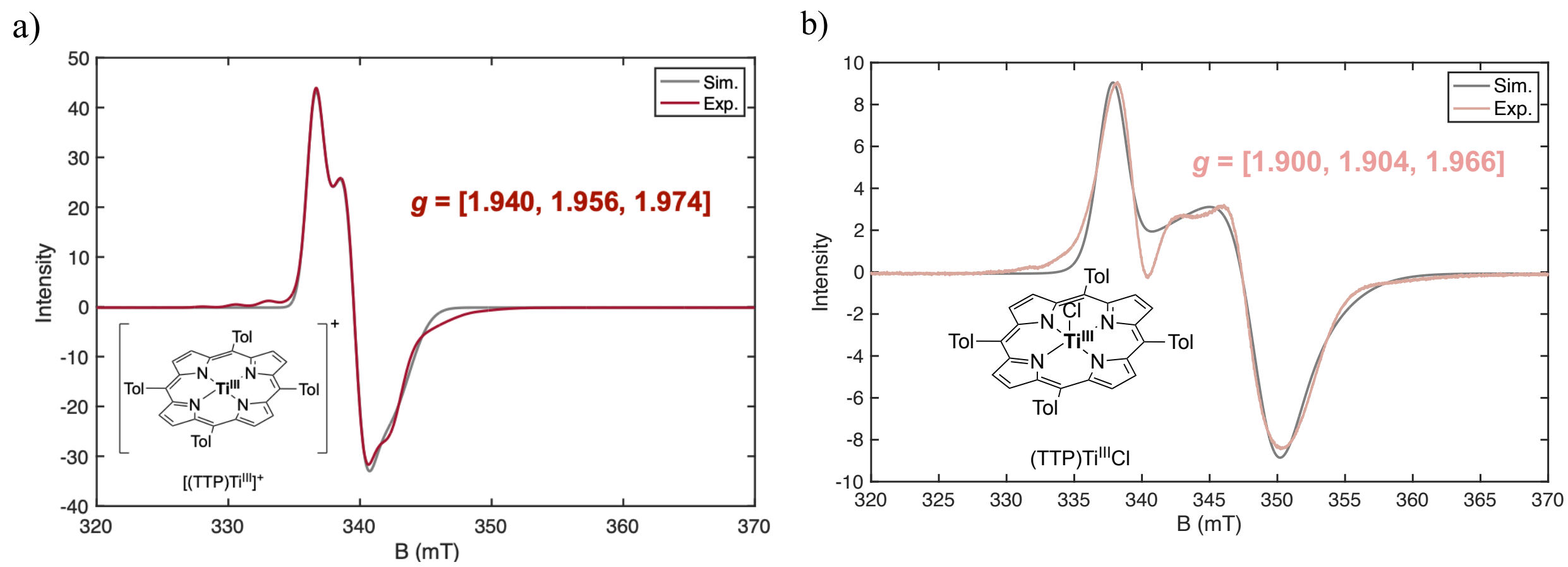

Figure S14. a) Simulation of the EPR spectra of $[(TTP)Ti^{III}(DMA)_2]^+$ by EasySpin. b) Simulation of the EPR spectra of $(TTP)Ti^{III}Cl$ by EasySpin.

Note: Both $[(TTP)Ti^{III}]^+$ and $(TTP)Ti^{III}Cl$ species (g = [1.900, 1.904, 1.966]) gave rise to signals consistent with metal-centered radical and therefore assigned as Ti^{III} species. EPR spectra are similar to precedented signals of Ti^{III}-porphyrin complexes.[16]

6. Quantitative ^1H and ^{11}B NMR spectroscopic studies of $(TTP)Ti^{IV}O$ with $B(OPh)_3$

6.1. Reactions of $(TTP)Ti^{IV}O$ with $B(OPh)_3$ and BPh_3 analyzed by ^1H NMR spectroscopy

Procedure: Seven parallel experiments were carried out in oven-dried 1-dram vials with $(TTP)Ti^{IV}O$ (10 mM, 0.01 mmol) in DMA (1 mL). In a nitrogen-filled glovebox, 1, 3, 6, 9, 12, and 60 equivalents of $B(OPh)_3$ were added separately to the solution, and all six solutions were stirred for 2 h. An aliquot was taken for ^1H NMR spectroscopy every 30 min (50 µL in 500 µL of $CDCl_3$ to quench the reaction) until the concentration of the new Ti species $(TTP)Ti^{IV}(OPh)_2$ and conversion of $(TTP)Ti^{IV}O$ reach equilibrium. 1,3,5-Trimethoxybenzene (0.0033 mmol, 0.33 equiv to [Ti]) was used as an internal standard (dissolved in DMA with the reactants).

(DFT results: K_{eq} = 9.4 x 10^{-2} M^{-1})

$$(TTP)Ti^{IV}O + 2\ B(OPh)_3 \underset{}{\overset{K_{eq} \sim 5.8 \times 10^{-3}\ M^{-1}}{\rightleftharpoons}} (TTP)Ti^{IV}(OPh)_2 + \left\{[(PhO)_2B]_2O\right\}$$

$$K_{eq} = \frac{[(TTP)Ti^{IV}(OPh)_2]\ [(PhO)_2B]_2O]}{[(TTP)Ti^{IV}O]\ [B(OPh)_3]^2}$$

(Assuming $[(PhO)_2B]_2O] = [(TTP)Ti^{IV}(OPh)_2]$)

Table S6. Percent distribution of Ti^{IV} species in redox-neutral deoxygenation with different equivalents of $B(OPh)_3$.

Equiv of $B(OPh)_3$	Conc. of $(TTP)Ti^{IV}O$ (mM)	Conc. of $[(TTP)Ti^{IV}(OPh)_2]$ (mM)	K (10^{-3} M^{-1})
1	9.1	0.7	5.8
3	8.4	1.5	6.3
6	4.5	3.1	5.9
9	3.7	4.0	5.4
12	1.5	5.0	5.8
60	<0.5	8.1	-

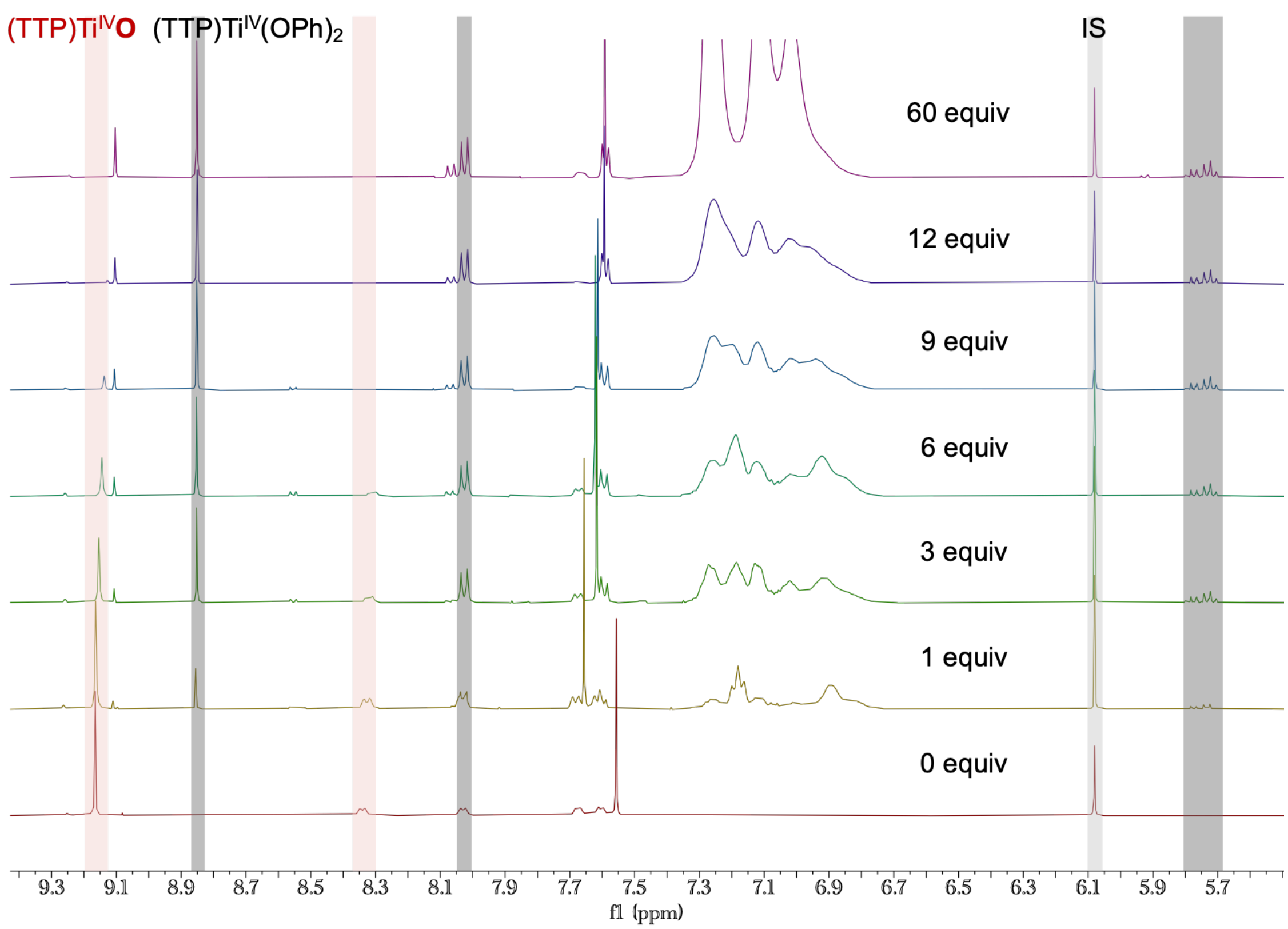

Figure S15. Stacked ^1H NMR spectra of reaction mixtures of $(TTP)Ti^{IV}O$ (10 mM) with $B(OPh)_3$ (1-60 equiv).

Notes: These results show that excess $B(OPh)_3$ can promote redox-neutral conversion to $(TTP)Ti^{IV}(OPh)_2$ with full consumption of $(TTP)Ti^{IV}O$ (≥12 equiv $B(OPh)_3$). Only 16% conversion of $(TTP)Ti^{IV}O$ was observed with 3 equiv of $B(OPh)_3$, which is the stoichiometry used for the CV and coulometry studies. These results suggest that the redox-neutral deoxygenation of $(TTP)Ti^{IV}O$ by $B(OPh)_3$ does not contribute significantly to deoxygenation of $(TTP)Ti^{IV}O$ under electroreductive conditions.

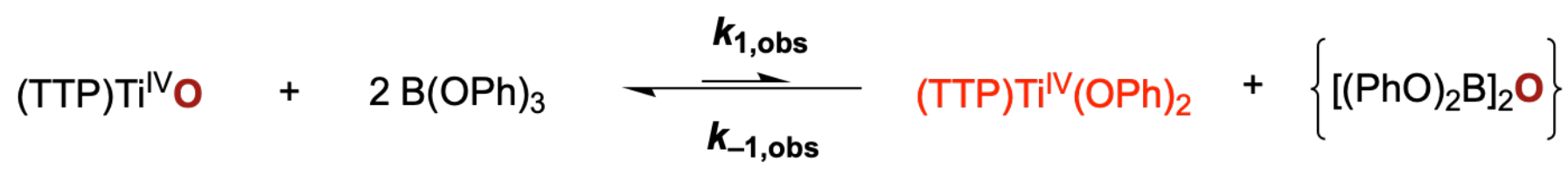

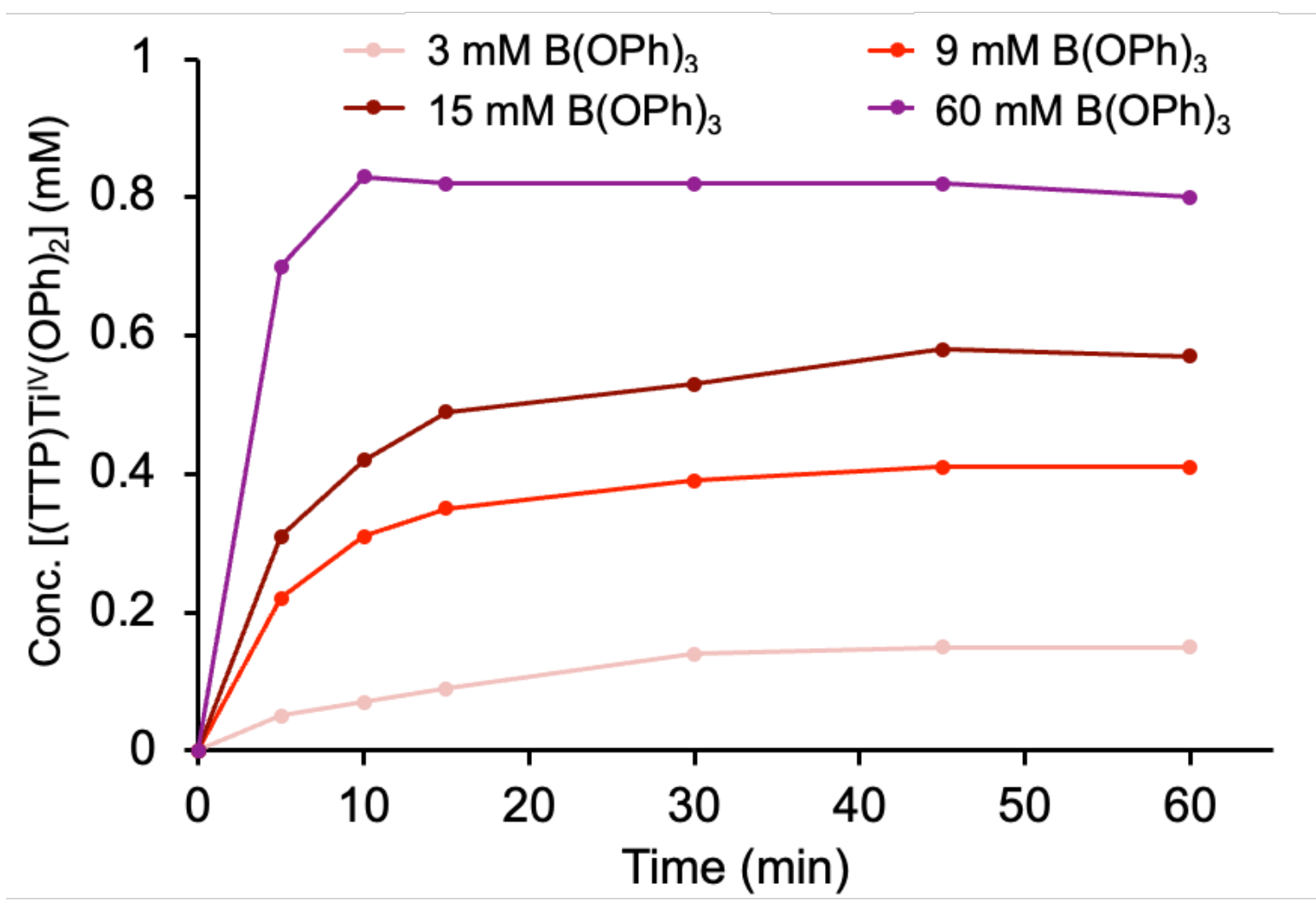

Figure S16. Time course for formation of $[(TTP)Ti^{IV}(OPh)_2]$ in the presence of various equivalents of $B(OPh)_3$ (3-60 equiv).

Notes: Fast equilibrium was observed (~15 min) for direct deoxygenation of $(TTP)Ti^{IV}O$ with $B(OPh)_3$, especially at catalytic conditions (60 equivalents, 70% of $(TTP)Ti^{IV}(OPh)_2$ generated in 5 mins). The forward rate constant, $k_{1,obs}$, is estimated as 3.9 $M^{-2}\,s^{-1}$ based on the time course data at 60 equivalents of $B(OPh)_3$.

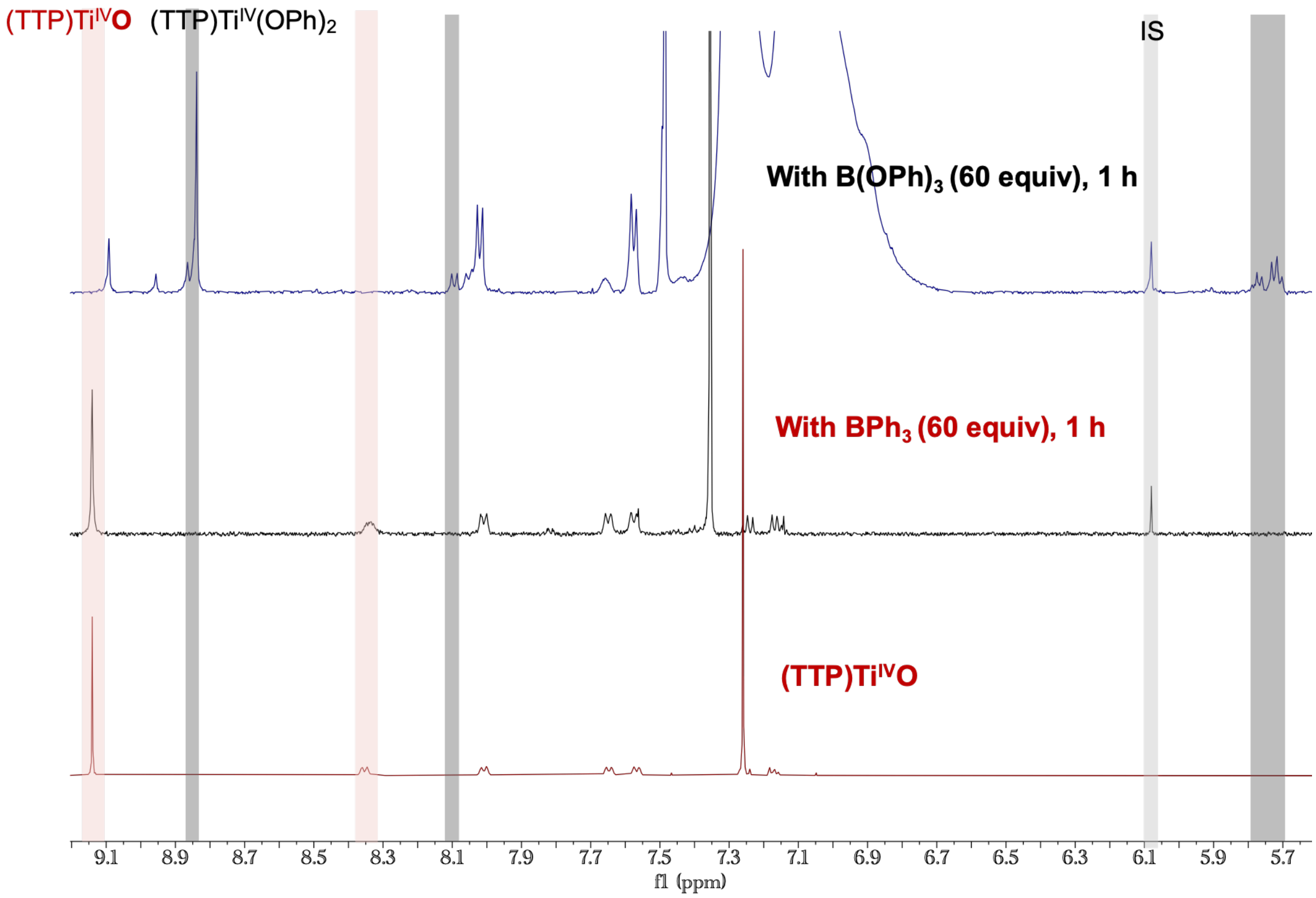

Figure S17. Comparison between ^{1}H NMR spectra of reaction mixtures of (TTP)Ti^{IV}O (1 mM) with 60 equiv of $B(OPh)_3$ (top), with 60 equiv of BPh_3 (middle) after 1 h, and (TTP)Ti^{IV}O only (bottom).

Notes:

1) In the presence of 60 equivalents of $B(OPh)_3$ (same as the $B(OPh)_3$: Ti ratio in catalytic conditions), 83% (TTP)Ti^{IV}(OPh)$_2$ was observed as the dominant species after reaching full equilibrium (1 hour).

2) In contrast, with 60 equivalents of BPh_3, (TTP)Ti^{IV}=O remains intact (92% recovery), indicating nearly no direct deoxygenation occurring with BPh_3 as the Lewis acid prior to reduction.

6.2. Analysis of reaction stoichiometry for reductive deoxygenation of Ph_2SO with $B(OPh)_3$ analyzed by ^{11}B NMR spectroscopy

The deoxygenation of substrates reported here uses $B(OPh)_3$ as the terminal O atom scavenger. Listed below are several possible terminal boron–oxygen adducts that could be formed in the reaction,[17,18] each reflects a different stoichiometry between $B(OPh)_3$ and Ph_2SO.

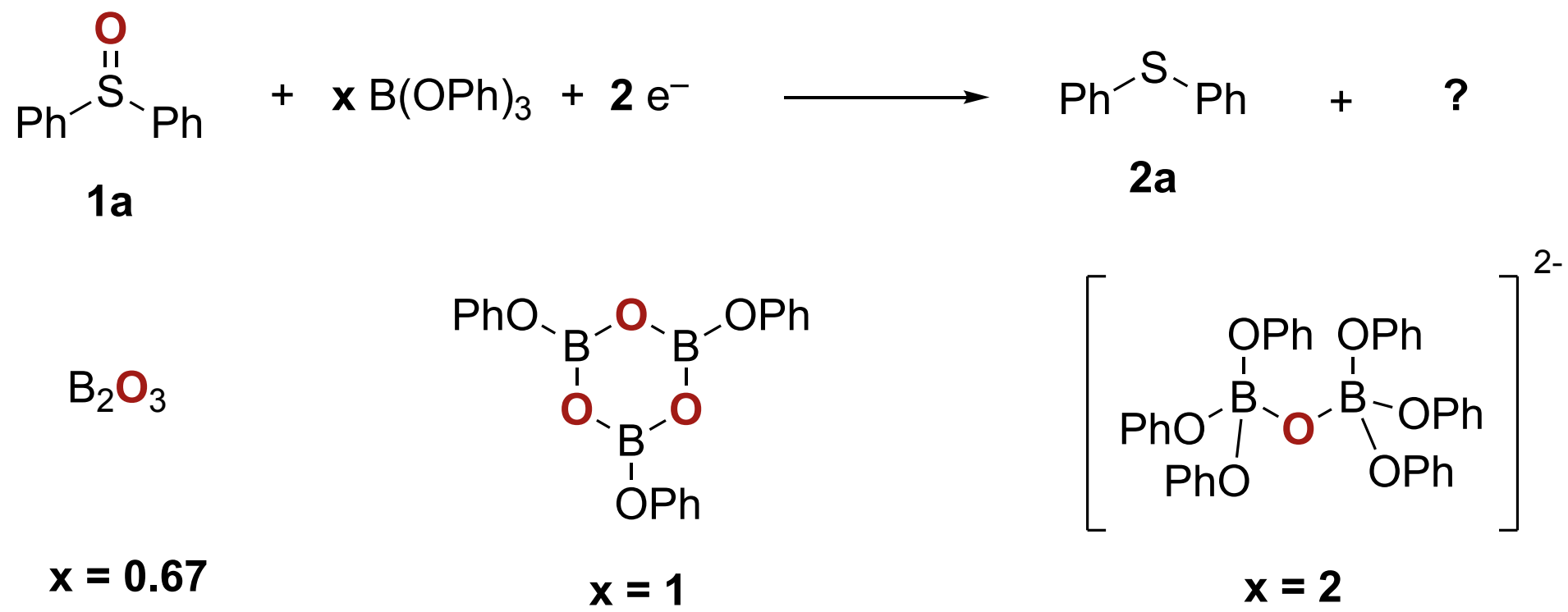

Figure S18. Potential terminal boron–oxygen adducts that form with different stoichiometry relative to Ph_2SO (**1a**).

To establish the stoichiometry of $B(OPh)_3$ consumption relative to charge passed during the deoxygenation of **1a** catalyzed by $(TTP)Ti^{IV}O$, ^{11}B NMR spectra were collected from 0 to 2 F/mol. The reaction was carried out using an H-type divided cell separated by a glass frit. In the cathodic chamber, diphenyl sulfoxide (101 mg, 0.5 mmol), $(TTP)Ti^{IV}O$ (18.3 mg, 5 mol%, 0.025 mmol), $B(OPh)_3$ (435 mg, 3 equiv, 1.5 mmol), and $TBAPF_6$ (387 mg, 0.2 M, 1 mmol) were dissolved in DMA (5 mL) and the chamber was equipped with an RVC (30 PPI, 4.0 cm × 1.0 cm × 0.6 cm, ~3.0 cm was immersed in the solution) as the working electrode and $Ag/AgNO_3$ as the reference electrode. In the anodic chamber, $TBAPF_6$ (387 mg, 0.2 M, 1 mmol) and triethylamine (209 µL, 3 equiv, 1.5 mmol) were dissolved in DMA (5 mL), and the chamber was equipped with a graphite rod (diameter: 0.6 cm; immersed height: ~1.5 cm) as an anode. Electrolysis at a constant potential of −1.6 V was carried out at 70 °C with magnetic stirring. The reaction was terminated after 2 F/mol. The reaction mixture was analyzed by ^{11}B NMR spectroscopy.

The electrolysis was set using a CHI 600 potentiostat, which allowed the charge passed to be monitored. An aliquot of 50 µL from the cathodic chamber was taken at every 0.2 F/mol charge passed (from 0 to 2 F/mol), and each aliquot was diluted by $CDCl_3$ to 500 µL and transferred into an NMR tube for ^{1}H NMR spectroscopy analysis and ^{11}B NMR quantification for each data points with 1,3,5-trimethoxybenzene (1 equiv, 0.5 mmol to **1a)**, added before electrolysis in the cathodic chamber) as the internal standard to quantify formation of **2a**. The conversion of $B(OPh)_3$ was determined by considering the overall concentration of boron-containing species (300 mM, 3 equiv with respect to **1a**) and determining the relative molar ratio between the area of boric ester peak (~17 ppm) and the tetra-coordinated borate peak (boric ester–oxygen adduct, ~2 ppm, the only new peak to emerge during electrolysis).

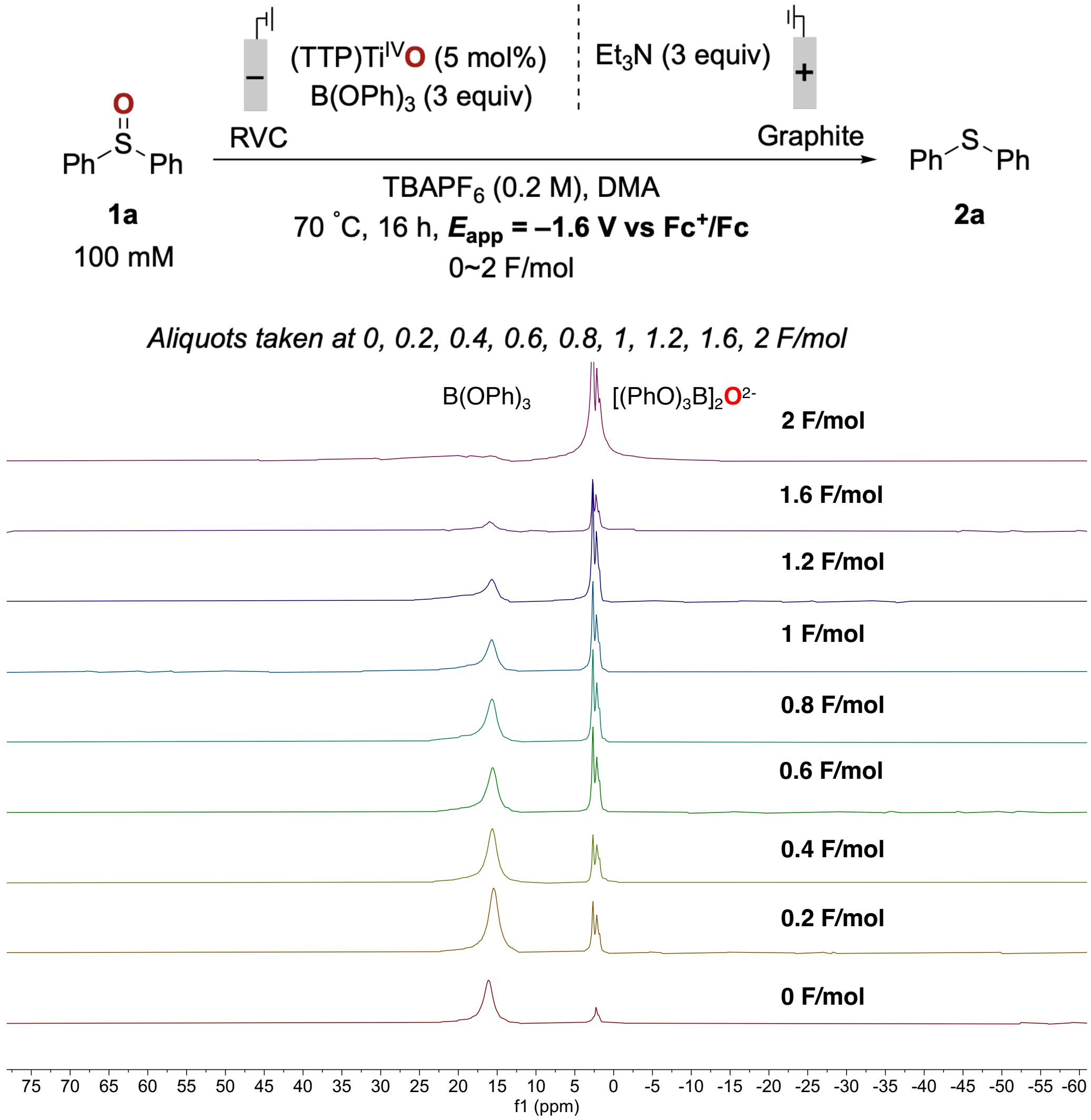

Figure S19. Stacked ^{11}B NMR spectra in $CDCl_3$ of aliquots from electrochemical Ph_2SO (**1a**) deoxygenation with 5 mol% of (TTP)$Ti^{IV}O$ and 3 equivalents of $B(OPh)_3$ at varying charge passed.

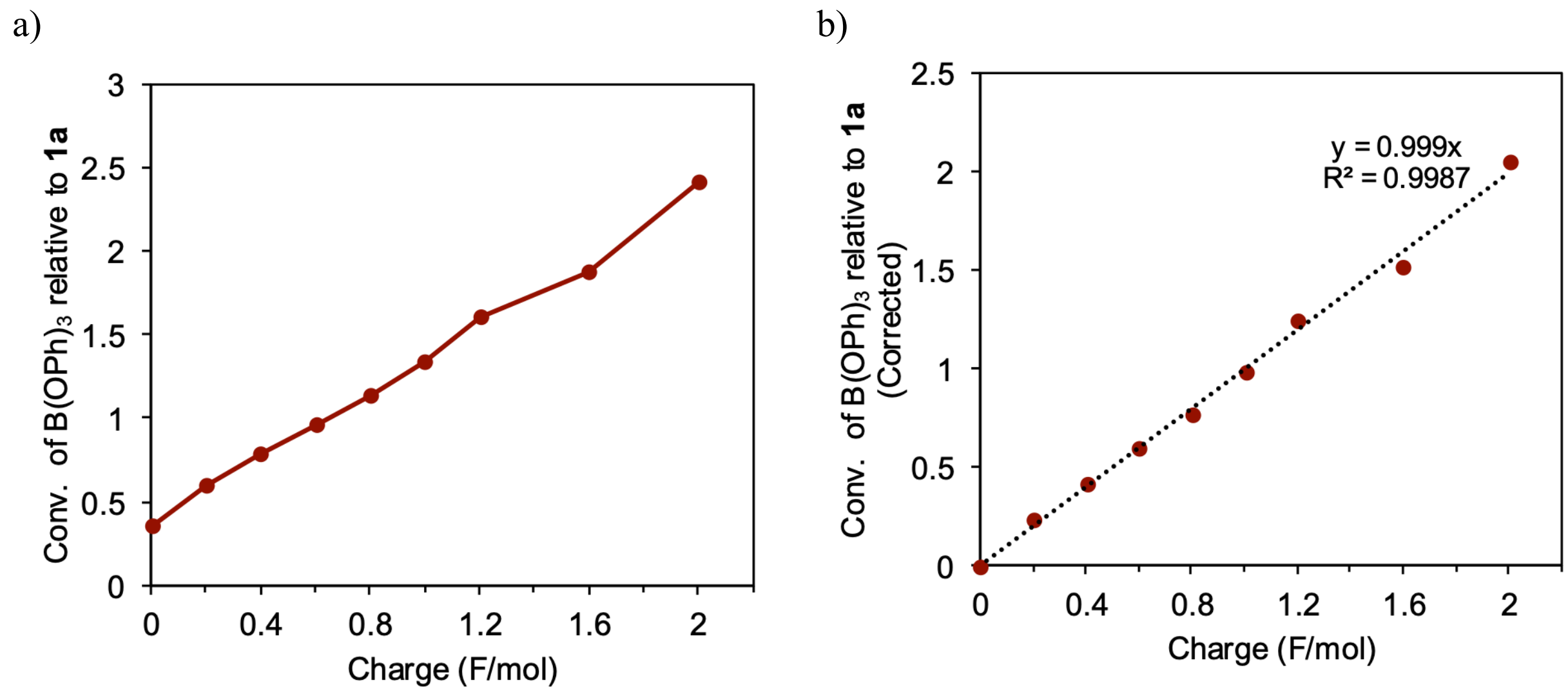

Figure S20. Conversion of $B(OPh)_3$ determined by ^{11}B NMR spectroscopy for catalytic Ph_2SO deoxygenation. a) Conversion of $B(OPh)_3$ vs charge passed. b) Corrected conversion of $B(OPh)_3$ vs charge passed by subtracting background conversion of $B(OPh)_3$ at (0 F/mol) from time point (red dots, from 0 to 2 F/mol) and linear fit (dashed line). The near unity slope of the linear fit indicates a 1:1 stoichiometry of $B(OPh)_3$ and electrons, supporting a two-Lewis acid, two-electron deoxygenation process.

Notes: The conversion of $B(OPh)_3$ against F/mol showed good linearity, supporting a two-electron, two-$B(OPh)_3$ deoxygenation pathway. In addition, the stoichiometry of $B(OPh)_3$ to O-atom from Ph_2SO is 2:1 (x = 2). The boric ester-oxygen adduct was identified via ^{11}B NMR. Based on the ratio of the consumption stoichiometry between $B(OPh)_3$ and Ph_2SO, we propose this boric ester-oxygen adduct is most likely $[(PhO)_3B]_2O^{2-}$.[17]

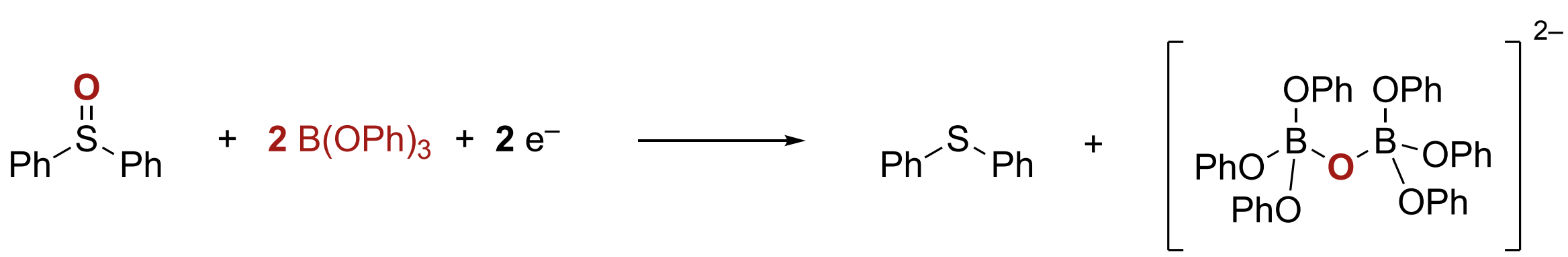

HRMS analysis of the post-electrolysis mixture with $B(OPh)_3$ revealed an ion at m/z = 307.1147, consistent with $[B(OPh)_3(OH)]^-$. An analogous ion assigned as $[BPh_3(OH)]^-$ was observed when BPh_3 was used (m/z = 259.1300). These species support incorporation of oxygen into boron-containing products. The proposed B–O–B-bridged dianions were not directly detected, possibly owing to hydrolysis or protonolysis during sample handling or ESI analysis. Accordingly, the HRMS data are presented as supporting evidence for oxygen transfer to boron rather than definitive assignment of the borate aggregation state:

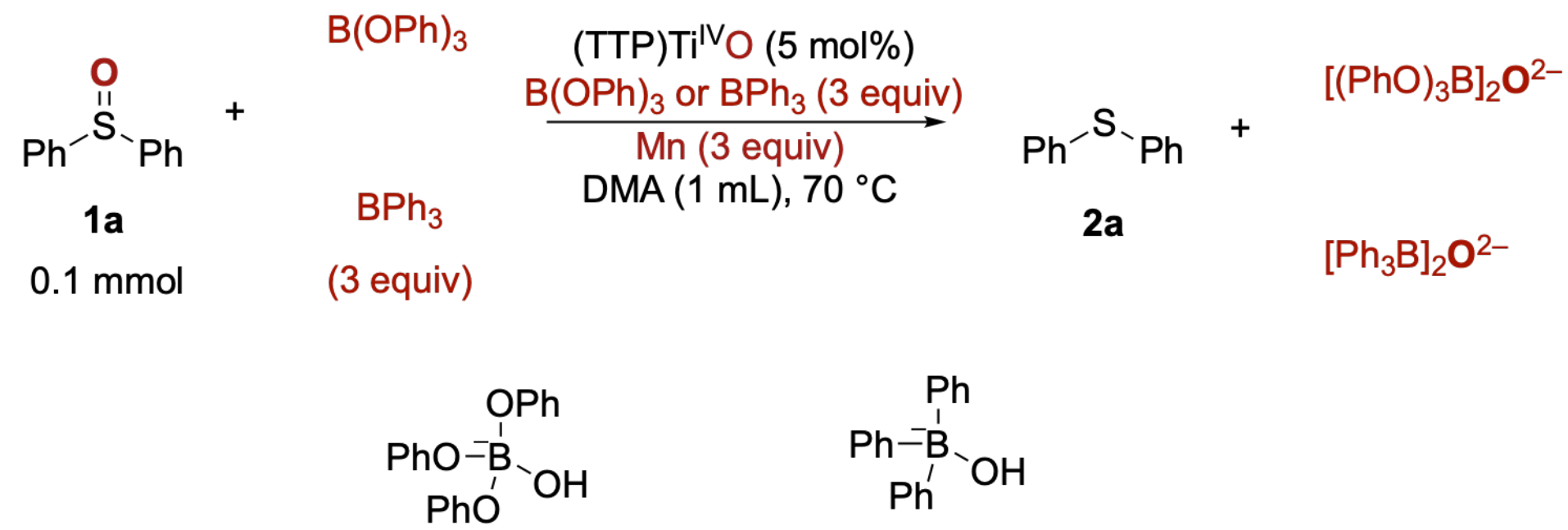

HRMS Calc: 307.1147
Found: 307.1146

HRMS Calc: 259.1300
Found: 259.1297

6.3. Analysis of reaction stoichiometry for reductive deoxygenation of (TTP)Ti^{IV}O with B(OPh)$_3$ analyzed by ^{11}B NMR spectroscopy

To probe the stoichiometry of B(OPh)$_3$ consumption during reduction of (TTP)Ti^{IV}O in the absence of substrate (see **Figure 3C**), we analyzed aliquots of the reaction mixture while performing constant potential electrolysis of (TTP)Ti^{IV}O at –1.3 V (0 → 1 F/mol) followed by –1.6 V (1 → 2 F/mol) in the presence of 4.5 equiv of B(OPh)$_3$.

Procedure: The reaction was carried out using an H-type divided cell (separated by a glass frit). In the cathodic chamber, (TTP)Ti^{IV}O (36.6 mg, 10 mM, 0.005 mmol), B(OPh)$_3$ (65.3 mg, 4.5 equiv, 0.015 mmol), and TBAPF$_6$ (387 mg, 0.2 M, 1 mmol) were dissolved in DMA (5 mL) and equipped with a RVC (30 PPI, 4.0 cm × 1.0 cm × 0.6 cm, ~3.0 cm was immersed in the solution) as the working electrode and a Ag/AgNO$_3$ reference electrode. In the anodic chamber, TBAPF$_6$ (387 mg, 0.2 M, 1 mmol) and triethylamine (21 µL, 3 equiv, 0.15 mmol) were dissolved in DMA (5 mL) and equipped with a graphite rod anode. A constant potential of −1.3 V vs Fc$^+$/Fc was applied at room temperature with magnetic stirring. When the charge passed reached 1 F/mol, the applied potential was switched to −1.6 V vs Fc$^+$/Fc and the electrolysis was continued until an additional 1 F/mol charge (2 F/mol in total) was passed.

The electrolysis was set using a CHI 600 potentiostat where the charge passed can be monitored. An aliquot of 50 µL from the cathodic chamber was taken at 0, 0.4, 0.6, 0.8, 1 and 2 F/mol, which was diluted by CDCl$_3$ to 500 µL and transferred into an NMR tube for ^{11}B NMR spectroscopic analysis for each data points. The conversion of B(OPh)$_3$ was determined by considering the overall concentration of boron-containing species (45 mM, 4.5 equiv with respect to (TTP)Ti^{IV}O) and determining the relative molar ratio between the area of boric ester peak (~17 ppm) and the tetra-coordinated borate peak (boric ester–oxygen adduct, ~2 ppm, the only new peak to emerge during electrolysis).

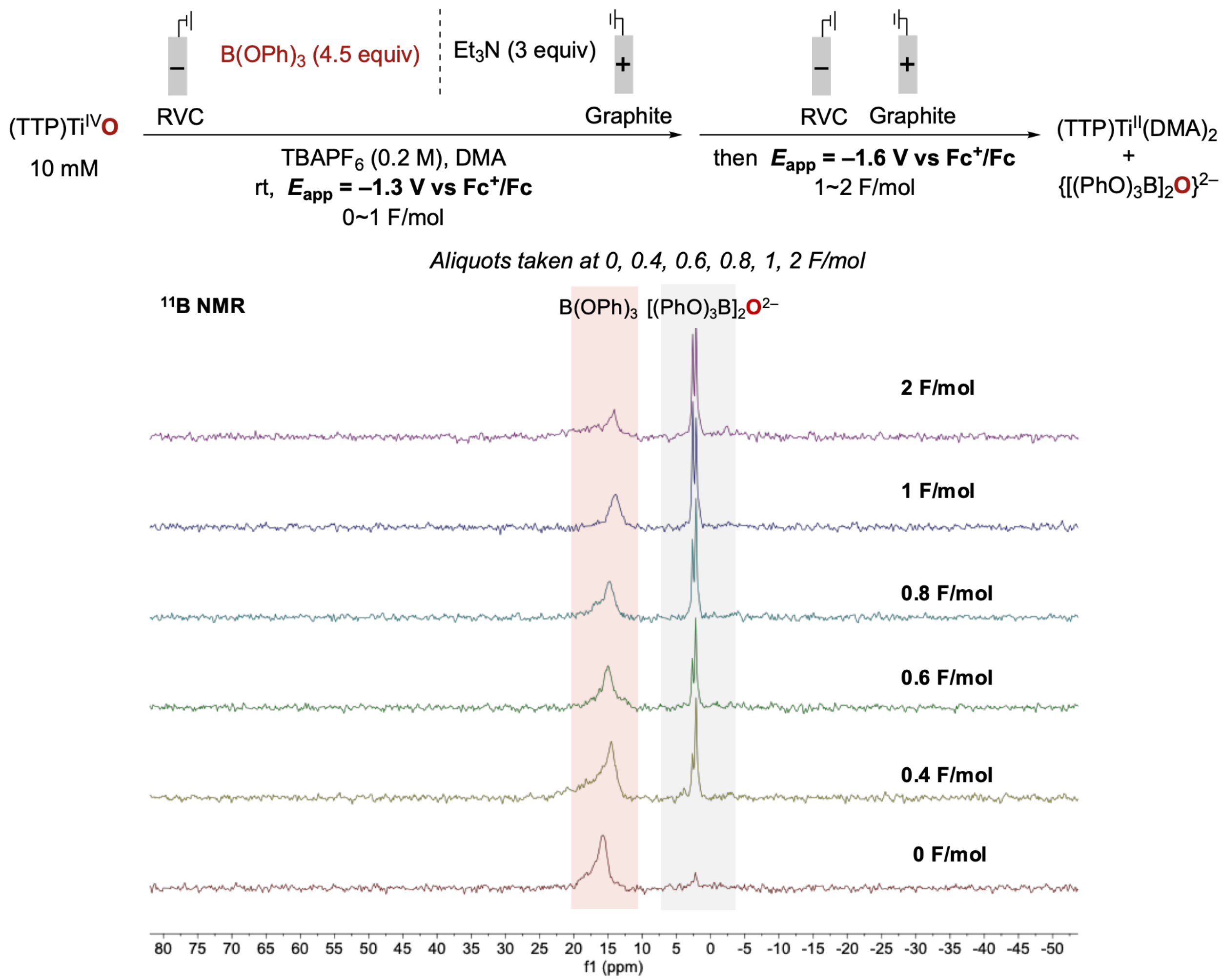

Figure S21. Stacked ^{11}B NMR spectra of aliquots from the electrochemical deoxygenation of (TTP)Ti^{IV}O in the presence of 4.5 equiv B(OPh)$_3$ at varying charge passed.

a)

b)

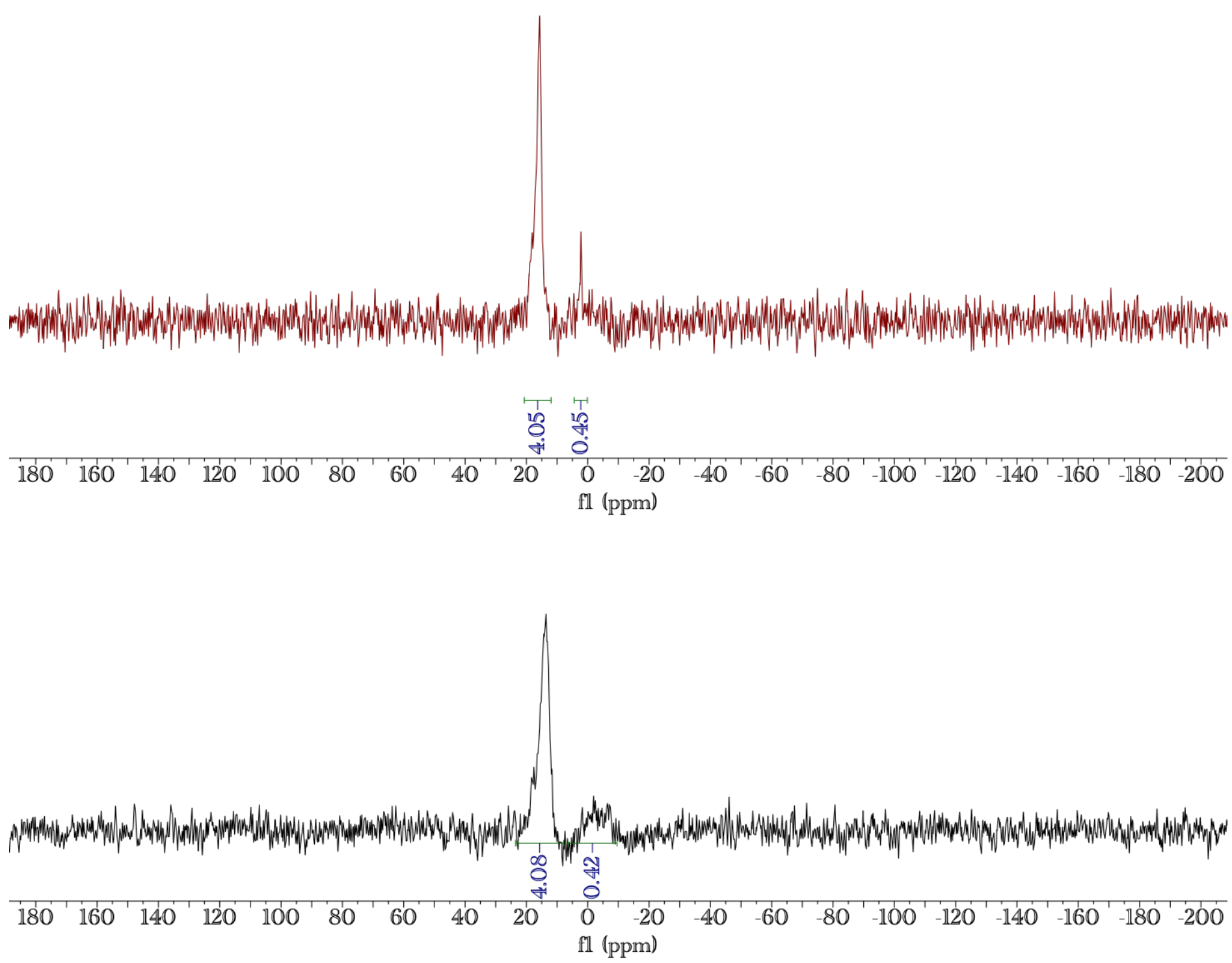

Figure S22. ^{11}B NMR at 0 F/mol before electrolysis from the cathodic solution (0.2 M $TBAPF_6$, 45 mM $B(OPh)_3$ in 5 mL DMA): a) with 10 mM (TTP)$Ti^{IV}O$ (same as the procedure in section 6.3); b) without (TTP)$Ti^{IV}O$: 100 µL of solution was taken and diluted by $CDCl_3$ to 500 µL and transferred into an NMR tube for ^{11}B NMR spectroscopy analysis.

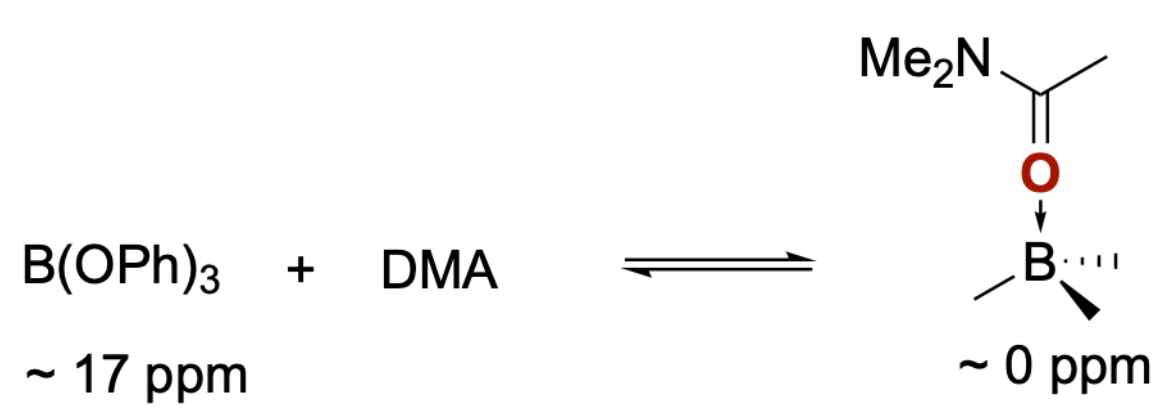

Conclusions: At t = 0, in the cathodic chamber, 4.5 mM of tetra-coordinated borate species (chemical shift ~ 0 ppm, 0.45 equiv to (TTP)$Ti^{IV}O$) was present based on ^{11}B NMR spectrum (**Table S7** and **Figure S17a**). To further confirm whether this borate species comes from coordination with (TTP)$Ti^{IV}O$ or from DMA coordination, we also prepared a solution identical to the cathodic chamber but without (TTP)$Ti^{IV}O$ catalyst (**Figure S17b**), and a borate peak with similar integration (4.2 mM, 0.42 equiv to (TTP)$Ti^{IV}O$) was observed on ^{11}B NMR. These results indicate that solvent coordination to $B(OPh)_3$, instead of Ti-oxo coordination, majorly contributes to the observed initial tetra-coordinated borate signal at ~ 0 ppm before electrolysis and therefore can be considered as background in further ^{11}B NMR analysis.

Table S7. Equivalent of borate formation (Consumption of $B(OPh)_3$) from electrolysis relative to $(TTP)Ti^{IV}O$.

Charge (F/mol)	Total borate (equiv to $(TTP)Ti^{IV}O$)	Formation of borate from electrolysis (equiv to $(TTP)Ti^{IV}O$)
0	0.45	0
0.4	1.18	0.73
0.6	1.63	1.18
0.8	2.01	1.56
1	2.56	2.11
2	2.5	2.05

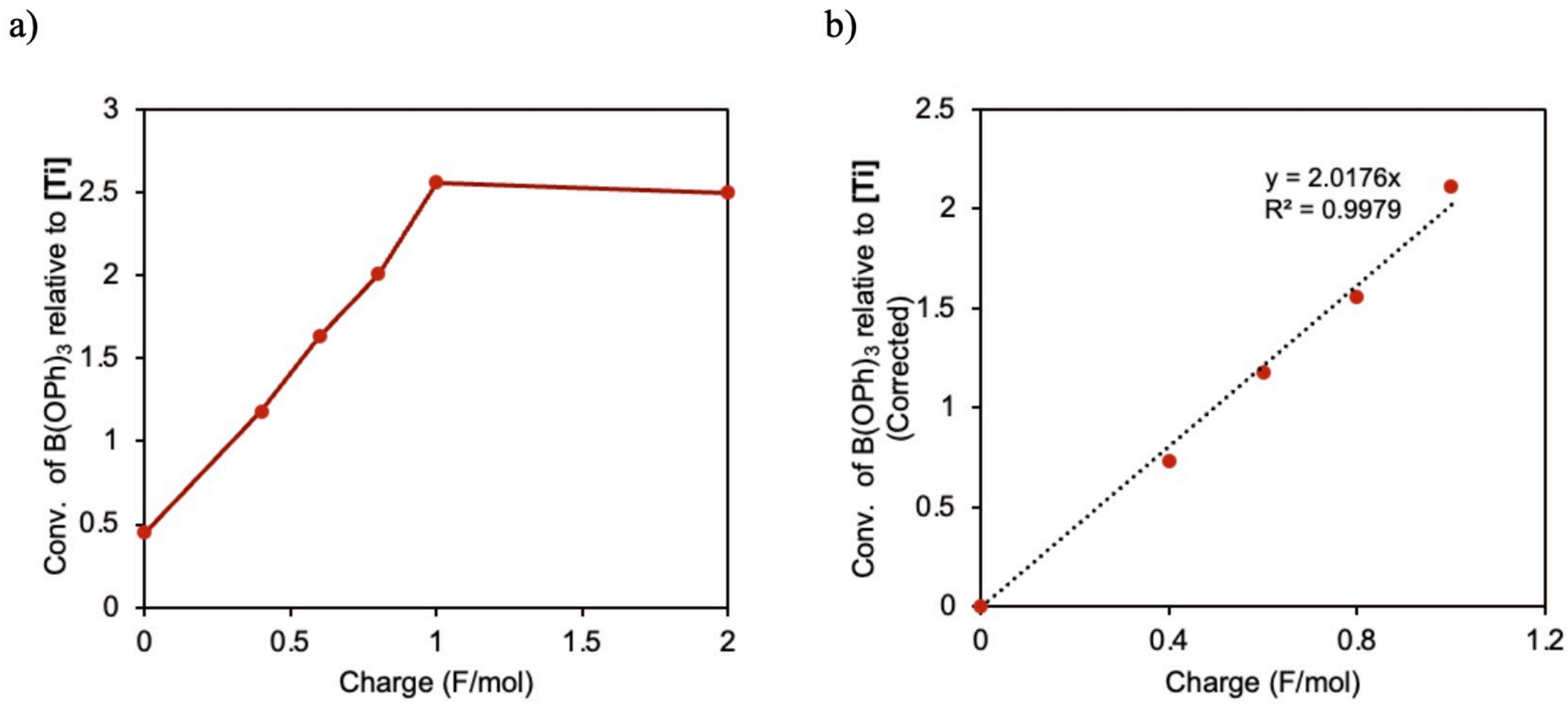

Figure S23. Conversion of $B(OPh)_3$ observed by ^{11}B NMR for stoichiometric deoxygenation of $(TTP)Ti^{IV}O$. a) Conversion of $B(OPh)_3$ vs charge. b) Linearity of conversion vs charge by subtracting background conversion of $B(OPh)_3$ to borate (0 F/mol) from each time point (red dots, from 0 to 2 F/mol). The slope (close to 2) indicates a 2:1 stoichiometry of conversion of $B(OPh)_3$ vs electrons and a two-Lewis acid, one-electron deoxygenation process from Ti^{IV} to Ti^{III}.

Notes: Since the stoichiometry for $B(OPh)_3$ to O-atom from $(TTP)Ti^{IV}O$ is 2:1, the conversion of $B(OPh)_3$ here reflects the stoichiometry of deoxygenated $(TTP)Ti^{IV}O$. At 1 F/mol charge passed, where Ti^{IV} to Ti^{III} happens, ~2 equivalents of $B(OPh)_3$ were converted to boric ester–oxygen adduct (the characteristic signal of tetravalent borate species appeared at 0–5 ppm in ^{11}B NMR). This indicates that 1 equivalent of $(TTP)Ti^{IV}O$ has been fully deoxygenated to a Ti^{III} species. Taking the net charge into consideration, the new Ti-species should be assigned as $\mathbf{[Ti^{III}]^+}$ (cationic Ti^{III}-porphyrin species).

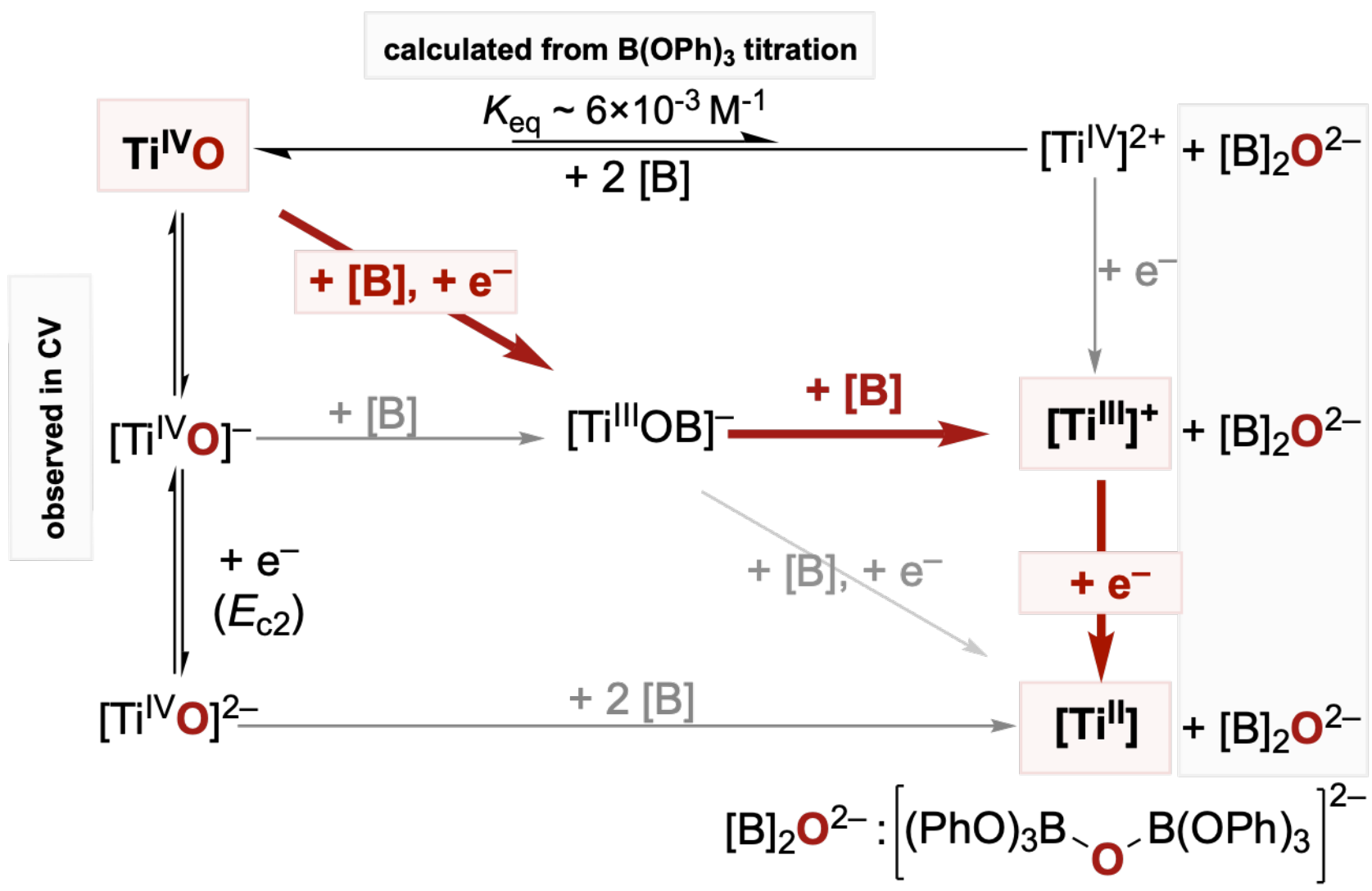

Figure S24. Summarized deoxygenation pathways for (TTP)Ti^{IV}O. a) A one-Lewis acid, one-electron step from Ti^{IV} to Ti^{III} followed by a second one-Lewis acid, one-electron step from Ti^{III} to Ti^{II}. b) A two-Lewis acid, one-electron step from Ti^{IV} to Ti^{III}, followed by one-electron reduction from Ti^{III} to Ti^{II}. Ligands on Ti are omitted for clarity.

Notes: The summarized deoxygenation pathway is shown in the figure above. The first 1-$B(OPh)_3$, 1-electron process converts (TTP)Ti^{IV}O to $\{(TTP)Ti^{III}O[B]\}^-$, followed by rapid deoxygenation involving a second molecule of $B(OPh)_3$ to afford cationic $[(TTP)Ti^{III}]^+$ species. A second 1-electron reduction of $[(TTP)Ti^{III}]^+$ provides access to $[(TTP)Ti^{II}]$ species.

7. Computational studies

7.1. Computational methods

All density functional theory (DFT) calculations were performed using Gaussian 16.[19] The B3LYP[20–23] functional was employed for all calculations. For the free energy calculations, LANL2DZ[24] basis set with the corresponding effective core potential (ECP) was employed for Ti, while def2-TZVP[25] was used for B, O, and S, and def2-SVP[25] for all other elements. For spin density analyses, the def2-SVP basis set was used for all atoms. Löwdin population analyses were also carried out for the α-HOMO, the α-HOMO–1 orbital in triplet species, and the spin density to quantify how much each is localized on the Ti center, the TTP ligand, and other ligands.

Solvent effects were modeled by polarizable continuum model (PCM) using the integral equation formalism (IEF-PCM)[26] variant with parameters corresponding to N,N-dimethylacetamide (DMA), consistent with the experiment conditions. For each Ti complex, several solvated possibilities with different numbers of coordinated DMA solvent molecules were examined.

All optimized structures were confirmed to be local minima on the potential energy surface by positive vibrational frequencies. Zero-point energies and thermal corrections were obtained within the harmonic approximation. For low frequency modes, quasi-harmonic treatment with cut-off value of 100 wavenumbers are applied for enthalpy and entropy corrections.[27,28] Furthermore, to better approximate condensed-phase thermodynamics, all computed free energies were further corrected by applying an empirical scaling factor of 0.5 to the translation and rotational entropy derived from the ideal gas approximation (the Wertz correction).[29–31] In addition, because the reactants were treated at 10 mM concentration, except for DMA, which was treated as 10 M based on its density, standard-state corrections of RTln(c) were added to the computed free energies.

Spin-state energetics were carefully examined for all Ti species. Ti^{II}, Ti^{III}, and Ti^{IV} complexes were treated in their triplet, doublet, and singlet spin states, respectively. The singlet state for Ti^{II} was also evaluated and found to be higher in energy than the triplet state, confirming the triplet state as the most stable spin state. For Ti^{III} complexes, spin density analyses were performed to determine whether the unpaired electron is primarily localized on the Ti center or delocalized over the ligand framework.

7.2. Calculation of BDFE

Gibbs free energies of the dissociation of the Ti^{IV}=O bond were calculated for various coordination states of both Ti^{II} and Ti^{IV} species (**Figure S25**). These states include (TTP)Ti^{II} with 0–2 explicitly coordinated DMA molecules and (TTP)Ti^{IV}O without coordinating DMA. A DMA-coordinated (TTP)Ti^{IV}O structure was not included because no stable minimum could be located for the 1-DMA adduct. The most stable coordination state was found to be with two coordinating DMA for (TTP)Ti^{II}. All free energies are referenced to (TTP)Ti^{IV}O without coordinated DMA. The total free energy of the solvated low-valent (TTP)Ti^{II} species together with a triplet oxygen atom is approximately 124 kcal/mol higher than that of the (TTP)Ti^{IV}O species in DMA. This large free energy difference highlights the substantial thermodynamic stability of the Ti^{IV}=O bond and provides a quantitative basis for the challenges associated with Ti^{IV}=O turnover.

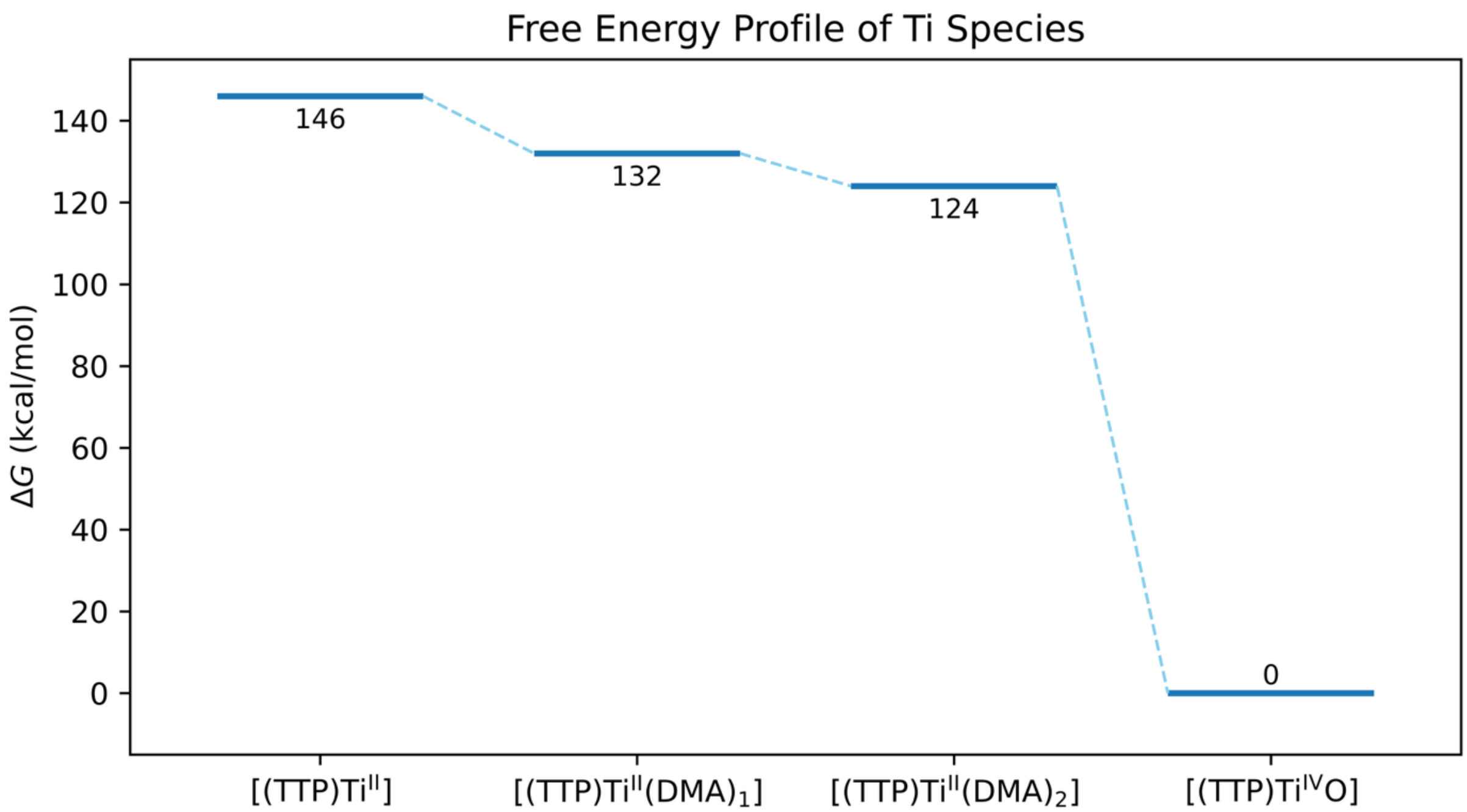

Figure S25. Computed free energy profile of Ti species under DMA solvent conditions. Relative Gibbs free energies are shown for (TTP)Ti^{II} species with 0–2 explicitly coordinated DMA molecules and for (TTP)Ti^{IV}O species without coordinated DMA. All states are stoichiometrically balanced: the free energy differences are deduced from balance reactions with triplet O atom and free DMA solvents included, but the O atom and free DMA molecules are omitted for simplicity. All values are referenced to (TTP)Ti^{IV}O without coordinated DMA.

7.3. Spin density results

The spin density distribution and molecular orbital of interest are shown below.

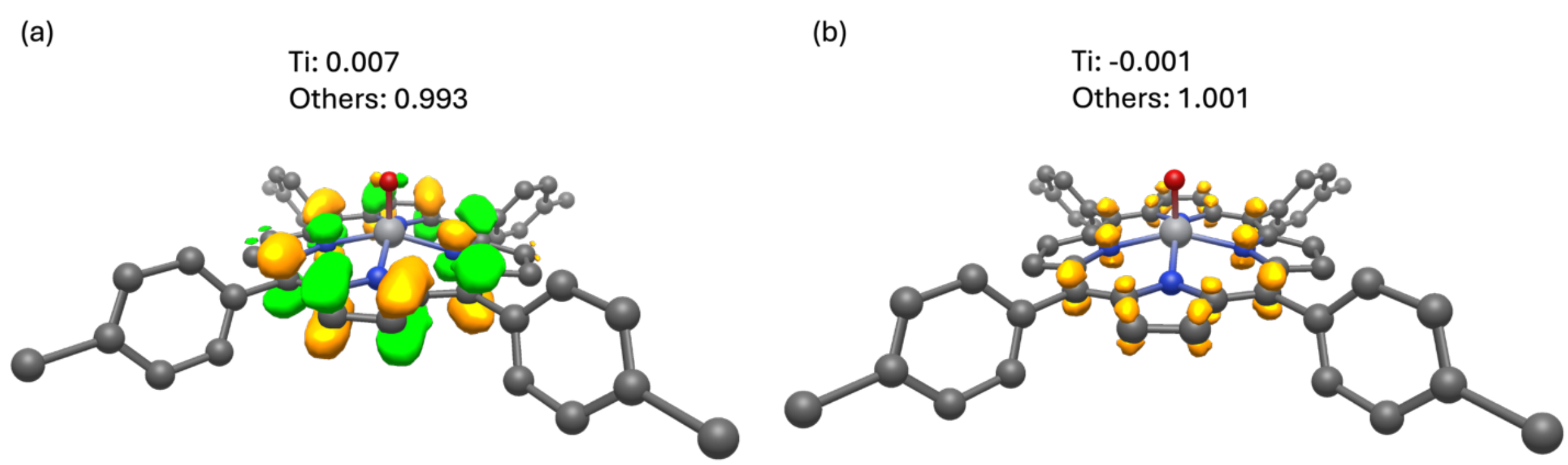

Figure S26. Singly occupied molecular orbital (SOMO) and spin-density distributions of [(TTP•)TiO]⁻ (a) SOMO (α-HOMO, –3.278 eV) and (b) spin-density distribution. For the molecular orbital in panel (a), orange and green denote the positive and negative phases of the orbital, respectively. For the spin-density distribution in panel (b), orange and green denote positive and negative values of $\rho_\alpha - \rho_\beta$, respectively. Unless otherwise noted, molecular orbital and spin-density isosurfaces are shown at isovalues of 0.03 and 0.005, respectively. Hydrogen atoms are omitted for clarity.

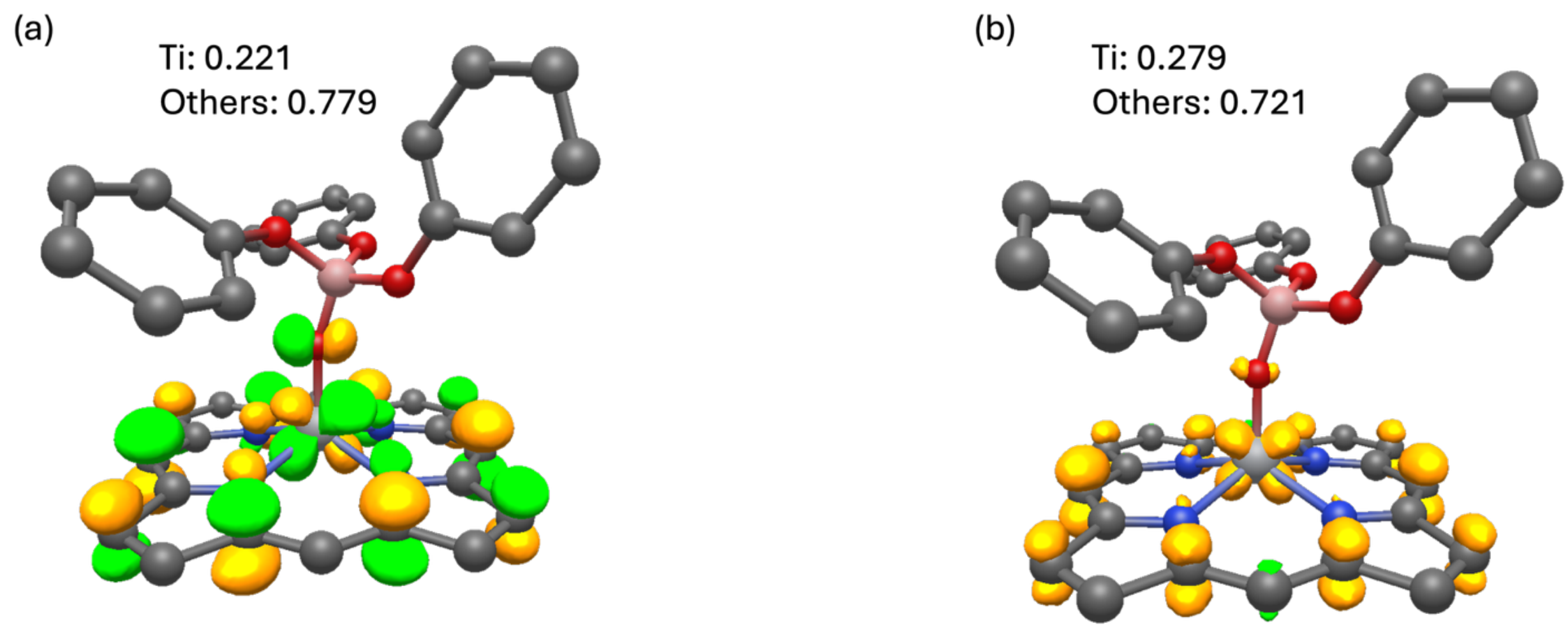

Figure S27. SOMO and spin-density distributions of {(TTP)TiO[B(OPh)$_3$]}$^-$ (a) SOMO (α-HOMO, –3.580 eV) and (b) spin-density distribution. For better visualization, the isosurface value of the α-HOMO and spin density is set to 0.04 and 0.004. The p-tolyl groups are omitted for clarity.

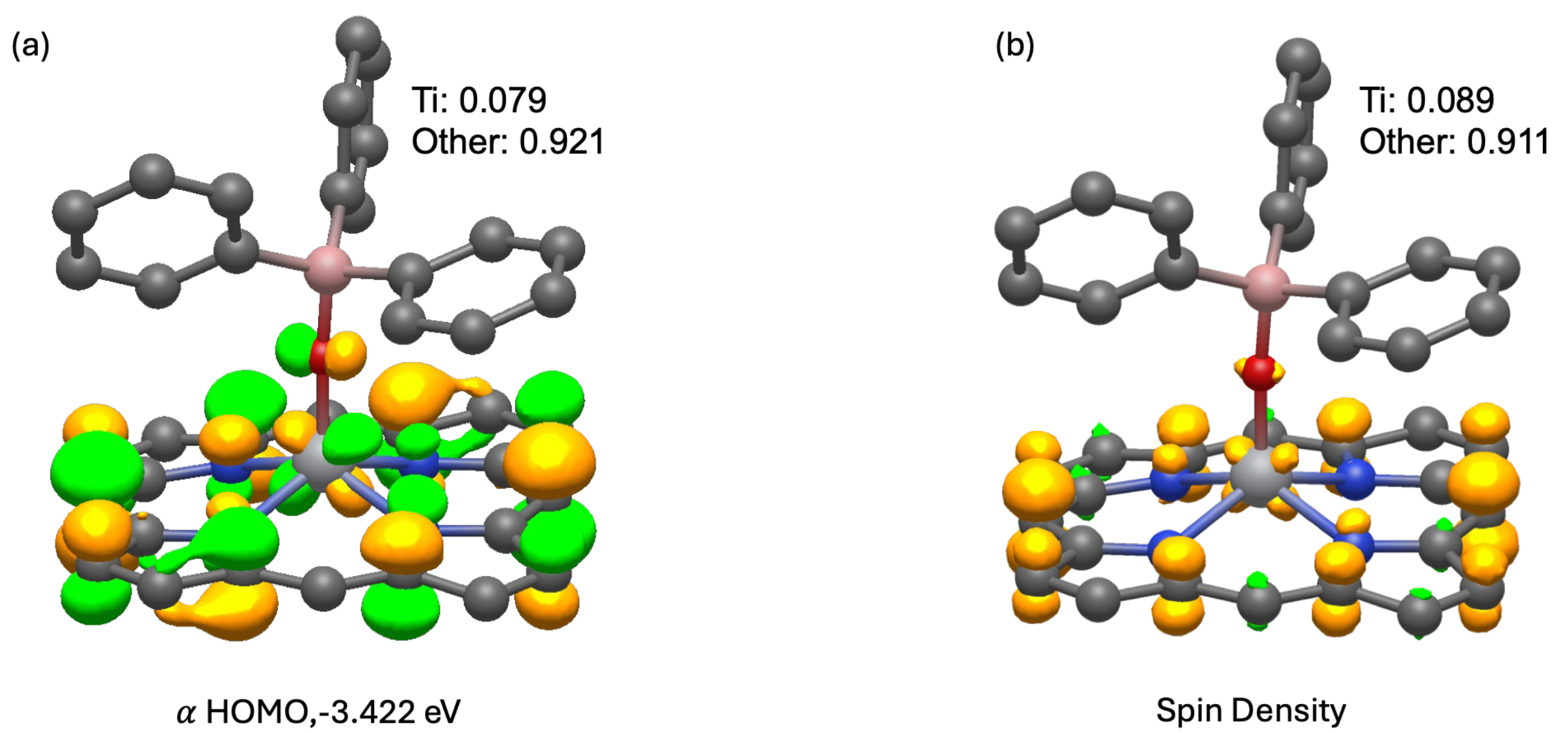

Figure S28. SOMO and spin-density distributions of {(TTP)TiO(BPh$_3$)]}$^-$ (a) SOMO (α-HOMO, –3.422 eV) and (b) spin-density distribution. For better visualization, the isosurface value of the α-HOMO and spin density is set to 0.04 and 0.004. The p-tolyl groups are omitted for clarity.

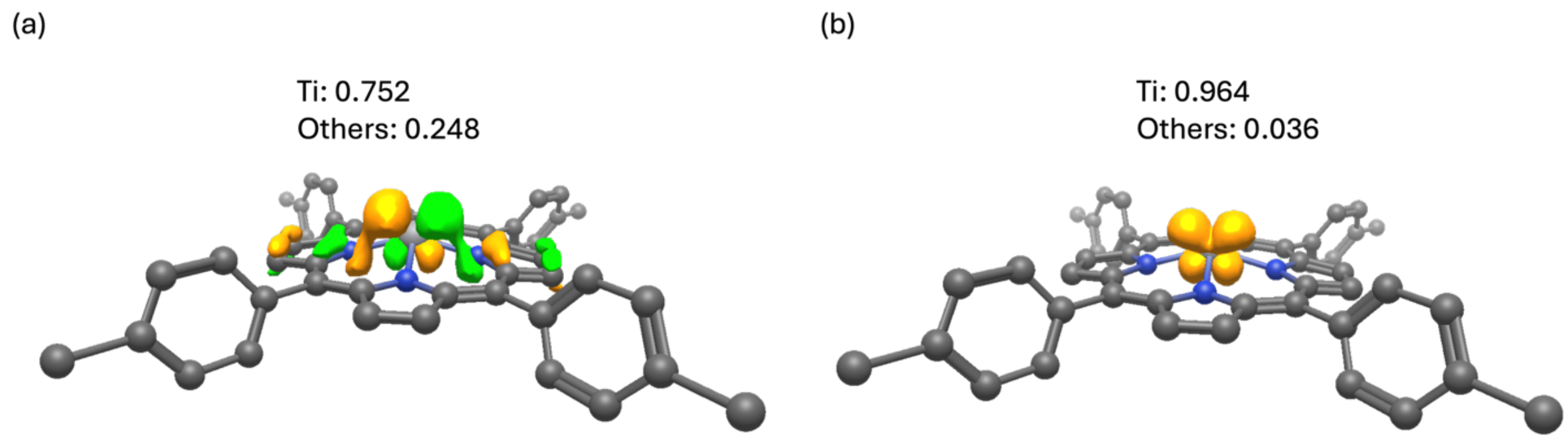

Figure S29. SOMO and spin-density distributions of $[(TTP)Ti]^+$, (a) SOMO (α-HOMO, –5.314 eV) and (b) spin-density distribution.

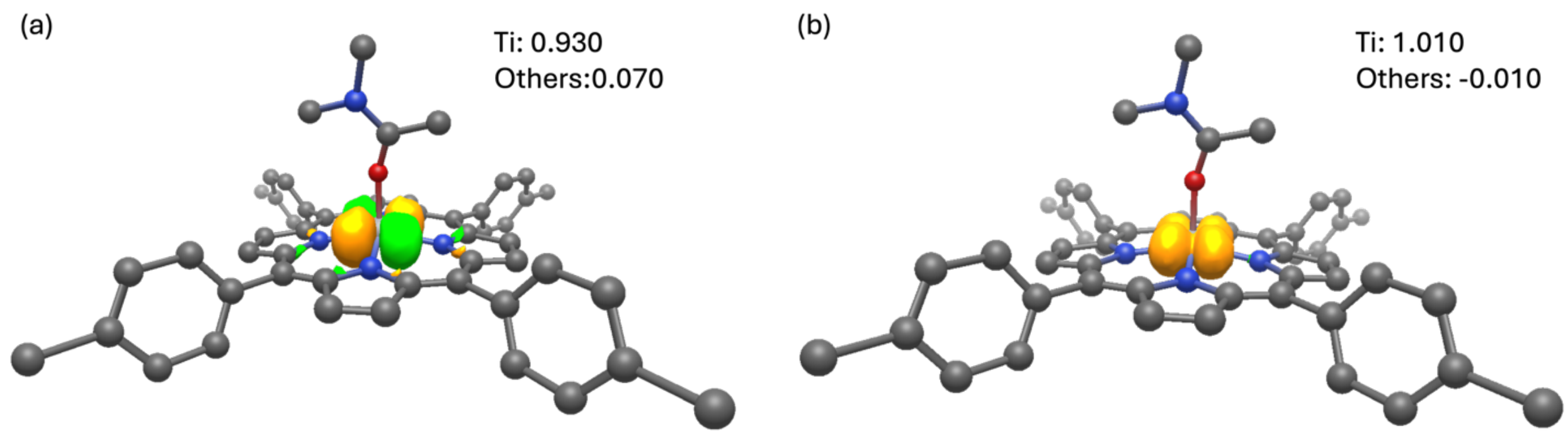

Figure S30. SOMO and spin-density distributions of $[(TTP)Ti(DMA)]^+$, (a) SOMO (α-HOMO, –5.446 eV) and (b) spin-density distribution.

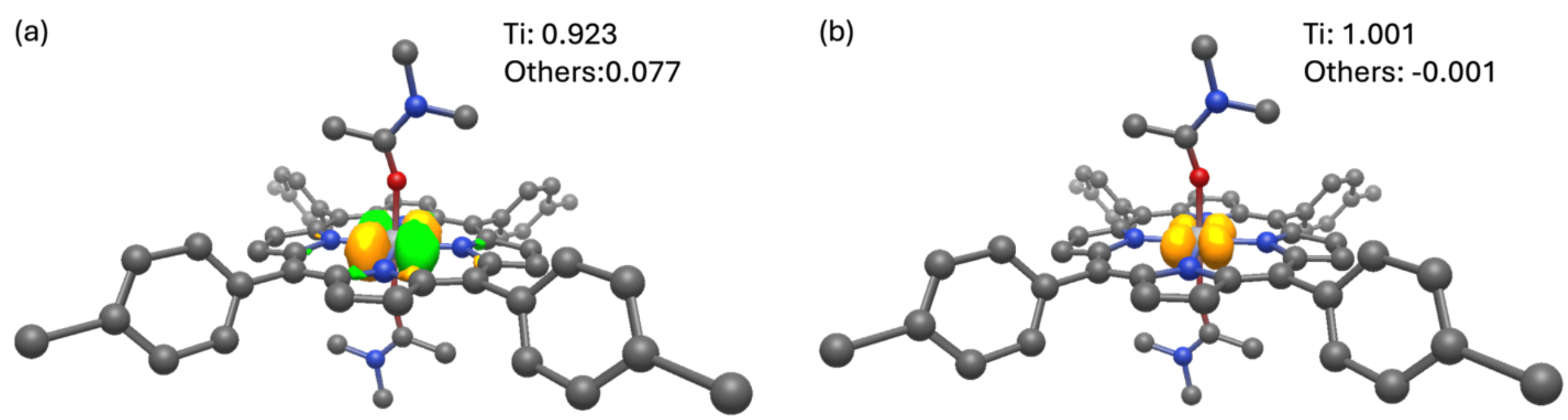

Figure S31. SOMO and spin-density distributions of $[(TTP)Ti(DMA)_2]^+$,(a) SOMO (α-HOMO, –4.970 eV) and (b) spin-density distribution.

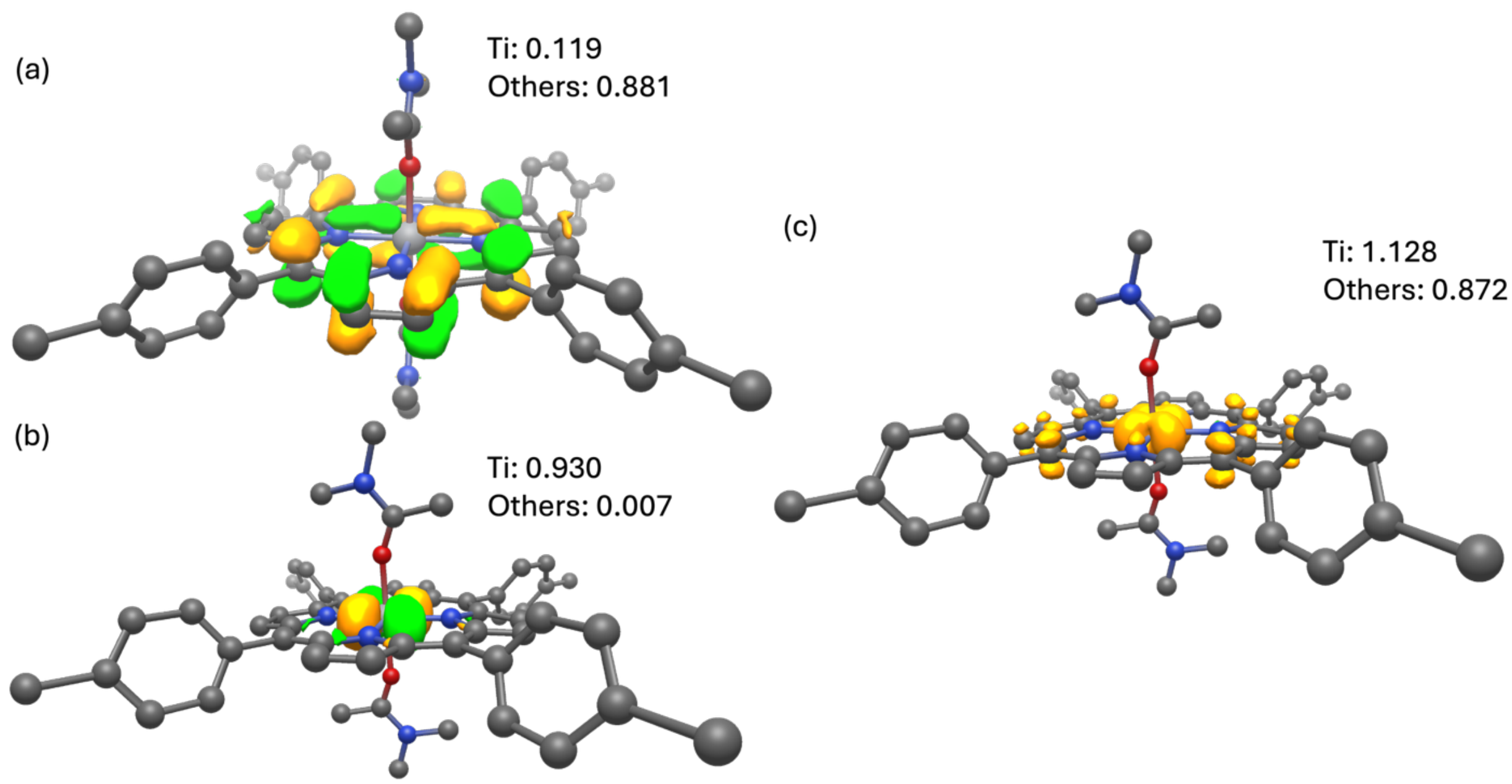

Figure S32. SOMO and spin-density distributions of [(TTP)$Ti^{II}(DMA)_2$] ,(a) α-HOMO, –3.407 eV, (b) α-HOMO–1, –4.300 eV and (c) spin-density distribution.

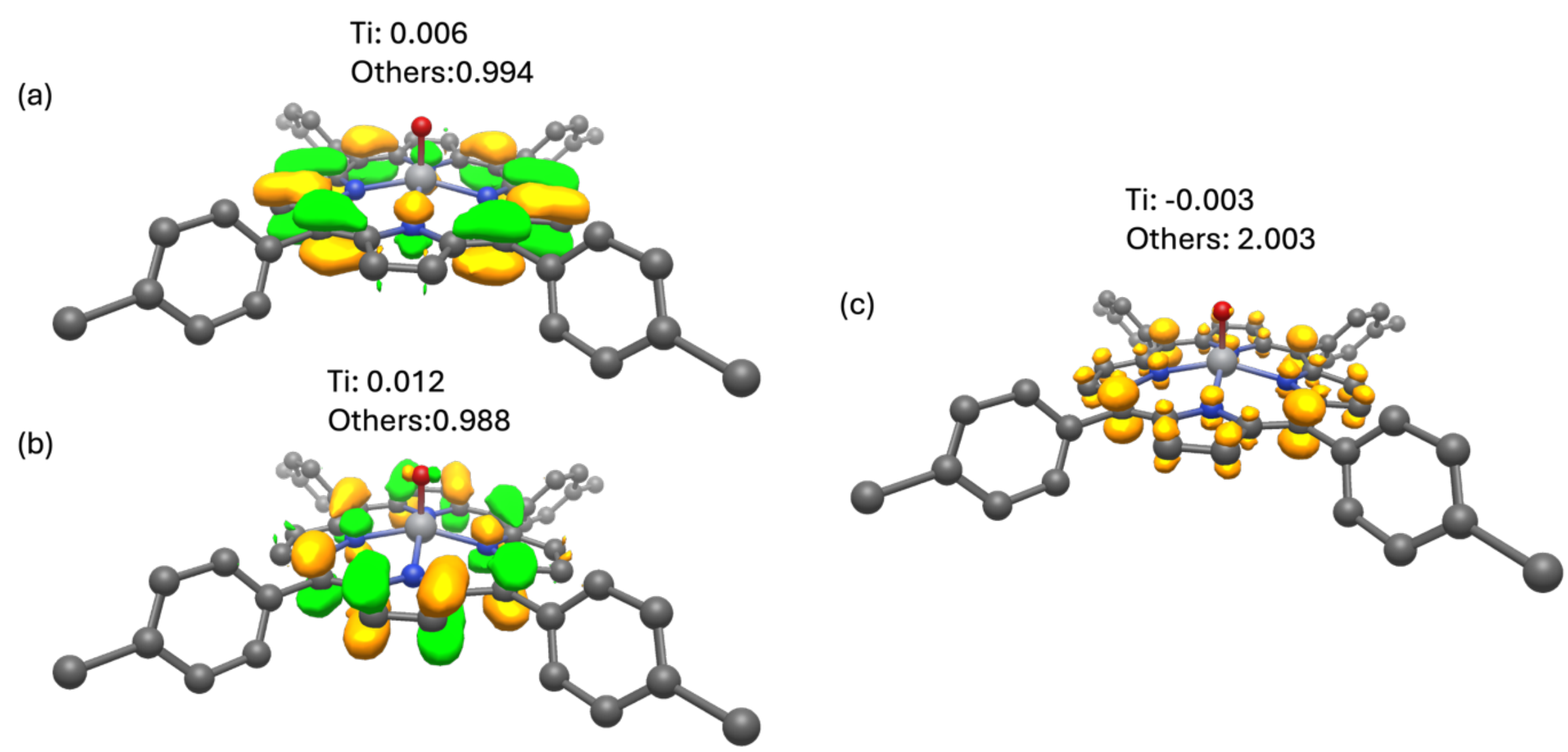

Figure S33. SOMO and spin-density distributions of $[(TTP)TiO]^{2-}$,(a) α-HOMO, –2.657 eV, (b) α-HOMO–1, –2.657 eV and (c) spin-density distribution.

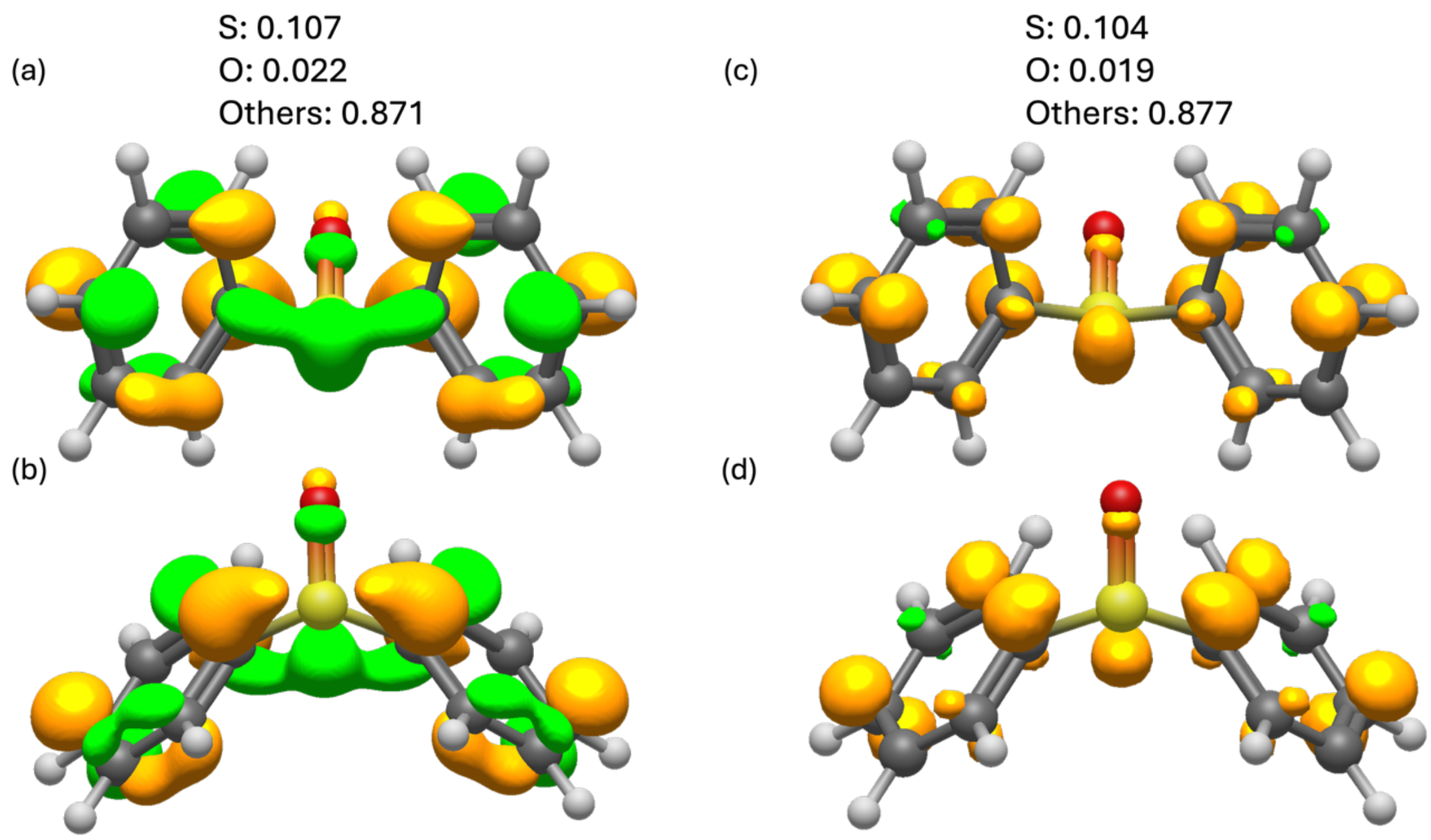

Figure S34. SOMO and spin-density distributions of $Ph2SO^{\bullet-}$, (a) and (b) SOMO (α-HOMO, –2.051 eV) front and back view respectively. (c) and (d) spin-density distribution front and back view, respectively. Isosurface value for HOMO and spin density are 0.05 and 0.005 respectively.

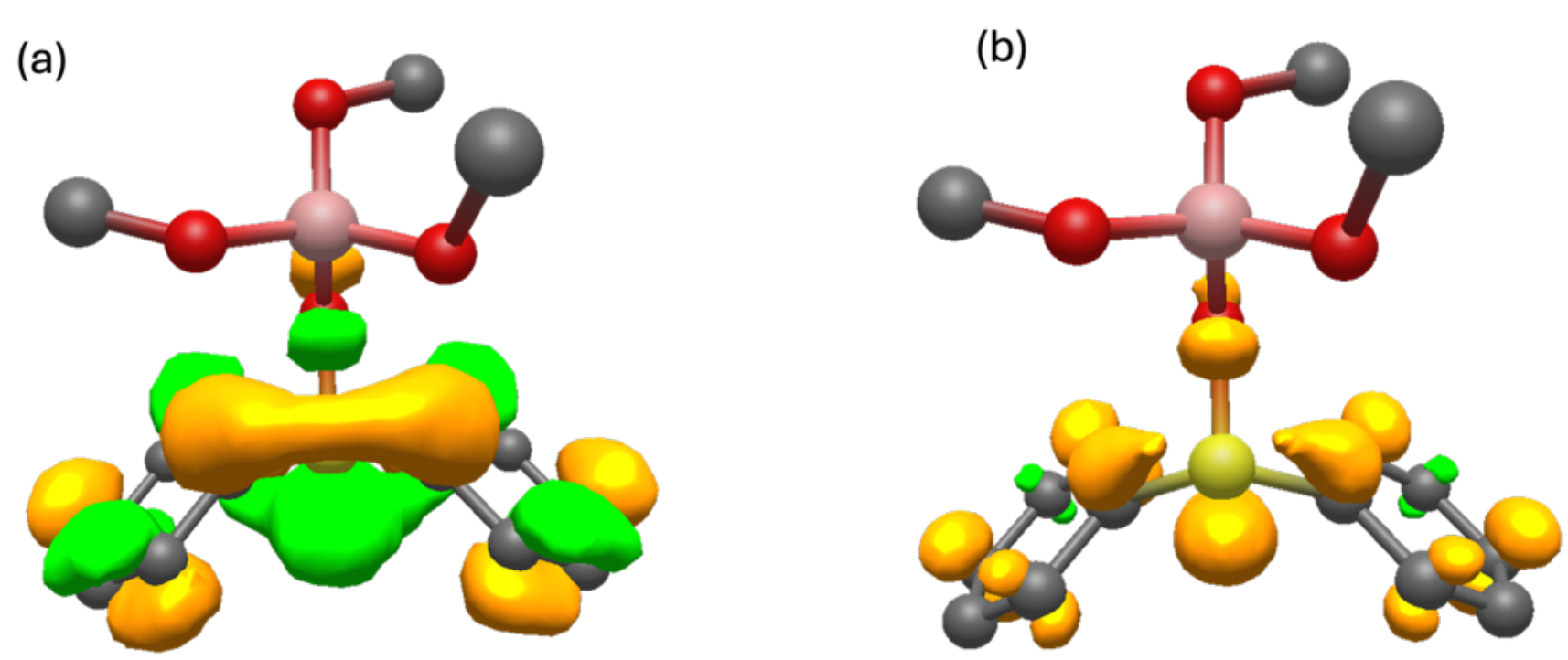

Figure S35. SOMO and spin-density distributions of $\{Ph_2SO[B(OMe)_3]\}^-$,(a) SOMO (α-HOMO, –2.632 eV) and (b) spin-density distribution.

Free energy calculations indicate that explicit coordination of two DMA molecule is thermodynamically more favored (**Figure S36**).

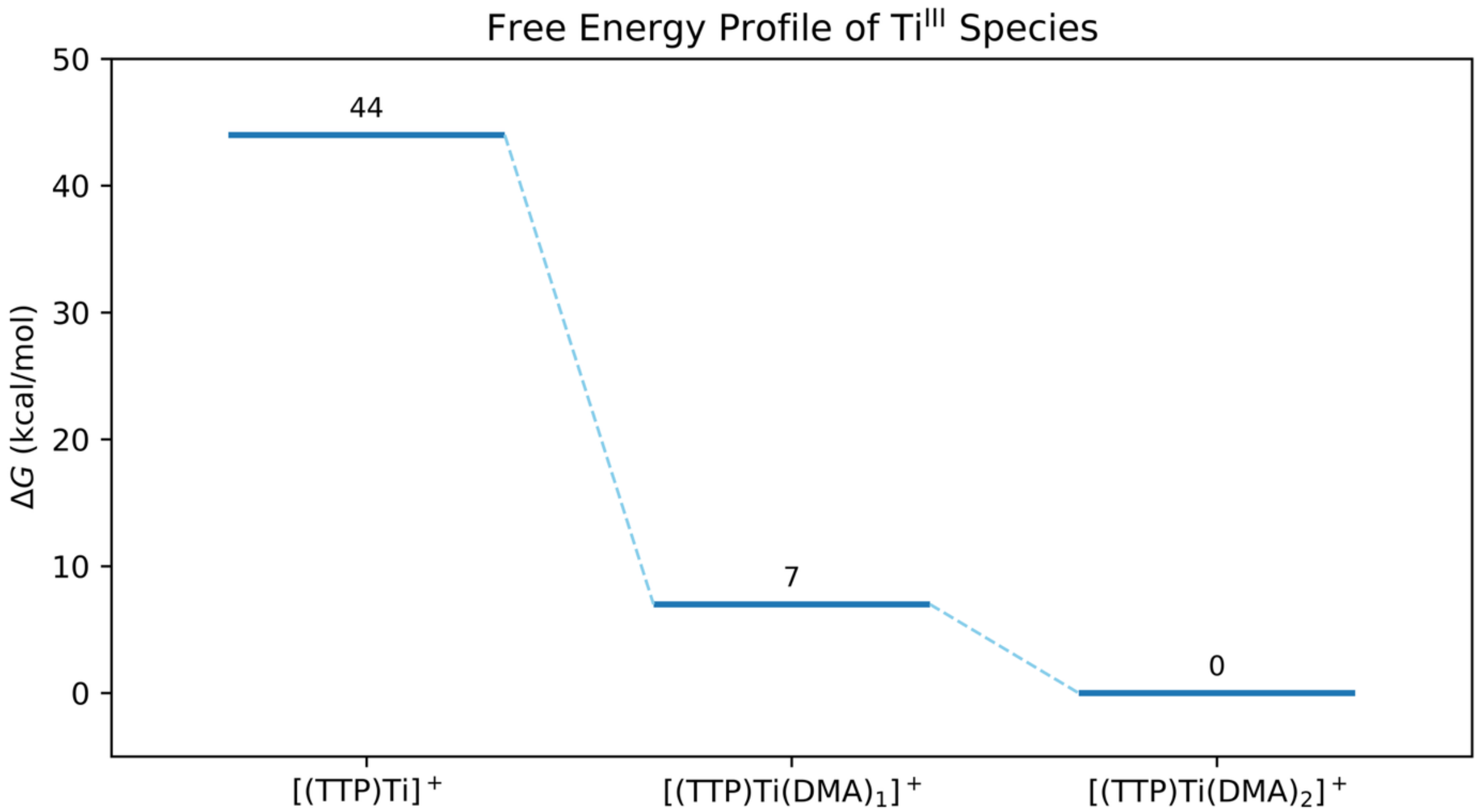

Figure S36. Computed free energy profile of Ti^{III} species under DMA solvent conditions. All states are stoichiometrically balanced; the O atom and free DMA molecules are omitted from the labels for simplicity. All values are referenced to $[(TTP)Ti(DMA)_2]^+$.

7.4. Free Energy

Table S8. Free energies of species of interest.

Species	Electronic Energy(Ha)	Free Energy(Ha)
(TTP)TiO	-2201.9185	-2201.3026
$[(TTP)TiO]^{-}$	-2202.0328	-2201.4209
$[(TTP)TiO]^{2-}$	-2202.1234	-2201.5102
$\{(TTP)TiO[B(OPh)_3]\}$	-3147.1554	-3146.2820
$\{(TTP)TiO[B(OPh)_3](DMA)\}$	-3434.8800	-3433.8974
$\{(TTP)TiO[B(OPh)_3]\}^{-}$	-3147.3055	-3146.4325
$[(TTP)Ti]^{+}$	-2126.4305	-2125.8151
$[(TTP)Ti(DMA)]^{+}$	-2414.2104	-2413.4805
$[(TTP)Ti(DMA)_2]^{+}$	-2701.9415	-2701.1005
$[(TTP)Ti^{II}]$	-2126.5758	-2125.9655
$[(TTP)Ti^{II}(DMA)]$	-2414.3220	-2413.5998
$[(TTP)Ti^{II}(DMA)_2]$	-2702.0596	-2701.2225
Ph_2S	-861.1935	-861.0411
Ph_2SO	-936.4222	-936.2670
$[Ph_2SO]^{-}$	-936.4888	-936.3383
DMA	-287.7202	-287.6076
$B(OPh)_3$	-945.2373	-944.9918
$\{[B(OPh)_3]_2O\}^{2-}$	-1966.0268	-1965.5234
$B(Ph)_3$	-719.34742	-719.10549
$[(BPh_3)_2O]^{2-}$	-1514.2066	-1513.7180
$[B(OPh)_4]^{-}$	-1252.1377	-1251.8054
$Fe(C_5H_5)_2$	-510.0979	-510.1023
$[Fe(C_5H_5)_2]^{+}$	-509.8972	-509.9015
$\{(TTP)TiO[B(Ph)_3]\}$	-2921.2708	-2920.4034
$\{(TTP)TiO[B(Ph)_3]\}^{-}$	-2921.4072	-2920.4034
$\{(TTP)TiO[B(Ph)_3]\}^{2-}$	-2921.4928	-2920.5433
$[Ph_3B\text{-}O\text{-}Ti^{IV}O(TTP)]^{2-}$	-2996.7433	-2995.8777
$[(TTP)Ti(OPh)_2]$	-2740.1425	-2739.3612
$\{(TTP)TiO[B(Ph)_3](DMA)\}$	-3208.9906	-3208.0104
$[(DMA)(TTP)Ti^{IV}(OPh)]^{+}$	-2720.9613	-2720.1485

Table S9. Calculated electrochemical potentials of selected reaction steps referenced to Fc^{+}/Fc. All reported potentials include a 0.7 V shift. This 0.7 V is justified by the relative nature of electrochemical potentials, which are defined with respect to a chosen reference electrode.

Reaction Steps	E_{calc} (V vs Fc^{+}/Fc)	E_{exp} (V vs Fc^{+}/Fc)
$(TTP)TiO + B(OPh)_3 + e^{-} \rightleftharpoons \{(TTP)TiO[B(OPh)_3]\}^{-}$	-1.0	-1.3
$[(TTP)Ti(DMA)_2]^{+} + e^{-} \rightleftharpoons [(TTP)Ti(DMA)_2]$	-1.4	-1.6
$(TTP)TiO + e^{-} \rightleftharpoons [(TTP)TiO]^{-}$	-1.6	-1.5
$[(TTP)TiO]^{-} + e^{-} \rightleftharpoons [(TTP)TiO]^{2-}$	-2.3	-2.0
$(TTP)TiO + B(Ph)_3 + e^{-} \rightleftharpoons \{(TTP)TiO[B(Ph)_3]\}^{-}$	-1.0	-1.3
$\{(TTP)TiO[B(Ph)_3]\}^{-} + e^{-} \rightleftharpoons \{(TTP)TiO[B(Ph)_3]\}^{2-}$	-1.6	-1.8
$Ph_2SO + e^{-} \rightleftharpoons [Ph_2SO]^{-}$	-2.8	-2.8

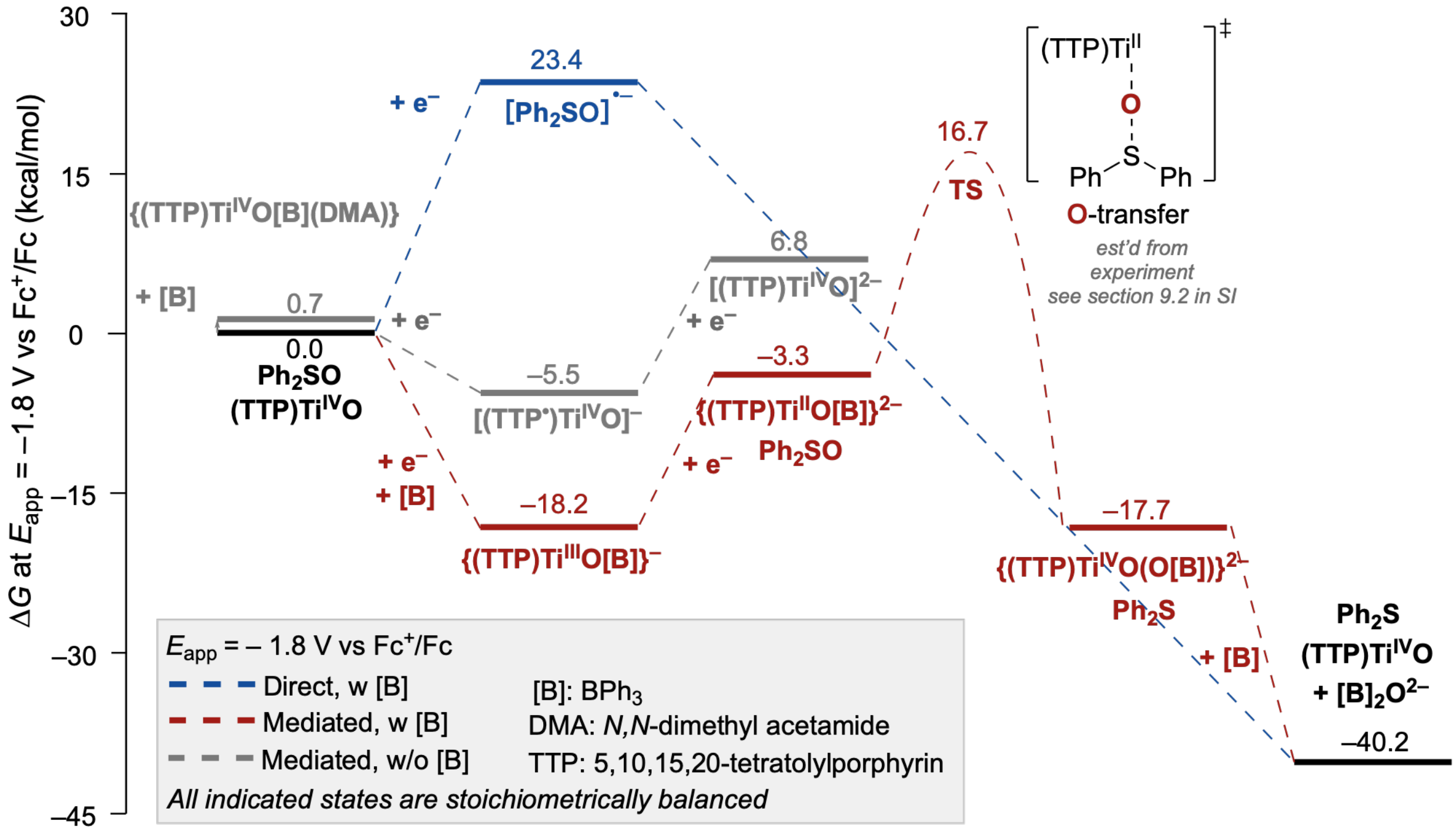

Figure S37. Energy diagram for comparison of three different pathways for electrochemical **1a** deoxygenation: direct, with [B] = BPh_3 (blue); mediated, with [B] (red); mediated, without [B] (gray). Relative free energies were obtained from DFT calculations and corrected based on ΔG = −*n*FE at E_{app} = −1.8 V vs Fc^+/Fc.

7.5. Optimized Geometries

The optimized geometries of the species involved in the free energy calculations are shown below.

$B(Ph)_3$

C	-1.5560560000	0.1962920000	0.0000820000
C	-2.1628500000	1.2819380000	-0.6732980000
C	-2.4137980000	-0.7044980000	0.6732390000
C	-3.5498030000	1.4512540000	-0.6888840000
H	-1.5350740000	1.9993670000	-1.2071980000
C	-3.7993390000	-0.5239360000	0.6883140000
H	-1.9841520000	-1.5553460000	1.2073800000
C	-4.3716470000	0.5516230000	-0.0004330000
H	-3.9920830000	2.2903250000	-1.2325350000
H	-4.4364160000	-1.2268070000	1.2317450000
H	-5.4563640000	0.6885190000	-0.0006680000
C	0.9477150000	1.2491140000	0.0001970000
C	2.1893090000	1.2330950000	-0.6768380000
C	0.5985370000	2.4408360000	0.6770190000
C	3.0294190000	2.3495900000	-0.6926420000
H	2.4950950000	0.3317750000	-1.2134860000
C	1.4478610000	3.5503400000	0.6920140000
H	-0.3516040000	2.4930640000	1.2140180000
C	2.6634040000	3.5096340000	-0.0004780000

H 3.9755710000 2.3141850000 -1.2392230000
H 1.1594190000 4.4523030000 1.2383400000
H 3.3243590000 4.3805450000 -0.0007290000
C 0.6078990000 -1.4454590000 0.0000090000
C -0.0274880000 -2.5137490000 -0.6747560000
C 1.8158930000 -1.7377070000 0.6749840000
C 0.5197680000 -3.7993790000 -0.6898310000
H -0.9618330000 -2.3288800000 -1.2101890000
C 2.3525770000 -3.0277740000 0.6906760000
H 2.3368740000 -0.9401430000 1.2100870000
C 1.7086800000 -4.0611870000 0.0006050000
H 0.0154240000 -4.6019260000 -1.2345910000
H 3.2789390000 -3.2279570000 1.2356530000
H 2.1327220000 -5.0689250000 0.0008610000
B -0.0002710000 -0.0000510000 0.0001330000

$[(BPh_3)_2O]^{2-}$
C 2.0594850000 -1.5106210000 0.2007460000
C 1.3602310000 -2.5748200000 0.8069850000
C 3.3608550000 -1.8220870000 -0.2547190000
C 1.9087860000 -3.8571440000 0.9521840000
H 0.3509750000 -2.3710840000 1.1730550000
C 3.9248160000 -3.0977100000 -0.1243250000
H 3.9603380000 -1.0402040000 -0.7314910000
C 3.1992430000 -4.1301740000 0.4841380000
H 1.3253690000 -4.6507360000 1.4324870000
H 4.9366310000 -3.2892120000 -0.4982360000
H 3.6336020000 -5.1288870000 0.5910500000
C 1.5928570000 0.7099680000 -1.3622670000
C 1.4650480000 2.1083260000 -1.5251670000
C 1.8829130000 -0.0066400000 -2.5416580000
C 1.6017500000 2.7461800000 -2.7638430000
H 1.2447080000 2.7202090000 -0.6459260000
C 2.0257670000 0.6113410000 -3.7927520000
H 1.9963460000 -1.0928540000 -2.4873540000
C 1.8860590000 1.9976170000 -3.9136100000
H 1.4903020000 3.8337160000 -2.8352720000
H 2.2480450000 0.0061450000 -4.6784340000
H 1.9986190000 2.4881330000 -4.8853530000
C 2.2956670000 0.8893080000 1.2443640000
C 3.5812910000 1.4050140000 0.9675300000
C 1.8284220000 1.1002460000 2.5584850000
C 4.3508000000 2.0759600000 1.9269850000
H 3.9954870000 1.2891590000 -0.0392870000
C 2.5827860000 1.7674750000 3.5335490000
H 0.8314250000 0.7284250000 2.8077620000
C 3.8554850000 2.2618270000 3.2239620000
H 5.3411270000 2.4617430000 1.6612290000
H 2.1751450000 1.9069300000 4.5409830000
H 4.4496420000 2.7870830000 3.9779290000
O -0.0031610000 -0.0068110000 0.6027430000

C -2.0660280000 1.5084780000 0.2573890000
C -3.3817380000 1.8262630000 -0.1505010000
C -1.3483970000 2.5625620000 0.8601330000
C -3.9416620000 3.0989080000 0.0195220000
H -3.9967370000 1.0513910000 -0.6190220000
C -1.8926890000 3.8417990000 1.0444900000
H -0.3284840000 2.3528310000 1.1913600000
C -3.1972220000 4.1215960000 0.6219370000
H -4.9652260000 3.2956480000 -0.3178850000
H -1.2947930000 4.6275760000 1.5198230000
H -3.6284180000 5.1178980000 0.7597090000
C -2.3005180000 -0.9354870000 1.2076660000
C -3.5731400000 -1.4665180000 0.9013480000
C -1.8436630000 -1.1773920000 2.5202830000
C -4.3407380000 -2.1788590000 1.8323950000
H -3.9780880000 -1.3291170000 -0.1065340000
C -2.5959800000 -1.8862150000 3.4668830000
H -0.8563140000 -0.7956620000 2.7918430000
C -3.8562780000 -2.3936730000 3.1287780000
H -5.3209680000 -2.5743410000 1.5444280000
H -2.1966660000 -2.0478140000 4.4743410000
H -4.4489590000 -2.9509470000 3.8605990000
C -1.5939730000 -0.6530170000 -1.3853680000
C -1.3978340000 -2.0360790000 -1.6011890000
C -1.9462370000 0.0892930000 -2.5309930000
C -1.5258690000 -2.6349140000 -2.8595080000
H -1.1264310000 -2.6649430000 -0.7488440000
C -2.0829100000 -0.4904520000 -3.8014590000
H -2.1155400000 1.1656260000 -2.4349880000
C -1.8720600000 -1.8616840000 -3.9758350000
H -1.3593170000 -3.7118070000 -2.9731790000
H -2.3555170000 0.1332630000 -4.6598070000
H -1.9784050000 -2.3226400000 -4.9626380000
B -1.3871230000 -0.0176340000 0.1419150000
B 1.3802150000 0.0190140000 0.1413580000

$[B(OPh)_4]^-$
O -0.8066950000 0.6255910000 1.0625650000
O -0.8035880000 -0.6294220000 -1.0565090000
O 0.8090320000 -1.0600860000 0.6324200000
C -1.7332240000 0.0230090000 1.8333530000
C -2.3564610000 0.8118720000 2.8239000000
C -2.1194430000 -1.3279220000 1.7095740000
C -3.3318760000 0.2698370000 3.6615580000
H -2.0562950000 1.8583380000 2.9188370000
C -3.0988780000 -1.8586810000 2.5558750000
H -1.6503230000 -1.9524420000 0.9509910000
C -3.7134250000 -1.0721660000 3.5360440000
H -3.7990490000 0.9034200000 4.4211610000
H -3.3839790000 -2.9089350000 2.4429950000
H -4.4772850000 -1.4968450000 4.1920060000

C -1.7206840000 -0.0254150000 -1.8372130000
C -2.1138940000 1.3232640000 -1.7111590000
C -2.3281750000 -0.8114300000 -2.8397660000
C -3.0845770000 1.8546110000 -2.5671130000
H -1.6571270000 1.9455590000 -0.9432070000
C -3.2951830000 -0.2688840000 -3.6868110000
H -2.0224880000 -1.8561310000 -2.9364970000
C -3.6837610000 1.0708670000 -3.5589470000
H -3.3753470000 2.9030890000 -2.4521910000
H -3.7501150000 -0.9002950000 -4.4555980000
H -4.4410370000 1.4960440000 -4.2221770000
C 1.7297870000 -1.8372350000 0.0293470000
C 2.3415660000 -2.8363100000 0.8165110000
C 2.1229320000 -1.7109210000 -1.3193470000
C 3.3120400000 -3.6800230000 0.2749970000
H 2.0362750000 -2.9331270000 1.8613170000
C 3.0971590000 -2.5634740000 -1.8496660000
H 1.6634560000 -0.9452420000 -1.9424300000
C 3.7001690000 -3.5521740000 -1.0648930000
H 3.7700660000 -4.4462010000 0.9073430000
H 3.3875880000 -2.4484020000 -2.8982270000
H 4.4601530000 -4.2128050000 -1.4892770000
O 0.8076970000 1.0589860000 -0.6237350000
C 1.7265690000 1.8377070000 -0.0198330000
C 2.3378440000 2.8372160000 -0.8068510000
C 2.1173280000 1.7133770000 1.3296600000
C 3.3058260000 3.6831990000 -0.2644460000
H 2.0340540000 2.9325990000 -1.8522440000
C 3.0890160000 2.5682650000 1.8609080000
H 1.6579960000 0.9476380000 1.9527820000
C 3.6917310000 3.5572510000 1.0762830000
H 3.7635180000 4.4496510000 -0.8967020000
H 3.3776650000 2.4548030000 2.9101310000
H 4.4497010000 4.2196470000 1.5015110000
B 0.0013360000 -0.0011910000 0.0039540000

{(TTP)TiO[B(Ph)$_3$]}
N -0.7655750000 2.0136410000 0.1452560000
N -1.6410230000 -1.9496500000 0.2036770000
N -2.9271200000 0.4244690000 1.1724150000
N 0.5241400000 -0.3638530000 -0.8213320000
Ti -0.9477940000 -0.0171700000 0.6363760000
C -1.5376380000 3.0409330000 0.6636400000
C -2.7312900000 -2.5533910000 0.8109070000
C 0.3701100000 2.6083980000 -0.3804810000
C -0.8934300000 -2.9727320000 -0.3577170000
C -3.4542470000 1.6763090000 1.4397550000
C -3.8978080000 -0.4924680000 1.5364010000
C 1.4233230000 0.5678380000 -1.3122300000
C 0.9105250000 -1.5883630000 -1.3401010000
C -2.8080870000 2.9097530000 1.2486590000

C	-3.8034860000	-1.8911980000	1.4323570000
C	1.4014900000	1.9538990000	-1.0749750000
C	0.2844800000	-2.8253720000	-1.1095150000
C	-0.8513460000	4.2950130000	0.4926660000
C	-2.6353170000	-3.9824280000	0.6660210000
C	0.3244990000	4.0283860000	-0.1463140000
C	-1.5084060000	-4.2399280000	-0.0598250000
C	-4.7936610000	1.5380720000	1.9497300000
C	-5.0649020000	0.2019500000	2.0140920000
C	2.3809720000	-0.0864460000	-2.1650600000
C	2.0614780000	-1.4130480000	-2.1870710000
H	-1.2231080000	5.2606720000	0.8234920000
H	-3.3457130000	-4.7005470000	1.0656080000
H	1.0967210000	4.7350600000	-0.4363650000
H	-1.1221020000	-5.2085500000	-0.3641240000
H	-5.4500140000	2.3631160000	2.2118050000
H	-5.9857730000	-0.2748670000	2.3378660000
H	3.1827910000	0.4127930000	-2.7016630000
H	2.5534890000	-2.2052280000	-2.7441730000
C	2.5239260000	2.7779360000	-1.6261230000
C	3.8254770000	2.6535460000	-1.1137020000
C	2.3090500000	3.6911370000	-2.6744260000
C	4.8740610000	3.4226410000	-1.6275480000
H	4.0205280000	1.9518350000	-0.2990540000
C	3.3607660000	4.4511430000	-3.1871710000
H	1.3077720000	3.7987420000	-3.0988540000
C	4.6642280000	4.3350260000	-2.6726520000
H	5.8760410000	3.3094330000	-1.2045270000
H	3.1657580000	5.1479360000	-4.0075540000
C	-3.5225290000	4.1548540000	1.6764280000
C	-3.7326900000	4.4367780000	3.0357310000
C	-4.0070280000	5.0717280000	0.7262000000
C	-4.4017270000	5.5991630000	3.4303410000
H	-3.3625790000	3.7430850000	3.7945780000
C	-4.6792830000	6.2273070000	1.1260760000
H	-3.8636890000	4.8707830000	-0.3384750000
C	-4.8878410000	6.5164770000	2.4860410000
H	-4.5471520000	5.7956140000	4.4962250000
H	-5.0528620000	6.9184880000	0.3649950000
C	-4.9257590000	-2.7138090000	1.9855910000
C	-5.1471630000	-2.7820010000	3.3711620000
C	-5.7908930000	-3.4289090000	1.1385580000
C	-6.1958730000	-3.5468110000	3.8895560000
H	-4.4888350000	-2.2356790000	4.0513430000
C	-6.8400780000	-4.1856390000	1.6628640000
H	-5.6473290000	-3.3814930000	0.0561890000
C	-7.0615150000	-4.2639000000	3.0487090000
H	-6.3429620000	-3.5855420000	4.9725780000
H	-7.5037390000	-4.7239690000	0.9800630000
C	0.8985180000	-4.0515640000	-1.7111510000
C	0.2489480000	-4.7522150000	-2.7419630000

C	2.1439170000	-4.5293020000	-1.2681890000
C	0.8273160000	-5.8902440000	-3.3085660000
H	-0.7148590000	-4.3948640000	-3.1130850000
C	2.7144080000	-5.6712800000	-1.8350150000
H	2.6670990000	-4.0057180000	-0.4646620000
C	2.0684800000	-6.3755690000	-2.8644950000
H	0.3038230000	-6.4092160000	-4.1165450000
H	3.6830990000	-6.0221820000	-1.4679540000
C	5.7905790000	5.1696740000	-3.2304140000
H	5.8974650000	5.0198190000	-4.3173850000
H	5.6029700000	6.2452630000	-3.0741820000
H	6.7512310000	4.9219690000	-2.7561690000
C	-5.5989030000	7.7790060000	2.9067070000
H	-6.5399060000	7.9134680000	2.3494520000
H	-5.8335700000	7.7726590000	3.9810480000
H	-4.9781720000	8.6686900000	2.7046000000
C	-8.1803450000	-5.1076780000	3.6079180000
H	-9.0998630000	-5.0036040000	3.0108500000
H	-7.9101000000	-6.1779670000	3.5973750000
H	-8.4109030000	-4.8358950000	4.6485020000
C	2.6744450000	-7.6250140000	-3.4543880000
H	2.3497840000	-8.5207510000	-2.8962460000
H	2.3684610000	-7.7668620000	-4.5018940000
H	3.7738770000	-7.5987340000	-3.4139710000
O	-0.1531490000	-0.1706600000	2.1112630000
C	1.6008470000	-1.5195720000	3.4218280000
C	2.8677910000	-1.4629990000	4.0362510000
C	1.2800770000	-2.7228010000	2.7593790000
C	3.7637020000	-2.5400630000	3.9982830000
H	3.1656710000	-0.5516850000	4.5617220000
C	2.1668360000	-3.8044320000	2.7067720000
H	0.3059320000	-2.8205780000	2.2730720000
C	3.4181020000	-3.7182670000	3.3294450000
H	4.7373550000	-2.4564140000	4.4907480000
H	1.8796820000	-4.7197760000	2.1808500000
H	4.1144450000	-4.5607990000	3.2934050000
C	1.2545970000	1.1374750000	3.8164960000
C	1.1935550000	1.7867480000	5.0651430000
C	2.0425420000	1.7601540000	2.8257790000
C	1.8759940000	2.9854900000	5.3161170000
H	0.5958480000	1.3463840000	5.8675890000
C	2.7222270000	2.9606860000	3.0577250000
H	2.1301480000	1.2906200000	1.8420840000
C	2.6425340000	3.5815750000	4.3104750000
H	1.8049320000	3.4565970000	6.3012920000
H	3.3184140000	3.4128200000	2.2593870000
H	3.1732680000	4.5190270000	4.4992140000
C	-0.6863730000	-0.6115140000	4.5771790000
C	-0.6705660000	-1.7017870000	5.4684470000
C	-1.8091580000	0.2410370000	4.6504780000
C	-1.7089990000	-1.9332000000	6.3819190000

H 0.1764340000 -2.3923930000 5.4526830000
C -2.8560380000 0.0198400000 5.5510850000
H -1.8643680000 1.1068910000 3.9847900000
C -2.8103470000 -1.0735560000 6.4259840000
H -1.6567980000 -2.7901600000 7.0604150000
H -3.7104210000 0.7029420000 5.5739270000
H -3.6237100000 -1.2496270000 7.1355720000
B 0.5272790000 -0.2953370000 3.5303640000

$\{(TTP)TiO[B(Ph)_3]\}^-$
N -0.8168640000 2.0345740000 0.0986700000
N -1.6130500000 -1.9936000000 0.1469220000
N -2.9318240000 0.3733410000 1.2083360000
N 0.5339690000 -0.3382000000 -0.9074920000
Ti -0.8877940000 -0.0364070000 0.6890050000
C -1.5976460000 3.0320370000 0.6401170000
C -2.6963410000 -2.6094760000 0.7346630000
C 0.2698120000 2.6505290000 -0.4821290000
C -0.8543650000 -2.9881740000 -0.4298980000
C -3.4685520000 1.6078590000 1.4927760000
C -3.8812600000 -0.5624780000 1.5475720000
C 1.3703850000 0.6174350000 -1.4372820000
C 0.9324660000 -1.5490260000 -1.4258720000
C -2.8439670000 2.8568990000 1.2806980000
C -3.7673260000 -1.9613780000 1.3880520000
C 1.2971720000 2.0100610000 -1.2092430000
C 0.3290050000 -2.8022110000 -1.1779460000
C -0.9578640000 4.3138880000 0.4347390000
C -2.5929750000 -4.0424530000 0.5602890000
C 0.1955750000 4.0782390000 -0.2566700000
C -1.4572580000 -4.2759940000 -0.1606140000
C -4.8068860000 1.4454940000 2.0198760000
C -5.0605440000 0.1044290000 2.0569300000
C 2.3268300000 -0.0115870000 -2.3231170000
C 2.0555400000 -1.3496150000 -2.3170570000
H -1.3380090000 5.2724990000 0.7777180000
H -3.2965080000 -4.7781380000 0.9409260000
H 0.9309970000 4.8091620000 -0.5820230000
H -1.0621550000 -5.2377820000 -0.4766540000
H -5.4754110000 2.2534200000 2.3055340000
H -5.9748720000 -0.3877910000 2.3780890000
H 3.0903470000 0.5055930000 -2.8982590000
H 2.5567650000 -2.1283460000 -2.8858990000
C 2.3667370000 2.8664850000 -1.8164430000
C 3.7003580000 2.7768760000 -1.3781860000
C 2.0769020000 3.7741170000 -2.8497010000
C 4.6998590000 3.5699510000 -1.9462520000
H 3.9565720000 2.0767540000 -0.5791070000
C 3.0808400000 4.5624080000 -3.4186560000
H 1.0525840000 3.8555140000 -3.2218570000
C 4.4110100000 4.4806290000 -2.9764790000

H	5.7268430000	3.4784260000	-1.5806460000
H	2.8241860000	5.2521050000	-4.2280020000
C	-3.5724430000	4.0828910000	1.7419160000
C	-3.7738570000	4.3317690000	3.1112020000
C	-4.0860790000	5.0149010000	0.8237810000
C	-4.4566030000	5.4711070000	3.5427120000
H	-3.3867920000	3.6223380000	3.8464400000
C	-4.7730480000	6.1511620000	1.2595890000
H	-3.9560720000	4.8416450000	-0.2474740000
C	-4.9686840000	6.4052790000	2.6266800000
H	-4.5942130000	5.6376140000	4.6151440000
H	-5.1694500000	6.8526480000	0.5197230000
C	-4.8807980000	-2.8104490000	1.9227410000
C	-5.1026530000	-2.9171000000	3.3073180000
C	-5.7416590000	-3.5148870000	1.0636150000
C	-6.1417600000	-3.7037510000	3.8098240000
H	-4.4480780000	-2.3804760000	3.9988060000
C	-6.7827810000	-4.2968780000	1.5709040000
H	-5.6012470000	-3.4402950000	-0.0177130000
C	-7.0017140000	-4.4113120000	2.9533360000
H	-6.2869270000	-3.7690810000	4.8921950000
H	-7.4417390000	-4.8255110000	0.8759260000
C	0.9776610000	-4.0135270000	-1.7762450000
C	0.3464520000	-4.7529930000	-2.7912210000
C	2.2472040000	-4.4407680000	-1.3470160000
C	0.9603030000	-5.8763690000	-3.3517950000
H	-0.6344180000	-4.4372330000	-3.1553240000
C	2.8552370000	-5.5666000000	-1.9069100000
H	2.7624520000	-3.8845030000	-0.5601200000
C	2.2240910000	-6.3091030000	-2.9190280000
H	0.4468800000	-6.4248540000	-4.1468800000
H	3.8419360000	-5.8746470000	-1.5487180000
C	5.4896060000	5.3547380000	-3.5681630000
H	5.2205370000	5.7022650000	-4.5768560000
H	5.6568640000	6.2515880000	-2.9461000000
H	6.4515820000	4.8227770000	-3.6303850000
C	-5.6815260000	7.6488560000	3.0989520000
H	-6.3511570000	8.0503330000	2.3236350000
H	-6.2774260000	7.4537390000	4.0039530000
H	-4.9601780000	8.4452990000	3.3532630000
C	-8.1047600000	-5.2829060000	3.5023450000
H	-8.9202980000	-5.4098910000	2.7745520000
H	-7.7264500000	-6.2907610000	3.7484030000
H	-8.5286250000	-4.8632470000	4.4276450000
C	2.8730640000	-7.5410650000	-3.5009450000
H	2.6814990000	-8.4250810000	-2.8675920000
H	2.4832750000	-7.7677870000	-4.5046030000
H	3.9658320000	-7.4267870000	-3.5715110000
O	-0.0271590000	-0.1883050000	2.2215020000
C	1.4122750000	-1.6786770000	3.7524240000
C	2.1333400000	-1.9880420000	4.9270570000

C 1.4688120000 -2.6403830000 2.7249820000
C 2.8583890000 -3.1756930000 5.0727890000
H 2.1309530000 -1.2762220000 5.7583330000
C 2.1921070000 -3.8356980000 2.8533610000
H 0.9248730000 -2.4478820000 1.7975330000
C 2.8934510000 -4.1117980000 4.0309540000
H 3.4013740000 -3.3734650000 6.0027500000
H 2.2045080000 -4.5552270000 2.0283550000
H 3.4593870000 -5.0418440000 4.1377060000
C 1.6026980000 0.9809210000 3.8288270000
C 1.9249950000 1.4897380000 5.1054960000
C 2.2804520000 1.5719230000 2.7421680000
C 2.8635480000 2.5130260000 5.2919860000
H 1.4179130000 1.0826000000 5.9856330000
C 3.2189100000 2.5986120000 2.9101060000
H 2.0595910000 1.2190000000 1.7310550000
C 3.5189250000 3.0762020000 4.1911120000
H 3.0800900000 2.8773630000 6.3015120000
H 3.7163070000 3.0309070000 2.0358920000
H 4.2494340000 3.8786770000 4.3291730000
C -0.7012430000 -0.2222170000 4.6709060000
C -1.2255350000 -1.3653820000 5.3068430000
C -1.3661510000 0.9935440000 4.9449290000
C -2.3376810000 -1.3091420000 6.1601520000
H -0.7482590000 -2.3345750000 5.1361360000
C -2.4759000000 1.0693220000 5.7944250000
H -0.9981140000 1.9142890000 4.4813720000
C -2.9705600000 -0.0873300000 6.4102440000
H -2.7078960000 -2.2233920000 6.6352290000
H -2.9556370000 2.0350630000 5.9840040000
H -3.8347500000 -0.0346720000 7.0787890000
B 0.5585780000 -0.2748860000 3.6010880000

{(TTP)TiO[B(Ph)$_3$]}$^{2-}$
N -0.7941170000 2.0386430000 0.1748990000
N -1.5810550000 -1.9874820000 0.2013760000
N -2.8838260000 0.3855210000 1.2387520000
N 0.5570790000 -0.3011200000 -0.8012370000
Ti -0.9336290000 -0.0363440000 0.6438360000
C -1.5456750000 3.0404630000 0.7884860000
C -2.6476140000 -2.6264390000 0.8331840000
C 0.3457290000 2.6659420000 -0.3248450000
C -0.7919630000 -2.9977460000 -0.3453190000
C -3.4727200000 1.6379920000 1.4827940000
C -3.8427070000 -0.5760730000 1.6039540000
C 1.3872090000 0.6732380000 -1.3798060000
C 0.9753300000 -1.5364580000 -1.3295830000
C -2.8662410000 2.8670620000 1.3380850000
C -3.7426060000 -1.9481790000 1.4779330000
C 1.3531490000 2.0303450000 -1.1363540000
C 0.4075730000 -2.7769380000 -1.1128270000

C	-0.8560650000	4.2675910000	0.7191230000
C	-2.5123920000	-4.0214770000	0.7029030000
C	0.3316690000	4.0323570000	0.0203670000
C	-1.3471530000	-4.2544390000	-0.0396620000
C	-4.8318340000	1.4395390000	1.9632190000
C	-5.0471060000	0.1060240000	2.0579560000
C	2.3171420000	0.0256390000	-2.2932620000
C	2.0784410000	-1.3063580000	-2.2526830000
H	-1.2105610000	5.2156460000	1.1166180000
H	-3.1926190000	-4.7646560000	1.1118730000
H	1.0986070000	4.7583630000	-0.2384620000
H	-0.9296190000	-5.2170350000	-0.3251330000
H	-5.5317370000	2.2403990000	2.1897100000
H	-5.9589410000	-0.3957900000	2.3726510000
H	3.0487690000	0.5474360000	-2.9054380000
H	2.5765420000	-2.0842350000	-2.8261980000
C	2.3912770000	2.9065720000	-1.7617410000
C	3.7557820000	2.7696680000	-1.4505300000
C	2.0440000000	3.9022220000	-2.6978660000
C	4.7271720000	3.5794060000	-2.0514640000
H	4.0622990000	2.0132040000	-0.7236520000
C	3.0126210000	4.7079550000	-3.2965540000
H	0.9929710000	4.0364840000	-2.9656260000
C	4.3776170000	4.5647370000	-2.9855590000
H	5.7790590000	3.4418840000	-1.7831430000
H	2.7047140000	5.4618010000	-4.0280560000
C	-3.6065900000	4.0989170000	1.7530090000
C	-3.9642720000	4.3271850000	3.0931500000
C	-3.9780320000	5.0809940000	0.8121980000
C	-4.6684710000	5.4755830000	3.4746700000
H	-3.6838970000	3.5915630000	3.8511870000
C	-4.6807070000	6.2242290000	1.1932830000
H	-3.7157430000	4.9361910000	-0.2389520000
C	-5.0410390000	6.4473130000	2.5352070000
H	-4.9289020000	5.6184360000	4.5278470000
H	-4.9618160000	6.9591290000	0.4323150000
C	-4.8599410000	-2.7991880000	1.9962800000
C	-5.1353760000	-2.8816910000	3.3719630000
C	-5.6801370000	-3.5467940000	1.1281110000
C	-6.1882520000	-3.6662360000	3.8577730000
H	-4.5117600000	-2.3220990000	4.0738060000
C	-6.7296360000	-4.3277770000	1.6137710000
H	-5.4934400000	-3.5051350000	0.0519800000
C	-7.0059310000	-4.4055690000	2.9912540000
H	-6.3735070000	-3.7053910000	4.9355720000
H	-7.3534400000	-4.8859790000	0.9084590000
C	1.0462910000	-3.9807150000	-1.7315770000
C	0.3882140000	-4.7311850000	-2.7264620000
C	2.3276670000	-4.4106960000	-1.3453330000
C	0.9880870000	-5.8474550000	-3.3103200000
H	-0.6093660000	-4.4247160000	-3.0514370000

C	2.9285260000	-5.5311550000	-1.9318630000
H	2.8627040000	-3.8572910000	-0.5694270000
C	2.2726320000	-6.2731500000	-2.9243350000
H	0.4507340000	-6.3990170000	-4.0882740000
H	3.9276670000	-5.8352380000	-1.6053030000
C	5.4163490000	5.4476880000	-3.6342400000
H	5.3549420000	5.3991190000	-4.7342890000
H	5.2745930000	6.5058590000	-3.3552640000
H	6.4354000000	5.1571570000	-3.3388210000
C	-5.7951500000	7.6919850000	2.9372480000
H	-6.7357220000	7.7928440000	2.3701910000
H	-6.0451900000	7.6850370000	4.0084900000
H	-5.2044830000	8.6018950000	2.7356570000
C	-8.1358260000	-5.2639920000	3.5072390000
H	-9.0844060000	-5.0311640000	2.9956710000
H	-7.9364990000	-6.3355790000	3.3339660000
H	-8.2891880000	-5.1237840000	4.5875400000
C	2.9074480000	-7.4893340000	-3.5548170000
H	2.3454550000	-8.4071370000	-3.3106260000
H	2.9233010000	-7.4105200000	-4.6545860000
H	3.9425890000	-7.6295840000	-3.2099030000
O	-0.1115260000	-0.2885900000	2.1528820000
C	1.3328300000	-1.7674220000	3.7024210000
C	2.0463740000	-2.0641580000	4.8849680000
C	1.3896430000	-2.7396370000	2.6857710000
C	2.7682370000	-3.2511670000	5.0474920000
H	2.0401780000	-1.3431080000	5.7080290000
C	2.1105810000	-3.9344610000	2.8326170000
H	0.8482830000	-2.5628770000	1.7549700000
C	2.8065680000	-4.1984310000	4.0158270000
H	3.3058730000	-3.4395110000	5.9824420000
H	2.1236720000	-4.6629470000	2.0156920000
H	3.3702840000	-5.1282660000	4.1355720000
C	1.5785340000	0.8769200000	3.7053670000
C	1.9270220000	1.4174970000	4.9618870000
C	2.2703930000	1.4003390000	2.5939050000
C	2.9057670000	2.4090730000	5.1068880000
H	1.4115960000	1.0603520000	5.8583350000
C	3.2476630000	2.3960940000	2.7215040000
H	2.0312410000	1.0247010000	1.5962000000
C	3.5745210000	2.9066520000	3.9828900000
H	3.1430270000	2.7997500000	6.1017370000
H	3.7531190000	2.7791150000	1.8295030000
H	4.3362410000	3.6847340000	4.0881270000
C	-0.7306730000	-0.2507090000	4.6337170000
C	-1.2726530000	-1.3724780000	5.2925680000
C	-1.3385280000	0.9899810000	4.9265330000
C	-2.3470220000	-1.2714780000	6.1884230000
H	-0.8406710000	-2.3594940000	5.1054230000
C	-2.4099250000	1.1099200000	5.8189020000
H	-0.9584750000	1.8932130000	4.4398560000

C -2.9214410000 -0.0254610000 6.4594150000
H -2.7335230000 -2.1698550000 6.6802200000
H -2.8459840000 2.0936170000 6.0206290000
H -3.7556030000 0.0615000000 7.1616580000
B 0.5022760000 -0.3568130000 3.5431530000

$[Ph_3B\text{-}O\text{-}Ti^{IV}O(TTP)]^{2-}$
N -0.7306400000 2.0780720000 0.1884820000
N -1.5613230000 -2.0791340000 0.2345730000
N -2.9283090000 0.3675930000 1.3131940000
N 0.6552170000 -0.3711040000 -0.8316030000
C -1.5126100000 3.0461040000 0.7546140000
C -2.6505050000 -2.6601710000 0.8217710000
C 0.3506850000 2.6648800000 -0.4016430000
C -0.7900050000 -3.0491550000 -0.3379980000
C -3.4144640000 1.6031040000 1.6276110000
C -3.8518530000 -0.5787030000 1.6521480000
C 1.4752980000 0.5949720000 -1.3480810000
C 1.0382620000 -1.5886920000 -1.3246450000
C -2.7630390000 2.8483110000 1.4038630000
C -3.7250480000 -1.9838790000 1.4639940000
C 1.3699330000 1.9977490000 -1.1362080000
C 0.4044400000 -2.8392580000 -1.0820920000
C -0.8594760000 4.3326320000 0.5547260000
C -2.5393560000 -4.1030620000 0.6571720000
C 0.2835480000 4.0986770000 -0.1564240000
C -1.3982320000 -4.3417160000 -0.0559720000
C -4.7455550000 1.4380940000 2.1966670000
C -5.0138240000 0.0983380000 2.2125730000
C 2.4509750000 -0.0486550000 -2.2153490000
C 2.1829150000 -1.3881280000 -2.2009560000
H -1.2233300000 5.2913530000 0.9157880000
H -3.2396950000 -4.8407060000 1.0409730000
H 1.0190850000 4.8323990000 -0.4764570000
H -1.0018690000 -5.3086560000 -0.3555810000
H -5.3968200000 2.2423710000 2.5298280000
H -5.9226740000 -0.3858550000 2.5612440000
H 3.2282430000 0.4587190000 -2.7810870000
H 2.7031710000 -2.1670970000 -2.7525320000
C 2.4227060000 2.8570410000 -1.7697470000
C 3.7723680000 2.7646300000 -1.3823690000
C 2.0989050000 3.7781470000 -2.7818990000
C 4.7517410000 3.5631650000 -1.9779300000
H 4.0574130000 2.0572470000 -0.5998780000
C 3.0815670000 4.5731370000 -3.3779090000
H 1.0620380000 3.8635070000 -3.1161520000
C 4.4272160000 4.4858930000 -2.9863770000
H 5.7912260000 3.4670890000 -1.6504040000
H 2.7957520000 5.2720450000 -4.1695910000
C -3.4791530000 4.0777830000 1.8798880000
C -3.6851190000 4.3167470000 3.2501910000

C	-3.9779090000	5.0279480000	0.9705670000
C	-4.3567660000	5.4602120000	3.6907960000
H	-3.3106240000	3.5957990000	3.9803850000
C	-4.6522630000	6.1681680000	1.4143620000
H	-3.8451130000	4.8639480000	-0.1017810000
C	-4.8521870000	6.4107110000	2.7834240000
H	-4.4977220000	5.6167780000	4.7643920000
H	-5.0361640000	6.8822490000	0.6796810000
C	-4.8523170000	-2.8341890000	1.9712320000
C	-5.1212140000	-2.9373700000	3.3482620000
C	-5.6820740000	-3.5483200000	1.0891960000
C	-6.1727000000	-3.7265830000	3.8199830000
H	-4.4912920000	-2.3952680000	4.0575560000
C	-6.7354650000	-4.3343580000	1.5650630000
H	-5.5064470000	-3.4782340000	0.0127550000
C	-7.0004110000	-4.4434540000	2.9395330000
H	-6.3529580000	-3.7878110000	4.8975190000
H	-7.3677280000	-4.8701790000	0.8508710000
C	1.0471650000	-4.0507160000	-1.6880740000
C	0.3948040000	-4.8032160000	-2.6804240000
C	2.3306540000	-4.4708230000	-1.2914070000
C	0.9987800000	-5.9285210000	-3.2488570000
H	-0.5966550000	-4.4949820000	-3.0213190000
C	2.9298760000	-5.5971860000	-1.8592520000
H	2.8634210000	-3.9071240000	-0.5219170000
C	2.2760680000	-6.3514130000	-2.8484270000
H	0.4664930000	-6.4857880000	-4.0253640000
H	3.9274290000	-5.8975700000	-1.5248690000
C	5.4835920000	5.3671240000	-3.6073730000
H	5.1897700000	5.7028860000	-4.6133330000
H	5.6533500000	6.2719970000	-2.9975200000
H	6.4502040000	4.8458190000	-3.6855260000
C	-5.5507490000	7.6603440000	3.2617270000
H	-6.3062920000	8.0031210000	2.5382320000
H	-6.0474300000	7.5004800000	4.2308230000
H	-4.8326720000	8.4884380000	3.3970730000
C	-8.1169110000	-5.3172050000	3.4577300000
H	-8.8798320000	-5.4993700000	2.6860140000
H	-7.7333430000	-6.3017990000	3.7785270000
H	-8.6109750000	-4.8652270000	4.3319510000
C	2.9188220000	-7.5825950000	-3.4393980000
H	2.8219610000	-8.4462290000	-2.7582620000
H	2.4512770000	-7.8639640000	-4.3947490000
H	3.9958230000	-7.4316170000	-3.6135390000
O	-0.2244120000	-0.1643630000	1.9333260000
C	1.2459580000	-1.6031320000	3.5367650000
C	1.9033430000	-1.8676510000	4.7602380000
C	1.3985920000	-2.5883430000	2.5421550000
C	2.6534990000	-3.0279780000	4.9819880000
H	1.8258640000	-1.1401780000	5.5744140000
C	2.1456210000	-3.7596480000	2.7449330000

H 0.9082540000 -2.4266570000 1.5814070000
C 2.7806700000 -3.9890020000 3.9694550000
H 3.1425860000 -3.1869170000 5.9493000000
H 2.2283650000 -4.4992210000 1.9412970000
H 3.3644030000 -4.8996410000 4.1351460000
C 1.4075500000 1.0364360000 3.5616300000
C 1.7181350000 1.5476490000 4.8421760000
C 2.1296880000 1.6063600000 2.4923860000
C 2.6777570000 2.5487300000 5.0470720000
H 1.1853450000 1.1587690000 5.7152980000
C 3.0906230000 2.6099470000 2.6758710000
H 1.9155310000 1.2500170000 1.4831040000
C 3.3737910000 3.0901820000 3.9602190000
H 2.8796370000 2.9124200000 6.0605370000
H 3.6193580000 3.0243780000 1.8109710000
H 4.1210210000 3.8752410000 4.1111170000
C -0.8814740000 -0.1854270000 4.4237840000
C -1.4377510000 -1.3566360000 4.9825310000
C -1.4741680000 1.0238670000 4.8511300000
C -2.4960590000 -1.3330690000 5.9029530000
H -1.0223820000 -2.3279430000 4.6966520000
C -2.5281360000 1.0709090000 5.7724210000
H -1.0869220000 1.9686090000 4.4563230000
C -3.0483560000 -0.1133580000 6.3093920000
H -2.8846440000 -2.2715720000 6.3127580000
H -2.9434920000 2.0365420000 6.0806460000
H -3.8672790000 -0.0847580000 7.0344010000
B 0.3406570000 -0.2177990000 3.2786970000
Ti -1.1491140000 0.0000870000 0.2272480000
O -2.0210870000 0.1591600000 -1.2521030000

[(TTP)Ti(OPh)$_2$]
N 1.1359880000 -2.3038070000 0.4949580000
N 1.0516610000 1.8720540000 0.4440080000
N 3.0895790000 -0.1909330000 1.0490040000
N -0.9422950000 -0.2664870000 -0.0452110000
C 2.2062220000 -3.0918410000 0.8541770000
C 2.0921590000 2.6957350000 0.7889810000
C 0.0526340000 -3.1286090000 0.2706550000
C -0.0543310000 2.6491030000 0.2008840000
C 3.9092340000 -1.2843780000 1.2114770000
C 3.8581760000 0.9462250000 1.1895870000
C -1.6791480000 -1.3939220000 -0.3023800000
C -1.7175390000 0.8317060000 -0.3274930000
C 3.5122790000 -2.6411260000 1.1578820000
C 3.4111030000 2.2864230000 1.1079960000
C -1.2450690000 -2.7320200000 -0.1303500000
C -1.3363790000 2.1892100000 -0.1889940000
C 1.7757830000 -4.4713500000 0.8810140000
C 1.6190210000 4.0643650000 0.7831990000
C 0.4563920000 -4.4936640000 0.5237660000

C	0.3013070000	4.0355890000	0.4217470000
C	5.2588300000	-0.8202470000	1.4412450000
C	5.2273150000	0.5463480000	1.4280840000
C	-2.9816450000	-0.9928560000	-0.7917450000
C	-3.0050610000	0.3735830000	-0.8070100000
H	2.3998020000	-5.3188330000	1.1516200000
H	2.2146920000	4.9374280000	1.0357510000
H	-0.1909540000	-5.3633030000	0.4502690000
H	-0.3744620000	4.8811180000	0.3259440000
H	6.1269080000	-1.4583100000	1.5831180000
H	6.0658150000	1.2252960000	1.5570740000
H	-3.7748620000	-1.6696920000	-1.0975680000
H	-3.8211060000	1.0152690000	-1.1280230000
C	-2.2453260000	-3.8159370000	-0.3952880000
C	-3.3760390000	-3.9736680000	0.4227800000
C	-2.0824650000	-4.7058690000	-1.4732570000
C	-4.3075620000	-4.9861970000	0.1736440000
H	-3.5247500000	-3.3012750000	1.2713240000
C	-3.0176380000	-5.7114390000	-1.7205890000
H	-1.2153820000	-4.6013090000	-2.1302230000
C	-4.1490670000	-5.8735670000	-0.9016770000
H	-5.1743690000	-5.0884400000	0.8326130000
H	-2.8672150000	-6.3840960000	-2.5701250000
C	4.5535810000	-3.6791570000	1.4445210000
C	5.1279940000	-3.7970430000	2.7208540000
C	4.9871960000	-4.5669070000	0.4422280000
C	6.0983560000	-4.7684370000	2.9850900000
H	4.8051160000	-3.1262790000	3.5209350000
C	5.9590760000	-5.5315840000	0.7099630000
H	4.5625450000	-4.4924170000	-0.5620220000
C	6.5342480000	-5.6533670000	1.9872210000
H	6.5224690000	-4.8401320000	3.9906140000
H	6.2806430000	-6.2031210000	-0.0915680000
C	4.4154100000	3.3648850000	1.3799100000
C	4.9740090000	3.5302610000	2.6579310000
C	4.8263630000	4.2456420000	0.3621260000
C	5.9074080000	4.5406670000	2.9087380000
H	4.6683530000	2.8653620000	3.4695830000
C	5.7620260000	5.2489950000	0.6163610000
H	4.4126690000	4.1352760000	-0.6433490000
C	6.3210950000	5.4185720000	1.8953340000
H	6.3199310000	4.6487100000	3.9158180000
H	6.0671860000	5.9136920000	-0.1971700000
C	-2.3778290000	3.2265950000	-0.4781100000
C	-2.2475890000	4.0982920000	-1.5752390000
C	-3.5139690000	3.3591300000	0.3369740000
C	-3.2202530000	5.0617840000	-1.8444120000
H	-1.3770440000	4.0112300000	-2.2300900000
C	-4.4828560000	4.3300340000	0.0657920000
H	-3.6369490000	2.7005750000	1.2003260000
C	-4.3574040000	5.1989340000	-1.0288140000

H -3.0952110000 5.7204970000 -2.7088580000
H -5.3533210000 4.4138920000 0.7225430000
C -5.1524230000 -6.9658220000 -1.1796190000
H -5.6064590000 -6.8447810000 -2.1773680000
H -4.6744630000 -7.9594120000 -1.1661490000
H -5.9634670000 -6.9691070000 -0.4370340000
C 7.5830880000 -6.7022350000 2.2642930000
H 8.4623130000 -6.5684000000 1.6122500000
H 7.9266860000 -6.6651080000 3.3082210000
H 7.1944940000 -7.7158250000 2.0701600000
C 7.3292610000 6.5101180000 2.1582060000
H 8.2123120000 6.4023870000 1.5066140000
H 6.9016850000 7.5056260000 1.9524130000
H 7.6746810000 6.4991580000 3.2021370000
C -5.4002110000 6.2474390000 -1.3291550000
H -4.9642050000 7.2601900000 -1.3101440000
H -5.8302210000 6.1051670000 -2.3345870000
H -6.2238960000 6.2194190000 -0.6011470000
O 0.5245840000 -0.1213870000 2.2507580000
C -0.2585540000 0.3303600000 3.2352890000
C 0.1227400000 1.4605940000 3.9872320000
C -1.4639360000 -0.3308210000 3.5486090000
C -0.6911060000 1.9168540000 5.0263860000
H 1.0594390000 1.9657560000 3.7422760000
C -2.2677740000 0.1363980000 4.5909060000
H -1.7510700000 -1.2086290000 2.9660930000
C -1.8893220000 1.2608180000 5.3351410000
H -0.3846570000 2.7944400000 5.6023440000
H -3.1999240000 -0.3852820000 4.8249150000
H -2.5213970000 1.6211130000 6.1502910000
Ti 1.0859850000 -0.2241040000 0.4864900000
O 1.4877040000 -0.1532920000 -1.3228530000
C 1.3822330000 0.3615560000 -2.5518980000
C 2.2136810000 1.4273260000 -2.9531400000
C 0.4534880000 -0.1707360000 -3.4696540000
C 2.1103940000 1.9470420000 -4.2454890000
H 2.9346420000 1.8314180000 -2.2394780000
C 0.3608910000 0.3590850000 -4.7584340000
H -0.1846490000 -0.9989230000 -3.1542310000
C 1.1856040000 1.4192850000 -5.1551910000
H 2.7612460000 2.7730750000 -4.5452980000
H -0.3632720000 -0.0622580000 -5.4611850000
H 1.1099910000 1.8288720000 -6.1653030000

{(TTP)TiO[B(Ph)$_3$](DMA)}
N -1.1368350000 2.2442530000 -1.4037000000
N -2.4336290000 -0.3618280000 -1.1790460000
N 1.2825620000 1.1626850000 -0.1716560000
N -0.0445800000 -1.4210500000 0.1279920000
C -0.3527190000 3.3787750000 -1.4372440000
C -1.0850430000 4.4372100000 -2.0925290000

C	-2.3095010000	3.9350930000	-2.4289620000
C	-2.3460820000	2.5611950000	-1.9853530000
C	2.2790780000	0.4897240000	0.4974270000
C	3.3370870000	1.4178920000	0.8240050000
C	2.9662310000	2.6400810000	0.3397060000
C	1.6770740000	2.4757540000	-0.2917800000
C	-3.4840220000	0.3525620000	-1.7143180000
C	-4.6649860000	-0.4782790000	-1.7183040000
C	-4.3149610000	-1.6820780000	-1.1758510000
C	-2.9126960000	-1.6091900000	-0.8393730000
C	-0.8387580000	-2.5457960000	0.1929130000
C	-0.0708470000	-3.6318700000	0.7553020000
C	1.1741600000	-3.1455570000	1.0362360000
C	1.1839000000	-1.7506310000	0.6617830000
C	-3.4560690000	1.7019270000	-2.1293050000
C	0.9492910000	3.5145300000	-0.9110070000
C	2.2748460000	-0.8762510000	0.8534120000
C	-2.1836860000	-2.6480560000	-0.2220060000
C	-2.8944330000	-3.9422820000	0.0321220000
C	3.5070330000	-1.4251210000	1.5054330000
C	1.6033520000	4.8596580000	-1.0025390000
C	-4.7034540000	2.2643730000	-2.7394080000
C	4.7077340000	-1.5458760000	0.7869080000
C	5.8583230000	-2.0569580000	1.3953730000
C	5.8553070000	-2.4598420000	2.7394430000
C	4.6527320000	-2.3319220000	3.4567640000
C	3.4998680000	-1.8276990000	2.8536570000
C	-3.1704970000	-4.3666470000	1.3441570000
C	-3.8428370000	-5.5668040000	1.5836810000
C	-4.2595580000	-6.3928000000	0.5262140000
C	-3.9833080000	-5.9653110000	-0.7825120000
C	-3.3149610000	-4.7630590000	-1.0283390000
C	2.7157830000	5.0659320000	-1.8343060000
C	3.3264030000	6.3210360000	-1.9177390000
C	2.8538040000	7.4122480000	-1.1728640000
C	1.7425060000	7.2008270000	-0.3378230000
C	1.1262410000	5.9516260000	-0.2546150000
C	-5.4483300000	3.2604840000	-2.0827250000
C	-6.6155140000	3.7749660000	-2.6504830000
C	-7.0824470000	3.3218590000	-3.8963080000
C	-6.3383790000	2.3253510000	-4.5478170000
C	-5.1720220000	1.8033230000	-3.9816040000
C	-8.3270160000	3.9051770000	-4.5188150000
C	3.5032690000	8.7712590000	-1.2637750000
C	7.0920210000	-3.0181300000	3.3996520000
C	-4.9558910000	-7.7049160000	0.7911620000
H	-0.7074670000	5.4388640000	-2.2784270000
H	-3.1165450000	4.4503360000	-2.9421470000
H	4.2417200000	1.1697130000	1.3722450000
H	3.5114290000	3.5766960000	0.4179540000
H	-5.6469550000	-0.1705920000	-2.0667440000

H	-4.9579880000	-2.5402020000	-1.0015140000
H	-0.4346820000	-4.6443640000	0.9064210000
H	2.0145370000	-3.6869820000	1.4616620000
H	4.7404170000	-1.2427270000	-0.2624930000
H	6.7774830000	-2.1449550000	0.8092920000
H	4.6189700000	-2.6299790000	4.5087990000
H	2.5813120000	-1.7342630000	3.4384190000
H	-2.8652180000	-3.7418760000	2.1873130000
H	-4.0522220000	-5.8652950000	2.6149770000
H	-4.2954540000	-6.5848070000	-1.6281890000
H	-3.1066190000	-4.4616080000	-2.0573310000
H	3.1043140000	4.2360610000	-2.4297620000
H	4.1879580000	6.4528500000	-2.5783160000
H	1.3537740000	8.0296380000	0.2611140000
H	0.2670490000	5.8160970000	0.4071350000
H	-5.1153550000	3.6273160000	-1.1086490000
H	-7.1786210000	4.5420460000	-2.1110020000
H	-6.6759200000	1.9482780000	-5.5173750000
H	-4.6153920000	1.0281680000	-4.5138820000
H	-8.7581710000	3.2288800000	-5.2717970000
H	-9.0966570000	4.1150360000	-3.7599670000
H	-8.1040530000	4.8610400000	-5.0244780000
H	2.8172460000	9.5099960000	-1.7118190000
H	3.7739840000	9.1527250000	-0.2656830000
H	4.4150390000	8.7438550000	-1.8779420000
H	7.9641960000	-2.9703430000	2.7315650000
H	7.3367760000	-2.4659920000	4.3218150000
H	6.9462330000	-4.0726180000	3.6891030000
H	-4.2226960000	-8.5115790000	0.9672170000
H	-5.5949870000	-7.6492400000	1.6857130000
H	-5.5793320000	-8.0114890000	-0.0620320000
C	-1.9870610000	1.2369640000	2.1493750000
O	-1.2197430000	0.9707410000	1.2081370000
C	-3.4777710000	0.9945430000	2.0201070000
H	-4.0495770000	1.9261600000	2.1475060000
H	-3.6767280000	0.5897770000	1.0232630000
H	-3.8338410000	0.2760270000	2.7739630000
N	-1.5112190000	1.7488100000	3.3110370000
C	-2.3308190000	2.0830000000	4.4666180000
H	-1.9985960000	1.5075010000	5.3469920000
H	-2.2333130000	3.1553570000	4.7060870000
H	-3.3874360000	1.8633790000	4.2909970000
C	-0.0864180000	2.0026150000	3.4787860000
H	0.0855410000	3.0683020000	3.7040930000
H	0.3106950000	1.4070770000	4.3178520000
H	0.4449680000	1.7353350000	2.5602770000
Ti	-0.4609450000	0.3144860000	-0.9634080000
O	0.1519130000	-0.1368280000	-2.4759560000
B	0.7104290000	-0.5737070000	-3.8688960000
C	2.3335400000	-0.7172570000	-3.7209690000
C	0.0042010000	-2.0074370000	-4.2233990000

C 0.3019030000 0.6028140000 -4.9300170000
C 3.0573020000 -1.7793450000 -4.2991410000
C 3.0960860000 0.2718800000 -3.0647550000
C -0.6512130000 -2.2671900000 -5.4427390000
C 0.0830970000 -3.0849130000 -3.3151040000
C 1.2128920000 1.1536890000 -5.8527840000
C -1.0224120000 1.0846550000 -4.9986650000
C 4.4553480000 -1.8562610000 -4.2302620000
H 2.5159790000 -2.5724480000 -4.8214860000
C 4.4916790000 0.2057490000 -2.9810740000
H 2.5832770000 1.1199660000 -2.6056260000
C -1.1956110000 -3.5237900000 -5.7451140000
H -0.7383590000 -1.4680530000 -6.1831610000
C -0.4572530000 -4.3430590000 -3.6003440000
H 0.5901180000 -2.9368450000 -2.3577020000
C 0.8317920000 2.1248310000 -6.7896770000
H 2.2524400000 0.8159200000 -5.8435230000
C -1.4170560000 2.0583310000 -5.9224390000
H -1.7693490000 0.6836510000 -4.3088700000
C 5.1818160000 -0.8631320000 -3.5664610000
H 4.9782340000 -2.6973370000 -4.6959700000
H 5.0439860000 0.9928830000 -2.4588690000
C -1.1022490000 -4.5700680000 -4.8231690000
H -1.6937160000 -3.6846620000 -6.7062200000
H -0.3743030000 -5.1515750000 -2.8679850000
C -0.4874760000 2.5852210000 -6.8277000000
H 1.5708410000 2.5245130000 -7.4909770000
H -2.4540620000 2.4071020000 -5.9376070000
H 6.2724810000 -0.9187240000 -3.5069030000
H -1.5240000000 -5.5525510000 -5.0535470000
H -0.7898660000 3.3451440000 -7.5538680000

[(DMA)(TTP)Ti^{IV}(OPh)]$^{+}$
N -0.0485300000 2.0840160000 -0.4125740000
N -2.1006760000 -0.0057650000 -0.4729150000
N 2.0471980000 0.0444500000 -0.4687360000
N -0.0068110000 -2.0446630000 -0.4012540000
C 1.0463790000 2.9110410000 -0.5631470000
C 0.5869540000 4.2760790000 -0.6484280000
C -0.7738700000 4.2580930000 -0.5156940000
C -1.1753560000 2.8800060000 -0.3708460000
C 2.8675860000 -1.0489450000 -0.2846290000
C 4.2315210000 -0.5909580000 -0.1694460000
C 4.2184620000 0.7691090000 -0.3099450000
C 2.8437530000 1.1697820000 -0.4905980000
C -2.9234730000 1.0890140000 -0.3079320000
C -4.2892370000 0.6320950000 -0.2176940000
C -4.2751480000 -0.7281130000 -0.3606530000
C -2.8981960000 -1.1300260000 -0.5168340000
C -1.1003320000 -2.8722530000 -0.5649570000
C -0.6395170000 -4.2368820000 -0.6461050000

C	0.7200270000	-4.2183870000	-0.5006010000
C	1.1200080000	-2.8404080000	-0.3515920000
C	-2.5141420000	2.4406410000	-0.2675430000
C	2.4005940000	2.5079980000	-0.5926200000
C	2.4583760000	-2.4002810000	-0.2417460000
C	-2.4536530000	-2.4685870000	-0.6136720000
C	-3.4989720000	-3.5326040000	-0.7425760000
C	3.5191350000	-3.4479780000	-0.1034890000
C	3.4472530000	3.5731490000	-0.6991280000
C	-3.5758530000	3.4891200000	-0.1409790000
C	4.4715160000	-3.6589570000	-1.1143510000
C	5.4541990000	-4.6436120000	-0.9787610000
C	5.5271510000	-5.4477480000	0.1692600000
C	4.5714710000	-5.2345160000	1.1780670000
C	3.5845000000	-4.2570850000	1.0458210000
C	-3.7108780000	-4.4770350000	0.2772770000
C	-4.6947750000	-5.4595730000	0.1497520000
C	-5.4986190000	-5.5426600000	-0.9995660000
C	-5.2861090000	-4.5953980000	-2.0145270000
C	-4.3075600000	-3.6065810000	-1.8901920000
C	4.2772850000	3.6521560000	-1.8295720000
C	5.2569390000	4.6441290000	-1.9318700000
C	5.4487270000	5.5868780000	-0.9103170000
C	4.6204680000	5.5003080000	0.2226970000
C	3.6370580000	4.5164020000	0.3278470000
C	-3.6547230000	4.2956420000	1.0092780000
C	-4.6422510000	5.2737990000	1.1313110000
C	-5.5849880000	5.4902590000	0.1110400000
C	-5.4985800000	4.6885450000	-1.0376590000
C	-4.5151560000	3.7031750000	-1.1632020000
C	-6.6567170000	6.5416740000	0.2598960000
C	6.4994720000	6.6642450000	-1.0169240000
C	6.5990800000	-6.4972480000	0.3295740000
C	-6.5363490000	-6.6276470000	-1.1475500000
H	1.2232210000	5.1441850000	-0.7963610000
H	-1.4504840000	5.1079190000	-0.5388070000
H	5.0945890000	-1.2275250000	0.0043960000
H	5.0677880000	1.4454310000	-0.2674770000
H	-5.1549890000	1.2693340000	-0.0605200000
H	-5.1261440000	-1.4032260000	-0.3363690000
H	-1.2740090000	-5.1051210000	-0.8007030000
H	1.3970840000	-5.0679600000	-0.5190280000
H	4.4360420000	-3.0547030000	-2.0241250000
H	6.1766630000	-4.7914490000	-1.7862720000
H	4.6008920000	-5.8445990000	2.0855110000
H	2.8570030000	-4.1121440000	1.8483800000
H	-3.1078550000	-4.4327450000	1.1874680000
H	-4.8442100000	-6.1742340000	0.9641000000
H	-5.8972740000	-4.6306980000	-2.9208010000
H	-4.1638530000	-2.8844790000	-2.6977960000
H	4.1500530000	2.9339520000	-2.6433490000

H	5.8838890000	4.6849220000	-2.8269180000
H	4.7507480000	6.2148280000	1.0406420000
H	3.0143920000	4.4694530000	1.2246020000
H	-2.9374360000	4.1480540000	1.8205150000
H	-4.6824600000	5.8818620000	2.0397050000
H	-6.2109710000	4.8386890000	-1.8536420000
H	-4.4691540000	3.1006610000	-2.0736710000
H	-7.2013350000	6.7006650000	-0.6821200000
H	-7.3933690000	6.2494000000	1.0277440000
H	-6.2302420000	7.5065060000	0.5782380000
H	6.0374760000	7.6644700000	-1.0727840000
H	7.1593320000	6.6673030000	-0.1340680000
H	7.1253650000	6.5324680000	-1.9113510000
H	7.1424560000	-6.6678030000	-0.6111630000
H	7.3371920000	-6.1948300000	1.0921880000
H	6.1734780000	-7.4579430000	0.6609900000
H	-6.0913380000	-7.5407760000	-1.5804350000
H	-6.9683960000	-6.9083590000	-0.1749870000
H	-7.3543730000	-6.3166200000	-1.8144740000
C	-0.3179740000	-0.1591740000	2.6666630000
O	0.0133080000	0.0409290000	1.4681440000
C	-1.7141170000	-0.6256240000	2.9978650000
H	-2.2136530000	0.0706630000	3.6871600000
H	-2.2957280000	-0.6907160000	2.0741630000
H	-1.6927370000	-1.6177470000	3.4736850000
N	0.5681230000	0.0457860000	3.6486070000
C	0.2810510000	-0.1552680000	5.0656500000
H	1.0424890000	-0.8207590000	5.5007460000
H	0.3192380000	0.8087640000	5.5981950000
H	-0.7026410000	-0.6057500000	5.2202580000
C	1.9235010000	0.5097440000	3.3616860000
H	2.1261000000	1.4281170000	3.9340690000
H	2.6532460000	-0.2576840000	3.6648660000
H	2.0322150000	0.7125900000	2.2929350000
Ti	-0.0267690000	0.0187860000	-0.5895310000
O	-0.0552020000	0.0115590000	-2.3645970000
C	-0.2333340000	-0.0173440000	-3.6973240000
C	0.2769060000	1.0267750000	-4.4883340000
C	-0.9234010000	-1.0895020000	-4.2894850000
C	0.0909100000	0.9918910000	-5.8714130000
H	0.8104390000	1.8488610000	-4.0073960000
C	-1.0992450000	-1.1086630000	-5.6744820000
H	-1.3108730000	-1.8907810000	-3.6576720000
C	-0.5954250000	-0.0721660000	-6.4697500000
H	0.4866450000	1.8035060000	-6.4870500000
H	-1.6354120000	-1.9417700000	-6.1357720000
H	-0.7368760000	-0.0935580000	-7.5526990000

$B(OPh)_3$

O	1.27196500	-4.30345900	5.21353000
O	-0.14881900	-5.68879900	3.92230400

O	-0.95307000	-3.62876800	4.76790400
B	0.05670000	-4.54026100	4.63434900
C	1.56964500	-3.16943500	5.95213800
C	1.36440500	-3.17276600	7.33500700
C	2.13550300	-2.06113500	5.31504200
C	1.72216400	-2.04726900	8.08402600
H	0.93045400	-4.05405400	7.81230000
C	2.48970400	-0.94091100	6.07359800
H	2.29595400	-2.08551600	4.23502000
C	2.28407000	-0.92932300	7.45760800
H	1.56105400	-2.04737500	9.16494300
H	2.93078600	-0.07297800	5.57712400
H	2.56329900	-0.05261400	8.04667400
C	0.81501400	-6.66651300	3.73460000
C	0.87663700	-7.74993000	4.61594100
C	1.66745100	-6.59366800	2.62890500
C	1.81062600	-8.76589600	4.38951400
H	0.19338300	-7.79028800	5.46700000
C	2.59795100	-7.61491100	2.41206300
H	1.59388600	-5.74296100	1.94795000
C	2.67347200	-8.70198700	3.28983200
H	1.86121100	-9.61300200	5.07813200
H	3.26624300	-7.55902900	1.54916000
H	3.40088800	-9.49834800	3.11591100
C	-2.21524900	-3.78487800	4.21779500
C	-2.47708000	-3.28698700	2.93795900
C	-3.22891600	-4.37695200	4.97693400
C	-3.76829800	-3.39391000	2.41157300
H	-1.67171000	-2.81906800	2.36767400
C	-4.51663000	-4.47861300	4.44098000
H	-3.00289600	-4.74940600	5.97840700
C	-4.79041800	-3.98945800	3.15891100
H	-3.97465400	-3.00664400	1.41067900
H	-5.31011400	-4.94239600	5.03227900
H	-5.79804900	-4.06975200	2.74441100

$\{[B(OPh)_3]_2O\}^{2-}$

O	-2.04448300	4.68698600	0.76442900
O	-4.13023100	3.55156400	0.12769500
O	-2.11256900	3.08654400	-1.03100500
O	-3.20719700	5.28997600	-1.21653400
O	-2.06655500	6.96619300	1.67517400
O	-3.44044600	5.31797400	2.70178200
O	-1.08460600	5.02444200	2.84671900
B	-2.83625300	4.17505000	-0.28263300
B	-2.17869700	5.48901200	1.91990300
C	-4.62400800	5.92453000	2.55540800
C	-5.03349700	6.61131700	1.39097200
C	-5.53211500	5.85037800	3.63665600
C	-6.29800400	7.20413300	1.32934200
H	-4.35737100	6.65600100	0.53886600

C	-6.79338800	6.44487700	3.56125600
H	-5.22122800	5.31578600	4.53823600
C	-7.18950700	7.13231400	2.40732500
H	-6.59288000	7.72869500	0.41501900
H	-7.47317500	6.37132200	4.41569600
H	-8.17578800	7.59964300	2.34757300
C	-0.79069900	5.50497400	4.06149900
C	-1.42626700	6.61106200	4.67109900
C	0.23965900	4.85556800	4.78198300
C	-1.03668000	7.03628100	5.94552300
H	-2.21846700	7.13032700	4.13553000
C	0.61842700	5.29038500	6.05221800
H	0.73362800	3.99904900	4.31566200
C	-0.01652600	6.38723400	6.64992100
H	-1.54457700	7.89560000	6.39459400
H	1.41908900	4.76549200	6.58217200
H	0.27907400	6.72861200	7.64517600
C	-1.03034400	7.62313900	1.14389700
C	-1.18238700	9.01208000	0.92137300
C	0.20544100	7.02834400	0.80420800
C	-0.14366900	9.77305600	0.38422900
H	-2.13718700	9.47572300	1.18245400
C	1.23847800	7.80337500	0.26674500
H	0.33990500	5.96052500	0.96599000
C	1.07993100	9.17711800	0.05056100
H	-0.29172500	10.84536300	0.22410700
H	2.18649300	7.31893800	0.01287900
H	1.89380400	9.77306800	-0.36997400
C	-4.28774500	2.48141000	0.91375900
C	-3.22993800	1.74683700	1.49744800
C	-5.61103600	2.05296200	1.17247200
C	-3.49952600	0.63415100	2.30031400
H	-2.20350500	2.05949900	1.31455100
C	-5.86591700	0.94016300	1.97506800
H	-6.43331800	2.61714200	0.72492300
C	-4.81235400	0.21661500	2.54879200
H	-2.66200700	0.08359000	2.74014300
H	-6.90125800	0.63453900	2.15446500
H	-5.01118600	-0.65500300	3.17745800
C	-3.88978100	5.20604500	-2.36583400
C	-4.36973000	4.00506900	-2.93630100
C	-4.14040400	6.41419400	-3.05855700
C	-5.06910200	4.02790700	-4.14736900
H	-4.18733800	3.06351800	-2.42173400
C	-4.83951800	6.42243400	-4.26588100
H	-3.76877100	7.34527700	-2.62264600
C	-5.31271900	5.22796900	-4.82491300
H	-5.43015800	3.08401900	-4.56767600
H	-5.01692900	7.37383300	-4.77652200
H	-5.86056200	5.23339200	-5.77057100
C	-0.95713300	3.19787100	-1.69607700

C	-0.22759400	4.39950600	-1.83496500
C	-0.43922400	2.03206000	-2.30793300
C	0.96836400	4.42034700	-2.55948000
H	-0.60323400	5.30396100	-1.36060600
C	0.75542100	2.06718400	-3.02820900
H	-0.99973000	1.09943200	-2.20289400
C	1.47341700	3.26291500	-3.16322100
H	1.51498700	5.36436100	-2.64936600
H	1.13023800	1.14871700	-3.49016800
H	2.40919300	3.29057000	-3.72717800

DMA

C	0.72618500	-0.28948800	0.00028400
O	1.07577800	-1.47688800	-0.00047500
C	1.77547900	0.81123300	0.00006200
H	1.69197100	1.45463200	0.88952300
H	2.76402800	0.33710100	-0.00116700
H	1.69025400	1.45593800	-0.88827900
N	-0.58777100	0.07540000	0.00159800
C	-1.07185900	1.44631200	-0.00074800
H	-1.68576100	1.63722600	-0.89811300
H	-1.70434700	1.63213700	0.88451800
H	-0.24880100	2.16657800	0.01023900
C	-1.62987500	-0.93960100	-0.00056300
H	-2.27339700	-0.83298500	0.88954500
H	-2.26740200	-0.83600300	-0.89542300
H	-1.17133500	-1.93379600	0.00251300

$[(TTP)Ti(DMA)]^+$

N	-0.01929300	2.04089100	-0.39547400
N	-2.03478700	-0.02001900	-0.41203000
N	2.03609000	0.02089600	-0.41995300
N	0.02161600	-2.03974100	-0.39499200
C	1.07903800	2.87425500	-0.50293300
C	0.64117000	4.23447200	-0.69072000
C	-0.72478200	4.22062000	-0.68847500
C	-1.13456600	2.85170200	-0.49996200
C	2.87701000	-1.07791000	-0.41090800
C	4.24956600	-0.63953900	-0.35481500
C	4.23589000	0.72586000	-0.35719900
C	2.85480800	1.13645400	-0.41444300
C	-2.87650800	1.07998500	-0.41009800
C	-4.24832100	0.64086200	-0.35945400
C	-4.23474200	-0.72512600	-0.36179000
C	-2.85440700	-1.13664300	-0.41348000
C	-1.07699700	-2.87277100	-0.50260700
C	-0.63934000	-4.23320100	-0.68982400
C	0.72661200	-4.21981300	-0.68762500
C	1.13666100	-2.85107000	-0.49912900
Ti	-0.00176300	0.00058200	0.14192100

C	-2.47786900	2.42905400	-0.46295700
C	2.43015500	2.47789200	-0.46842500
C	2.47945600	-2.42773000	-0.46211500
C	-2.42861100	-2.47729100	-0.46854400
C	-3.47359000	-3.54957900	-0.51712100
C	3.54620000	-3.47845500	-0.50503000
C	3.47553200	3.54967300	-0.51573300
C	-3.54408100	3.48045200	-0.50684200
C	4.38725300	-3.61433400	-1.62136200
C	5.37622200	-4.60187800	-1.65839000
C	5.56576400	-5.48411700	-0.58376300
C	4.72231600	-5.34460700	0.53245200
C	3.73003100	-4.36441100	0.57237200
C	-3.64589100	-4.43810600	0.55977800
C	-4.61995800	-5.43656300	0.51597000
C	-5.45449600	-5.59307900	-0.60441800
C	-5.27838500	-4.70590100	-1.67756700
C	-4.30798500	-3.70052400	-1.63669600
C	4.31308400	3.69827900	-1.63323700
C	5.28332800	4.70389400	-1.67371500
C	5.45636300	5.59334800	-0.60205200
C	4.61854800	5.43929000	0.51632500
C	3.64458600	4.44082100	0.55964000
C	-3.73014400	4.36400200	0.57220200
C	-4.72266300	5.34390100	0.53268700
C	-5.56372000	5.48600600	-0.58505200
C	-5.37288600	4.60530400	-1.66063800
C	-4.38347800	3.61807200	-1.62409000
C	-6.62898200	6.55411200	-0.61792200
C	6.49351200	6.68840500	-0.64545200
C	6.63847700	-6.54484400	-0.61263400
C	-6.49011300	-6.68950800	-0.64988900
H	1.29453800	5.09165900	-0.82835400
H	-1.39587000	5.06442200	-0.82356500
H	5.11693700	-1.29251900	-0.31346200
H	5.09002800	1.39620900	-0.31813600
H	-5.11623900	1.29339500	-0.32267700
H	-5.08949900	-1.39495000	-0.32730700
H	-1.29301200	-5.09024900	-0.82688300
H	1.39741400	-5.06380500	-0.82296800
H	4.26049000	-2.94606200	-2.47669300
H	6.01119200	-4.68880500	-2.54451500
H	4.84556200	-6.01564700	1.38756100
H	3.09095300	-4.27850500	1.45480800
H	-3.01423600	-4.33849900	1.44611800
H	-4.73664700	-6.10738900	1.37212700
H	-5.91059100	-4.80208900	-2.56473600
H	-4.19231400	-3.02783000	-2.49013300
H	4.19971800	3.02393200	-2.48567300
H	5.91768800	4.79853600	-2.55950500
H	4.73251700	6.11227700	1.37116300

H	3.01028000	4.34320600	1.44429700
H	-3.09326100	4.27572800	1.45600200
H	-4.84850600	6.01223700	1.38954000
H	-6.00764600	4.69263900	-2.54687000
H	-4.25610800	2.95061300	-2.47996500
H	-7.21929100	6.50862100	-1.54452800
H	-7.32218400	6.45211200	0.23336100
H	-6.18497400	7.56124100	-0.54781000
H	6.01832300	7.68117600	-0.72458700
H	7.10327900	6.69920900	0.27230200
H	7.16923500	6.57200400	-1.50522200
H	7.15235100	-6.57438900	-1.58435700
H	7.39843700	-6.36210000	0.16578000
H	6.21732800	-7.54470500	-0.41767100
H	-6.01431100	-7.67880400	-0.76327000
H	-7.07916900	-6.72361600	0.28054100
H	-7.18462400	-6.55557600	-1.49201600
C	-0.44747000	-0.03220400	3.25942100
O	0.01662800	0.00281300	2.06762500
C	-1.93368700	-0.14421700	3.45629200
H	-2.32177300	0.71987400	4.01577000
H	-2.42060100	-0.18128800	2.47620600
H	-2.18790200	-1.05614800	4.01663900
N	0.38724700	0.03039200	4.28385500
C	-0.04279700	-0.00473700	5.68196800
H	0.39387800	-0.88649400	6.17473300
H	0.31996500	0.89895800	6.19375600
H	-1.13097900	-0.04964400	5.76881000
C	1.83604700	0.13700900	4.09434500
H	2.19398000	1.06946400	4.55598800
H	2.32805600	-0.71326200	4.58962000
H	2.08247700	0.13549000	3.02947700

[(TTP)Ti(DMA)]

N	-0.01713000	2.09144600	-0.37364300
N	-2.04008000	-0.02203100	-0.41326700
N	2.05427700	0.02070900	-0.43868000
N	0.02523900	-2.09227800	-0.37216400
C	1.08686400	2.91646100	-0.47498200
C	0.64860800	4.27345800	-0.63829200
C	-0.72839800	4.26003400	-0.63249000
C	-1.13777900	2.89483900	-0.46530700
C	2.89298700	-1.09325100	-0.43397600
C	4.26734000	-0.63810900	-0.39313000
C	4.25332500	0.72503300	-0.39477600
C	2.86982100	1.15168200	-0.43641000
C	-2.88126800	1.09331100	-0.41296400
C	-4.25436700	0.63716900	-0.36940200
C	-4.24051900	-0.72623300	-0.37185100
C	-2.85835400	-1.15411000	-0.41632300
C	-1.07804200	-2.91889800	-0.46690500

C	-0.63987600	-4.27528900	-0.63322300
C	0.73710000	-4.26000000	-0.63533600
C	1.14659000	-2.89427200	-0.47117500
Ti	0.00813200	-0.00024700	-0.14408400
C	-2.48631400	2.43739300	-0.44010500
C	2.44551500	2.48752200	-0.45978200
C	2.49605100	-2.43744600	-0.45486700
C	-2.43593100	-2.48975900	-0.44474300
C	-3.48657100	-3.55878600	-0.48105700
C	3.56779900	-3.48525300	-0.49018000
C	3.49503400	3.55744900	-0.49857300
C	-3.55927900	3.48424300	-0.47181400
C	4.39934200	-3.64436500	-1.61092300
C	5.39503200	-4.62663000	-1.63892900
C	5.59964200	-5.48757300	-0.55038300
C	4.76483500	-5.33005600	0.57018500
C	3.76927800	-4.35244700	0.60012500
C	-3.67099800	-4.43009500	0.60897200
C	-4.64495500	-5.42912400	0.57724700
C	-5.47398900	-5.60476400	-0.54494900
C	-5.28722200	-4.73862800	-1.63253000
C	-4.31313900	-3.73489300	-1.60278600
C	4.32120300	3.73211400	-1.62091600
C	5.29456200	4.73647700	-1.65302800
C	5.48124600	5.60482300	-0.56720400
C	4.65266100	5.43080900	0.55559000
C	3.67942500	4.43118300	0.58962000
C	-3.76000600	4.34839600	0.62099500
C	-4.75654800	5.32507500	0.59479100
C	-5.59267300	5.48480100	-0.52450800
C	-5.38909500	4.62642600	-1.61522600
C	-4.39237800	3.64503100	-1.59100700
C	-6.66809700	6.54386400	-0.54088100
C	6.53141800	6.68876800	-0.59212800
C	6.67785800	-6.54375400	-0.56890400
C	-6.52417700	-6.68873900	-0.56777800
H	1.29901600	5.13566000	-0.76226000
H	-1.39686600	5.10916600	-0.75066200
H	5.13913700	-1.28649500	-0.36122600
H	5.11156300	1.39136400	-0.36455200
H	-5.12638700	1.28526800	-0.33842500
H	-5.09913100	-1.39211900	-0.34319400
H	-1.29016100	-5.13813000	-0.75339300
H	1.40568700	-5.10841000	-0.75782100
H	4.26121100	-2.99311900	-2.47782200
H	6.02272600	-4.72678200	-2.52923300
H	4.89898100	-5.98426500	1.43702500
H	3.13727600	-4.25312700	1.48640900
H	-3.04273600	-4.31716100	1.49630200
H	-4.76656400	-6.08604500	1.44388500
H	-5.91201200	-4.85146900	-2.52334500

H	-4.18844300	-3.07993600	-2.46893000
H	4.19652400	3.07559200	-2.48587400
H	5.91878600	4.84811600	-2.54440100
H	4.77414000	6.08946600	1.42093500
H	3.05169500	4.31950600	1.47747000
H	-3.12692100	4.24724400	1.50631500
H	-4.89056000	5.97670500	1.46359000
H	-6.01865800	4.72761800	-2.50407700
H	-4.25533100	2.99542500	-2.45931000
H	-7.21277100	6.55244400	-1.49631100
H	-7.40248800	6.37986300	0.26560800
H	-6.24266400	7.54900600	-0.38345500
H	6.08349100	7.68408000	-0.43467700
H	7.27396400	6.54402600	0.21060000
H	7.07047900	6.70763200	-1.55059400
H	7.18333400	-6.58860400	-1.54467900
H	7.44487300	-6.34409200	0.19860300
H	6.26463200	-7.54274900	-0.35271100
H	-6.07466500	-7.68481900	-0.41967100
H	-7.26031900	-6.54893400	0.24162000
H	-7.07056000	-6.70243400	-1.52216900
C	-0.41098600	-0.01029900	3.03818000
O	0.11227100	0.00719100	1.89443700
C	-1.91226600	-0.06421700	3.18226400
H	-2.29069300	0.80562400	3.73978100
H	-2.35880700	-0.06845700	2.18275300
H	-2.22669400	-0.97274100	3.71793100
N	0.36913300	0.01913100	4.13112800
C	-0.13620500	-0.00086500	5.49851900
H	0.27961300	-0.86760200	6.03709700
H	0.17550200	0.91436600	6.02750900
H	-1.22706200	-0.06541500	5.52583900
C	1.82357700	0.07520800	4.01815800
H	2.20278600	0.97251900	4.53278300
H	2.27077900	-0.81222300	4.49429800
H	2.11656000	0.10880600	2.96480100

[(TTP)Ti(DMA)$_2$]

N	-2.11188400	0.05070700	0.01991700
N	-0.06755300	-2.09595900	-0.02530100
N	0.04092300	2.08831900	-0.02336400
N	2.08803300	-0.05841100	0.03192000
C	-2.88562000	1.18460200	-0.03951100
C	-4.27392600	0.79114500	-0.16096500
C	-4.30969500	-0.57742700	-0.15503000
C	-2.94363200	-1.04178900	-0.03083400
C	1.17546400	2.86215600	0.04503800
C	0.78179400	4.24804700	0.18414900
C	-0.58679700	4.28376800	0.18140500
C	-1.05163800	2.92010900	0.04249000
C	-1.20158300	-2.86987300	0.04756300

C	-0.80741100	-4.25582300	0.18367900
C	0.56119800	-4.29133400	0.17702400
C	1.02548300	-2.92765000	0.03606400
C	2.85969500	-1.19226800	-0.05272900
C	4.24479900	-0.79838400	-0.20433900
C	4.28047700	0.57021600	-0.19809200
C	2.91757400	1.03435200	-0.04341400
Ti	-0.01145000	-0.00382300	0.00610100
C	-2.54196000	-2.40288600	0.01225200
C	-2.41418900	2.52322100	-0.00011800
C	2.51583300	2.39558300	0.00136200
C	2.38814500	-2.53092500	-0.01362100
C	3.41745300	-3.61934500	-0.02658500
C	3.60166000	3.42770700	-0.00253700
C	-3.44358400	3.61205900	0.00100000
C	-3.62781300	-3.43535700	0.02128900
C	3.74721700	4.32612600	-1.07293800
C	4.76568400	5.28404300	-1.07549000
C	5.67288000	5.38628900	-0.00991700
C	5.52395900	4.48832000	1.06177500
C	4.51253100	3.52721500	1.06626000
C	4.32602200	-3.77292600	1.03769100
C	5.28159900	-4.78958800	1.02606900
C	5.37493100	-5.69156900	-0.04848800
C	4.47112600	-5.53453000	-1.11032100
C	3.50835800	-4.52076600	-1.10057000
C	-3.55194600	4.50971600	-1.07440000
C	-4.51395500	5.52427900	-1.07127000
C	-5.39952300	5.68563100	0.00517600
C	-5.28869700	4.78724200	1.08103800
C	-4.33355700	3.76998400	1.08006300
C	-4.52063400	-3.54113300	1.10446700
C	-5.53150900	-4.50288200	1.11147100
C	-5.69748400	-5.39526200	0.03763400
C	-4.80783200	-5.28697600	-1.04200900
C	-3.79015100	-4.32820000	-1.05121300
C	-6.79664500	-6.42896700	0.05545000
C	-6.43170100	6.78625300	0.02087300
C	6.77229600	6.41982900	-0.00422600
C	6.40942200	-6.79009700	-0.04707200
H	-5.11850200	1.46898000	-0.25367400
H	-5.18852500	-1.21095900	-0.24227000
H	1.45960300	5.09132400	0.28774600
H	-1.22014300	5.16131400	0.28161800
H	-1.48489700	-5.09928400	0.28801000
H	1.19484900	-5.16877200	0.27611000
H	5.08686300	-1.47588800	-0.31962500
H	5.15668200	1.20392900	-0.30766900
H	3.06065500	4.26829900	-1.92118100
H	4.85746500	5.96412600	-1.92708900
H	6.21169300	4.54465100	1.91073100

H	4.41902700	2.84663200	1.91617400
H	4.27460000	-3.09091500	1.89001200
H	5.96893500	-4.88686900	1.87162700
H	4.52131500	-6.21549800	-1.96467800
H	2.82271400	-4.42154000	-1.94573300
H	-2.88058400	4.40717300	-1.93059100
H	-4.57816000	6.20237900	-1.92697800
H	-5.96175300	4.88772400	1.93764100
H	-4.26811900	3.09076100	1.93368400
H	-4.41345200	-2.86497100	1.95631500
H	-6.20517700	-4.56416300	1.97130000
H	-4.91288800	-5.96296200	-1.89534100
H	-3.11728400	-4.26566300	-1.91006100
H	-6.78700800	-7.04538800	-0.85514800
H	-6.69639800	-7.10255500	0.92281300
H	-7.78911800	-5.95469900	0.13427200
H	-7.44159900	6.38549200	0.20666800
H	-6.22490900	7.51337500	0.82441100
H	-6.45207900	7.33535700	-0.93171100
H	6.75330800	7.03566200	-0.91507200
H	6.68076000	7.09418500	0.86355600
H	7.76546900	5.94563500	0.06505800
H	7.42408200	-6.38394200	0.09705100
H	6.23180400	-7.50227000	0.77645100
H	6.39949600	-7.35647300	-0.98963400
C	0.30299900	-0.05056800	3.23481000
O	-0.06043200	-0.00348100	2.02851700
C	1.76843100	-0.17497800	3.56899500
H	1.96812100	-1.09840800	4.13302700
H	2.33611800	-0.19678400	2.63354500
H	2.10979500	0.67515600	4.17822000
N	-0.61056900	0.00985200	4.20847700
C	-0.29392300	-0.03793400	5.63227400
H	-0.65327400	0.88040800	6.12266300
H	-0.80156600	-0.89875800	6.09532400
H	0.78131100	-0.13049700	5.80442900
C	-2.03445300	0.13267200	3.90192600
H	-2.58002500	-0.71829000	4.33882000
H	-2.42860200	1.06189600	4.34276500
H	-2.18432600	0.14759100	2.81844400
C	-0.31868500	-0.03303600	-3.22872400
O	0.03526000	-0.00132400	-2.02021600
C	-1.78432000	-0.12493700	-3.57449500
H	-1.99942000	-1.04434200	-4.13957700
H	-2.36230700	-0.13485100	-2.64534000
H	-2.10288400	0.73173700	-4.18670800
N	0.60193900	0.01403300	-4.19743400
C	0.29250100	-0.02041500	-5.62319300
H	0.66429400	0.89702800	-6.10612200
H	0.79310100	-0.88371700	-6.08932400
H	-0.78263600	-0.10058800	-5.80195200

C	2.02618400	0.10711700	-3.88364900
H	2.55624800	-0.75847200	-4.31079400
H	2.44338400	1.02455100	-4.32773100
H	2.17096200	0.12623100	-2.79990800

[(TTP)Ti(DMA)$_2$]

N	-2.09754300	0.05518700	0.02816900
N	-0.04264500	-2.08500300	-0.01544900
N	0.06511300	2.08523500	-0.01968400
N	2.12075100	-0.05546700	0.01313000
C	-2.87413500	1.19044000	-0.03585600
C	-4.24732000	0.80373600	-0.16266900
C	-4.28351300	-0.58131900	-0.15906800
C	-2.93241100	-1.03859900	-0.03015700
C	1.20719100	2.86755100	0.02523200
C	0.80472000	4.26165700	0.14638900
C	-0.55770300	4.29736400	0.15235800
C	-1.03368600	2.92606600	0.03574700
C	-1.18427800	-2.86729400	0.04269300
C	-0.78065400	-4.26134600	0.15963500
C	0.58176400	-4.29718100	0.15122000
C	1.05658000	-2.92608800	0.02803600
C	2.89603300	-1.19092000	-0.06784500
C	4.26652300	-0.80405600	-0.21942400
C	4.30239000	0.58100000	-0.21883200
C	2.95385700	1.03838900	-0.06688500
Ti	0.01180500	0.00009200	-0.00537300
C	-2.51371000	-2.41324800	0.01383500
C	-2.38507600	2.54178300	0.00507900
C	2.53625000	2.41316500	-0.01752400
C	2.40771600	-2.54204000	-0.01801800
C	3.43981900	-3.62876200	-0.02061500
C	3.62481600	3.44336400	-0.01965500
C	-3.41683600	3.62888600	0.01172800
C	-3.60166100	-3.44418300	0.02066500
C	3.78664800	4.33729700	-1.09191600
C	4.80489000	5.29695200	-1.09005800
C	5.70379100	5.40334500	-0.01854100
C	5.54428800	4.50848600	1.05470600
C	4.53058700	3.54947900	1.05403600
C	4.33904800	-3.78336800	1.05269200
C	5.29639900	-4.79864000	1.05528800
C	5.40421100	-5.70368800	-0.01587600
C	4.51268500	-5.54846500	-1.08757200
C	3.55090800	-4.53223500	-1.09137900
C	-3.54072200	4.52810800	-1.06119500
C	-4.50178900	5.54495200	-1.04957700
C	-5.37993800	5.70492400	0.03239400
C	-5.25913600	4.80430800	1.10589800
C	-4.30234300	3.78852200	1.09562400
C	-4.49458300	-3.55650000	1.10435000

C	-5.50796000	-4.51589500	1.11153800
C	-5.67966200	-5.40498400	0.03539500
C	-4.79330200	-5.29254500	-1.04589800
C	-3.77557300	-4.33241000	-1.05441900
C	-6.78091500	-6.43732200	0.05283100
C	-6.42040600	6.79844500	0.05332400
C	6.80598000	6.43477300	-0.00855000
C	6.44504200	-6.79698200	-0.00309200
H	-5.09424200	1.47986800	-0.25279800
H	-5.16464900	-1.21264400	-0.24591500
H	1.48243500	5.10787400	0.23037000
H	-1.18939500	5.17787400	0.24204200
H	-1.45759600	-5.10743300	0.25099600
H	1.21425800	-5.17770100	0.23484700
H	5.11144300	-1.48019000	-0.32690900
H	5.18128400	1.21238200	-0.32596500
H	3.10703900	4.27638500	-1.94580200
H	4.90335600	5.97433000	-1.94352800
H	6.22524500	4.56725600	1.90940900
H	4.43078100	2.87090600	1.90501900
H	4.27795200	-3.09869600	1.90244200
H	5.97321600	-4.89422200	1.90995500
H	4.57203200	-6.23219500	-1.93959200
H	2.87530100	-4.43493800	-1.94508200
H	-2.87560000	4.42715500	-1.92268400
H	-4.57120800	6.22537700	-1.90348000
H	-5.92510200	4.90387700	1.96859600
H	-4.23072100	3.10765900	1.94763500
H	-4.38501000	-2.88263800	1.95788800
H	-6.17898100	-4.57950200	1.97371800
H	-4.90143800	-5.96538200	-1.90178800
H	-3.10592300	-4.26652000	-1.91579200
H	-6.77169500	-7.05364900	-0.85811700
H	-6.68324800	-7.11248700	0.91949200
H	-7.77368600	-5.96297500	0.13041400
H	-7.43913400	6.38095300	0.12105500
H	-6.28956300	7.45916900	0.92667500
H	-6.37072500	7.42120300	-0.85192700
H	6.78121500	7.06167300	-0.91196000
H	6.72439700	7.09966900	0.86761600
H	7.79960200	5.95887100	0.04569600
H	7.46420200	-6.37935400	0.05638300
H	6.32128000	-7.45752000	0.87146700
H	6.38806300	-7.42008900	-0.90766900
C	0.39126600	-0.02649900	3.25331800
O	-0.03419800	0.00553400	2.07540700
C	1.87626700	-0.10038900	3.51990300
H	2.13312600	-1.00364200	4.09365200
H	2.39529700	-0.12534200	2.55612700
H	2.22131100	0.77334700	4.09327500
N	-0.47423900	0.00409700	4.28262400

C	-0.08322500	-0.02241000	5.68598500
H	-0.48210800	0.86620600	6.20142200
H	-0.49782300	-0.91820900	6.17676000
H	1.00377600	-0.03222000	5.80258600
C	-1.91344700	0.06597400	4.04675500
H	-2.40684100	-0.79431500	4.52716700
H	-2.32644600	0.98937000	4.48467100
H	-2.11485100	0.04996600	2.97090700
C	-0.34987800	-0.02623900	-3.26102600
O	0.05452500	0.00147100	-2.07552100
C	-1.83102200	-0.09082400	-3.55048400
H	-2.08339700	-0.98624000	-4.13821000
H	-2.36838500	-0.12405100	-2.59708200
H	-2.16307700	0.79095600	-4.11932600
N	0.53246200	0.00112200	-4.27524600
C	0.16351300	-0.01904900	-5.68482800
H	0.57724800	0.86788700	-6.19125700
H	0.57917800	-0.91671100	-6.17116000
H	-0.92151200	-0.02015600	-5.81858000
C	1.96847400	0.05519900	-4.01724500
H	2.46485400	-0.80392800	-4.49632200
H	2.39166200	0.97973800	-4.44261600
H	2.15552200	0.03106600	-2.93910000

[(TTP)TiO(DMA)]

N	-1.64420000	1.09378600	-0.60759100
N	1.18842000	1.69465000	-0.72935800
N	-1.03476400	-1.73836000	-0.65127800
N	1.79422500	-1.13728900	-0.81587600
C	-2.92433200	0.60629800	-0.44249500
C	-3.82718500	1.70453400	-0.19345100
C	-3.08552800	2.85024900	-0.22376900
C	-1.71996000	2.46646600	-0.49050800
C	-0.55449900	-3.03257900	-0.71476800
C	-1.66373000	-3.95534600	-0.73466900
C	-2.80724200	-3.21341600	-0.66217200
C	-2.41287800	-1.82674500	-0.59728700
C	0.70567500	2.98921400	-0.71101900
C	1.80780600	3.91360500	-0.82722700
C	2.95138700	3.17149400	-0.89539800
C	2.56410400	1.78320700	-0.82197000
C	3.08540500	-0.65205500	-0.77987000
C	4.00878800	-1.75438300	-0.65513000
C	3.26700000	-2.90003900	-0.63010000
C	1.88100900	-2.51213900	-0.73927600
Ti	0.04843100	-0.01486200	-1.21484400
C	-0.64415600	3.37232100	-0.58353500
C	-3.31219600	-0.74828500	-0.48087900
C	0.80067600	-3.41711900	-0.73876400
C	3.46886700	0.70324200	-0.82925700
C	4.93419100	1.01192600	-0.87182900

C	1.11764000	-4.88135700	-0.73977500
C	-4.77359300	-1.05827200	-0.36910000
C	-0.95857900	4.83571600	-0.52053600
C	0.84591200	-5.68420200	0.38070400
C	1.15252700	-7.04755700	0.37726100
C	1.73397600	-7.66121000	-0.74376100
C	2.00565700	-6.85463200	-1.86199400
C	1.70633800	-5.49093200	-1.86190400
C	5.70546900	0.69028400	-2.00245400
C	7.07070100	0.98073600	-2.04500600
C	7.71987800	1.59443700	-0.96061700
C	6.94623200	1.91374500	0.16709000
C	5.57891700	1.63026900	0.21289000
C	-5.29496700	-1.70572100	0.76274100
C	-6.66067600	-1.98807900	0.86238700
C	-7.55352200	-1.63947500	-0.16253700
C	-7.02754600	-0.99371800	-1.29530000
C	-5.66623300	-0.70479400	-1.39757000
C	-1.66008500	5.46704800	-1.56242300
C	-1.95102500	6.83185400	-1.50669100
C	-1.56184800	7.61619100	-0.40808300
C	-0.86178900	6.98173500	0.63114700
C	-0.56325900	5.61804500	0.57797000
C	-1.90804600	9.08310100	-0.33625000
C	-9.02961100	-1.93482500	-0.05829800
C	2.03709200	-9.13927000	-0.76007600
C	9.20186100	1.87566700	-0.99854600
H	-4.89191700	1.60888000	0.00094500
H	-3.42965100	3.86755500	-0.05869000
H	-1.57893800	-5.03663300	-0.79829900
H	-3.83167300	-3.57516600	-0.65596000
H	1.71837000	4.99599200	-0.85549600
H	3.97119900	3.53408400	-0.98962900
H	5.08839400	-1.66137200	-0.57528300
H	3.62580600	-3.92033400	-0.52598500
H	0.40023400	-5.23375400	1.27108400
H	0.93876200	-7.64539500	1.26783200
H	2.46037800	-7.30163500	-2.75080100
H	1.92768500	-4.88924600	-2.74700000
H	5.22689500	0.21706900	-2.86354500
H	7.64287500	0.72827300	-2.94240700
H	7.42138500	2.39313000	1.02775100
H	5.00410000	1.88742500	1.10603700
H	-4.62563400	-1.98561400	1.58000000
H	-7.03854200	-2.48870200	1.75829500
H	-7.69623200	-0.71394300	-2.11462900
H	-5.28546300	-0.20555700	-2.29210000
H	-1.97143900	4.88357800	-2.43250700
H	-2.48897800	7.29764700	-2.33739100
H	-0.54372600	7.56457400	1.50033100
H	-0.02005100	5.15192600	1.40377000

H	-1.19100600	9.63771300	0.28751600
H	-1.92653300	9.54295800	-1.33609000
H	-2.90864800	9.23056000	0.10688800
H	-9.61787100	-1.00327600	-0.00079400
H	-9.39058700	-2.48511900	-0.94247200
H	-9.26062500	-2.53357500	0.83468700
H	2.10858800	-9.54873200	0.25846700
H	1.24315700	-9.69881100	-1.28503100
H	2.98088500	-9.35153800	-1.28598100
H	9.77947500	0.98869800	-0.68407000
H	9.53737000	2.13543700	-2.01428300
H	9.47464500	2.69927400	-0.32189200
O	-0.04148900	0.00193500	-2.84884500
C	-0.08291900	0.05567600	4.32376900
O	0.84093900	0.52486500	3.64607700
C	-1.07245500	-0.91179600	3.69393800
H	-2.10094900	-0.52024800	3.72603600
H	-0.78393300	-1.05890800	2.64644300
H	-1.06955400	-1.88776500	4.20335900
N	-0.24923300	0.38075200	5.63749900
C	-1.30645300	-0.13035000	6.49479400
H	-0.87741200	-0.68092600	7.34999700
H	-1.90885500	0.70145700	6.89901500
H	-1.97573500	-0.80544400	5.95382800
C	0.67218900	1.29993200	6.28754800
H	0.13003900	2.18029200	6.67330500
H	1.17083400	0.80716900	7.13985500
H	1.42888300	1.62882700	5.56786800

[(TTP)TiOB(OPh)$_3$(DMA)]

N	-1.81016300	1.02597700	-0.19219600
N	0.99490100	1.85433100	-0.24497700
N	-0.98170800	-1.76233200	-0.17844500
N	1.82423700	-0.94584600	-0.31284300
C	-3.05683500	0.43962900	-0.20017800
C	-4.05687300	1.46430600	-0.01109200
C	-3.39845000	2.65503100	0.11565900
C	-1.98646500	2.38093000	-0.01355000
C	-0.38770600	-3.00497600	-0.10300900
C	-1.41412100	-4.01706600	-0.18464100
C	-2.61357600	-3.37313800	-0.30739400
C	-2.34406000	-1.95556800	-0.27829100
C	0.40842600	3.09416000	-0.11659400
C	1.43634500	4.10656500	-0.18415700
C	2.62901700	3.46377100	-0.35712100
C	2.35384600	2.04567200	-0.37114300
C	3.06614100	-0.35214300	-0.34995800
C	4.07587700	-1.36761000	-0.16659000
C	3.42687700	-2.55784500	0.00629800
C	2.01082100	-2.29436600	-0.09941900
Ti	0.00066500	0.04410700	-0.52016100

C	-0.97039500	3.35987000	0.02150700
C	-3.32968200	-0.94361900	-0.28985400
C	0.99646600	-3.27285900	-0.00752000
C	3.33430700	1.03253900	-0.44100400
C	4.76909200	1.44658500	-0.56144800
C	1.41149900	-4.69684100	0.19894300
C	-4.76714500	-1.36052200	-0.34856100
C	-1.38628200	4.78947700	0.19685900
C	1.06584300	-5.37862300	1.37806200
C	1.45458300	-6.70580400	1.58129300
C	2.19534300	-7.40408800	0.61532400
C	2.53839600	-6.71983900	-0.56384700
C	2.15775300	-5.39315900	-0.76984100
C	5.49560000	1.16769300	-1.73071400
C	6.83588500	1.54743300	-1.85012200
C	7.50049100	2.21201200	-0.80810100
C	6.77218500	2.48188300	0.36388400
C	5.43270800	2.11016600	0.48653100
C	-5.36962100	-2.05868700	0.71425900
C	-6.71187400	-2.43518800	0.65441900
C	-7.50541800	-2.13416400	-0.46660900
C	-6.90114800	-1.43846100	-1.52469500
C	-5.55794800	-1.05407200	-1.46815600
C	-2.04512300	5.48494400	-0.83307000
C	-2.42837900	6.81622200	-0.66529900
C	-2.17383600	7.50374700	0.53452000
C	-1.51595600	6.80621800	1.55888100
C	-1.12570600	5.47337200	1.39480700
C	-2.59856300	8.94177900	0.70438700
C	-8.95762400	-2.54077800	-0.51611500
C	2.60072100	-8.84295100	0.81972400
C	8.94298700	2.63593900	-0.93591000
H	-5.12831600	1.29211400	0.04312200
H	-3.83323500	3.63607400	0.28646000
H	-1.23889500	-5.08927600	-0.17214700
H	-3.59717700	-3.82355000	-0.40766600
H	1.26424000	5.17785300	-0.12626500
H	3.61211700	3.91354700	-0.46365700
H	5.14722400	-1.18824900	-0.13864800
H	3.87267800	-3.52977900	0.19797500
H	0.49440400	-4.86036800	2.15212000
H	1.17800600	-7.20653800	2.51337200
H	3.11301900	-7.23640900	-1.33818700
H	2.43255900	-4.89148000	-1.70022800
H	5.00470200	0.65131600	-2.55942600
H	7.37462800	1.32127100	-2.77454800
H	7.26425500	2.98931400	1.19876400
H	4.89528500	2.32620800	1.41320400
H	-4.78071700	-2.30081400	1.60234800
H	-7.15403300	-2.97236700	1.49858700
H	-7.48931100	-1.19322500	-2.41344100

H	-5.11473600	-0.51776800	-2.31048600
H	-2.24864200	4.97821300	-1.77964100
H	-2.93389800	7.33403800	-1.48568300
H	-1.30479400	7.31221300	2.50513800
H	-0.61714300	4.95540300	2.21196800
H	-2.30297400	9.33780000	1.68675900
H	-2.14954500	9.58435700	-0.07100200
H	-3.69236900	9.04761000	0.61108500
H	-9.54572900	-2.00587500	0.24882000
H	-9.40537000	-2.32364700	-1.49672500
H	-9.07858000	-3.61775400	-0.31511300
H	3.66942900	-8.99389700	0.59793700
H	2.41380400	-9.17362600	1.85178900
H	2.03757300	-9.51198200	0.14661300
H	9.41163500	2.21066900	-1.83529600
H	9.02881600	3.73416600	-1.00064300
H	9.53233100	2.32132000	-0.05956900
C	-0.19726000	-0.12280900	2.89688300
O	0.18183900	0.06919100	1.72300900
C	-1.63210400	-0.49986900	3.19351000
H	-2.10567400	0.22600000	3.87119300
H	-2.19013300	-0.52366500	2.25328300
H	-1.69220800	-1.49206500	3.66613700
N	0.67055800	0.00715800	3.92326900
C	0.33113500	-0.20230900	5.32433800
H	0.99395300	-0.96837100	5.75890500
H	0.47014000	0.73176800	5.89387000
H	-0.70364800	-0.53334500	5.44590500
C	2.05935100	0.38127900	3.68238500
H	2.30731400	1.28880500	4.25642600
H	2.73324000	-0.42769600	4.00969800
H	2.21222300	0.57127100	2.61567000
O	-0.09937300	0.05481000	-2.23102900
O	0.67507800	-0.23221300	-4.51222500
O	-1.65327100	-0.66179000	-3.83615800
O	-0.82164600	1.54762900	-3.99873500
B	-0.45849200	0.16798500	-3.68476100
C	1.18429200	-1.47147100	-4.67571500
C	2.28629400	-1.59526600	-5.54772700
C	0.68535800	-2.62959600	-4.04798000
C	2.86790200	-2.83994700	-5.79021900
H	2.66914000	-0.69371600	-6.03173200
C	1.27494900	-3.87295600	-4.30492800
H	-0.16608500	-2.55425400	-3.37331700
C	2.36524700	-3.99272200	-5.17279700
H	3.72037500	-2.91064400	-6.47172300
H	0.86744100	-4.76227700	-3.81618900
H	2.81635900	-4.96876900	-5.36680800
C	-0.01147700	2.59419600	-4.26755800
C	-0.60952000	3.72911400	-4.85380300
C	1.36780900	2.62874500	-3.98503900

C	0.14991200	4.86103100	-5.15510500
H	-1.68006500	3.69877500	-5.07044300
C	2.11843400	3.76795100	-4.29466100
H	1.84607100	1.76537500	-3.52523200
C	1.52313700	4.88979100	-4.88123500
H	-0.33649200	5.72790500	-5.61140600
H	3.18836100	3.77434100	-4.06737500
H	2.11901400	5.77421000	-5.11936300
C	-2.30990900	-0.88616000	-4.99540200
C	-1.95119100	-0.32253000	-6.23649900
C	-3.42558100	-1.74731500	-4.94271300
C	-2.69681100	-0.61945500	-7.38263200
H	-1.09236700	0.34379500	-6.29983600
C	-4.16102600	-2.03443300	-6.09364000
H	-3.69984500	-2.18432100	-3.98008000
C	-3.80345800	-1.47303100	-7.32592900
H	-2.40214900	-0.17220400	-8.33647300
H	-5.02261700	-2.70480400	-6.02598800
H	-4.37885400	-1.69785200	-8.22719800

Ferrocene

C	1.69241500	-0.84080700	-0.87933300
C	1.69194300	-1.09623900	0.52795000
C	1.69208300	0.16328900	1.20573200
C	1.69218600	1.19713000	0.21732500
C	1.69235600	0.57653800	-1.07129100
H	1.67044100	-1.59332400	-1.66649200
H	1.66967700	-2.07742400	1.00033700
H	1.66996400	0.30942000	2.28485500
H	1.66996400	2.26861700	0.41176200
H	1.67030600	1.09259900	-2.03024100
C	-1.69221400	-1.19698400	-0.21808100
C	-1.69217000	-0.57727400	1.07095800
C	-1.69227000	0.84020200	0.87997000
C	-1.69204100	1.09659700	-0.52713700
C	-1.69228800	-0.16246700	-1.20578000
H	-1.67002200	-2.26833600	-0.41326600
H	-1.66991600	-1.09399400	2.02954900
H	-1.67014000	1.59218800	1.66763100
H	-1.66988300	2.07809900	-0.99887100
H	-1.67039200	-0.30785900	-2.28500800
Fe	0.00000000	0.00000400	-0.00008200

Ferrocene^{+}

C	1.70730900	0.61776800	-1.04900700
C	1.70701100	-0.80693000	-0.91170200
C	1.70682700	-1.11656100	0.48565500
C	1.70691200	0.11669900	1.21193000
C	1.70724700	1.18862300	0.26350600
H	1.66509300	1.16987300	-1.98652000
H	1.66452600	-1.52799800	-1.72642200

H	1.66401600	-2.11426200	0.91961300
H	1.66414500	0.22103900	2.29489600
H	1.66487100	2.25088300	0.49891100
C	-1.70695900	-0.11603000	-1.21197600
C	-1.70691100	-1.18858200	-0.26425800
C	-1.70705600	-0.61858400	1.04863300
C	-1.70719100	0.80620300	0.91225400
C	-1.70719000	1.11675000	-0.48489400
H	-1.66423200	-0.21963200	-2.29501500
H	-1.66424500	-2.25067300	-0.50036700
H	-1.66462100	-1.17128000	1.98578800
H	-1.66486000	1.52676200	1.72743500
H	-1.66469200	2.11474900	-0.91819800
Fe	0.00000000	0.00016900	-0.00003800

Ph_2S

C	1.46065800	-0.60285200	0.91843200
C	1.40804800	0.50618300	0.05708900
C	2.52490500	0.82686200	-0.73035300
C	3.68567500	0.04990000	-0.65017500
C	3.73303800	-1.06109200	0.19710400
C	2.61512200	-1.38641700	0.97590200
H	0.59960700	-0.85077700	1.54240400
H	2.48252400	1.68208600	-1.40882800
H	4.55098700	0.30939100	-1.26537400
H	4.63641200	-1.67337300	0.25098200
H	2.64565800	-2.25102800	1.64399800
S	-0.00004600	1.59892000	0.00003300
C	-1.40806200	0.50608900	-0.05706700
C	-1.46035200	-0.60338600	-0.91787700
C	-2.52521200	0.82714000	0.72979600
C	-2.61478100	-1.38698900	-0.97537100
H	-0.59906800	-0.85162600	-1.54140100
C	-3.68594600	0.05012000	0.64958800
H	-2.48311300	1.68269400	1.40787700
C	-3.73299200	-1.06129600	-0.19714300
H	-2.64506300	-2.25193600	-1.64304300
H	-4.55147100	0.30991700	1.26435800
H	-4.63633200	-1.67362600	-0.25104900

Ph_2SO

C	1.87470200	0.57874300	0.98019300
C	1.37237100	0.27525400	-0.28863700
C	1.94212400	-0.73260400	-1.07321600
C	3.01672500	-1.46880400	-0.56302100
C	3.52027800	-1.18276900	0.71073700
C	2.95274100	-0.15790000	1.47846000
H	1.42831200	1.38896400	1.56055500
H	1.55732000	-0.93979300	-2.07508800
H	3.46775600	-2.25939600	-1.16744400

H	4.36421300	-1.75462200	1.10404600
H	3.35327800	0.06996900	2.46936800
S	0.00033700	1.24207400	-0.96927100
O	-0.01423900	2.55342400	-0.24353000
C	-1.37022700	0.25956500	-0.30557900
C	-1.84262100	-0.83267700	-1.04113000
C	-1.96954700	0.63685200	0.89891300
C	-2.91697900	-1.57628700	-0.54309600
H	-1.38199200	-1.10134700	-1.99534900
C	-3.04678800	-0.10990500	1.38588800
H	-1.59687600	1.51046800	1.43759900
C	-3.51747500	-1.21651200	0.66917100
H	-3.29194100	-2.43242500	-1.10901500
H	-3.52230200	0.17559600	2.32751500
H	-4.36106900	-1.79572800	1.05228300

$[Ph_2SO]^-$

C	-1.70301700	-0.28781300	-1.11324800
C	-1.39246600	0.48046500	0.04801500
C	-2.14465600	0.25700300	1.23110400
C	-3.15294000	-0.69821800	1.26257300
C	-3.43950500	-1.47954700	0.11800900
C	-2.70493100	-1.25580300	-1.05872300
H	-1.17934000	-0.09103000	-2.05030600
H	-1.91890800	0.84687600	2.12551900
H	-3.73205300	-0.84115000	2.18005200
H	-4.22685900	-2.23671800	0.14576500
H	-2.93296200	-1.83963800	-1.95686300
S	-0.00000100	1.56400900	0.14809700
O	0.00000100	2.38826500	-1.13883600
C	1.39246700	0.48046500	0.04801600
C	2.14465200	0.25700000	1.23110600
C	1.70302100	-0.28780900	-1.11324700
C	3.15293700	-0.69822000	1.26257500
H	1.91890200	0.84687100	2.12552200
C	2.70493400	-1.25580000	-1.05872300
H	1.17934600	-0.09102300	-2.05030600
C	3.43950500	-1.47954600	0.11801100
H	3.73204800	-0.84115500	2.18005600
H	2.93296700	-1.83963300	-1.95686400
H	4.22685900	-2.23671700	0.14576700

$[(TTP)Ti]^+$

N	-1.83103900	0.95021000	0.00299400
N	1.82992300	-0.95405800	-0.02048200
N	0.94523700	1.83318100	0.02475200
N	-0.94769100	-1.83361100	-0.00034400
Ti	-0.00014700	-0.00113000	-0.00021800
C	-2.05033800	2.31850700	0.01315300
C	3.07083900	-0.33989100	0.03161300
C	-3.07306300	0.33682000	0.02370400

C	2.04612400	-2.32064400	0.04314200
C	0.33432100	3.07459100	-0.03553300
C	2.31332400	2.04658300	-0.02917500
C	-2.31624200	-2.04824300	-0.02361100
C	-0.33821100	-3.07806500	-0.00698400
C	-1.05697800	3.32199900	-0.01461200
C	3.31676000	1.05157800	0.00005400
C	-3.31790100	-1.05345700	-0.00098300
C	1.05197100	-3.32503100	0.02395100
C	-3.46718700	2.56595100	0.05840700
C	4.08826300	-1.34982100	0.15193800
C	-4.09590300	1.34819400	0.05121600
C	3.45847500	-2.56745700	0.15639800
C	1.34605600	4.09060200	-0.14838400
C	2.56247000	3.45849700	-0.14745100
C	-2.56897900	-3.46430500	-0.04984300
C	-1.35328500	-4.09704500	-0.05286600
H	-3.93413100	3.54654800	0.09154700
H	5.15482900	-1.16173500	0.23989900
H	-5.16604700	1.15981500	0.06781900
H	3.92120400	-3.54666400	0.24527200
H	1.15977100	5.15767500	-0.23485400
H	3.54238800	3.91958700	-0.23615700
H	-3.55201400	-3.92721100	-0.06808300
H	-1.16972400	-5.16758100	-0.08364700
C	-4.74811100	-1.50669200	-0.00265600
C	-5.34160100	-2.01904300	-1.16807100
C	-5.52968500	-1.43422200	1.16271500
C	-6.67338500	-2.44172800	-1.16612200
H	-4.75510900	-2.08582000	-2.08796000
C	-6.85985400	-1.86051100	1.15927900
H	-5.08852500	-1.04763900	2.08500300
C	-7.45966900	-2.36926700	-0.00475600
H	-7.10982800	-2.83593100	-2.08825900
H	-7.44245600	-1.79975400	2.08299300
C	-1.50683600	4.75123100	-0.02139900
C	-2.16698400	5.30138300	-1.13338200
C	-1.28278300	5.58024400	1.09160600
C	-2.58478300	6.63434200	-1.13046800
H	-2.35088600	4.68025800	-2.01356500
C	-1.70729600	6.91063600	1.09072000
H	-0.78085500	5.17451000	1.97357400
C	-2.36306700	7.46604400	-0.02051700
H	-3.09393000	7.03615800	-2.01108100
H	-1.52851000	7.52871900	1.97519700
C	4.74428300	1.50441100	-0.00239300
C	5.58606300	1.23716600	-1.09618600
C	5.28361200	2.20469900	1.09082400
C	6.91708700	1.65983200	-1.09566300
H	5.19177900	0.69760700	-1.96087200
C	6.61713200	2.61958200	1.08818400

H	4.65495900	2.41503500	1.95965800
C	7.45963800	2.36015200	-0.00552000
H	7.54710700	1.44065200	-1.96240400
H	7.01207800	3.15264200	1.95770300
C	1.50751400	-4.75241500	0.03665700
C	1.28139200	-5.57813600	1.15141400
C	2.16977100	-5.30584200	-1.07270800
C	1.70555400	-6.90881800	1.15423900
H	0.77185700	-5.17231400	2.02891800
C	2.58717000	-6.63873500	-1.06608500
H	2.34899800	-4.68871300	-1.95670900
C	2.36738100	-7.46606900	0.04744800
H	1.51784400	-7.52704700	2.03674100
H	3.09050300	-7.04499000	-1.94802600
C	2.84847200	-8.89596600	0.06496700
H	2.89370300	-9.31940000	-0.94968100
H	3.86437300	-8.96404600	0.49221700
H	2.19550700	-9.53449900	0.67907800
C	-8.90637000	-2.79805400	-0.01193700
H	-9.21007800	-3.21013500	0.96242800
H	-9.56987700	-1.94096100	-0.22251200
H	-9.09938600	-3.55682800	-0.78506100
C	-2.79031900	8.91300400	-0.03045500
H	-3.04718000	9.26497900	0.98017700
H	-1.97731600	9.56065800	-0.40302400
H	-3.65976500	9.07338100	-0.68559400
C	8.88991700	2.83995000	-0.02003900
H	8.96066500	3.85212600	-0.45566000
H	9.30812500	2.89440200	0.99639600
H	9.53113600	2.18106900	-0.62480900

(TTP)Ti

N	-1.84801600	0.95731700	0.00950600
N	1.84569800	-0.95962900	-0.00951300
N	0.95711500	1.84595300	0.01720900
N	-0.95952400	-1.84798400	-0.00162200
Ti	-0.00102400	-0.00109100	0.00385900
C	-2.05902000	2.32514000	0.00976200
C	3.08454000	-0.34364500	0.02725300
C	-3.08728200	0.34123100	0.01842000
C	2.05492100	-2.32716200	0.02922700
C	0.34063300	3.08452900	-0.02094400
C	2.32455300	2.05576300	-0.02011200
C	-2.32740900	-2.05783800	-0.01125100
C	-0.34436300	-3.08781300	-0.00166300
C	-1.05422900	3.32055800	-0.00960100
C	3.32077000	1.05124000	0.00318300
C	-3.32243000	-1.05312700	0.00329100
C	1.05035700	-3.32324500	0.01793300
C	-3.48355500	2.57524000	0.03301700
C	4.10735400	-1.36298300	0.11046700

C	-4.11308800	1.36070900	0.02851900
C	3.47707900	-2.57716000	0.11001700
C	1.35935400	4.10786300	-0.10169600
C	2.57382500	3.47813900	-0.10273800
C	-2.57909400	-3.48201000	-0.02139100
C	-1.36506200	-4.11249900	-0.02545300
H	-3.94886400	3.55755900	0.04780200
H	5.17666300	-1.17692900	0.17069300
H	-5.18403800	1.17391500	0.03444500
H	3.93994100	-3.55899500	0.16949200
H	1.17234300	5.17712300	-0.16077900
H	3.55522300	3.94172600	-0.16288600
H	-3.56223500	-3.94594400	-0.02769100
H	-1.18000900	-5.18357100	-0.04028800
C	-4.75387200	-1.50654600	0.00251900
C	-5.34728600	-2.03346100	-1.15683900
C	-5.54222300	-1.42263700	1.16289300
C	-6.67887100	-2.45813000	-1.15490300
H	-4.75735800	-2.11184000	-2.07380300
C	-6.87275400	-1.84937800	1.16168200
H	-5.10413300	-1.02404300	2.08169300
C	-7.46940700	-2.37278400	0.00264400
H	-7.11209100	-2.86424700	-2.07370200
H	-7.45829700	-1.77775700	2.08306200
C	-1.50556100	4.75181100	-0.02194000
C	-2.11329600	5.31404300	-1.15750200
C	-1.33742200	5.57379700	1.10581900
C	-2.53421400	6.64661300	-1.16392700
H	-2.25702600	4.69884100	-2.04950300
C	-1.76167600	6.90499200	1.09677700
H	-0.87513800	5.16105500	2.00626100
C	-2.36561300	7.46991200	-0.03865800
H	-3.00345800	7.05487300	-2.06389100
H	-1.62411300	7.51629300	1.99359700
C	4.75085900	1.50464400	0.00205500
C	5.57950500	1.29220900	-1.11335500
C	5.30865700	2.15247500	1.11790700
C	6.91149100	1.71419100	-1.11238800
H	5.17329700	0.79106700	-1.99572400
C	6.64197000	2.57036600	1.11663000
H	4.69096700	2.32309700	2.00340200
C	7.46976400	2.36362900	0.00089600
H	7.52975500	1.53515000	-1.99700700
H	7.04855100	3.06397300	2.00425800
C	1.50551200	-4.75325900	0.03001400
C	1.32937800	-5.56423500	1.16443500
C	2.11926900	-5.32721900	-1.09651200
C	1.75260400	-6.89583700	1.17064600
H	0.85525900	-5.14486600	2.05557100
C	2.53922400	-6.66000200	-1.08765500
H	2.26201000	-4.72275000	-1.99600600

C	2.36785000	-7.47069400	0.04628600
H	1.60161500	-7.50073000	2.06964300
H	3.00626500	-7.07956300	-1.98356900
C	2.85150800	-8.90033200	0.06600200
H	2.85470200	-9.33991900	-0.94295500
H	3.88441700	-8.96315500	0.45156600
H	2.22511500	-9.53019700	0.71600400
C	-8.91615900	-2.80317200	-0.00394200
H	-9.22473900	-3.19771000	0.97629800
H	-9.58108100	-1.95214100	-0.23434900
H	-9.10442900	-3.57725400	-0.76320700
C	-2.79353300	8.91723400	-0.05700600
H	-3.09509700	9.26213100	0.94384300
H	-1.96714100	9.56991600	-0.38954100
H	-3.63456900	9.08161400	-0.74760100
C	8.89987700	2.84602500	-0.01149100
H	8.96626000	3.87639400	-0.40324000
H	9.33185400	2.85613400	1.00077800
H	9.53414700	2.21469200	-0.65223800

(TTP)TiO

N	-0.00008200	-2.04382600	0.00238800
N	0.00007900	2.04380700	0.00236600
N	2.04924000	-0.00008900	0.01964800
N	-2.04924200	0.00007100	0.01965500
Ti	0.00000000	-0.00000700	0.54149200
C	1.10870200	-2.86861900	0.02182200
C	1.10892600	2.86851300	0.02179500
C	-1.10892900	-2.86853200	0.02182900
C	-1.10870500	2.86859900	0.02179800
C	2.86602800	-1.10813600	-0.08061000
C	2.86611500	1.10789200	-0.08062100
C	-2.86611800	-1.10791200	-0.08059800
C	-2.86603100	1.10811600	-0.08061300
C	2.45404200	-2.45489100	-0.03336200
C	2.45423500	2.45468000	-0.03338100
C	-2.45423700	-2.45470000	-0.03334900
C	-2.45404500	2.45487200	-0.03337700
C	0.68230300	-4.24545300	0.08859600
C	0.68263400	4.24538100	0.08856600
C	-0.68263800	-4.24540000	0.08859500
C	-0.68230700	4.24543500	0.08854700
C	4.23154200	-0.68267800	-0.27252500
C	4.23160000	0.68232500	-0.27250100
C	-4.23160000	-0.68234800	-0.27250400
C	-4.23155000	0.68265600	-0.27249500
H	1.34442300	-5.10573700	0.13209000
H	1.34482000	5.10561400	0.13206100
H	-1.34482400	-5.10563300	0.13208900
H	-1.34442600	5.10572000	0.13202800
H	5.08084000	-1.34600300	-0.41107700

H	5.08095400	1.34558200	-0.41103700
H	-5.08095100	-1.34560700	-0.41104700
H	-5.08085100	1.34597900	-0.41103400
C	-3.51615000	-3.51079200	-0.06783300
C	-4.40032000	-3.67912800	1.01000300
C	-3.66153100	-4.35948700	-1.18056100
C	-5.39184000	-4.66443900	0.97737200
H	-4.30678900	-3.03683900	1.88923300
C	-4.65618400	-5.33757200	-1.21007100
H	-2.99168200	-4.24428700	-2.03636000
C	-5.54100200	-5.51152900	-0.13119600
H	-6.06171300	-4.77677700	1.83449100
H	-4.74934300	-5.97865600	-2.09159700
C	3.51587300	-3.51106600	-0.06785200
C	4.40002200	-3.67948500	1.00999000
C	3.66118600	-4.35977000	-1.18058200
C	5.39145600	-4.66488000	0.97736000
H	4.30653200	-3.03720000	1.88922800
C	4.65575500	-5.33794100	-1.21009100
H	2.99134300	-4.24451700	-2.03637800
C	5.54055800	-5.51197200	-0.13121600
H	6.06130500	-4.77729100	1.83448700
H	4.74885300	-5.97903700	-2.09161400
C	3.51614500	3.51077500	-0.06786300
C	4.40037600	3.67905200	1.00993000
C	3.66153500	4.35946000	-1.18060100
C	5.39193900	4.66432000	0.97727000
H	4.30691100	3.03669900	1.88912100
C	4.65622900	5.33749900	-1.21014100
H	2.99170800	4.24423300	-2.03641400
C	5.54105100	5.51145800	-0.13126600
H	6.06191400	4.77654700	1.83432200
H	4.74945000	5.97852100	-2.09170400
C	-3.51587200	3.51105000	-0.06786800
C	-3.66118200	4.35975000	-1.18060500
C	-4.40007800	3.67941800	1.00993200
C	-4.65578300	5.33788400	-1.21014300
H	-2.99135100	4.24447500	-2.03640800
C	-5.39154600	4.66478100	0.97727300
H	-4.30665200	3.03707700	1.88913600
C	-5.54059600	5.51191600	-0.13127400
H	-4.74892600	5.97893100	-2.09169600
H	-6.06148800	4.77709600	1.83434000
O	0.00000300	0.00000300	2.17791500
C	-6.61191700	-6.57363300	-0.17532100
H	-7.30675400	-6.40358800	-1.01471300
H	-6.17464200	-7.57513200	-0.32222200
H	-7.20101500	-6.59300100	0.75299600
C	6.61145300	-6.57409400	-0.17538100
H	7.30695900	-6.40336500	-1.01408600
H	7.19984500	-6.59423400	0.75336800

H	6.17426400	-7.57545000	-0.32346400
C	6.61151900	6.57402300	-0.17513900
H	7.30180100	6.40909000	-1.01924900
H	6.17312300	7.57623700	-0.31386700
H	7.20548700	6.58839800	0.75016000
C	-6.61110500	6.57443600	-0.17522800
H	-6.17294500	7.57641300	-0.31631600
H	-7.30271400	6.40808000	-1.01798400
H	-7.20367200	6.59030200	0.75094500

$[(TTP)TiO]^{2-}$

N	-0.00099400	-2.05573800	-0.01254400
N	0.00102800	2.05569400	-0.01257600
N	2.05921900	-0.00107000	0.00203200
N	-2.05918900	0.00102500	0.00208300
Ti	0.00002200	-0.00001600	0.50623300
C	1.11912600	-2.89003500	0.03321400
C	1.12198300	2.88886800	0.03311700
C	-1.12194800	-2.88891300	0.03316500
C	-1.11909200	2.88999300	0.03318700
C	2.88351300	-1.12086700	-0.11489500
C	2.88460900	1.11792300	-0.11494400
C	-2.88457800	-1.11797000	-0.11488200
C	-2.88348500	1.12082100	-0.11484200
C	2.46818300	-2.47175100	-0.03790900
C	2.47061500	2.46922600	-0.03802900
C	-2.47058200	-2.46927100	-0.03797800
C	-2.46815400	2.47170600	-0.03788900
C	0.68810400	-4.24893100	0.14799900
C	0.69232700	4.24820100	0.14786500
C	-0.69228800	-4.24824000	0.14797600
C	-0.68806500	4.24888700	0.14798400
C	4.22880800	-0.69241800	-0.34358400
C	4.22947800	0.68814400	-0.34365600
C	-4.22945500	-0.68819400	-0.34355000
C	-4.22878100	0.69236700	-0.34351700
H	1.34536300	-5.11235000	0.21872400
H	1.35045300	5.11096500	0.21851600
H	-1.35041000	-5.11100200	0.21868000
H	-1.34532000	5.11230600	0.21873400
H	5.07871700	-1.35217700	-0.50186600
H	5.08003000	1.34706100	-0.50199600
H	-5.08001100	-1.34711500	-0.50185900
H	-5.07869200	1.35212300	-0.50180200
C	-3.53291500	-3.52222800	-0.06848200
C	-4.47966800	-3.64001900	0.96557900
C	-3.63723000	-4.43613600	-1.13684300
C	-5.47831600	-4.61937000	0.93494800
H	-4.42887400	-2.95151600	1.81309600
C	-4.63341200	-5.41399700	-1.16680700
H	-2.92340500	-4.37094700	-1.96213000

C	-5.57689600	-5.52741700	-0.13036700
H	-6.19301200	-4.68184100	1.76143100
H	-4.68410600	-6.10143800	-2.01730800
C	3.52948800	-3.52574600	-0.06830600
C	4.47610400	-3.64436800	0.96578900
C	3.63294300	-4.43984300	-1.13658800
C	5.47378900	-4.62470100	0.93527100
H	4.42596600	-2.95574100	1.81324400
C	4.62816700	-5.41868500	-1.16643700
H	2.91920300	-4.37402800	-1.96189800
C	5.57151900	-5.53292800	-0.12996900
H	6.18840000	-4.68780100	1.76178100
H	4.67820900	-6.10625000	-2.01687600
C	3.53293600	3.52219300	-0.06853600
C	4.47973900	3.63994200	0.96548000
C	3.63723500	4.43610700	-1.13689800
C	5.47841000	4.61927100	0.93482200
H	4.42902300	2.95137000	1.81294500
C	4.63343900	5.41394300	-1.16688900
H	2.92343000	4.37088500	-1.96220000
C	5.57692800	5.52737000	-0.13045200
H	6.19319600	4.68165500	1.76123300
H	4.68417600	6.10133500	-2.01742800
C	-3.52945600	3.52570400	-0.06828200
C	-3.63296900	4.43974400	-1.13661000
C	-4.47607400	3.64432400	0.96580900
C	-4.62822800	5.41854600	-1.16648900
H	-2.91928900	4.37386500	-1.96196800
C	-5.47379600	4.62462100	0.93526300
H	-4.42595500	2.95568000	1.81325100
C	-5.57153700	5.53283600	-0.12998400
H	-4.67836000	6.10602100	-2.01699600
H	-6.18844900	4.68767700	1.76174000
O	0.00004800	0.00000600	2.16531800
C	-6.65213000	-6.58686500	-0.17246800
H	-7.30072900	-6.46670300	-1.05688700
H	-6.21818700	-7.59921000	-0.23324100
H	-7.29258800	-6.54811300	0.72117400
C	6.64580700	-6.59333900	-0.17200900
H	7.29526100	-6.47305900	-1.05579000
H	7.28557400	-6.55586000	0.72218300
H	6.21099500	-7.60523400	-0.23395300
C	6.65170800	6.58729200	-0.17222300
H	7.29559600	6.47243800	-1.06073600
H	6.21715100	7.59986900	-0.22485200
H	7.29677600	6.54374300	0.71787500
C	-6.64545700	6.59362900	-0.17175100
H	-6.21013400	7.60573500	-0.22682200
H	-7.29092800	6.47781200	-1.05900300
H	-7.28912600	6.55209300	0.71945500

[(TTP)TiOB(OPh)$_3$]

N	0.66361500	2.21335400	0.26204800
N	-0.19586100	-1.65086300	-0.55892700
N	-1.72738700	0.75824800	-0.30422100
N	2.19777500	-0.19496700	0.00880400
Ti	0.16751400	0.18413200	0.37750000
C	-0.23721700	3.25800400	0.41910800
C	-1.44981800	-2.18317300	-0.82456300
C	1.92265000	2.75776900	0.47017700
C	0.69022700	-2.71840700	-0.59666500
C	-2.28164600	2.02502800	-0.21711600
C	-2.74228100	-0.07680000	-0.74364500
C	3.24991300	0.70343100	0.10958400
C	2.77112700	-1.43198300	-0.23991400
C	-1.61987100	3.19678500	0.18509000
C	-2.64795800	-1.46295600	-0.94750300
C	3.14795700	2.08166000	0.35903100
C	2.08580800	-2.63845900	-0.46470500
C	0.46858700	4.45530000	0.78956600
C	-1.34697400	-3.60866800	-0.98695400
C	1.79875000	4.14978100	0.81030600
C	-0.03122300	-3.93852600	-0.84194200
C	-3.65532400	1.98429400	-0.64033700
C	-3.93792400	0.69030000	-0.96988100
C	4.49583500	0.01606500	-0.09805800
C	4.20179900	-1.30023400	-0.30522100
H	0.00416800	5.41467100	0.99907300
H	-2.18125600	-4.27595400	-1.18367900
H	2.62855000	4.81108400	1.04293200
H	0.41396900	-4.92771000	-0.89372900
H	-4.31564800	2.84503700	-0.69635000
H	-4.87413600	0.28879200	-1.34720400
H	5.47486400	0.48633000	-0.10287400
H	4.89474000	-2.10996300	-0.51462000
C	4.41039800	2.87660500	0.48416500
C	5.29235200	2.67979700	1.55876200
C	4.74819400	3.84137600	-0.48307300
C	6.47034100	3.42577400	1.66246600
H	5.04848100	1.94761800	2.33144700
C	5.92807000	4.57796500	-0.37677600
H	4.08211300	4.00819600	-1.33329100
C	6.81308800	4.38652100	0.69905500
H	7.13460200	3.25734600	2.51470000
H	6.16830500	5.31619200	-1.14742100
C	-2.42653000	4.44982600	0.32729200
C	-3.37691600	4.57586300	1.35323600
C	-2.26028200	5.52707800	-0.56212100
C	-4.12981000	5.74638100	1.48747300
H	-3.52603600	3.75534400	2.05893300
C	-3.02045300	6.68902700	-0.42616600
H	-1.53432500	5.44849800	-1.37526200

C	-3.96875900	6.82397700	0.60340800
H	-4.85809400	5.82045000	2.29989100
H	-2.87596400	7.50825800	-1.13651000
C	-3.88950700	-2.20773300	-1.32955400
C	-4.94291800	-2.36991700	-0.41562800
C	-4.03462800	-2.76000500	-2.61504100
C	-6.10116300	-3.06538000	-0.77537300
H	-4.85282800	-1.95554900	0.59152000
C	-5.19578400	-3.44729200	-2.97005000
H	-3.23183900	-2.64041500	-3.34691600
C	-6.25175300	-3.61639400	-2.05711500
H	-6.90346500	-3.18184200	-0.04154300
H	-5.28513500	-3.85941300	-3.97935800
C	2.89525900	-3.88724200	-0.62083800
C	2.93923600	-4.57790100	-1.84472100
C	3.65153400	-4.39312200	0.45086000
C	3.70852100	-5.73443300	-1.98671600
H	2.37676300	-4.19639300	-2.70030500
C	4.41424300	-5.55414500	0.30439600
H	3.64134200	-3.87241300	1.41139300
C	4.45664400	-6.24974500	-0.91498000
H	3.73204500	-6.24413600	-2.95408300
H	4.98947600	-5.92578000	1.15700300
O	-0.07204800	-0.12126600	2.03050300
O	0.92507600	-0.60112900	4.19616000
O	-1.19755900	-1.53220200	3.67186300
O	-0.99868400	0.79190300	4.09286100
B	-0.34296700	-0.37375800	3.53540400
C	8.08492500	5.19131300	0.80367000
H	8.73525100	5.02280500	-0.07085000
H	7.87053300	6.27240600	0.83716000
H	8.65609700	4.93008500	1.70619600
C	-4.77837000	8.08928000	0.74500100
H	-5.33635600	8.31128800	-0.17960800
H	-5.50160500	8.01665300	1.57009800
H	-4.12741700	8.95795100	0.94024800
C	-7.49664900	-4.37310200	-2.44969100
H	-7.93661000	-3.96568500	-3.37453800
H	-7.27008800	-5.43485300	-2.64542000
H	-8.26117300	-4.33221900	-1.66040800
C	5.25828900	-7.51896600	-1.06361200
H	4.63358900	-8.40489800	-0.85367700
H	5.64765400	-7.63294100	-2.08672700
H	6.10678500	-7.54566600	-0.36361200
C	-2.29987100	1.15087800	4.01549900
C	-2.73798200	2.14777300	4.91059600
C	-3.22278000	0.60973800	3.10052100
C	-4.06429500	2.58197800	4.89993600
H	-2.01685900	2.56726400	5.61610400
C	-4.55032000	1.05163500	3.10148600
H	-2.90101900	-0.15645700	2.39699400

C	-4.98464600	2.03455300	3.99693800
H	-4.38223600	3.35346300	5.60686100
H	-5.25265800	0.61814400	2.38416700
H	-6.02418300	2.37087500	3.99094600
C	-0.90031100	-2.84351500	3.53492300
C	0.32558000	-3.34011600	3.05432900
C	-1.90768700	-3.76001000	3.89970000
C	0.52899900	-4.72131000	2.95499900
H	1.11447700	-2.64672800	2.76648800
C	-1.69269800	-5.13446100	3.79430600
H	-2.85769300	-3.36773300	4.27020900
C	-0.46970700	-5.62824600	3.32271500
H	1.48752600	-5.08951200	2.57958100
H	-2.48807000	-5.82632500	4.08557000
H	-0.29904700	-6.70448600	3.24223400
C	1.88255400	0.29346300	4.52749800
C	2.94096900	-0.18118700	5.32871100
C	1.88958100	1.64267400	4.12537600
C	3.97140200	0.67146100	5.72614400
H	2.93102200	-1.22983100	5.63516900
C	2.92738700	2.48798200	4.53335600
H	1.08133900	2.02746100	3.50554100
C	3.97215200	2.01647500	5.33475000
H	4.77995100	0.28166600	6.35096200
H	2.91450500	3.53369700	4.21384800
H	4.77621000	2.68608200	5.64924100

$[(TTP)TiOB(OPh)_3]^-$

N	-1.29916400	1.09882200	0.00682000
N	1.51625300	1.69015500	-0.38538400
N	-0.71913600	-1.73965800	-0.13145900
N	2.09393300	-1.14724900	-0.52102400
C	-2.57365000	0.62415400	0.22746800
C	-3.47305600	1.73421800	0.45428100
C	-2.72346000	2.87369600	0.39470400
C	-1.36385500	2.47160200	0.10856300
C	-0.23574800	-3.02986700	-0.11586900
C	-1.31712200	-3.95100500	0.15676000
C	-2.45070000	-3.20386500	0.30228300
C	-2.07048100	-1.81946000	0.12446900
C	1.05521500	2.98120500	-0.24979000
C	2.15658500	3.90806600	-0.39019900
C	3.28139900	3.16289300	-0.59983600
C	2.87768700	1.77389700	-0.58119100
C	3.37854900	-0.67128300	-0.66722500
C	4.30683300	-1.77989600	-0.70303100
C	3.56940500	-2.92201600	-0.57838200
C	2.18517600	-2.52007900	-0.45587100
Ti	0.30427300	-0.01367400	-0.87384200
C	-0.28195000	3.36742600	-0.01896400
C	-2.95949500	-0.73239700	0.26009600

C	1.10965100	-3.41714100	-0.28639700
C	3.76246800	0.68505300	-0.73038100
C	5.21695800	0.98578900	-0.93828200
C	1.41849600	-4.88507700	-0.26100800
C	-4.40841600	-1.04708200	0.48783500
C	-0.57248400	4.83270900	0.12027100
C	2.06518500	-5.47455700	0.83826700
C	2.34456300	-6.84359100	0.85800800
C	1.99408200	-7.67506600	-0.21833900
C	1.34406600	-7.08354900	-1.31396400
C	1.06010200	-5.71553200	-1.33671800
C	5.83170800	0.74497300	-2.18038800
C	7.18590600	1.02140100	-2.37629200
C	7.98100000	1.54535700	-1.34207500
C	7.36584400	1.78000900	-0.10284500
C	6.00877000	1.50789800	0.09708000
C	-5.01147800	-0.85073400	1.74210400
C	-6.35948500	-1.15508700	1.94642300
C	-7.15823600	-1.66188400	0.90770500
C	-6.55170500	-1.86048700	-0.34308600
C	-5.20271900	-1.56050100	-0.55132400
C	-1.25208100	5.53537800	-0.89097300
C	-1.52516600	6.89780400	-0.75656500
C	-1.13435800	7.61209900	0.38947700
C	-0.45809700	6.90723100	1.39680200
C	-0.17948700	5.54287800	1.26579100
C	-1.42826300	9.08693200	0.51839100
C	-8.62234800	-1.95783300	1.12379400
C	2.32715000	-9.14707300	-0.20969500
C	9.44108100	1.85292900	-1.56911900
H	-4.53926800	1.65298300	0.64829200
H	-3.06185100	3.89686700	0.53562700
H	-1.21857700	-5.02982700	0.24527500
H	-3.45243300	-3.55730600	0.53246500
H	2.07930600	4.99097300	-0.33990600
H	4.29517500	3.52354100	-0.75228500
H	5.38601500	-1.69572700	-0.80082700
H	3.93284800	-3.94605900	-0.56147300
H	2.34543700	-4.85427500	1.69352900
H	2.84191500	-7.27491100	1.73166100
H	1.05327400	-7.70390600	-2.16673600
H	0.55245000	-5.28378600	-2.20304000
H	5.23814200	0.33903800	-3.00353200
H	7.63506800	0.82659700	-3.35468700
H	7.95833100	2.18015900	0.72493700
H	5.55956700	1.69645000	1.07550600
H	-4.41397200	-0.46346400	2.57141100
H	-6.79860600	-0.99929500	2.93619800
H	-7.14478100	-2.25776000	-1.17195200
H	-4.75872200	-1.72536500	-1.53633200
H	-1.56261600	5.00918100	-1.79682000

H	-2.05172100	7.41840900	-1.56204600
H	-0.14479500	7.43236200	2.30370500
H	0.34548000	5.02018700	2.06956700
H	-1.15617800	9.46836100	1.51341500
H	-0.86720300	9.67128500	-0.23065700
H	-2.49706600	9.29867200	0.35145800
H	-9.23489500	-1.05138300	0.97374300
H	-8.98939300	-2.71898200	0.41895100
H	-8.81612800	-2.31202600	2.14795100
H	3.31555900	-9.33091300	-0.66650800
H	2.36383000	-9.54726500	0.81486100
H	1.59182100	-9.73120300	-0.78372800
H	9.96481500	0.99849200	-2.02752700
H	9.56461300	2.70813100	-2.25549900
H	9.95281200	2.10347600	-0.62836700
O	0.02654000	0.03045700	-2.63903200
O	0.45398800	-0.13379100	-5.02330400
O	-1.41473200	-1.31591900	-3.92034800
O	-1.47279000	1.03380600	-4.26754600
B	-0.56684400	-0.09621100	-3.94199100
C	-1.12337500	2.32087000	-4.42531500
C	-2.13630300	3.22397300	-4.81869700
C	0.17916700	2.82610800	-4.22330200
C	-1.85607600	4.57759800	-5.01154000
H	-3.14579100	2.83472200	-4.97366700
C	0.44675300	4.18455200	-4.42162100
H	0.97222800	2.14807800	-3.91176100
C	-0.55976700	5.07242600	-4.81750800
H	-2.65965700	5.25287900	-5.32010700
H	1.46444600	4.55252400	-4.26009400
H	-0.33935600	6.13171500	-4.97055200
C	-2.12178700	-1.83118600	-4.94061300
C	-2.86182700	-3.00677900	-4.68043800
C	-2.17333400	-1.28471800	-6.24128000
C	-3.62330500	-3.61247800	-5.68050100
H	-2.82141500	-3.43033400	-3.67366400
C	-2.94041500	-1.90280300	-7.23456300
H	-1.61101000	-0.37949400	-6.46354600
C	-3.67063600	-3.06656200	-6.96989300
H	-4.18613600	-4.52184600	-5.44976100
H	-2.96510000	-1.46142200	-8.23559300
H	-4.26669700	-3.54037100	-7.75377600
C	1.45112800	-1.02388500	-5.15375500
C	1.56959900	-2.20632300	-4.39238200
C	2.43208800	-0.76002700	-6.13574100
C	2.63565400	-3.08431400	-4.61567700
H	0.82224200	-2.42956900	-3.63216300
C	3.49041200	-1.64439400	-6.34846400
H	2.34121600	0.15503900	-6.72639300
C	3.60360200	-2.81712300	-5.58998700
H	2.70588000	-3.99443000	-4.01260700

H	4.23532400	-1.41480200	-7.11615200
H	4.43202600	-3.51004800	-5.75666600

[(TTP)TiO]$^-$

N	-0.00098400	-2.05457600	0.00822800
N	0.00102200	2.05451700	0.00826200
N	2.04758700	-0.00103600	0.02542600
N	-2.04755000	0.00098400	0.02537000
Ti	0.00000900	-0.00003700	0.53583800
C	1.11050400	-2.88438400	0.04140600
C	1.11331000	2.88324200	0.04162300
C	-1.11327300	-2.88330000	0.04156900
C	-1.11046700	2.88432600	0.04141300
C	2.86992600	-1.11802000	-0.07141300
C	2.87101400	1.11516200	-0.07120400
C	-2.87097500	-1.11521500	-0.07126900
C	-2.86989100	1.11796700	-0.07145400
C	2.47075600	-2.45297300	-0.02338800
C	2.47314600	2.45050300	-0.02303300
C	-2.47310900	-2.45055700	-0.02310100
C	-2.47072100	2.45291800	-0.02340000
C	0.69034800	-4.24206000	0.12472800
C	0.69446400	4.24133000	0.12484200
C	-0.69442700	-4.24138300	0.12489100
C	-0.69031000	4.24199800	0.12480800
C	4.24044300	-0.68188300	-0.26248700
C	4.24110300	0.67772500	-0.26238300
C	-4.24107000	-0.67777800	-0.26240200
C	-4.24040500	0.68183000	-0.26254500
H	1.34967300	-5.10465900	0.17483000
H	1.35461900	5.10328900	0.17502800
H	-1.35458100	-5.10333900	0.17513500
H	-1.34963500	5.10459500	0.17494700
H	5.09121300	-1.34422500	-0.40071000
H	5.09251600	1.33926400	-0.40049800
H	-5.09248600	-1.33931800	-0.40049400
H	-5.09117400	1.34417300	-0.40077300
C	-3.53300500	-3.50840500	-0.06476400
C	-4.43268300	-3.68005300	1.00036300
C	-3.66629200	-4.36603300	-1.17370900
C	-5.42679100	-4.66389300	0.95773700
H	-4.35067200	-3.03517300	1.87910800
C	-4.65966500	-5.34515000	-1.21453300
H	-2.98308100	-4.25582300	-2.01973700
C	-5.56141200	-5.51543800	-0.14854600
H	-6.10925300	-4.77209300	1.80588500
H	-4.74027400	-5.98974100	-2.09520600
C	3.52960400	-3.51186000	-0.06530300
C	4.42927200	-3.68449900	0.99967300
C	3.66186100	-4.36952700	-1.17433900
C	5.42239000	-4.66932600	0.95680800

H	4.34802900	-3.03962700	1.87849500
C	4.65425300	-5.34963100	-1.21540300
H	2.97861800	-4.25857700	-2.02024400
C	5.55600700	-5.52089600	-0.14957900
H	6.10486800	-4.77829000	1.80484600
H	4.73407500	-5.99423200	-2.09614100
C	3.53302400	3.50836700	-0.06473800
C	4.43277800	3.68000000	1.00032300
C	3.66626600	4.36597100	-1.17371000
C	5.42690400	4.66382400	0.95762300
H	4.35087000	3.03507200	1.87904200
C	4.65965600	5.34506500	-1.21460800
H	2.98305700	4.25570300	-2.01973200
C	5.56142900	5.51539600	-0.14864700
H	6.10948200	4.77195200	1.80568700
H	4.74028800	5.98958100	-2.09533400
C	-3.52956700	3.51181000	-0.06529500
C	-3.66189100	4.36942400	-1.17436700
C	-4.42922800	3.68443800	0.99968500
C	-4.65431200	5.34949400	-1.21543700
H	-2.97871700	4.25841200	-2.02032000
C	-5.42237900	4.66923600	0.95681300
H	-4.34800000	3.03954100	1.87849000
C	-5.55600700	5.52080800	-0.14956800
H	-4.73423400	5.99400900	-2.09622800
H	-6.10489400	4.77814500	1.80482800
O	-0.00003200	-0.00007200	2.18415200
C	-6.63233500	-6.57784300	-0.20425100
H	-7.31457700	-6.41373700	-1.05535200
H	-6.19471700	-7.58138500	-0.33712000
H	-7.23670900	-6.59126000	0.71458800
C	6.62595200	-6.58426900	-0.20559100
H	7.30888300	-6.42001000	-1.05611700
H	7.22976300	-6.59907600	0.71359700
H	6.18748300	-7.58727400	-0.33966200
C	6.63187400	6.57829500	-0.20412700
H	7.30950400	6.41928000	-1.05982000
H	6.19326600	7.58248700	-0.32887200
H	7.24105800	6.58679000	0.71158900
C	-6.62556100	6.58458700	-0.20532800
H	-6.18626200	7.58816000	-0.33250600
H	-7.30461200	6.42464700	-1.05973500
H	-7.23342200	6.59519400	0.71124500

8. Reaction optimization of Ti-catalyzed deoxygenation of *N*-oxides and epoxides

8.1 Optimization of deoxygenation of N-oxides

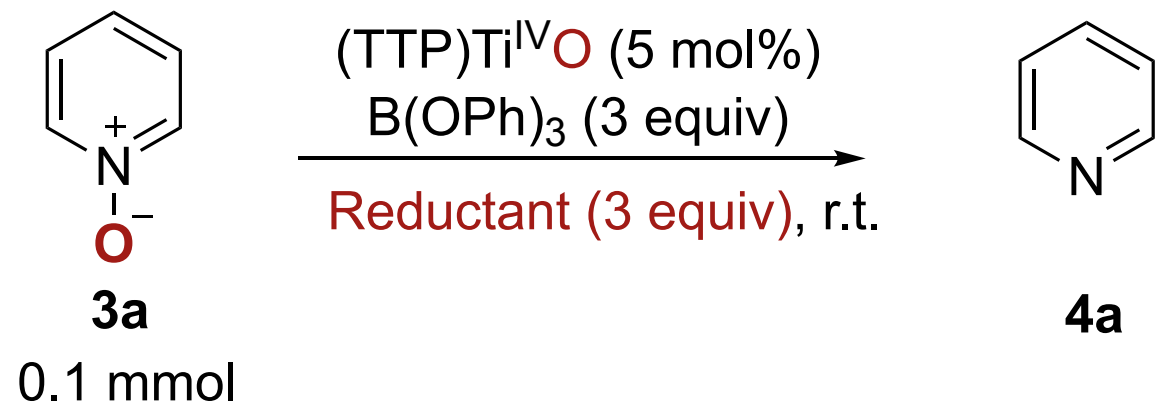

General chemical procedure: To a 1-dram vial was added pyridine *N*-oxide (0.1 mmol), (TTP)Ti^{IV}O (3.7 mg, 5 mol%, 0.005 mmol), reductant (3 equiv), $B(OPh)_3$ (87 mg, 3 equiv, 0.3 mmol) and DMA (1 mL) in the N_2-filled glovebox. The vials were sealed under N_2 atmosphere before removal from the glovebox. Reactions were then stirred in a sand bath on a stir plate at 800 rpm and heated to 70°C overnight. 1,3,5-trimethoxybenzene was added as an internal standard (0.33 equiv, 0.033 mmol) for analysis by ^{1}H NMR spectroscopy.

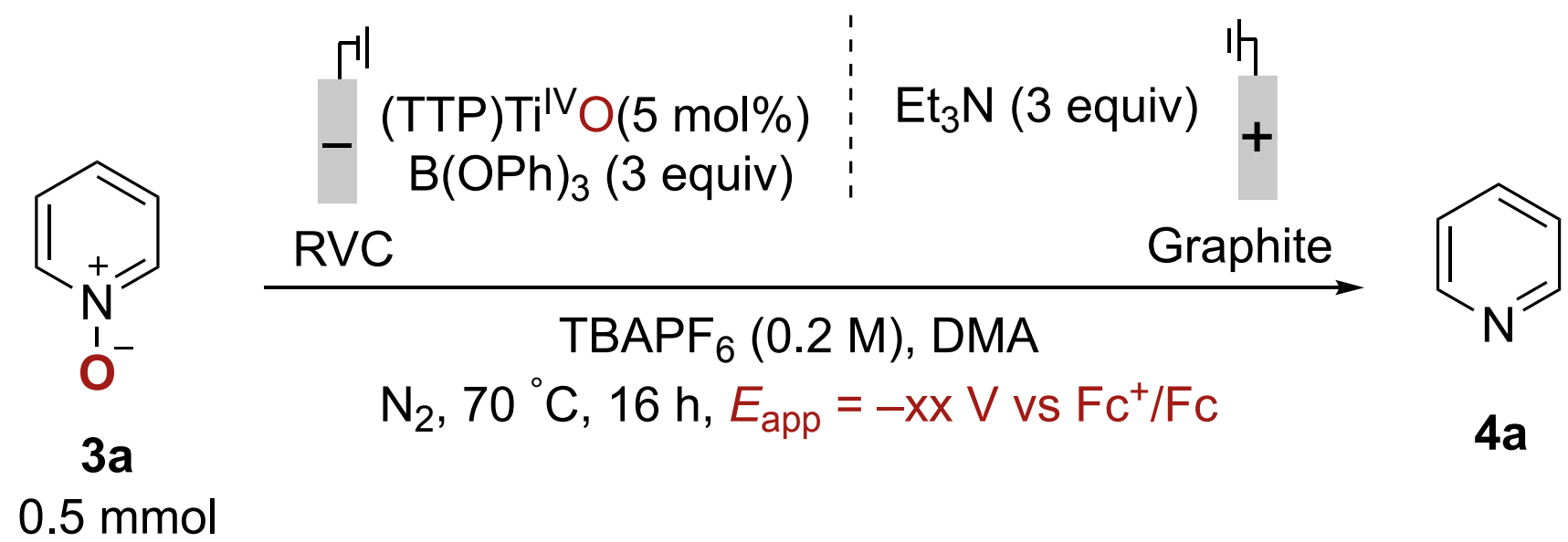

Notes: General electrochemical procedure for *N*-oxides is the same as **Condition A** (see section 3.2).

Table S10. Screening of reductants (chemical/electrochemical) for deoxygenation of **3a**.

Entry	Catalyst loading (mol%)	Equiv of $B(OPh)_3$	Reductant	Time (h)	Temperature (°C)	Conversion, 3a (%)	Yield, 4a (%)
1	5	0	Zn	16	70	>95	50
2	5	3	Mn	16	70	100	99
3	5	3	Mg	16	70	100	99
4	5	3	−1.3 V	16	70	75	72
5	5	3	−1.8 V	16	25 (rt)	100	99

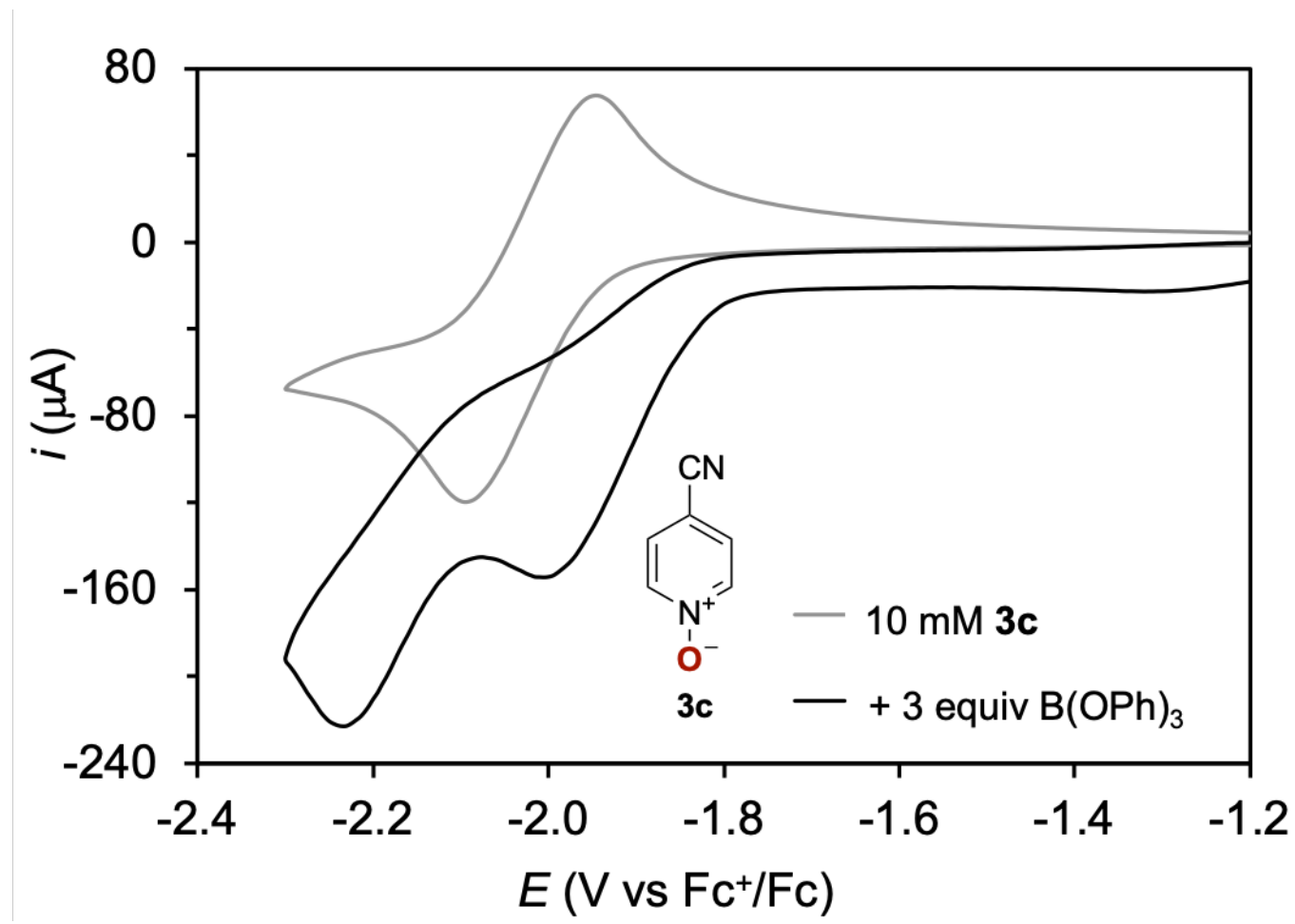

Figure S38. CV studies of reduction of **3c** (4-cyanopyridine *N*-oxide) in the absence (grey) or presence (black).

Notes: Deoxygenation of **3c** (4-cyanopyridine *N*-oxide) gave 52% isolated yields under standard deoxygenation conditions of pyridine *N*-oxides. To investigate further into the incomplete reactivity, CV studies was carried out to probe the direct reduction of **3c**. Direct reduction of **3c** occurs with an onset potential of ~−1.9 V vs Fc^+/Fc. In the presence of $B(OPh)_3$, an anodic shift was observed for the reduction feature with an onset potential of −1.8 V, which is close to the standard constant potential electrolysis conditions. Therefore, direct reduction of **3c** can happen under the electrochemical conditions and lead to decomposition of the starting material.

8.2 Optimization of deoxygenation of epoxides

General chemical deoxygenation procedure: To a 1-dram vial was added epoxides (0.1 mmol), (TTP)Ti^{IV}O (3.7 mg, 5 mol%, 0.005 mmol), reductant (2 equiv), $B(OPh)_3$ (87 mg, 3 equiv, 0.3 mmol) and solvent (1 mL) in the N_2-filled glovebox. After removing the vials from the glovebox, reactions were stirred in a sand bath on a stir plate at 800 rpm with heating to a given temperature overnight. 1,3,5-trimethoxybenzene was added as an internal standard for analysis by ^{1}H NMR spectroscopy (0.33 equiv, 0.033 mmol).

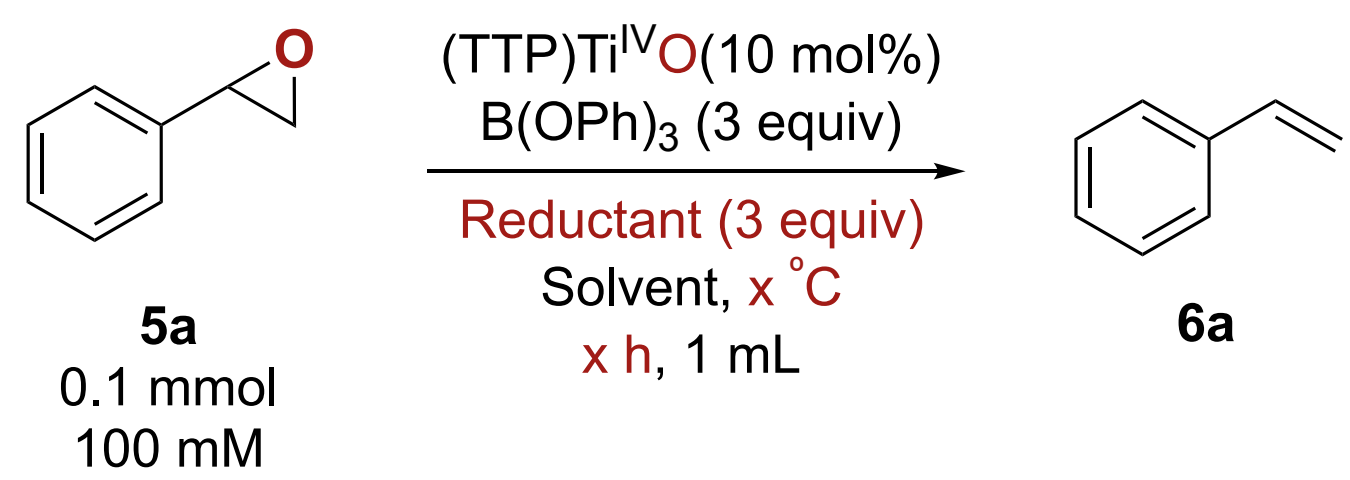

Table S11. Screening of conditions for chemical deoxygenation of **5a**.

**Entry**	Reductant	Solvent	Temp (°C)	Time (h)	**Conversion, 5a (%)**	**Yield, 6a (%)**
1	Mg	THF	70	16	100	9
2	Mn	THF	70	3	69	36
3	Zn	THF	70	3	67	**54**
4	Zn	THF	25	3	69	16
5	Zn	DMA	70	3	62	17
6	Zn	Toluene	70	3	95	52

General electrochemical procedure ***(Condition B)***: The reaction was carried out using an undivided cell equipped with a nickel foam (5.0 cm × 1.0 cm × 1.25 cm, ~1.0 cm was immersed in the solution) as cathode, Ag/$AgNO_3$ as a reference electrode, and a magnesium plate (5.0 cm × 1.0 cm × 1.0 cm, ~1.3 cm was immersed in the solution) as an anode. Epoxide (0.50 mmol), (TTP)Ti^{IV}O (36.6 mg, 10 mol%, 0.05 mmol), $B(OPh)_3$ (435 mg, 3.00 equiv, 1.5 mmol), and $TBAPF_6$ (387 mg, 0.2 M, 1 mmol) were dissolved in THF (5 mL) and added to the electrochemical cell. A constant potential electrolysis at −1.8 V was applied at 65 °C with magnetic stirring. The reaction mixture was analyzed by ^{1}H NMR spectroscopy using 1,3,5-trimethoxybenzene as an internal standard (0.33 equiv, 0.167 mmol).

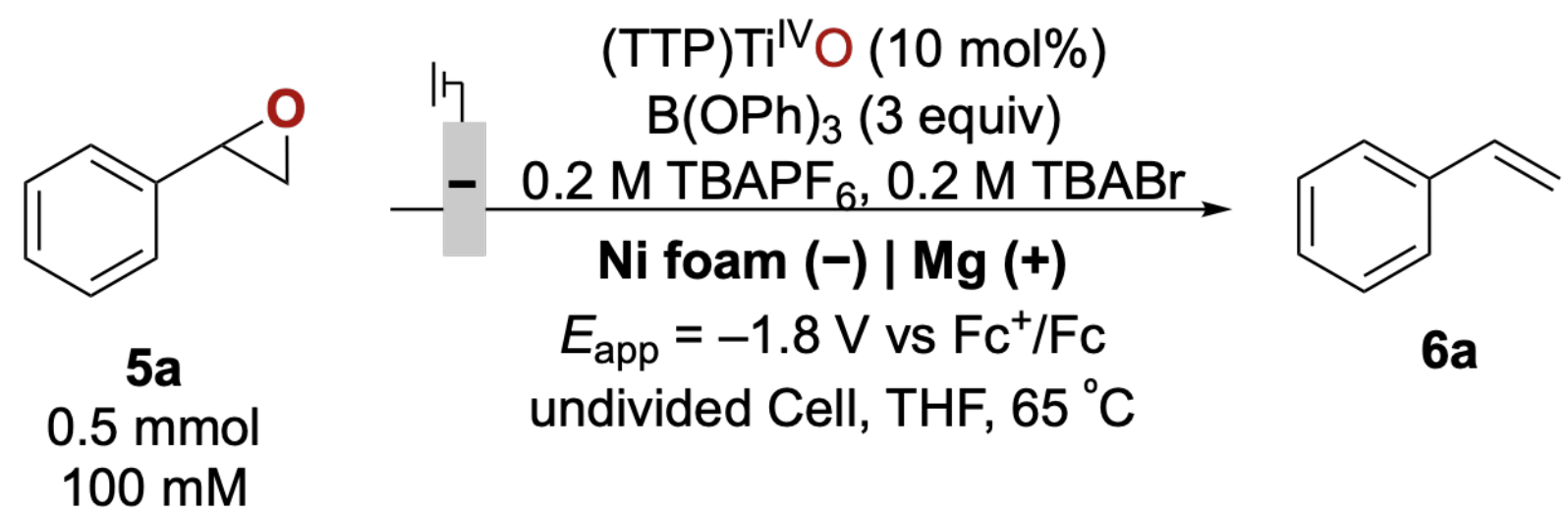

Table S12. Screening of conditions for electrochemical deoxygenation of **5a**.

**Entry**	Deviation from Standard Conditions	**Conversion, 5a** (%)	**Yield, 6a (%)**	**Charge (F/mol)**
**1**	**none**	**>99**	**81**	**1.89**
2	Graphite rod instead of Ni foam	80	65	1.43
3	RVC instead of Ni foam	61	55	1.15
4	(TTP)Ti^{IV}Cl$_2$ instead of (TTP)Ti^{IV}O	76	49	0.99
5	DMA, divided cell (Condition A)	71	11	1.55
6	Graphite (+) with Et$_3$N (2 equiv)	91	18	3.25
7	TBACl instead of TBABr	74	47	1.01
8	Divided cell, −2 mA, 2.5 F/mol	93	7	2.5
9	DME instead of THF	63	11	1.60

TBABr, *n*-tetrabutylammonium bromide.

8.3 Bulk electrolysis data for Ti-mediated deoxygenation reactions

The (TTP)Ti-catalyzed electrochemical deoxygenation of sulfoxides, *N*-oxides, and epoxides present in Figure 4 of the manuscript were conducted according to the optimized conditions outlined above:

- Sulfoxide deoxygenation: see Section 3.2, Condition A
- *N*-oxide deoxygenation: see Section 3.2, Condition A (same conditions optimized for sulfoxides)
- Epoxides: see Section 8.2, Condition B

The product characterization data are provided in Section 10 below, and a summary of the electrolysis data is included in **Table S13**:

Table S13. Electrolysis data for (TTP)Ti-catalyzed electrochemical deoxygenation of sulfoxides, *N*-oxides, and epoxides (see Figure 4 and Section 10 for chemical structures)

Product	Conversion (%)	Yield (%)	Charge (F/mol)	F.E. (%)
**2a**	100	99	2.25	90
**2b**	100	77	2.05	75
**2c**	98	80	2.09	77
**2d**	100	99	2.25	89
**2e**	89	83	2.4	69
**2f**	100	99	2.25	89
**2g**	100	99	2.25	90
**2h**	100	99	2.25	90
**2i**	92	92	2.49	74
**4a**	100	99	2.25	90
**4b**	83	83	2.28	73
**4c**	52	52	2.9	36
**4d**	91	91	2.25	81
**4e**	86	86	2.53	68
**6a**	100	81	1.89	86
**6b**	90	74	2.25	66
**6c**	89	67	1.61	83
**6d**	22	77	3.11	50
**6e**	100	53	3.06	35

9. Comparison of mediated and direct electrochemical reductive deoxygenation of Ph_2SO

9.1 General procedures for direct and mediated electrolysis

General Procedure for "direct" deoxygenation of sulfoxides: The reaction was carried out using an H-type divided cell separated by a glass frit. In the cathodic chamber, sulfoxide (0.5 mmol), triphenyl borate (435 mg, 3 equiv, 1.5 mmol), and $TBAPF_6$ (387 mg, 0.2 M, 1 mmol) were dissolved in DMA (5 mL), and the chamber was equipped with an RVC (30 PPI, 4.0 cm × 1.0 cm × 0.6 cm, ~3.0 cm was immersed in the solution) as the working electrode and $Ag/AgNO_3$ as a reference electrode. **No Ti catalyst was included.** In the anodic chamber, $TBAPF_6$ (387 mg, 0.2 M, 1 mmol) and triethylamine (209 µL, 3 equiv, 1.5 mmol) were dissolved in DMA (5 mL), and the chamber was equipped with a graphite rod (diameter: 0.6 cm; immersed height: ~1.5 cm) as an anode. Electrolysis at a constant potential of −2.8 V was carried out at 70 °C with magnetic stirring. The reaction mixture was analyzed by ^1H NMR spectroscopy using 1,3,5-trimethoxybenzene as an internal standard (0.33 equiv).

General Procedure for "mediated" deoxygenation of sulfoxide has been included in Section 3.2.

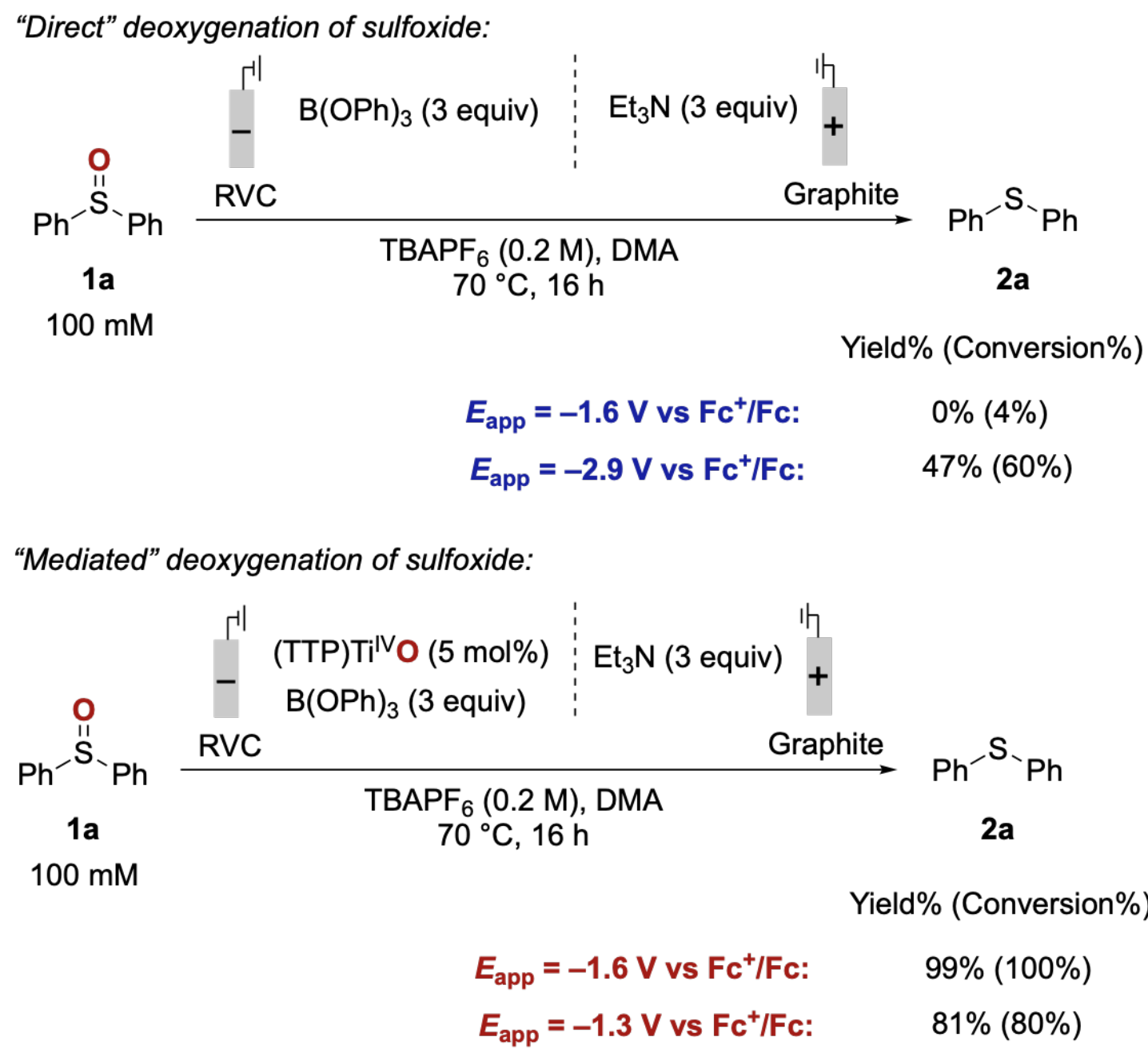

Figure S39. Constant potential electrolysis for direct and mediated deoxygenation of **1a**. Potential was monitored against Ag/$AgNO_3$ reference electrode and converted to Fc^+/Fc. The reduction peak potential of **1a** from CV studies was chosen for direct electrochemical reduction (−2.9 V vs Fc^+/Fc) of **1a**. CV conditions (blue trace): 1 mM **1a** in DMA using 0.2 M $TBAPF_6$, glassy carbon as working electrode, Pt coil as counter electrode, and Ag/$AgNO_3$ as reference electrode, scan rate 100 mV/s.

9.2 Calculation of $\Delta G^‡$ based on Figure 3E results

The activation free energy ($\Delta G^‡$) for the oxygen-atom transfer (OAT) reaction between (TTP)Ti^{II} and Ph_2SO (**1a**) was estimated based on the kinetic results shown in Figure 3E and section 5.2. The OAT reaction kinetics were assumed to be first-order in [Ph_2SO] and [Ti^{II}]. Since [Ph_2SO] >> [Ti^{II}], assuming [Ph_2SO] ≈ 0.1 M (constant) and the reaction can be approximated as pseudo-first-order in [Ti^{II}]:

$$rate_{OAT} = k[Ph_2SO][Ti^{II}] \approx k_{obs}[Ti^{II}]$$

The half-life for [Ti^{II}] in the OAT reaction is estimated from Figure 3E as approximately 300 sec. Using the equation for first-order reactions, k_{obs} can be estimated as:

$$t_{\frac{1}{2}} = \frac{ln(2)}{k_{obs}}$$

$$k_{obs} = 2.31 \times 10^{-3}\ s^{-1}, k = 2.31 \times 10^{-2}\ M^{-1}\ s^{-1}$$

The activation free energy is then determined from the Eyring equation @ 1 M [Ph_2SO]:

$$k = \frac{k_B T}{h}\ e^{-\frac{\Delta G^‡}{RT}}$$

$$\Delta G^{\ddagger} = 19.5\ kcal/mol$$

Similarly, according to the kinetic data of Ti^{II}-medated OAT transfer from coulometry experiments using BPh_3 (see section 5.1 and 5.2), the kinetic barrier can be calculated as:

$$\Delta G^{\ddagger} = 20.0\ kcal/mol$$

10. Product characterization data for (TTP)Ti-catalyzed deoxygenation reactions.

All products shown are known in the literature. Therefore, yields were determined by analysis of crude ^1H NMR spectra (relative to an internal standard), according to the following procedure. An aliquot was taken (500 µL) from the reaction mixture with 1,3,5-trimethoxybenzene as the internal standard. Aqueous KOH (1 M, 1.5 mL) was added to the aliquot, and the mixture was stirred for 1 min, followed by extraction with hexane (5 mL × 4). The organic layer was combined, dried with Na_2SO_4, and the solvent was removed under reduced pressure. $CDCl_3$ (0.5 mL) was added and a crude ^1H NMR spectrum was collected. The yield was determined relative to the internal standard added afterwards.

For **2e**, the acetyl protecting group is not compatible with the KOH conditions noted here, so the product was isolated from the reaction mixture by flash chromatography as discussed below. In addition, the crude NMR spectra for **4b**, **4d**, and **4e** were suboptimal, so these compounds were also purified by flash chromatography as discussed below.

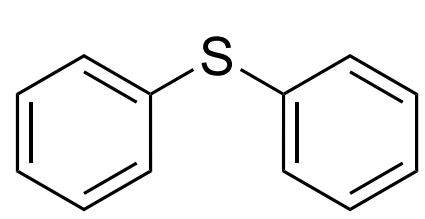

2a

Diphenyl sulfide, **2a**.

Prepared via Condition A outlined in section 3.2 from diphenyl sulfoxide **1a** (101 mg, 0.5 mmol) and 5 mol% (TTP)Ti^{IV}O (18.3 mg, 0.025 mmol) under constant potential electrolysis at −1.8 V vs Fc^+/Fc for 2.25 F/mol in DMA (~4 h, F.E. = 90%). ^1H NMR yield was calculated with 1,3,5-trimethoxybenzene (84.0 mg, 0.5 mmol) as the internal standard (99% yield). Characterization data matches with the literature values.[32]

^{1}H NMR (400 MHz, $CDCl_3$) δ 7.37–7.21 (m, 10H).

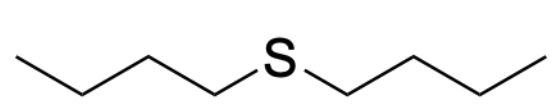

2b

Dibutyl sulfide, **2b**.

Prepared via Condition A outlined in section 3.2, from dibutyl sulfoxide **1b** (81.0 mg, 0.5 mmol) and 5 mol% (TTP)$Ti^{IV}O$ (18.3 mg, 0.025 mmol) under constant potential electrolysis at −1.8 V vs Fc^+/Fc in DMA. ^1H NMR yield was calculated with 1,3,5-trimethoxybenzene (28 mg, 0.167 mmol) as the internal standard (77% yield, F.E. = 75%). Characterization data matches with the literature values.[33]

^{1}H NMR (500 MHz, $CDCl_3$) δ 2.51 (t, 4H, *J* = 7.6 Hz).

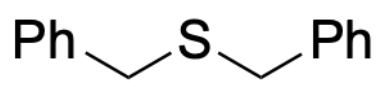

2c

Dibenzyl sulfide, **2c**.

Prepared via Condition A outlined in section 3.2, from dibenzyl sulfoxide **1c** (115.2 mg, 0.5 mmol) and 5 mol% (TTP)$Ti^{IV}O$ (18.3 mg, 0.025 mmol) under constant potential electrolysis at −1.8 V vs Fc^+/Fc in DMA (16 h). ^1H NMR yield was calculated with 1,3,5-trimethoxybenzene (168.0 mg, 1 mmol) as the internal standard (80% yield, F.E. = 77%). Characterization data matches with the literature values.[34]

^{1}H NMR (500 MHz, $CDCl_3$) δ 7.35–7.21 (m, 10H), 3.60 (s, 4H).

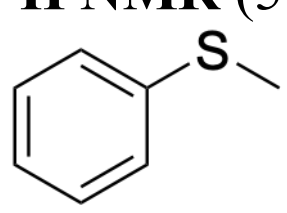

2d

Methyl phenyl sulfide, **2d.**

Prepared via Condition A outlined in section 3.2 from methyl phenyl sulfoxide **1d** (70.1 mg, 0.5 mmol) and 5 mol% (TTP)$Ti^{IV}O$ (18.3 mg, 0.025 mmol) under constant potential electrolysis at −1.8 V vs Fc^+/Fc in DMA (16 h). ^1H NMR yield was calculated with 1,3,5-trimethoxybenzene (84.0 mg, 0.5 mmol) as the internal standard (99% yield, F.E. = 89%). Characterization data matches with the literature values.[33]

^{1}H NMR (500 MHz, $CDCl_3$) δ 7.32–7.24 (m, 4H), 7.14 (tt, *J* = 5.9, 2.1 Hz, 1H), 2.49 (s, 3H).

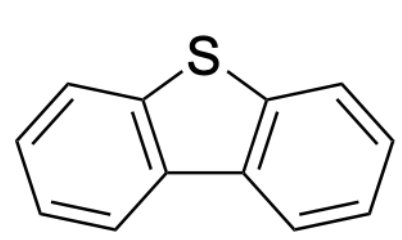

2e

Dibenzo[b,d]thiophene, **2e**.

Prepared via Condition A outlined in section 3.2 from dibenzo[b,d]thiophene 5-oxide **1e** (100.0 mg, 0.5 mmol) and 5 mol% (TTP)$Ti^{IV}O$ (18.3 mg, 0.025 mmol) under constant potential electrolysis at −1.8 V vs Fc^+/Fc in DMA (16 h). ^1H NMR yield was calculated with 1,3,5-trimethoxybenzene (84.0 mg, 0.5 mmol) as the internal standard (83% yield, F.E. = 69%). Characterization data matches with the literature values.[32]

^{1}H NMR (500 MHz, $CDCl_3$) δ 8.20–8.13 (m, 2H), 7.88–7.84 (m, 2H), 7.46 (dd, *J* = 6.0, 3.2 Hz, 4H).

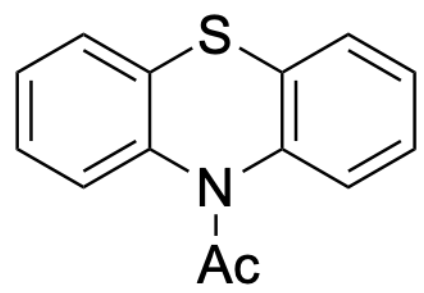

2f

10-Acetyl-10H-phenothiazine, **2f**.

Prepared via Condition A outlined in section 3.2 from 1-(5-oxido-10H-phenothiazin-10-yl)ethan-1-one **1f** (128.7 mg, 0.5 mmol) and 5 mol% (TTP)Ti^{IV}O (18.3 mg, 0.025 mmol) under constant potential electrolysis at −1.8 V vs Fc^+/Fc in DMA (~4 h). Most of the solvent was removed under reduced pressure, and the residue was extracted with 40 mL of ethyl acetate/water (1:1). The aqueous phase was extracted with ethyl acetate (10 mL × 2), and the organic phase was combined with solvent removed under reduced pressure. The crude residue was purified by column chromatography on silica gel with a gradient eluent of CH_2Cl_2:CH_3OH (97:3) with an isolated yield of 99% (F.E. = 89%). Characterization data matches with the literature values.[35]

^{1}H NMR (500 MHz, $CDCl_3$) δ 7.50 (d, *J* = 7.9 Hz, 1H), 7.44 (dd, *J* = 7.8, 1.5 Hz, 1H), 7.32 (td, *J* = 7.6, 1.4 Hz, 1H), 7.22 (td, *J* = 7.6, 1.4 Hz, 1H), 2.20 (s, 2H).

^{13}C NMR (126 MHz, $CDCl_3$) δ 169.4, 139.0, 133.1, 128.0, 127.3, 127.1, 126.9, 23.1.

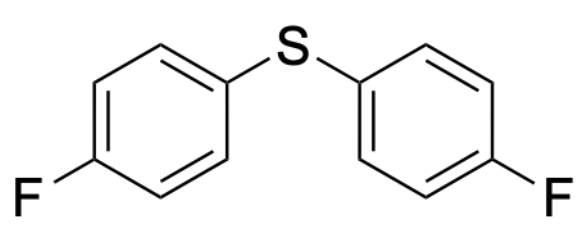

2g

Bis(4-fluorophenyl) sulfide, **2g.**

Prepared via Condition A outlined in section 3.2 (Condition A), from bis(4-fluorophenyl) sulfoxide **1g** (119.1 mg, 0.5 mmol) and 5 mol% (TTP)Ti^{IV}O (18.3 mg, 0.025 mmol) under constant potential electrolysis at −1.8 V vs Fc^+/Fc for 2.25 F/mol in DMA (~5 h). ^{1}H NMR yield was calculated with 1,3,5-trimethoxybenzene (84.0 mg, 0.5 mmol) as the internal standard (99% yield, F.E. = 90%). Characterization data matches with the literature values.[33]

^{1}H NMR (500 MHz, $CDCl_3$) δ 7.35–7.23 (m, 4H), 7.05–6.96 (m, 4H).

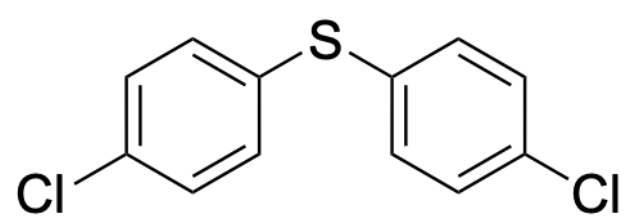

2h

Bis(4-chlorophenyl) sulfide, **2h.**

Prepared via Condition A outlined in section 3.2 (Condition A), from bis(4-chlorophenyl) sulfoxide **1h** (135.6 mg, 0.5 mmol) and 5 mol% (TTP)$Ti^{IV}O$ (18.3 mg, 0.025 mmol) under constant potential electrolysis at −1.8 V vs Fc^{+}/Fc for 2.25 F/mol in DMA (~5 h). ^{1}H NMR yield was calculated with 1,3,5-trimethoxybenzene (84.0 mg, 0.5 mmol) as the internal standard (99% yield, F.E. = 90%). Characterization data matches with the literature values.[33]

^{1}H NMR (500 MHz, $CDCl_3$) δ 7.30–7.20 (m, 8H).

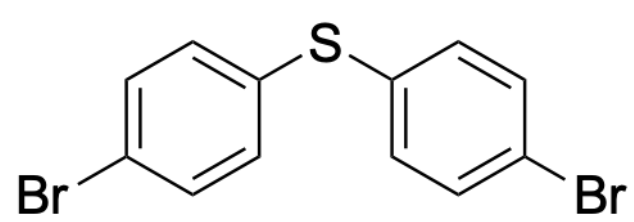

2i

Bis(4-bromophenyl) sulfide, **2i.**

Prepared via Condition A outlined in section 3.2 (Condition A), from bis(4-bromophenyl) sulfoxide **1i** (180.0 mg, 0.5 mmol) and 5 mol% (TTP)$Ti^{IV}O$ (18.3 mg, 0.025 mmol) under constant potential electrolysis at −1.8 V vs Fc^{+}/Fc in DMA (16 h). ^{1}H NMR yield was calculated with 1,3,5-trimethoxybenzene (84.0 mg, 0.5 mmol) as the internal standard (92% yield, F.E. = 74%). Characterization data matches with the literature values.[32]

^{1}H NMR (500 MHz, $CDCl_3$) δ 7.46–7.40 (m, 4H), 7.22–7.15 (m, 4H).

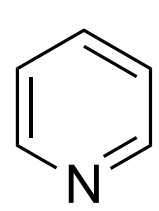

4a

Pyridine, **4a**.

Prepared via Condition A outlined in section 3.2 from pyridine *N*-oxide **3a** (47.5 mg, 0.5 mmol) and 5 mol% (TTP)$Ti^{IV}O$ (18.3 mg, 0.025 mmol) under constant potential electrolysis at −1.8 V vs Fc^{+}/Fc for 2.25 F/mol in DMA (~5 h). ^{1}H NMR yield was calculated with 1,3,5-trimethoxybenzene (28.0 mg, 0.167 mmol) as the internal standard (99% yield, F.E. = 90%). Characterization data matches with the literature values.[36]

^{1}H NMR (400 MHz, $CDCl_3$) δ 8.62 (br s, 2H), 7.72 (t, *J* = 6.4 Hz, 2H), 7.33 (d, *J* = 6.2 Hz, 2H).

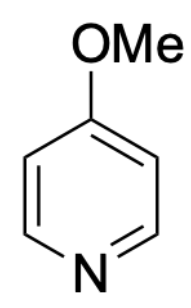

4b

4-Methoxypyridine, **4b**.

Prepared via Condition A outlined in section 3.2 from 4-methoxypyridine *N*-oxide, **3b** (62.5 mg, 0.5 mmol) and 5 mol% (TTP)$Ti^{IV}O$ (18.3 mg, 0.025 mmol) under constant potential electrolysis at −1.8 V vs Fc^+/Fc in DMA (16 h). ^{1}H NMR yield was calculated with 1,3,5-trimethoxybenzene (28.0 mg, 0.167 mmol) as the internal standard (83% yield, F.E. = 73%). Characterization data matches with the literature values.[36]

^{1}H NMR (400 MHz, $CDCl_3$) δ 8.62 (d, *J* = 4.2 Hz, 2H).

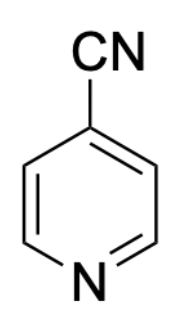

4c

4-Cyanopyridine, **4c**.

Prepared via Condition A outlined in section 3.2 from 4-cyanopyridine *N*-oxide **3c** (60.0 mg, 0.5 mmol) and 5 mol% (TTP)$Ti^{IV}O$ (18.3 mg, 0.025 mmol) under constant potential electrolysis at −1.8 V vs Fc^+/Fc in DMA (16 h). The solvent was removed under reduced pressure, and the crude residue was purified by column chromatography on silica gel with a gradient eluent of hexane:EtOAc (1:1) to afford a white solid (26.8 mg, 52% yield, F.E. = 36%). Characterization data matches with the literature values.[37]

^{1}H NMR (500 MHz, $CDCl_3$) δ 8.83–8.76 (m, 2H), 7.52 (dt, *J* = 4.3, 1.4 Hz, 2H).

^{13}C NMR (126 MHz, $CDCl_3$) δ 150.9, 125.3, 120.5, 116.5.

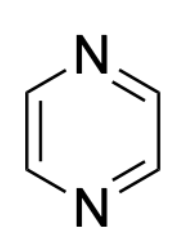

4d

Pyrazine, **4d**.

Prepared via Condition A outlined in section 3.2 from pyrazine *N*-oxide, **3d** (48.0 mg, 0.5 mmol) and 5 mol% (TTP)$Ti^{IV}O$ (18.3 mg, 0.025 mmol) under constant potential electrolysis at −1.8 V vs Fc^+/Fc in DMA for 2.25 F/mol (~5 h). The solvent was removed under reduced pressure, and the crude residue was purified by column chromatography on silica gel with a gradient eluent of hexane:EtOAc (1:1) to afford a colorless liquid (72.4 mg, 91% yield, F.E. = 81%). Characterization data matches with the literature values.[38]

^{1}H NMR (500 MHz, $CDCl_3$) δ 8.54 (s, 4H).

^{13}C NMR (126 MHz, $CDCl_3$) δ 145.2.

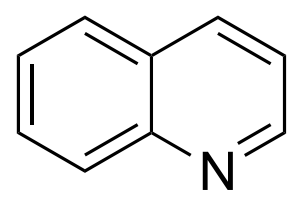

4e

Quinoline, **4e**.

Prepared via Condition A outlined in section 3.2 from quinoline *N*-oxide **3e** (72.5 mg, 0.5 mmol) and 5 mol% (TTP)Ti^{IV}O (18.3 mg, 0.025 mmol) under constant potential electrolysis at −1.8 V vs Fc^+/Fc in DMA (16 h). The solvent was removed under reduced pressure, and the crude residue was purified by column chromatography on silica gel with a gradient eluent of hexane:EtOAc (1:1). The solvent was removed under reduced pressure, and the crude residue was purified by column chromatography on silica gel with a gradient eluent of hexane:EtOAc (1:1) to afford a yellowish liquid (55.7 mg, 86% yield, F.E. = 68%). Characterization data matches with the literature values.[36]

^{1}H NMR (500 MHz, $CDCl_3$) δ 8.92 (dd, *J* = 4.3, 1.8 Hz, 1H), 8.17–8.08 (m, 2H), 7.81 (dd, *J* = 8.3, 1.4 Hz, 1H), 7.71 (ddd, *J* = 8.4, 6.8, 1.5 Hz, 1H), 7.54 (ddd, *J* = 8.1, 6.8, 1.2 Hz, 1H), 7.38 (dd, *J* = 8.3, 4.2 Hz, 1H).

^{13}C NMR (126 MHz, $CDCl_3$) δ 150.5, 148.4, 136.1, 129.6, 129.6, 128.4, 127.9, 126.6, 121.2.

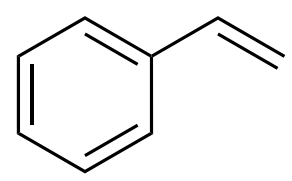

6a

Styrene, **6a**.

Prepared via Condition B outlined in section 8.2 from styrene oxide **5a** (60.0 mg, 0.5 mmol) and 10 mol% (TTP)Ti^{IV}O (36.6 mg, 0.05 mmol) under constant potential electrolysis at −1.8 V vs Fc^+/Fc in THF (~18 h). ^{1}H NMR yield was calculated with 1,3,5-trimethoxybenzene (28.0 mg, 0.167 mmol) as the internal standard (81% yield, F.E. = 86%). Characterization data matches with the literature values.[39]

^{1}H NMR (500 MHz, $CDCl_3$) δ 7.46–7.40 (m, 2H), 7.33 (dd, *J* = 8.4, 6.7 Hz, 2H), 7.28–7.24 (m, 1H), 6.74 (dd, *J* = 17.6, 10.9 Hz, 1H), 5.76 (d, *J* = 17.8 Hz, 1H), 5.25 (d, *J* = 11.0 Hz, 1H).

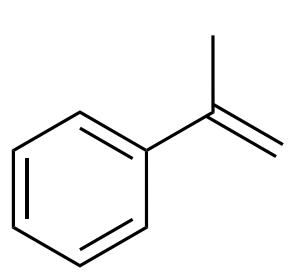

6b

Isopropenylbenzene, **6b.**

Prepared via Condition B outline in section 8.2 from 2-methyl-2-phenyloxirane **5b** (67.0 mg, 0.5 mmol) and 10 mol% (TTP)Ti^{IV}O (36.6 mg, 0.05 mmol) under constant potential electrolysis at −1.8 V vs Fc^+/Fc in THF (18 h). ^{1}H NMR yield was calculated with 1,3,5-trimethoxybenzene (28.0 mg, 0.167 mmol) as the internal standard (74% yield, F.E. = 66%). Characterization data matches with the literature values.[39]

^{1}H NMR (500 MHz, $CDCl_3$) δ 7.50–7.44 (m, 2H), 7.37–7.30 (m, 2H), 7.30–7.23 (m, 1H), 5.37 (dd, *J* = 1.6, 0.8 Hz, 1H), 5.09 (p, *J* = 1.6 Hz, 1H), 2.16 (s, 3H).

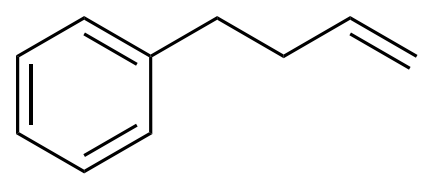

6c

4-Phenylbutene, **6c.**

Prepared via Condition B outlined in section 8.2 from 1,2-epoxy-4-phenylbutane **5c** (74.0 mg, 0.5 mmol) and 10 mol% (TTP)Ti^{IV}O (36.6 mg, 0.05 mmol) under constant potential electrolysis at −1.8 V vs Fc^{+}/Fc in THF (18 h). ^{1}H NMR yield was calculated with 1,3,5-trimethoxybenzene (28.0 mg, 0.167 mmol) as the internal standard (67% yield, F.E. = 83%). Characterization data matches with the literature values.[39]

^{1}H NMR (500 MHz, $CDCl_3$) δ 7.32–7.17 (m, 5H), 5.88 (ddt, *J* = 16.9, 10.2, 6.6 Hz, 1H), 5.06 (dq, *J* = 17.1, 1.7 Hz, 1H), 4.99 (dq, *J* = 10.2, 1.4 Hz, 1H), 2.74 (dd, *J* = 9.0, 6.7 Hz, 2H).

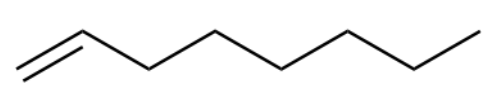

6d

1-Octene, **6d.**

Prepared via Condition B outlined in section 8.2 (Condition B), from 1,2-Epoxyoctane, **5d** (64.0 mg, 0.5 mmol) and 10 mol% (TTP)Ti^{IV}O (36.6 mg, 0.05 mmol) under constant potential electrolysis at −1.8 V vs Fc^{+}/Fc in THF (18 h). ^{1}H NMR yield was calculated with 1,3,5-trimethoxybenzene (28.0 mg, 0.167 mmol) as the internal standard (77% yield, F.E. = 50%). Characterization data matches with the literature values.[25]

^{1}H NMR (500 MHz, $CDCl_3$) δ 7.32–7.14 (m, 5H), 5.83 (ddt, *J* = 16.9, 10.0, 6.7 Hz, 1H), 5.01 (dd, *J* = 17.1, 2.1 Hz, 1H), 4.95 (d, *J* = 10.2 Hz, 1H).

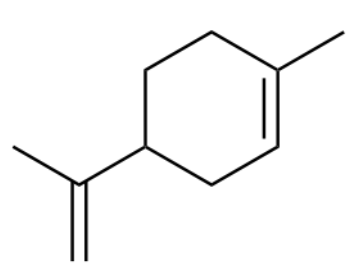

6e

1-Methyl-4-(prop-1-en-2-yl)cyclohex-1-ene (Limonene), **6e**

Prepared via Condition B outlined in section 8.2 from 1-methyl-4-(prop-1-en-2-yl)-7-oxabicyclo[4.1.0]heptane **5e** (76.1 mg, 0.5 mmol) and 10 mol% (TTP)Ti^{IV}O (36.6 mg, 0.05 mmol) under constant potential electrolysis at −1.8 V vs Fc^{+}/Fc in THF (18 h). ^{1}H NMR yield was calculated with 1,3,5-trimethoxybenzene (28.0 mg, 0.167 mmol) as the internal standard (53% yield, F.E. = 35%). Characterization data matches with the literature values.[40,41]

^{1}H NMR (500 MHz, $CDCl_3$) δ 5.44–5.41 (m, 1H).

11. Crystallographic data for Ti-porphyrin complexes

11.1. (TTP)Ti^{IV}O

CCDC Deposition Number: 2543659

Data collection

An orange crystal with approximate dimensions 0.12 × 0.04 × 0.04 mm^3 was selected under oil under ambient conditions and attached to the tip of a MiTeGen MicroMount©. The crystal was mounted in a stream of cold nitrogen at 130(1) K and centered in the X-ray beam by using a video camera.

The crystal evaluation and data collection were performed on a Bruker D8 VENTURE PhotonIII four-circle diffractometer with Cu Kα (λ = 1.54178 Å) radiation and the detector to crystal distance of 5.0 cm.[42]

The initial cell constants were obtained from a 180° φ scan conducted at a 2θ = 50° angle with the exposure time of 1 second per frame. The reflections were successfully indexed by an automated indexing routine built in the APEX6 program. The final cell constants were calculated from a set of 9775 strong reflections from the actual data collection.

The data were collected by using the full sphere data collection routine to survey the reciprocal space to the extent of a full sphere to a resolution of 0.78 Å. A total of 71171 data were harvested by collecting 48 sets of frames with 0.9º scans in ω and φ with an exposure time 1–18 seconds per frame. These highly redundant datasets were corrected for Lorentz and polarization effects. The absorption correction was based on fitting a function to the empirical transmission surface as sampled by multiple equivalent measurements.[43]

Structure solution and refinement

The systematic absences in the diffraction data were uniquely consistent for the space group $P2_1/n$ yet only the non-centrosymmetric space group $P2_1$ yielded chemically reasonable and computationally stable results of refinement.[44–49]

A successful solution by intrinsic phasing provided most non-hydrogen atoms from the *E*-map. The remaining non-hydrogen atoms were located in an alternating series of least-squares cycles and difference Fourier maps. All non-hydrogen atoms were refined with anisotropic displacement coefficients unless specified otherwise. All hydrogen atoms were included in the structure factor calculation at idealized positions and were allowed to ride on the neighboring atoms with relative isotropic displacement coefficients.

The chemical composition of the structure is a Ti complex with one solvent molecule of toluene.

In the Ti complex there are two disordered parts: the Ti=O fragment is disordered over two positions with the major component contribution of 0.814(3). The ring fragment between C15 and C20 including the tolyl substituent at C16 is equally disordered over two positions.

The toluene molecule is disordered over two positions with the major component contribution of 0.814(3). The minor component was refined isotropically with an idealized geometry.[50] All disordered fragments were refined with atomic displacement parameter restraints.

The absolute structure was established by resonant scattering effects.

The final least-squares refinement of 707 parameters against 9177 data resulted in residuals R (based on F^2 for $I\geq 2\sigma$) and wR (based on F^2 for all data) of 0.0443 and 0.1324, respectively. The final difference Fourier map was featureless.

Summary

Crystal Data for $C_{55}H_{44}N_4OTi$ (M =824.84 g/mol): monoclinic, space group $P2_1$ (no. 4), a = 14.7501(12) Å, b = 9.5738(7) Å, c = 15.2688(16) Å, β = 95.539(7)°, V = 2146.1(3) Å^3, Z = 2, T = 130.00 K, μ(CuKα) = 2.038 mm^{-1}, $Dcalc$ = 1.276 g/cm^3, 71171 reflections measured (5.816° ≤ 2Θ ≤ 158.574°), 9177 unique (R_{int} = 0.0398, R_{sigma} = 0.0234) which were used in all calculations. The final R_1 was 0.0443 ($I > 2\sigma(I)$) and wR_2 was 0.1324 (all data).

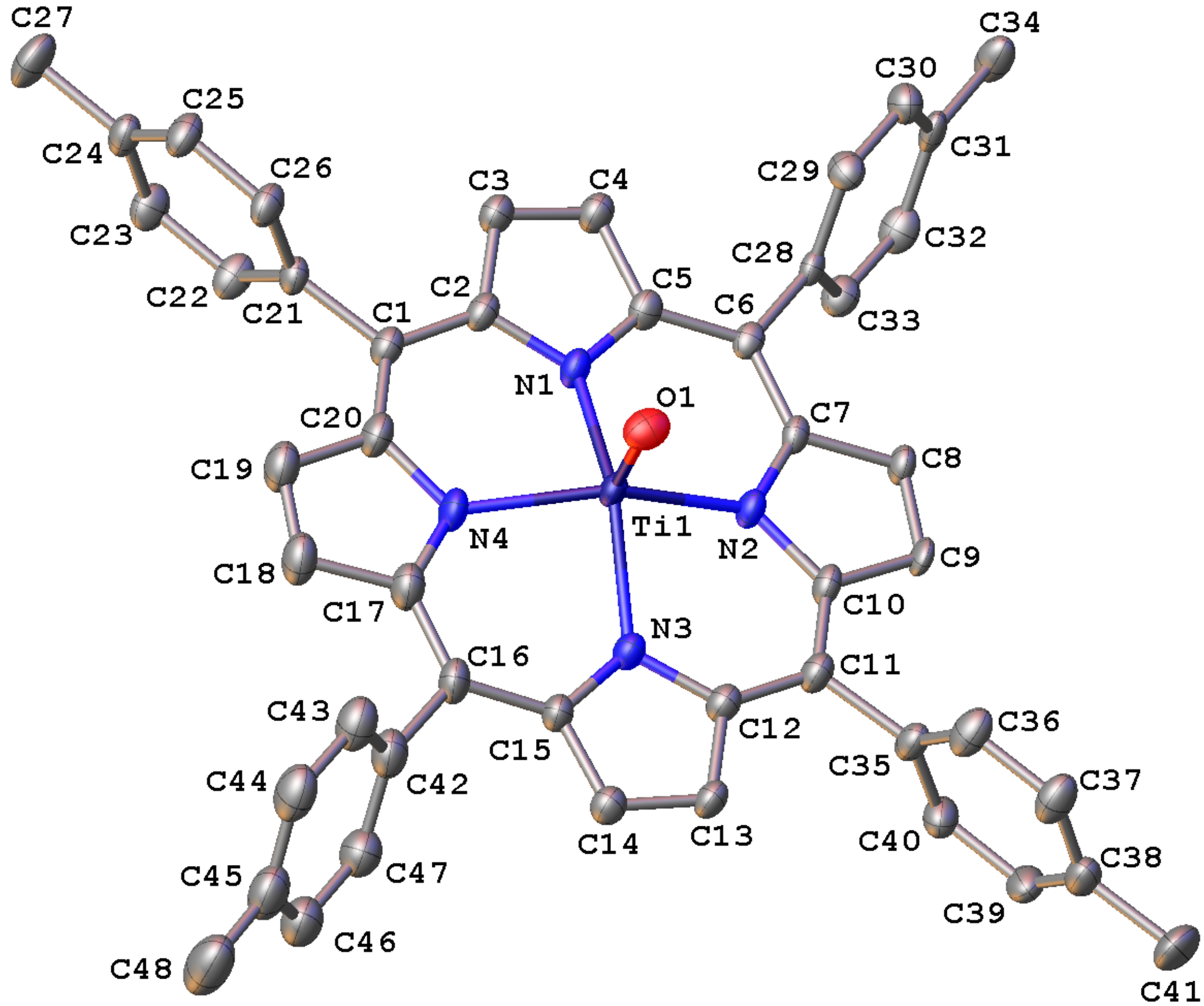

Figure S40. A molecular drawing of (TTP)Ti^{IV}O shown with 50% probability ellipsoids. All H atoms, minor disorder components, and solvent molecules are omitted.

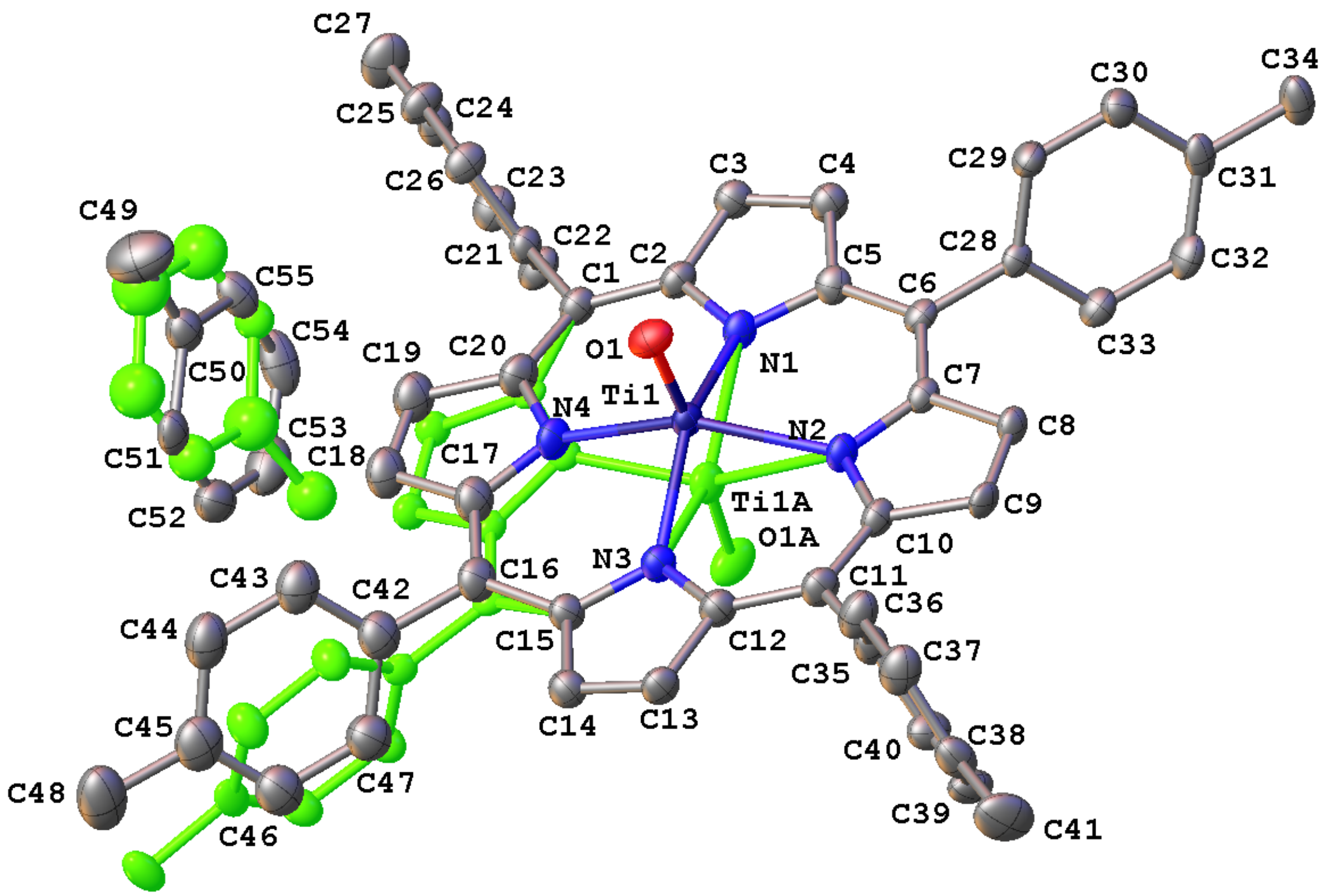

Figure S41. A molecular drawing of (TTP)Ti^{IV}O shown with 50% probability ellipsoids. All H atoms are omitted. The minor disorder components are shown in green; their labels are not shown, but they correspond to the labeled atoms with the suffix 'a' in the tables.

Table S14. Crystal data and structure refinement for (TTP)Ti^{IV}O.

Identification code	Stahl309
Empirical formula	$C_{55}H_{44}N_4OTi$
Formula weight	824.84
Temperature/K	130.00
Crystal system	monoclinic
Space group	$P2_1$
a/Å	14.7501(12)
b/Å	9.5738(7)
c/Å	15.2688(16)
α/°	90
β/°	95.539(7)
γ/°	90
Volume/Å^3	2146.1(3)
Z	2
ρ_{calc}g/cm^3	1.276
μ/mm^{-1}	2.038
F(000)	864.0

Crystal size/mm^3	0.12 × 0.04 × 0.04
Radiation	CuKα (λ = 1.54178)
2Θ range for data collection/°	5.816 to 158.574
Index ranges	-18 ≤ h ≤ 18, -12 ≤ k ≤ 12, -19 ≤ l ≤ 17
Reflections collected	71171
Independent reflections	9177 [R_{int} = 0.0398, R_{sigma} = 0.0234]
Data/restraints/parameters	9177/609/707
Goodness-of-fit on F^2	1.041
Final R indexes [I>=2σ (I)]	R_1 = 0.0443, wR_2 = 0.1248
Final R indexes [all data]	R_1 = 0.0519, wR_2 = 0.1324
Largest diff. peak/hole / e Å^{-3}	0.22/-0.67
Flack parameter	0.091(6)

Table S15. Bond Lengths for (TTP)Ti^{IV}O.

Atom	Atom	Length/Å	Atom	Atom	Length/Å
Ti1	O1	1.641(3)	C32	C33	1.378(4)
Ti1	N1	2.105(3)	C35	C36	1.382(5)
Ti1	N2	2.118(3)	C35	C40	1.389(5)
Ti1	N3	2.113(3)	C36	C37	1.394(5)
Ti1	N4	2.076(14)	C37	C38	1.392(6)

11.2. (TTP)Ti^{IV}(OPh)$_2$

CCDC Deposition Number: 2543660

Data collection

A black crystal with approximate dimensions 0.036 × 0.035 × 0.007 mm^3 was selected under oil under ambient conditions and attached to the tip of a MiTeGen MicroMount©. The crystal was mounted in a stream of cold nitrogen at 100(1) K and centered in the X-ray beam by using a video camera.

The crystal evaluation and data collection were performed on a Bruker D8 VENTURE PhotonIII four-circle diffractometer with Cu Kα (λ = 1.54178 Å) radiation and the detector to crystal distance of 5.0 cm.[42]

The initial cell constants were obtained from a 180° φ scan conducted at a 2θ = 30° angle with the exposure time of 1 second per frame. The reflections were successfully indexed by an automated indexing routine built in the APEX6 program. The final cell constants were calculated from a set of 9878 strong reflections from the actual data collection.

The data were collected by using the half sphere data collection routine to survey the reciprocal space to the extent of a half sphere to a resolution of 0.78 Å. A total of 71539 data were harvested by collecting 18 sets of frames with 0.8º scans in ω and φ with exposure times of 4–40 seconds per frame. These highly redundant datasets were corrected for Lorentz and polarization effects. The absorption

correction was based on fitting a function to the empirical transmission surface as sampled by multiple equivalent measurements.[43]

Structure solution and refinement

The systematic absences in the diffraction data were uniquely consistent for the space group $P2_1/n$ that yielded chemically reasonable and computationally stable results of refinement.[44–49]

A successful solution by intrinsic phasing provided most non-hydrogen atoms from the *E*-map. The remaining non-hydrogen atoms were located in an alternating series of least-squares cycles and difference Fourier maps. All non-hydrogen atoms were refined with anisotropic displacement coefficients, unless otherwise noted. All hydrogen atoms were included in the structure factor calculation at idealized positions and were allowed to ride on the neighboring atoms with relative isotropic displacement coefficients.

The asymmetric unit contains two compositionally disordered Ti compounds. Both Ti compounds are coordinated by a porphyrin, and the major Ti compound is coordinated by two *trans* phenoxy ligands while the minor compound has the Ti coordinated to one oxo ligand. There is also one partially occupied toluene solvent molecule in the asymmetric unit for an overall composition of $[TiC_{48}H_{36}N_4(C_6H_5O)_2]_{0.9183}[TiC_{48}H_{36}N_4O]_{0.0817}\cdot 0.1638(C_7H_8)$.

The Ti atom is positionally disordered over two sites that correspond to the compositional disorder of the axial ligands. The dominant component contains two *trans* phenoxy ligands about Ti1 with an occupation of 91.83(18) %. The minor component contains the T1A to O1A oxo ligand with an occupation of 8.17(18) %. Atomic distance restraints are applied between the Ti and O atoms. Given the low occupation of the minor component, the O1A atom is refined with a constrained isotropic displacement parameter.

The phenyl ring of the O1 phenoxy ligand is disordered over two positions with an occupation ratio of 68.7(3) % / 23.2(3) %. The phenyl ring of the O2 phenoxy ligand is used as a model to restrain the geometry of the disordered O1 phenyl rings which are refined with restrained anisotropic displacement parameters.

The C42, C45–C48 atoms of the toluene substituent on the porphyrin are disordered over two positions with a major component occupation of 53(3) %. The C54–C60 atoms are used as a model to restrain the geometries of the C42–C48 fragments containing both the major and minor component of the disorder which are refined with restrained anisotropic displacement parameters.

There is one partially occupied toluene molecule in the asymmetric unit with an occupation of 16.38(18) %. This toluene is disordered around an inversion center generating two positions for the molecule. The toluene molecule is refined with a constrained geometry and isotropic atomic displacement parameters.[50]

The final least-squares refinement of 732 parameters against 10668 data resulted in residuals *R* (based on F^2 for $I \geq 2\sigma$) and *wR* (based on F^2 for all data) of 0.0471 and 0.1283, respectively. The final difference Fourier map was featureless.

Summary

Crystal Data for $C_{60.165}H_{46.4925}N_4O_{1.9175}Ti$ (M =904.06 g/mol): monoclinic, space group $P2_1/n$ (no. 14), a = 11.4924(15) Å, b = 21.783(2) Å, c = 19.798(3) Å, β = 92.460(9)°, V = 4951.6(11) Å^3, Z = 4, T = 100.00 K, μ(CuKα) = 1.827 mm^{-1}, $Dcalc$ = 1.213 g/cm^3, 70648 reflections measured (8.118° ≤ 2Θ ≤ 158.608°), 10668 unique (R_{int} = 0.0665, R_{sigma} = 0.0402) which were used in all calculations. The final R_1 was 0.0471 (I > 2σ(I)) and wR_2 was 0.1283 (all data).

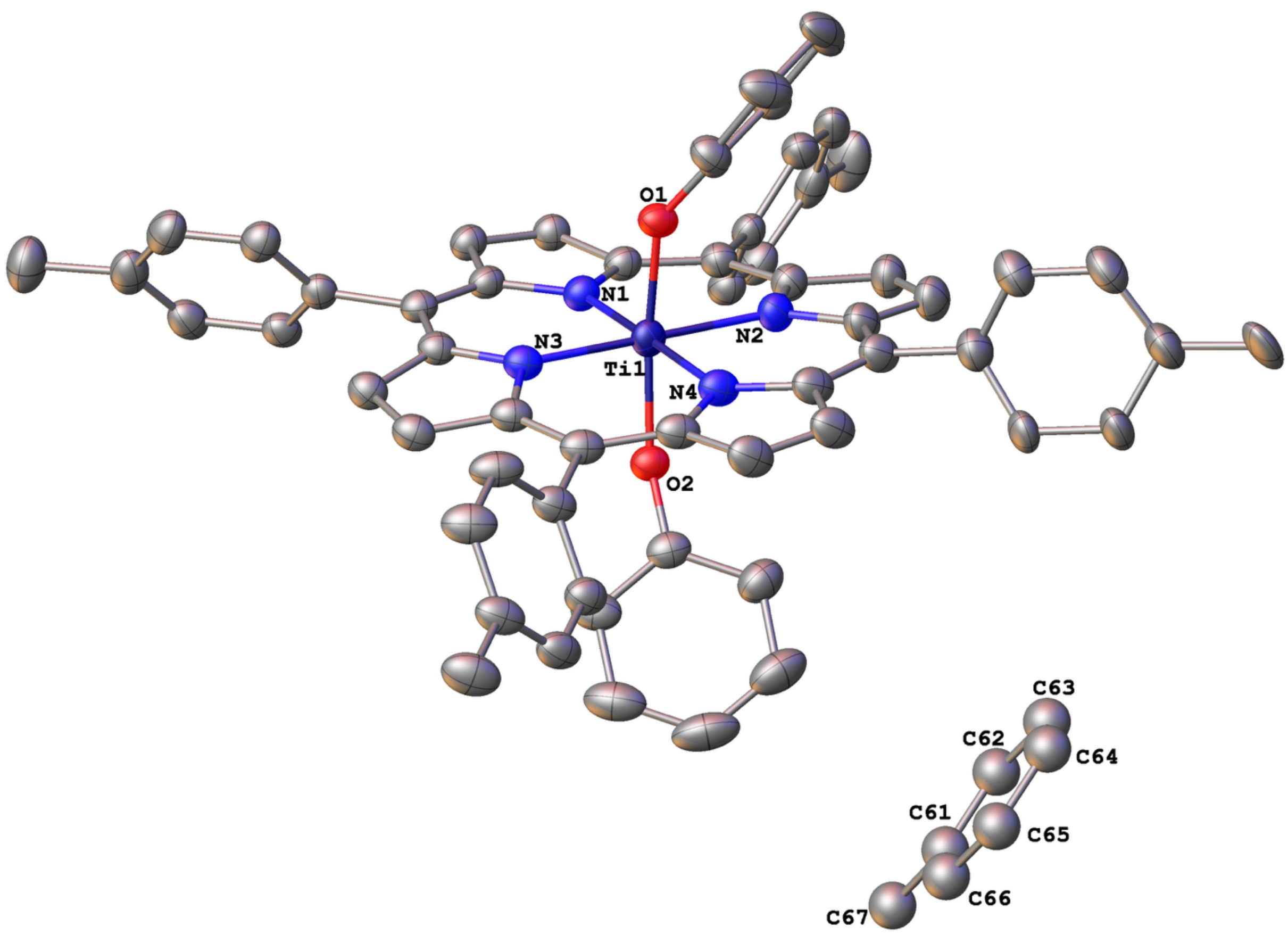

Figure S42. A molecular drawing of (TTP)Ti^{IV}(OPh)$_2$ shown with 50% probability ellipsoids. Labels are shown for the non-carbon atoms and the toluene solvent molecule. All H atoms and minor components of disorder are omitted.

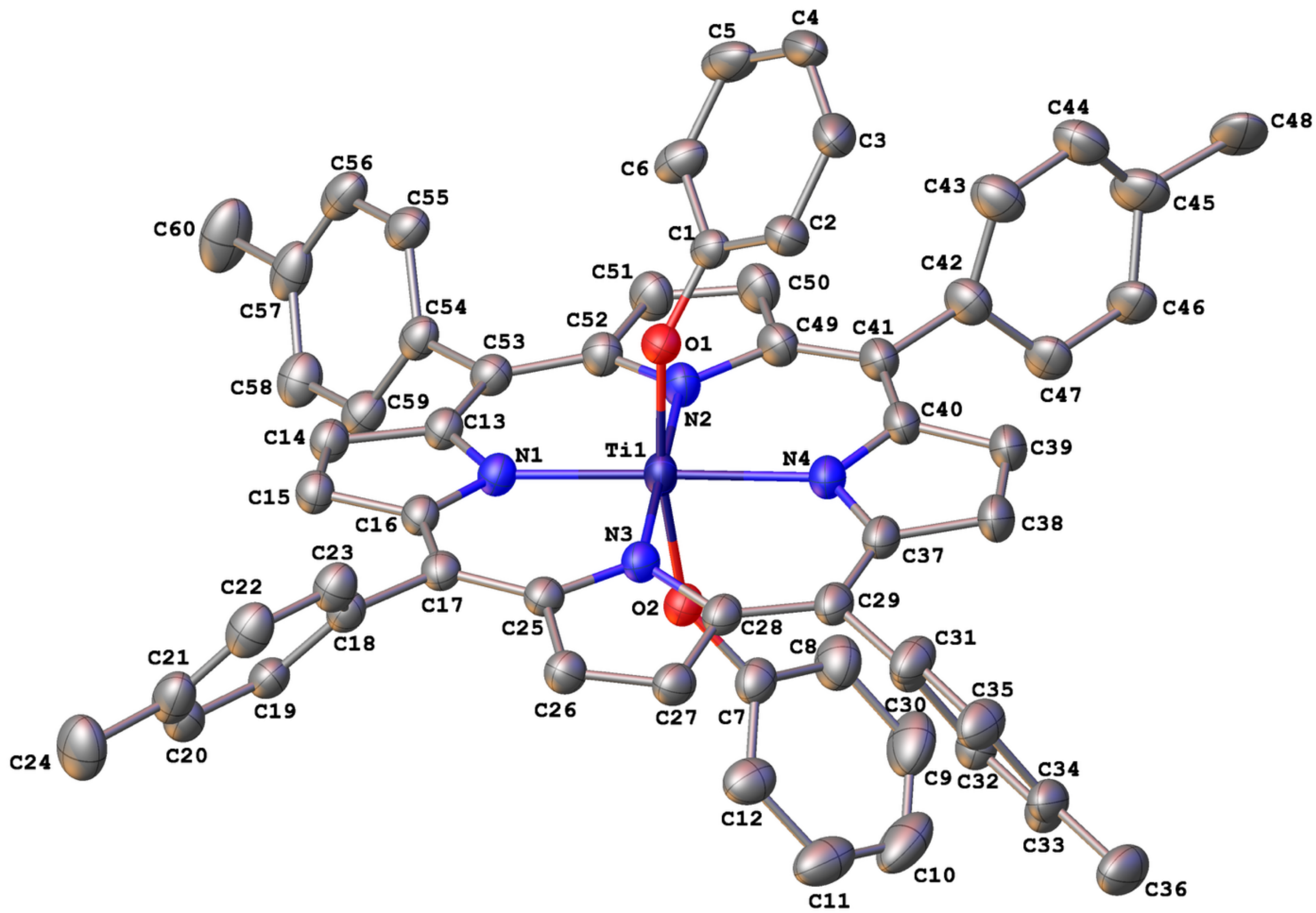

Figure S43. A molecular drawing of the major Ti1 compound in (TTP)Ti^{IV}(OPh)$_2$ shown with 50% probability ellipsoids. All H atoms, minor components of disorder, and solvent molecules are omitted.

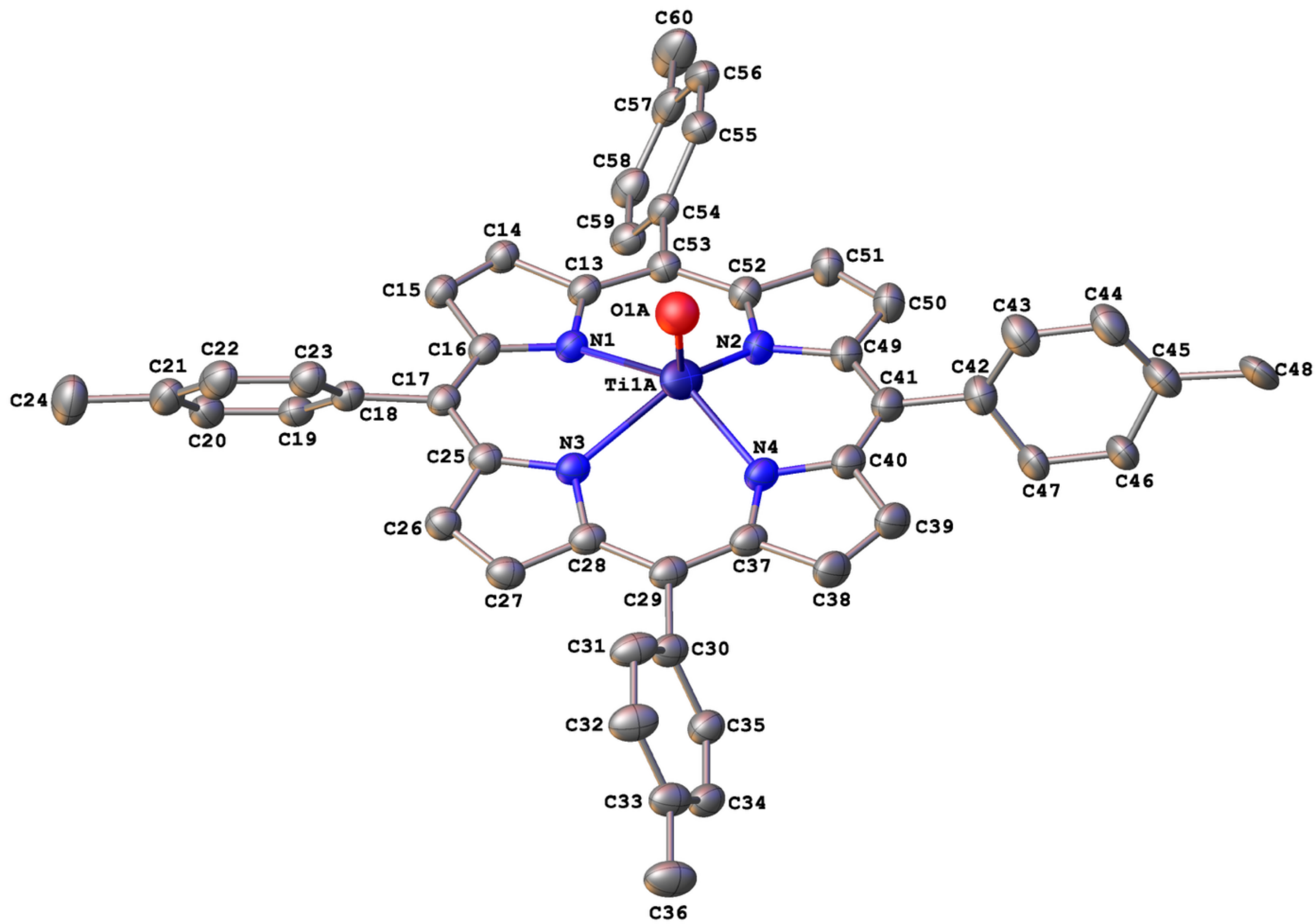

Figure S44. A molecular drawing of the minor Ti1A compound in (TTP)$Ti^{IV}(OPh)_2$ shown with 50% probability ellipsoids. All H atoms, minor components of disorder, and solvent molecules are omitted.

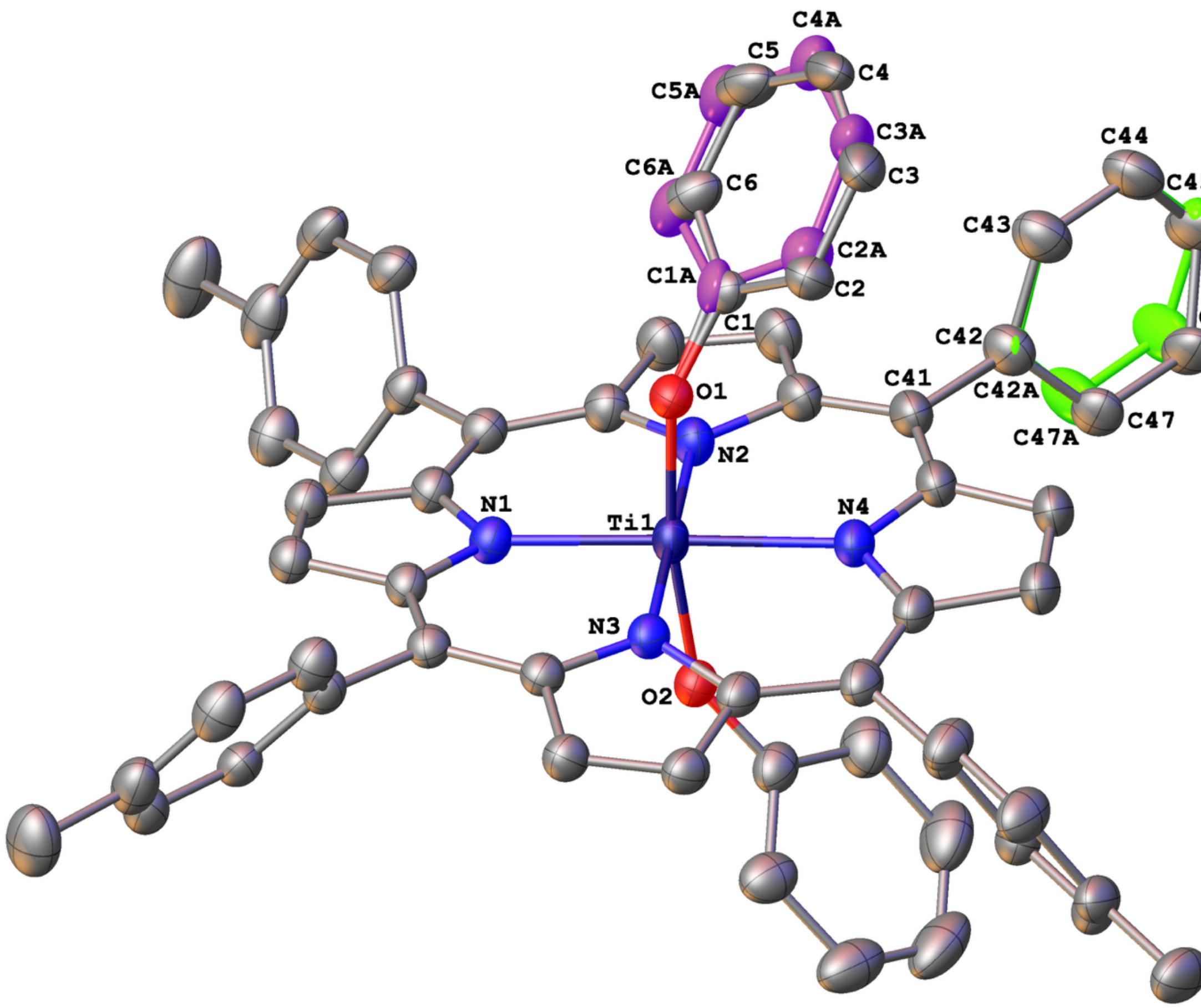

Figure S45. A molecular drawing of the major Ti1 compound in (TTP)Ti^{IV}(OPh)$_2$ highlighting the positionally disordered C atoms. The C1A–C6A and C42A, C45A–C48A minor components of disorder are drawn in purple and green, respectively. Atoms are drawn with 50% probability ellipsoids and all H atoms and solvent molecules are omitted.

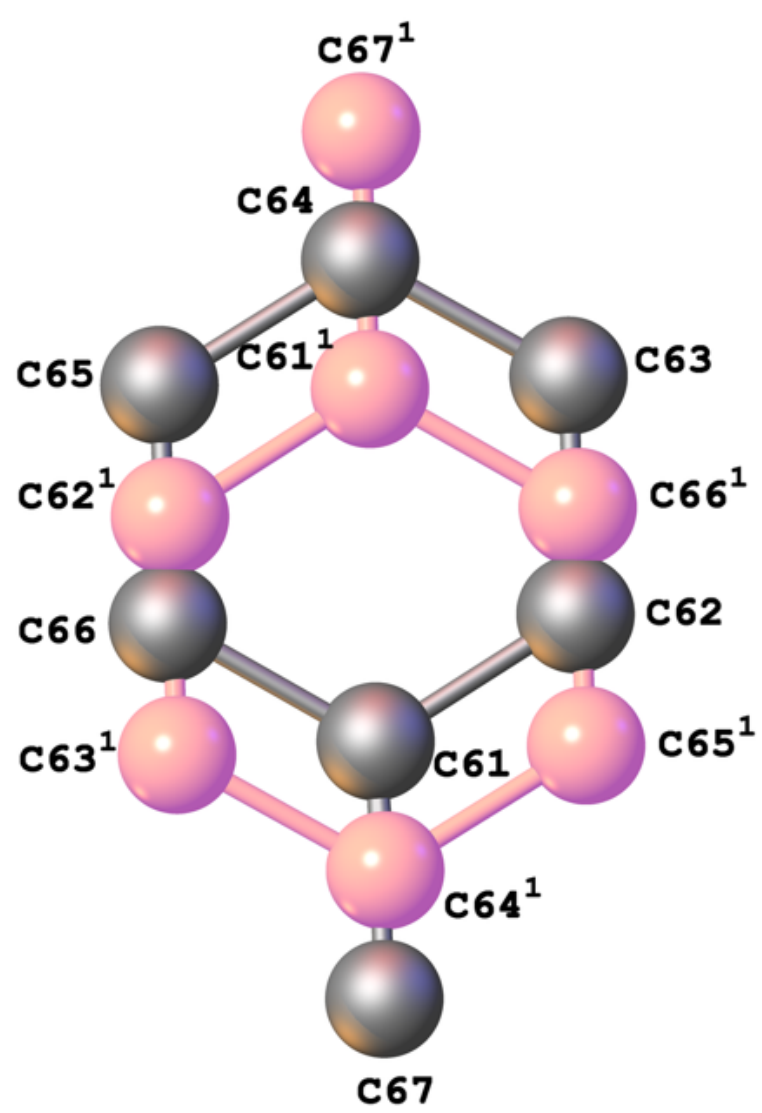

Figure S46. A molecular drawing of the toluene disorder about the inversion center in (TTP)$Ti^{IV}(OPh)_2$. The two symmetry related toluene molecules are drawn in grey and pink. All H atoms are omitted. Symmetry code: (1) 1-X,1-Y,2-Z.

Table S16. Crystal data and structure refinement for (TTP)$Ti^{IV}(OPh)_2$.

Identification code	Stahl313
Empirical formula	$[TiC_{48}H_{36}N_4 (C_6H_5O)_2]_{0.9183}[TiC_{48}H_{36}N_4O]_{0.0817}$•$0.1638(C_7H_8)$
Formula weight	904.06
Temperature/K	100.00
Crystal system	monoclinic
Space group	$P2_1/n$
a/Å	11.4924(15)
b/Å	21.783(2)
c/Å	19.798(3)
α/°	90
β/°	92.460(9)
γ/°	90
Volume/Å^3	4951.6(11)
Z	4
ρ_{calc}g/cm^3	1.213
μ/mm^{-1}	1.827
F(000)	1891.0
Crystal size/mm^3	0.036 × 0.035 × 0.007
Radiation	CuKα (λ = 1.54178)
2Θ range for data collection/°	8.118 to 158.608
Index ranges	$-14 \le h \le 14$, $-26 \le k \le 27$, $-25 \le l \le 20$
Reflections collected	70648
Independent reflections	10668 [R_{int} = 0.0665, R_{sigma} = 0.0402]
Data/restraints/parameters	10668/692/732
Goodness-of-fit on F^2	1.055
Final R indexes [I>=2σ (I)]	R_1 = 0.0471, wR_2 = 0.1203
Final R indexes [all data]	R_1 = 0.0598, wR_2 = 0.1283
Largest diff. peak/hole / e Å^{-3}	0.38/-0.40

Table S17. Bond Lengths for (TTP)$Ti^{IV}(OPh)_2$ (**[$Ti^{IV}(OPh)_2$]**).

Atom	Atom	Length/Å	Atom	Atom	Length/Å
Ti1	O1	1.8559(15)	C22	C23	1.392(3)
Ti1	O2	1.8772(14)	C25	C26	1.442(2)
Ti1	N1	2.0536(15)	C26	C27	1.359(3)
Ti1	N2	2.0843(16)	C27	C28	1.438(3)
Ti1	N3	2.0560(16)	C28	C29	1.411(2)
Ti1	N4	2.0810(15)	C29	C30	1.498(2)

11.3. (TTP)Ti^{III}Cl

CCDC Deposition Number: 2560731

Data collection

A dark green crystal with approximate dimensions 0.111 × 0.047 × 0.031 mm^3 was selected under oil under ambient conditions and attached to the tip of a MiTeGen MicroMount©. The crystal was mounted in a stream of cold nitrogen at 100(1) K and centered in the X-ray beam by using a video camera.

The crystal evaluation and data collection were performed on a Bruker D8 VENTURE Photon III four-circle diffractometer with Diamond II IμS Mo Kα (λ = 0.71073 Å) radiation and the detector to crystal distance of 5.0 cm.[42]

The initial cell constants were obtained from a 180° φ scan conducted at a 2θ = 30° angle with the exposure time of 2 seconds per frame. The reflections were successfully indexed by an automated indexing routine built in the APEX6 program. The final cell constants were calculated from a set of 9980 strong reflections from the actual data collection.

The data were collected by using the half sphere data collection routine to survey the reciprocal space to the extent of a half sphere to a resolution of 0.75 Å. A total of 162390 data were harvested by collecting 9 sets of frames with 0.7º scans in ω and φ with an exposure time of 1–39.5 seconds per frame. These highly redundant datasets were corrected for Lorentz and polarization effects. The absorption correction was based on fitting a function to the empirical transmission surface as sampled by multiple equivalent measurements.[43]

Structure solution and refinement

The systematic absences in the diffraction data were consistent for the space groups *P*2/*n* and *Pn*.[44–49] The *E*-statistics suggested the centrosymmetric space group *P*2/*n*; however, this space group did not yield a chemically reasonable solution. The space group *Pn* led to a computationally stable and chemically reasonable refinement.

A successful solution by intrinsic phasing provided most non-hydrogen atoms from the *E*-map. The remaining non-hydrogen atoms were located in an alternating series of least-squares cycles and difference Fourier maps. All non-hydrogen atoms were refined with anisotropic atomic displacement coefficients. All hydrogen atoms were included in the structure factor calculation at idealized positions and were allowed to ride on the neighboring atoms with relative isotropic atomic displacement coefficients.

The asymmetric unit contains two symmetry-independent $TiC_{48}H_{36}N_4Cl$ molecules and three solvent molecules. The two $TiC_{48}H_{36}N_4Cl$ molecules have identical compositions and similar conformations. There are also three solvent accessible voids containing electron density in the asymmetric

unit. Two toluene molecules occupy the first two voids, and the third void contains a mix of positionally disordered THF and toluene molecules. The overall chemical formula is $TiC_{48}H_{36}N_4Cl{\cdot}(C_7H_8)_{1.15}{\cdot}(THF)_{0.35}$.

The compound crystallizes as a racemic twin with an 89(3) % contribution from the dominant component. Additional analysis suggest that the diffraction data might be consistent with the centrosymmetric space group $P2_1/n$; however, the reflections that should be extinct for the 2_1 screw axis (*0k0*, *k*=odd), although weaker, still had observable intensities. The structure can be solved in the space group $P2_1/n$; however, the residuals, *R* and *wR*, remained high and there is a significantly disordered tolyl group in the $TiC_{48}H_{36}N_4Cl$ molecule that is not present in the two $TiC_{48}H_{36}N_4Cl$ molecules of the lower symmetry *Pn* structure. In addition, if the structure were truly centrosymmetric the refinement of the Flack *x* parameter in the space group *Pn* should yield a value of 0.5, indicating an equal contribution from both components. In this structure, the Flack *x* parameter is only 11(3) % confirming the non-centrosymmetric space group *Pn*. The racemic twinning may contribute to the skewed *E*-statistics that suggest a centrosymmetric space group.

The absolute structure was established by resonant scattering effects.

Atomic distance restraints are applied to various C–C bond distances within the tolyl groups of the $TiC_{48}H_{36}N_4Cl$ molecules. The N4, C37, and C40 atoms are refined with restrained anisotropic atomic displacement coefficients.

The C49 and C56 toluene solvent molecules are refined with constrained geometries and restrained anisotropic atomic displacement coefficients.[50]

There are three solvent accessible voids containing electron density. The C49 and C56 toluene solvent molecules occupy two of these voids, and they are refined as previously described. The third void contains a mix of positionally disordered toluene and THF molecules. A significant amount of time was invested in identifying and refining the disordered molecules. Bond length restraints and constraints were applied to model the molecules, but the resulting isotropic atomic displacement coefficients suggested the molecules were mobile. In addition, the refinement was computationally unstable. The OLEX2.Mask was used to correct the diffraction data for diffuse scattering effects and to identify the solvent molecules. OLEX2 calculated the upper limit of volume that can be occupied by the solvent to be 434 $Å^3$, or 9.4 % of the unit cell volume. The program calculated 44 electrons in the unit cell for the diffuse species. This approximately corresponds with 0.3 of a toluene molecule and 0.7 of a THF molecule in the asymmetric unit (43 electrons). Please note that all derived results in the following tables are based on the known contents. No data are given for the diffusely scattering species.

The final least-squares refinement of 1080 parameters against 22934 data resulted in residuals *R* (based on F^2 for $I \geq 2\sigma$) and *wR* (based on F^2 for all data) of 0.0476 and 0.1341, respectively. The final difference Fourier map was featureless.

Summary

Crystal Data for $C_{60.165}H_{46.4925}N_4O_{1.9175}Ti$ (M =904.06 g/mol): monoclinic, space group $P2_1/n$ (no. 14), a = 11.4924(15) Å, b = 21.783(2) Å, c = 19.798(3) Å, β = 92.460(9)°, V = 4951.6(11) Å^3, Z = 4, T = 100.00 K, μ(CuKα) = 1.827 mm^{-1}, $Dcalc$ = 1.213 g/cm^3, 70648 reflections measured (8.118° ≤ 2Θ ≤ 158.608°), 10668 unique (R_{int} = 0.0665, R_{sigma} = 0.0402) which were used in all calculations. The final R_1 was 0.0471 (I > 2σ(I)) and wR_2 was 0.1283 (all data).

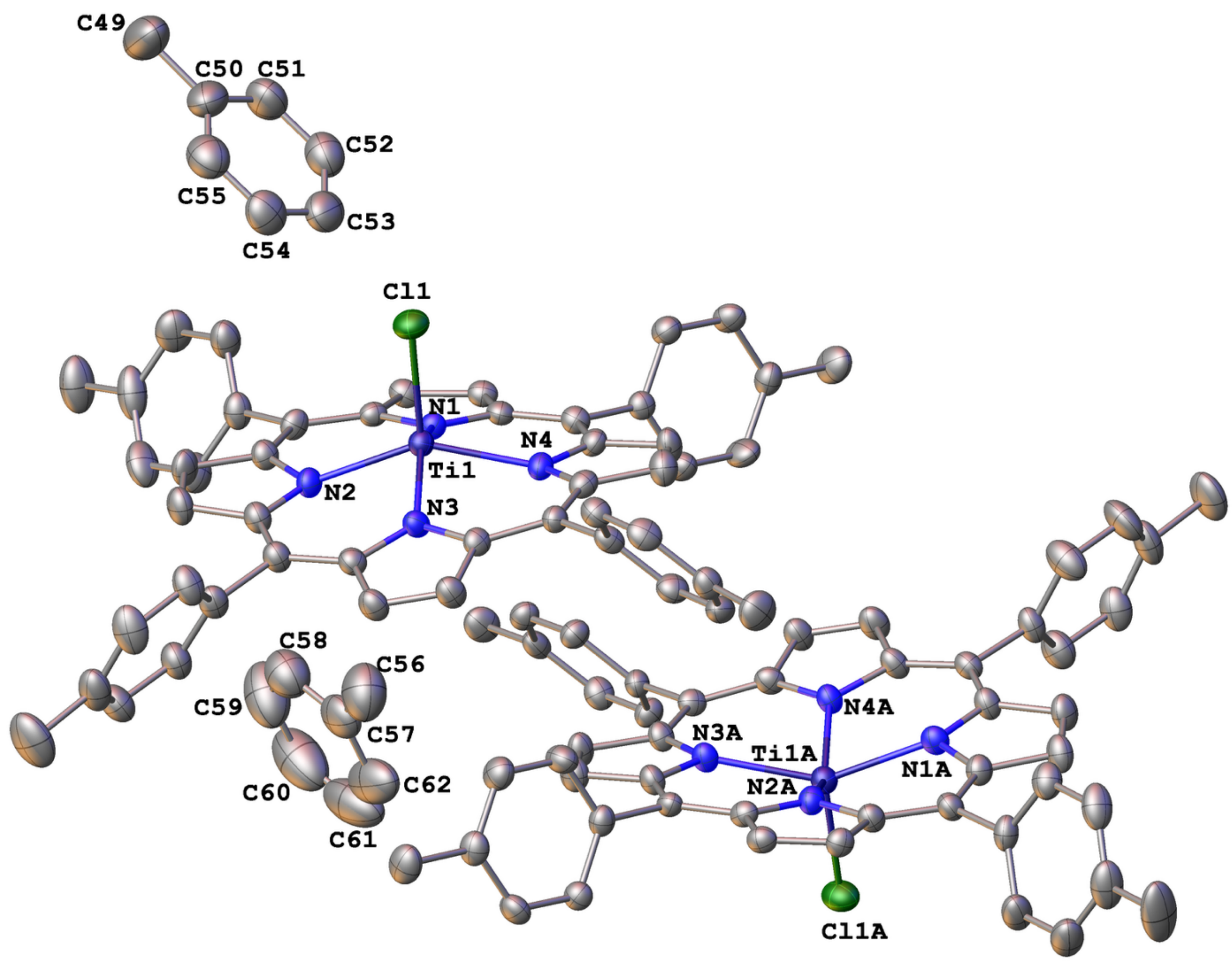

Figure S47. A molecular drawing of (TTP)Ti^{III}Cl shown with 50% probability ellipsoids. All H atoms and C atom labels in the $TiC_{48}H_{36}N_4Cl$ molecules are omitted.

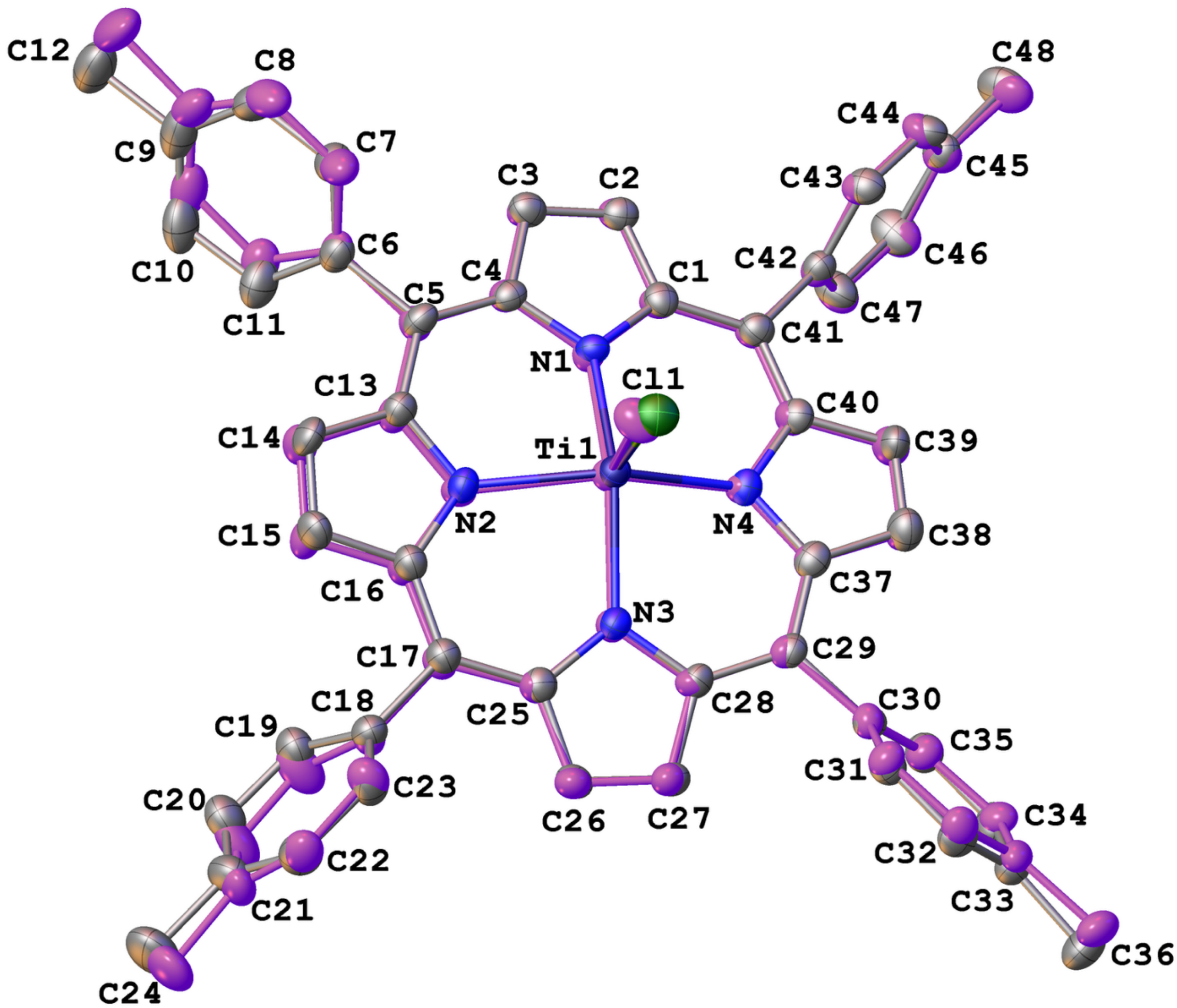

Figure S48. A drawing of the two symmetry-independent $TiC_{48}H_{36}N_4Cl$ molecules in (TTP)Ti^{III}Cl overlayed. The H atoms, the toluene solvent molecules, and the second $TiC_{48}H_{36}N_4Cl$ molecule labels are omitted.

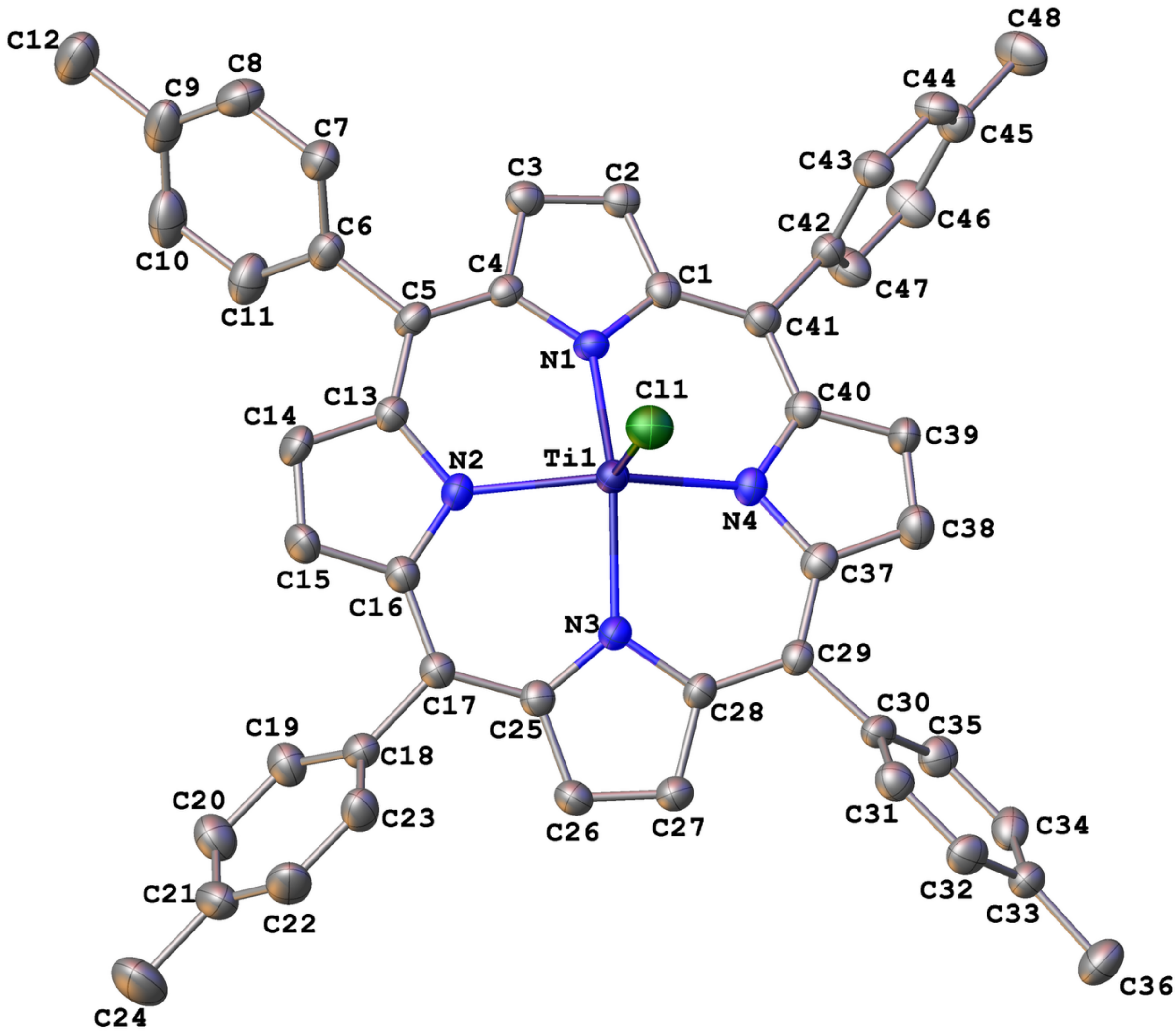

Figure S49. A drawing of one of the symmetry-independent $TiC_{48}H_{36}N_4Cl$ molecules in (TTP)Ti^{III}Cl. The H atoms, the toluene solvent molecules, and the second $TiC_{48}H_{36}N_4Cl$ molecule are omitted.

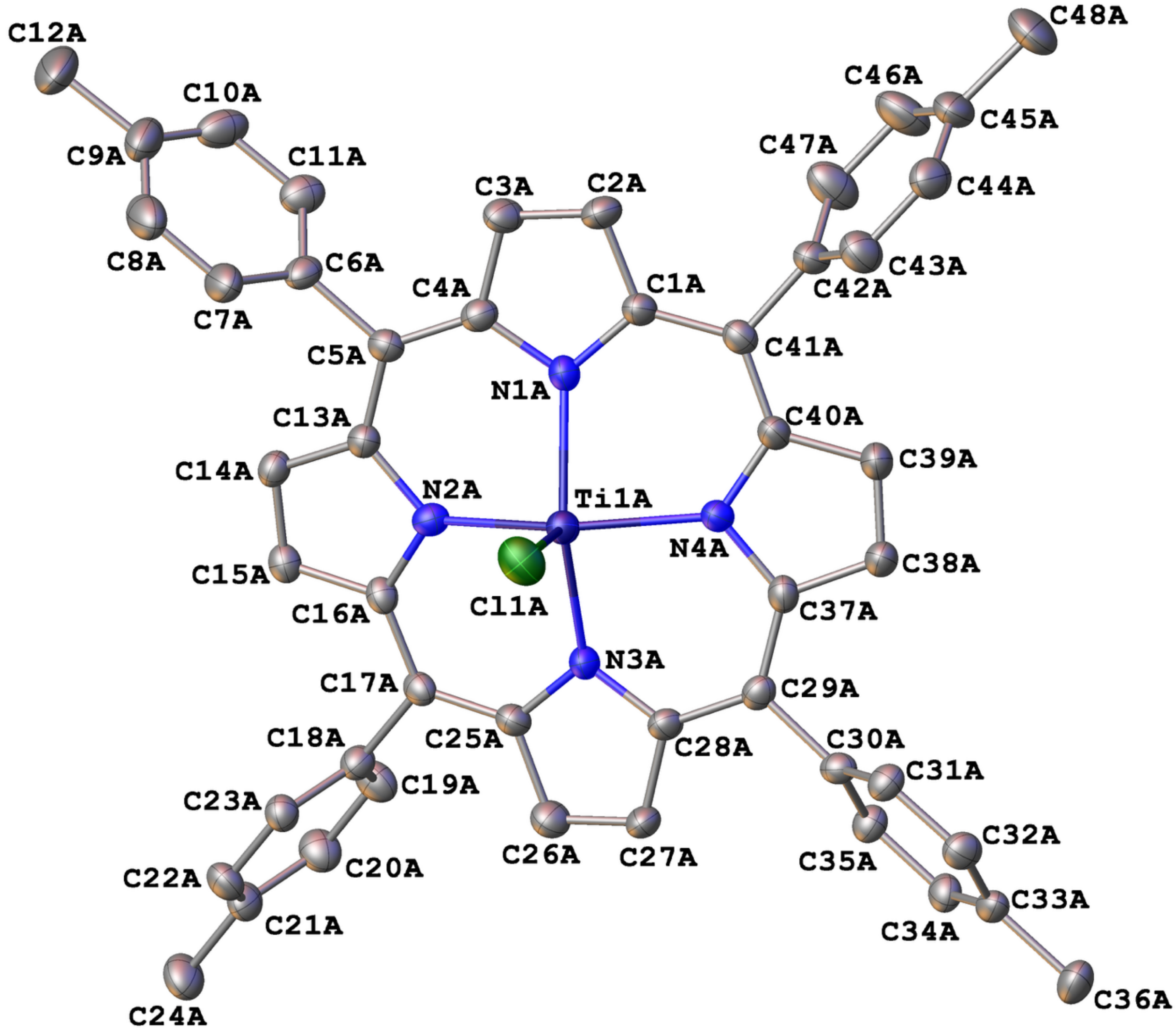

Figure S50. A drawing of the second symmetry-independent $TiC_{48}H_{36}N_4Cl$ molecule in (TTP)Ti^{III}Cl. The H atoms, the toluene solvent molecules, and the first $TiC_{48}H_{36}N_4Cl$ molecule are omitted.

Table S18. Crystal data and structure refinement for (TTP)Ti^{III}Cl.

Identification code	Stahl335
Empirical formula	$TiC_{48}H_{36}N_4Cl\cdot(C_7H_8)_{1.15}\cdot(THF)_{0.35}$
([Formula weight	883.35
Temperature/K	100.00
Crystal system	monoclinic
Space group	*Pn*
a/Å	10.860(3)
b/Å	14.778(3)
c/Å	28.869(8)
α/°	90
β/°	93.245(7)

γ/°	90
Volume/Å^3	4626(2)
Z	4
ρ_{calc}g/cm^3	1.268
μ/mm^{-1}	0.287
F(000)	1850.0
Crystal size/mm^3	0.111 × 0.047 × 0.031
Radiation	Mo Kα (λ = 0.71073)
2Θ range for data collection/°	3.938 to 56.584
Index ranges	$-14 \le h \le 14$, $-18 \le k \le 19$, $-38 \le l \le 38$
Reflections collected	158683
Independent reflections	22934 [R_{int} = 0.0532, R_{sigma} = 0.0388]
Data/restraints/parameters	22934/632/1080
Goodness-of-fit on F^2	1.029
Final R indexes [I>=2σ (I)]	R_1 = 0.0476, wR_2 = 0.1232
Final R indexes [all data]	R_1 = 0.0649, wR_2 = 0.1341
Largest diff. peak/hole / e Å^{-3}	0.65/-0.25
Flack parameter	0.11(3)

Table S19. Bond Lengths for (TTP)Ti^{III}Cl.

Atom	Atom	Length/Å	Atom	Atom	Length/Å
Ti1	Cl1	2.2564(14)	N2A	C13A	1.401(6)
Ti1	N1	2.115(3)	N2A	C16A	1.382(5)
Ti1	N2	2.105(3)	N3A	C25A	1.383(5)
Ti1	N3	2.109(4)	N3A	C28A	1.389(5)
Ti1	N4	2.098(4)	N4A	C37A	1.384(5)

11.4. (TTP)Ti^{II}(DMA)$_2$

CCDC Deposition Number: 2543658

Data collection

A brown crystal with approximate dimensions 0.12 × 0.08 × 0.07 mm^3 was selected under oil under ambient conditions and attached to the tip of a MiTeGen MicroMount©. The crystal was mounted in a stream of cold nitrogen at 100(1) K and centered in the X-ray beam by using a video camera.

The crystal evaluation and data collection were performed on a Bruker D8 VENTURE PhotonIII four-circle diffractometer with Cu Kα (λ = 1.54178 Å) radiation and the detector to crystal distance of 5.0 cm.[42]

The initial cell constants were obtained from a 180° φ scan conducted at a 2θ = 50° angle with the exposure time of 1 second per frame. The reflections were successfully indexed by an automated indexing

routine built in the APEX6 program. The final cell constants were calculated from a set of 9818 strong reflections from the actual data collection.

The data were collected by using the full sphere data collection routine to survey the reciprocal space to the extent of a full sphere to a resolution of 0.78 Å. A total of 104803 data were harvested by collecting 50 sets of frames with 0.5º scans in ω and φ with an exposure time 1–15 seconds per frame. These highly redundant datasets were corrected for Lorentz and polarization effects. The absorption correction was based on fitting a function to the empirical transmission surface as sampled by multiple equivalent measurements.[43]

Structure solution and refinement

The systematic absences in the diffraction data were consistent for the space groups *Ia* and *I*2/*a*. The *E*-statistics strongly suggested the centrosymmetric space group *I2/a* that yielded chemically reasonable and computationally stable results of refinement.[44–49]

A successful solution by intrinsic phasing provided most non-hydrogen atoms from the *E*-map. The remaining non-hydrogen atoms were located in an alternating series of least-squares cycles and difference Fourier maps. All non-hydrogen atoms were refined with anisotropic displacement coefficients. All hydrogen atoms were included in the structure factor calculation at idealized positions and were allowed to ride on the neighboring atoms with relative isotropic displacement coefficients.

The composition of the system is $(C_{56}H_{54}N_6O_2Ti)\cdot0.8C_5H_{12}\cdot0.2C_4H_8O$. The Ti complex resides on crystallographic inversion center; thus, only one half of the complex is symmetry-independent.

Each DMA ligand in the Ti complex is disordered over two positions with the major component contribution of 0.800(4). The disordered moieties were refined with restraints.

The solvent molecules presented a problem: whereas it was possible to identify a partially occupied pentane residing on a crystallographic 2-fold axis, there were additional peaks of electron density at the same site. The chemist indicated that only THF could be present in addition to pentane, but no successful partially occupied THF model could be refined. Bond length restraints[50] were applied to model the molecules but the resulting isotropic displacement coefficients suggested the molecules were mobile. In addition, the refinement was computationally unstable. Option SQUEEZE of program PLATON[51] was used to correct the diffraction data for diffuse scattering effects and to identify the solvate molecule. PLATON calculated the upper limit of volume that can be occupied by the solvent to be 648 $Å^3$, or 12.8% of the unit cell volume. The program calculated 165 electrons in the unit cell for the diffuse species. This approximately corresponds to 3.2 C_5H_{12} and 0.8 C_4H_8O molecules in the unit cell (166.4 electrons). Please note that all derived results in the following tables are based on the known contents. No data are given for the solvent molecules.

The final least-squares refinement of 343 parameters against 5439 data resulted in residuals *R* (based on F^2 for $I \geq 2\sigma$) and *wR* (based on F^2 for all data) of 0.0420 and 0.1271, respectively. The final difference Fourier map was featureless.

Summary

Crystal Data for $C_{60.8}H_{65.2}N_6O_{2.2}Ti$ (*M* =963.09 g/mol): monoclinic, space group *I*2/*a* (no. 15), *a* = 18.571(2) Å, *b* = 13.6048(10) Å, *c* = 19.9956(12) Å, *β* = 92.617(4)°, *V* = 5046.8(8) Å^3, *Z* = 4, *T* = 100.00 K, μ(CuKα) = 1.832 mm^{-1}, *Dcalc* = 1.268 g/cm^3, 104803 reflections measured (7.862° ≤ 2Θ ≤ 158.476°), 5439 unique (R_{int} = 0.0397, R_{sigma} = 0.0162) which were used in all calculations. The final R_1 was 0.0420 ($I > 2\sigma(I)$) and wR_2 was 0.1271 (all data).

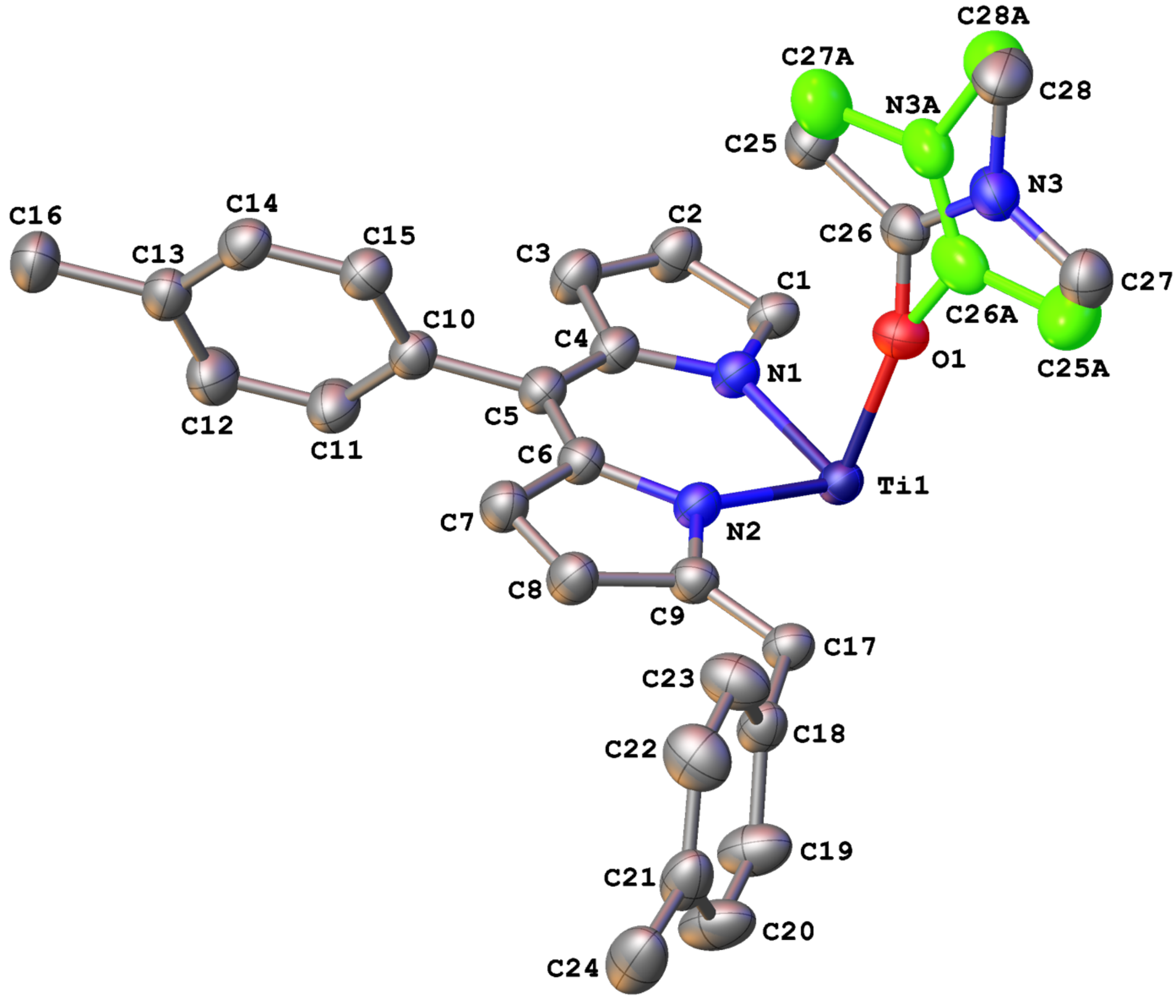

Figure S51. A molecular drawing of symmetry-independent portion of (TTP)Ti^{II}(DMA)$_2$ shown with 50% probability ellipsoids. The complex resides on a crystallographic inversion center and only one half of the molecule is symmetry-independent (shown). The minor component of the DMA disorder is shown in green. All H atoms are omitted.

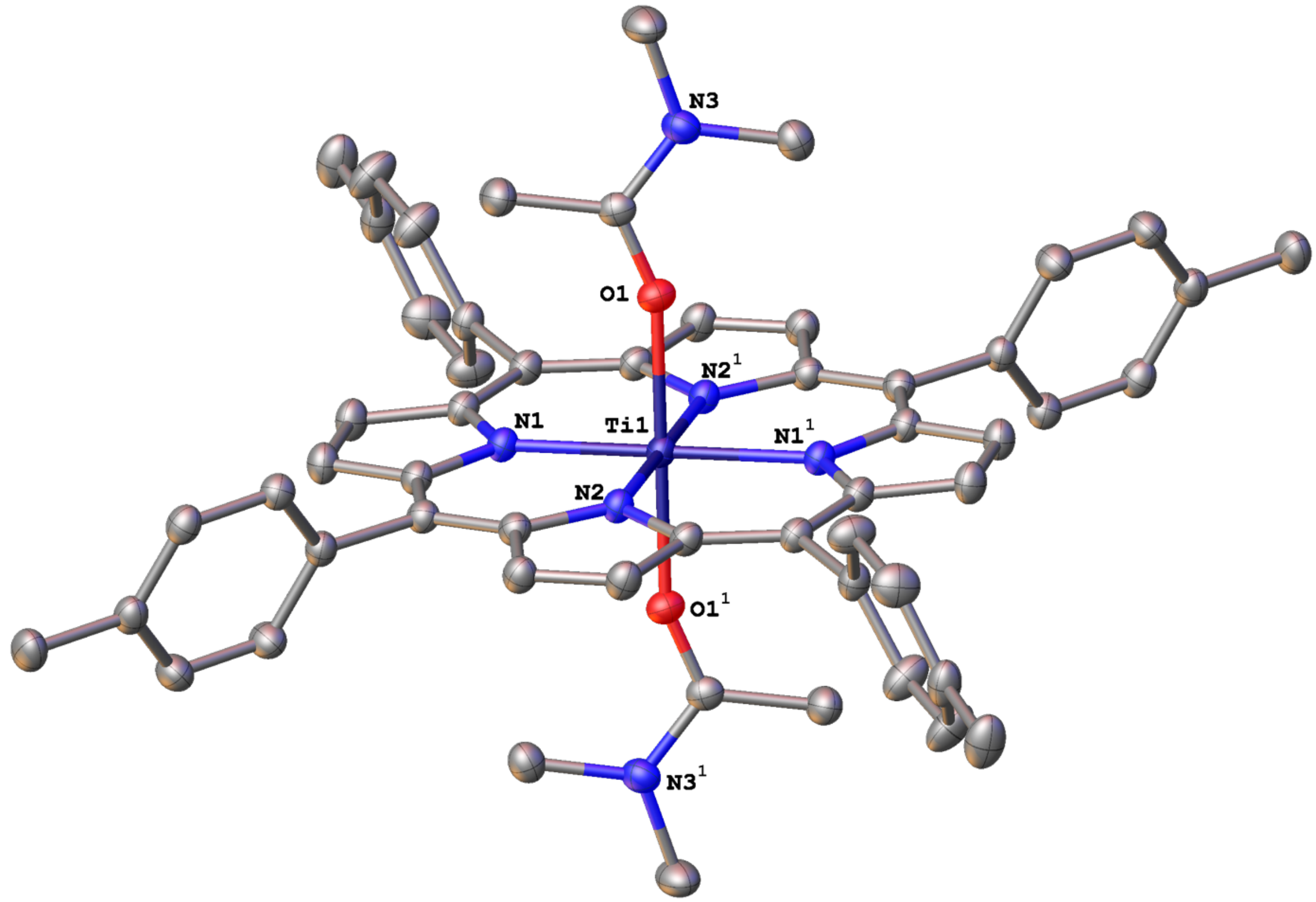

Figure S52. A molecular drawing of (TTP)Ti^{II}(DMA)$_2$_sq shown with 50% probability ellipsoids. All H atoms and minor disorder components are omitted. The solvent molecules cannot be shown (see text). Symmetry code: (1) 1-x, 1-y, 1-z.

Table S20. Crystal data and structure refinement for (TTP)Ti^{II}(DMA)$_2$.

Identification code	stahl308i_sq
Empirical formula	$C_{56}H_{54}N_6O_2Ti \cdot 0.8C_5H_{12} \cdot 0.2THF$
Formula weight	963.09
Temperature/K	100.00
Crystal system	monoclinic
Space group	I2/a
a/Å	18.571(2)
b/Å	13.6048(10)
c/Å	19.9956(12)
α/°	90
β/°	92.617(4)
γ/°	90
Volume/Å^3	5046.8(8)
Z	4
ρ_{calc}g/cm^3	1.268
μ/mm^{-1}	1.832
F(000)	2046.0

Crystal size/mm^3	0.12 × 0.08 × 0.07
Radiation	CuKα (λ = 1.54178)
2Θ range for data collection/°	7.862 to 158.476
Index ranges	-23 ≤ h ≤ 22, -17 ≤ k ≤ 17, -25 ≤ l ≤ 25
Reflections collected	104803
Independent reflections	5439 [R_{int} = 0.0397, R_{sigma} = 0.0162]
Data/restraints/parameters	5439/59/343
Goodness-of-fit on F^2	1.054
Final R indexes [I>=2σ (I)]	R_1 = 0.0420, wR_2 = 0.1244
Final R indexes [all data]	R_1 = 0.0440, wR_2 = 0.1271
Largest diff. peak/hole / e Å^{-3}	0.51/-0.25

Table S21. Bond Lengths for (TTP)$Ti^{II}(DMA)_2$.

Atom	Atom	Length/Å	Atom	Atom	Length/Å
Ti1	N1^1	2.0704(11)	C11	C12	1.382(2)
Ti1	N1	2.0704(11)	C12	C13	1.386(2)
Ti1	N2	2.0548(12)	C13	C14	1.383(2)
Ti1	N2^1	2.0548(12)	C13	C16	1.509(2)
Ti1	O1	2.0499(10)	C14	C15	1.393(2)
Ti1	O1^1	2.0499(10)	C17	C18	1.4966(19)

12. References

(1) *Fine Extruded Graphite Rod, 0.25"OD x 12"L*. https://www.graphitestore.com/fine-extruded-graphite-rod-0-25od-x-12l-nc001310 (accessed 2025-01-20).

(2) *Magnesium Alloy Sheet AZ31B Plate Silver 1 x 100 x 100mm*. https://www.amazon.com/Yodaoke-Magnesium-Alloy-Sheet-Silver/dp/B083RLH984.

(3) *1.6mm Thick Nickel Foam*. https://mtixtl.com/products/bcnf-16m.

(4) *Non-Aqueous Reference Electrode*. https://www.sigmaaldrich.com/US/en/product/aldrich/basmw1085.

(5) Arnold, J. The First Structurally Characterized Alkali Metal Porphyrin: 7Li NMR Behaviour and X-Ray Crystal Structure of the Dilithiurn Salt of Octaethylporphyrin(2-). *J. Chem. Soc. Chem. Commun.* **1990**, 976–978.

(6) Berreau, L. M.; Hays, J. A.; Young, V. G.; Woo, L. K. Synthesis of Early Transition Metal Porphyrin Halide Complexes: First Structural Characterization of a Vanadium(III) Porphyrin Complex. *Inorg. Chem.* **1994**, *33* (1), 105–108. https://doi.org/10.1021/ic00079a020.

(7) Belokon', Y. N.; Caveda-Cepas, S.; Green, B.; Ikonnikov, N. S.; Khrustalev, V. N.; Larichev, V. S.; Moscalenko, M. A.; North, M.; Orizu, C.; Tararov, V. I.; Tasinazzo, M.; Timofeeva, G. I.; Yashkina, L. V. The Asymmetric Addition of Trimethylsilyl Cyanide to Aldehydes Catalyzed by Chiral (Salen)Titanium Complexes. *J. Am. Chem. Soc.* **1999**, *121* (16), 3968–3973. https://doi.org/10.1021/ja984197v.

(8) Stolzenberg, A. M.; Haymond, G. S. Activation Barriers to *Meso* -Aryl Group Rotation in Titanyl Tetraaryltetrapyrroles. An Investigation of the Out-of-Plane Flexibility of Hydroporphyrins. *Inorg. Chem.* **2002**, *41* (2), 300–308. https://doi.org/10.1021/ic010936o.

(9) Gray, S. D.; Thorman, J. L.; Berreau, L. M.; Woo, L. K. Alkoxido, Amido, and Imido Derivatives of Titanium(IV) Tetratolylporphyrin. *Inorg. Chem.* **1997**, *36* (3), 278–283. https://doi.org/10.1021/ic960977y.

(10) Wang, X.; Gray, S. D.; Chen, J.; Woo, L. K. Facile Syntheses of Titanium(II), Tin(II), and Vanadium(II) Porphyrin Complexes through Homogeneous Reduction. Reactivity of *Trans* -(TTP)TiL_2 (L = THF, *t* -BuNC). *Inorg. Chem.* **1998**, *37* (1), 5–9. https://doi.org/10.1021/ic970961n.
(11) Su, Z.-M.; Deng, R.; Stahl, S. S. Zinc and Manganese Redox Potentials in Organic Solvents and Their Influence on Nickel-Catalysed Cross-Electrophile Coupling. *Nat. Chem.* **2024**, *16* (12), 2036–2043. https://doi.org/10.1038/s41557-024-01627-5.
(12) Sandford, C.; Edwards, M. A.; Klunder, K. J.; Hickey, D. P.; Li, M.; Barman, K.; Sigman, M. S.; White, H. S.; Minteer, S. D. A Synthetic Chemist's Guide to Electroanalytical Tools for Studying Reaction Mechanisms. *Chem. Sci.* **2019**, *10* (26), 6404–6422. https://doi.org/10.1039/C9SC01545K.
(13) Siu, J. C.; Sauer, G. S.; Saha, A.; Macey, R. L.; Fu, N.; Chauviré, T.; Lancaster, K. M.; Lin, S. Electrochemical Azidooxygenation of Alkenes Mediated by a TEMPO–N_3 Charge-Transfer Complex. *J. Am. Chem. Soc.* **2018**, *140* (39), 12511–12520. https://doi.org/10.1021/jacs.8b06744.
(14) Malinski, T.; Chang, D.; Latour, J. M.; Marchon, J. C.; Gross, M.; Giraudeau, A.; Kadish, K. M. Electrochemistry of Oxo- and Peroxotitanium(IV) Porphyrins. Mechanism of the Two-Electron Reduction of a η^2-Coordinated Peroxo Ligand. *Inorg. Chem.* **1984**, *23* (24), 3947–3955. https://doi.org/10.1021/ic00192a022.
(15) Dawson, G. A.; Lin, Q.; Neary, M. C.; Diao, T. Ligand Redox Activity of Organonickel Radical Complexes Governed by the Geometry. *J. Am. Chem. Soc.* **2023**, *145* (37), 20551–20561. https://doi.org/10.1021/jacs.3c07031.
(16) Latour, J. M.; Marchon, J. C.; Nakajima, M. Titanium(III) Porphyrins and Their Dioxygen Adducts. *J. Am. Chem. Soc.* **1979**, *101* (14), 3974–3976. https://doi.org/10.1021/ja00508a050.
(17) Elias, J. S.; Costentin, C.; Nocera, D. G. Direct Electrochemical P(V) to P(III) Reduction of Phosphine Oxide Facilitated by Triaryl Borates. *J. Am. Chem. Soc.* **2018**, *140* (42), 13711–13718. https://doi.org/10.1021/jacs.8b07149.
(18) Hu, G.; Gupta, A. K.; Huang, R. H.; Mukherjee, M.; Wulff, W. D. Substrate-Induced Covalent Assembly of a Chemzyme and Crystallographic Characterization of a Chemzyme−Substrate Complex. *J. Am. Chem. Soc.* **2010**, *132* (41), 14669–14675. https://doi.org/10.1021/ja1070224.
(19) Frisch, M. J.; Trucks, G. W.; Schlegel, H. B.; Scuseria, G. E.; Robb, M. A.; Cheeseman, J. R.; Scalmani, G.; Barone, V.; Petersson, G. A.; Nakatsuji, H.; Li, X.; Caricato, M.; Marenich, A. V.; Bloino, J.; Janesko, B. G.; Gomperts, R.; Mennucci, B.; Hratchian, H. P.; Ortiz, J. V.; Izmaylov, A. F.; Sonnenberg, J. L.; Williams-Young, D.; Ding, F.; Lipparini, F.; Egidi, F.; Goings, J.; Peng, B.; Petrone, A.; Henderson, T.; Ranasinghe, D.; Zakrzewski, V. G.; Gao, J.; Rega, N.; Zheng, G.; Liang, W.; Hada, M.; Ehara, M.; Toyota, K.; Fukuda, R.; Hasegawa, J.; Ishida, M.; Nakajima, T.; Honda, Y.; Kitao, O.; Nakai, H.; Vreven, T.; Throssell, K.; Montgomery, J. A., Jr.; Peralta, J. E.; Ogliaro, F.; Bearpark, M. J.; Heyd, J. J.; Brothers, E. N.; Kudin, K. N.; Staroverov, V. N.; Keith, T. A.; Kobayashi, R.; Normand, J.; Raghavachari, K.; Rendell, A. P.; Burant, J. C.; Iyengar, S. S.; Tomasi, J.; Cossi, M.; Millam, J. M.; Klene, M.; Adamo, C.; Cammi, R.; Ochterski, J. W.; Martin, R. L.; Morokuma, K.; Farkas, O.; Foresman, J. B.; Fox, D. J. Gaussian˜16 Revision C.01, 2016.
(20) Lee, C.; Yang, W.; Parr, R. G. Development of the Colle-Salvetti Correlation-Energy Formula into a Functional of the Electron Density. *Phys. Rev. B* **1988**, *37* (2), 785–789. https://doi.org/10.1103/PhysRevB.37.785.
(21) Becke, A. D. Density-functional Thermochemistry. III. The Role of Exact Exchange. *J. Chem. Phys.* **1993**, *98* (7), 5648–5652. https://doi.org/10.1063/1.464913.
(22) Vosko, S. H.; Wilk, L.; Nusair, M. Accurate Spin-Dependent Electron Liquid Correlation Energies for Local Spin Density Calculations: A Critical Analysis. *Can. J. Phys.* **1980**, *58* (8), 1200–1211. https://doi.org/10.1139/p80-159.
(23) Stephens, P. J.; Devlin, F. J.; Chabalowski, C. F.; Frisch, M. J. Ab Initio Calculation of Vibrational Absorption and Circular Dichroism Spectra Using Density Functional Force Fields. *J. Phys. Chem.* **1994**, *98* (45), 11623–11627. https://doi.org/10.1021/j100096a001.

(24) Hay, P. J.; Wadt, W. R. Ab Initio Effective Core Potentials for Molecular Calculations. Potentials for the Transition Metal Atoms Sc to Hg. *J. Chem. Phys.* **1985**, *82* (1), 270–283. https://doi.org/10.1063/1.448799.
(25) Weigend, F.; Ahlrichs, R. Balanced Basis Sets of Split Valence, Triple Zeta Valence and Quadruple Zeta Valence Quality for H to Rn: Design and Assessment of Accuracy. *Phys. Chem. Chem. Phys.* **2005**, *7* (18), 3297–3305. https://doi.org/10.1039/B508541A.
(26) Cancès, E.; Mennucci, B.; Tomasi, J. A New Integral Equation Formalism for the Polarizable Continuum Model: Theoretical Background and Applications to Isotropic and Anisotropic Dielectrics. *J. Chem. Phys.* **1997**, *107* (8), 3032–3041. https://doi.org/10.1063/1.474659.
(27) Li, Y.-P.; Gomes, J.; Mallikarjun Sharada, S.; Bell, A. T.; Head-Gordon, M. Improved Force-Field Parameters for QM/MM Simulations of the Energies of Adsorption for Molecules in Zeolites and a Free Rotor Correction to the Rigid Rotor Harmonic Oscillator Model for Adsorption Enthalpies. *J. Phys. Chem. C* **2015**, *119* (4), 1840–1850. https://doi.org/10.1021/jp509921r.
(28) Grimme, S. Supramolecular Binding Thermodynamics by Dispersion-Corrected Density Functional Theory. *Chem. – Eur. J.* **2012**, *18* (32), 9955–9964. https://doi.org/10.1002/chem.201200497.
(29) Wertz, D. H. Relationship between the Gas-Phase Entropies of Molecules and Their Entropies of Solvation in Water and 1-Octanol. *J. Am. Chem. Soc.* **1980**, *102* (16), 5316–5322. https://doi.org/10.1021/ja00536a033.
(30) Cooper, J.; Ziegler, T. A Density Functional Study of SN2 Substitution at Square-Planar Platinum(II) Complexes. *Inorg. Chem.* **2002**, *41* (25), 6614–6622. https://doi.org/10.1021/ic020294k.
(31) Léonard, C.; Le Quéré, F.; Adjei, D.; Denisov, S. A.; Mostafavi, M.; Archirel, P. Oxidation of Silver Cyanide Ag(CN)2– by the OH Radical: From Ab Initio Calculation to Molecular Simulation and to Experiment. *J. Phys. Chem. A* **2020**, *124* (51), 10787–10798. https://doi.org/10.1021/acs.jpca.0c08038.
(32) Kong, Z.; Pan, C.; Li, M.; Wen, L.; Guo, W. Scalable Electrochemical Reduction of Sulfoxides to Sulfides. *Green Chem.* **2021**, *23* (7), 2773–2777. https://doi.org/10.1039/D1GC00591J.
(33) Gao, Y.; Chai, F.; Szostak, M.; Liu, C. Samarium(II) Diiodide-Mediated Deoxygenation of Sulfoxides. *J. Org. Chem.* **2025**, *90* (6), 2421–2428. https://doi.org/10.1021/acs.joc.4c02971.
(34) Ishikawa, H.; Yamaguchi, S.; Nakata, A.; Nakajima, K.; Yamazoe, S.; Yamasaki, J.; Mizugaki, T.; Mitsudome, T. Phosphorus-Alloying as a Powerful Method for Designing Highly Active and Durable Metal Nanoparticle Catalysts for the Deoxygenation of Sulfoxides: Ligand and Ensemble Effects of Phosphorus. *JACS Au* **2022**, *2* (2), 419–427. https://doi.org/10.1021/jacsau.1c00461.
(35) Kim, M.; Na, H.; Shen, L.; Jeong, J. H. Direct Introduction of an Acetyl Group at the α-Carbon Atom of an Arene Ring through an Amide Photo-Fries Rearrangement upon Exposure to UV Light. *Eur. J. Org. Chem.* **2024**, *27* (26), e202400281. https://doi.org/10.1002/ejoc.202400281.
(36) Kokatla, H. P.; Thomson, P. F.; Bae, S.; Doddi, V. R.; Lakshman, M. K. Reduction of Amine *N* -Oxides by Diboron Reagents. *J. Org. Chem.* **2011**, *76* (19), 7842–7848. https://doi.org/10.1021/jo201192c.
(37) Lecroq, W.; Schleinitz, J.; Billoue, M.; Perfetto, A.; Gaumont, A.; Lalevée, J.; Ciofini, I.; Grimaud, L.; Lakhdar, S. Metal-Free Deoxygenation of Amine *N* -Oxides: Synthetic and Mechanistic Studies. *ChemPhysChem* **2021**, *22* (12), 1237–1242. https://doi.org/10.1002/cphc.202100108.
(38) Wang, L.; Tu, Y.; Valkonen, A.; Rissanen, K.; Jiang, W. Selective Recognition of Phenazine by 2,6-Dibutoxylnaphthalene-Based Tetralactam Macrocycle. *Chin. J. Chem.* **2019**, *37* (9), 892–896. https://doi.org/10.1002/cjoc.201900233.
(39) Cao, D.; Xia, S.; Li, L.; Zeng, H.; Li, C.-J. PPh_3 -Promoted Direct Deoxygenation of Epoxides to Alkenes. *Org. Lett.* **2024**, *26* (30), 6418–6423. https://doi.org/10.1021/acs.orglett.4c02207.
(40) Cabral, T. L. G.; Poggetto, G. D.; Brussolo Da Silva, J. P.; Nilsson, M.; Tormena, C. F. Determining the Absolute Configuration of Small Molecules by Diffusion NMR Experiments. *Angew. Chem. Int. Ed.* **2025**, *64* (5), e202418508. https://doi.org/10.1002/anie.202418508.

(41) Tibbetts, J. D.; Bull, S. D. Dimethyl Sulfide Facilitates Acid Catalysed Ring Opening of the Bicyclic Monoterpenes in Crude Sulfate Turpentine to Afford *p* -Menthadienes in Good Yield. *Green Chem.* **2021**, *23* (1), 597–610. https://doi.org/10.1039/D0GC03452E.
(42) APEX6, 2024.
(43) Krause, L.; Herbst-Irmer, R.; Sheldrick, G. M.; Stalke, D. Comparison of Silver and Molybdenum Microfocus X-Ray Sources for Single-Crystal Structure Determination. *J. Appl. Crystallogr.* **2015**, *48* (1), 3–10. https://doi.org/10.1107/S1600576714022985.
(44) Sheldrick, G. M. XPREP, 2013.
(45) Sheldrick, G. M. *The* SHELX *homepage*.
(46) Sheldrick, G. M. *SHELXT* – Integrated Space-Group and Crystal-Structure Determination. *Acta Crystallogr. Sect. Found. Adv.* **2015**, *71* (1), 3–8. https://doi.org/10.1107/S2053273314026370.
(47) Sheldrick, G. M. Crystal Structure Refinement with *SHELXL*. *Acta Crystallogr. Sect. C Struct. Chem.* **2015**, *71* (1), 3–8. https://doi.org/10.1107/S2053229614024218.
(48) Dolomanov, O. V.; Bourhis, L. J.; Gildea, R. J.; Howard, J. A. K.; Puschmann, H. *OLEX2* : A Complete Structure Solution, Refinement and Analysis Program. *J. Appl. Crystallogr.* **2009**, *42* (2), 339–341. https://doi.org/10.1107/S0021889808042726.
(49) Guzei, I. A. Programs *Gn*., 2007.
(50) Guzei, I. A. An Idealized Molecular Geometry Library for Refinement of Poorly Behaved Molecular Fragments with Constraints. *J. Appl. Crystallogr.* **2014**, *47* (2), 806–809. https://doi.org/10.1107/S1600576714004427.
(51) Spek, A. L. *PLATON* SQUEEZE: A Tool for the Calculation of the Disordered Solvent Contribution to the Calculated Structure Factors. *Acta Crystallogr. Sect. C Struct. Chem.* **2015**, *71* (1), 9–18. https://doi.org/10.1107/S2053229614024929.

13. NMR Spectra

(see the following pages for NMR spectroscopic data)

^{1}H NMR (500 MHz, $CDCl_3$)

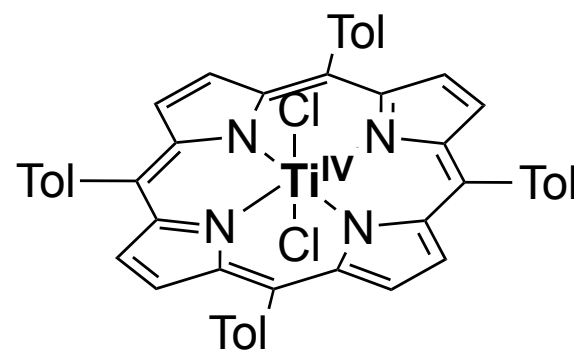

(TTP)Ti^{IV}Cl$_2$

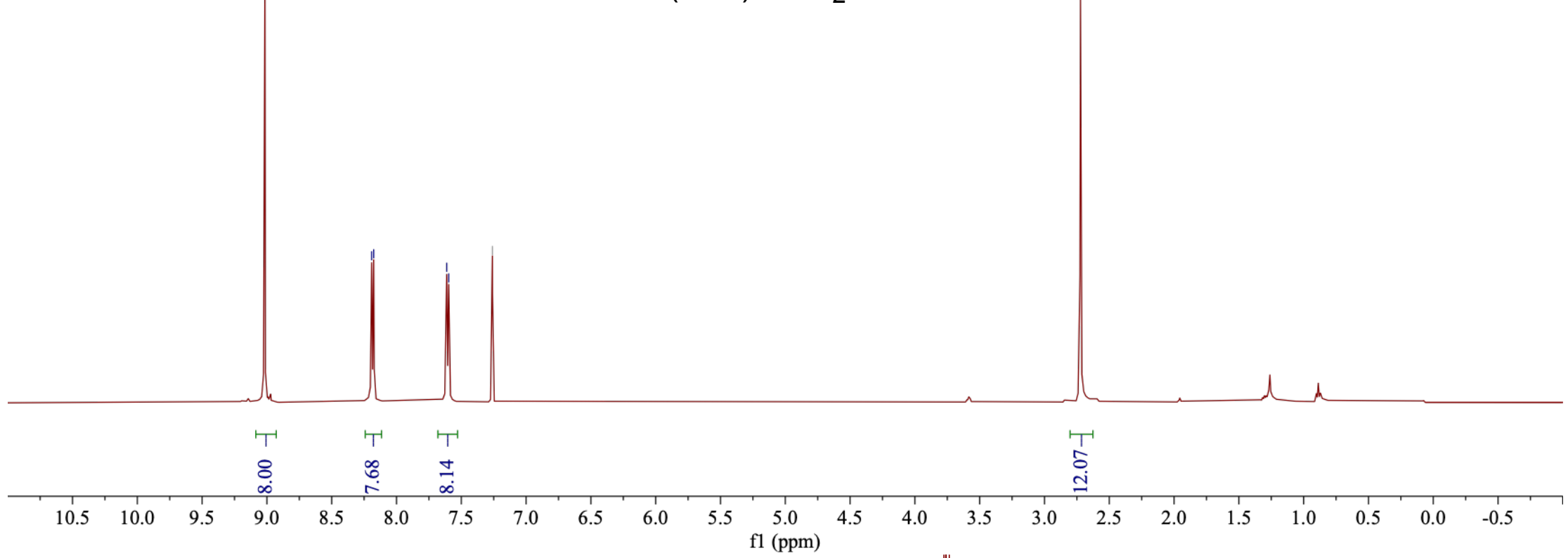

^{13}C NMR (126 MHz, $CDCl_3$)

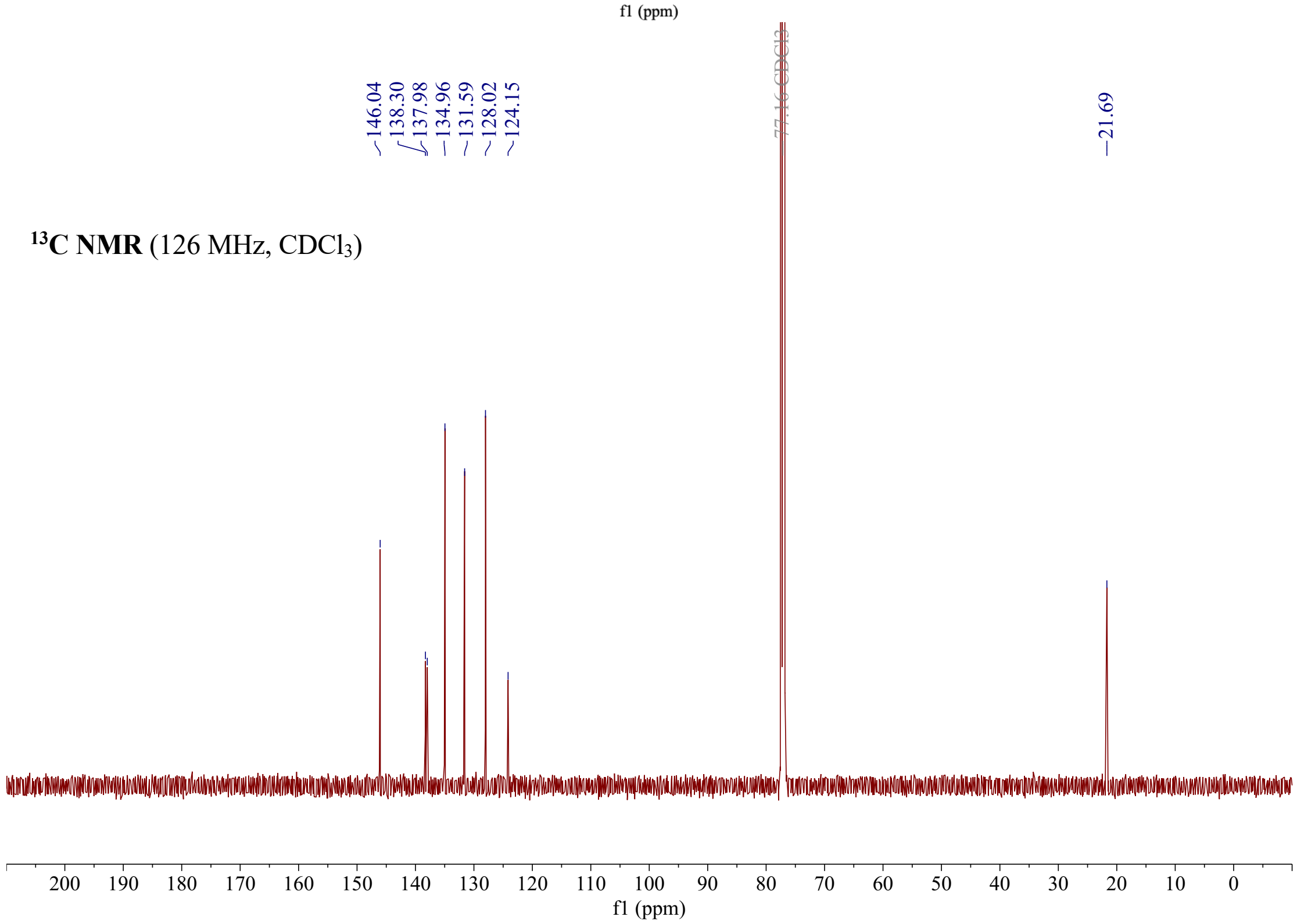

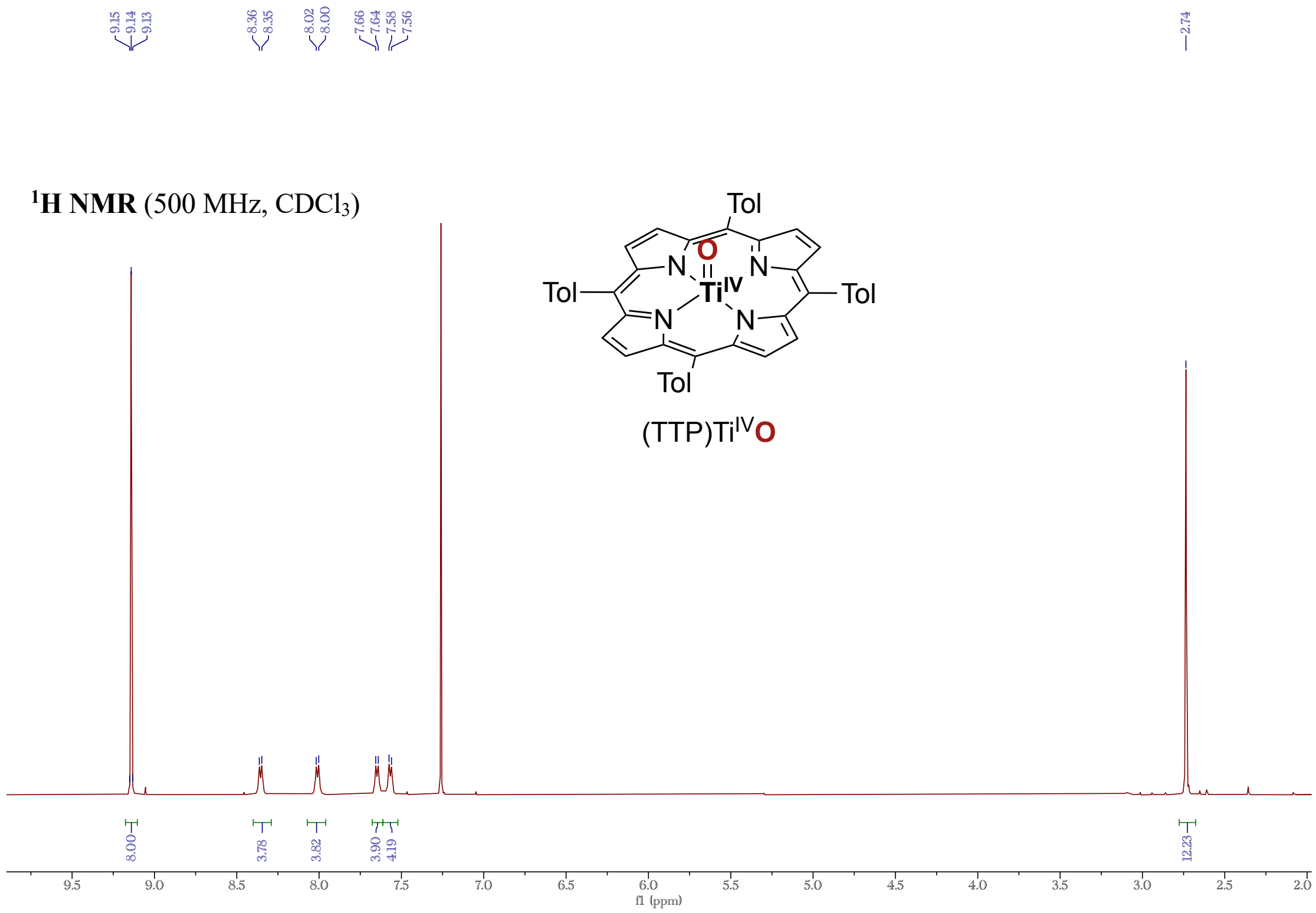

9.15
9.14
9.13
8.36
8.35
8.02
8.00
7.66
7.64
7.58
7.56
2.74
¹H NMR (500 MHz, CDCl₃)
Tol
O
N
Ti^IV
N
N
N
Tol
Tol
Tol
(TTP)Ti^IV O
8.00
3.78
3.82
3.90
4.19
12.23
9.5
9.0
8.5
8.0
7.5
7.0
6.5
6.0
5.5
5.0
4.5
4.0
3.5
3.0
2.5
2.0
f1 (ppm)

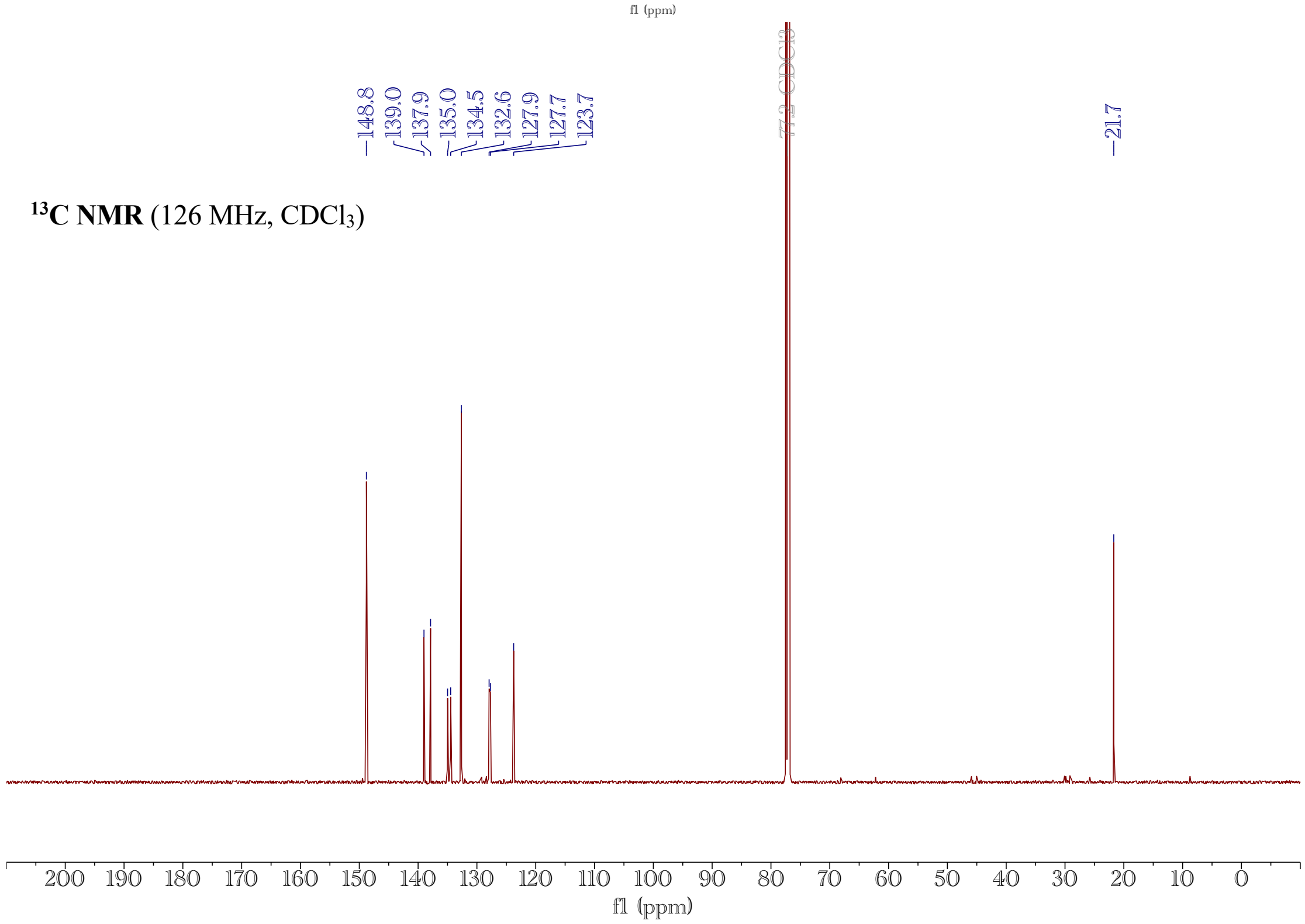

148.8
139.0
137.9
135.0
134.5
132.6
127.9
127.7
123.7
77.2 CDCl3
21.7
¹³C NMR (126 MHz, CDCl₃)
200
190
180
170
160
150
140
130
120
110
100
90
80
70
60
50
40
30
20
10
0
f1 (ppm)

^{1}H NMR (500 MHz, $CDCl_3$)

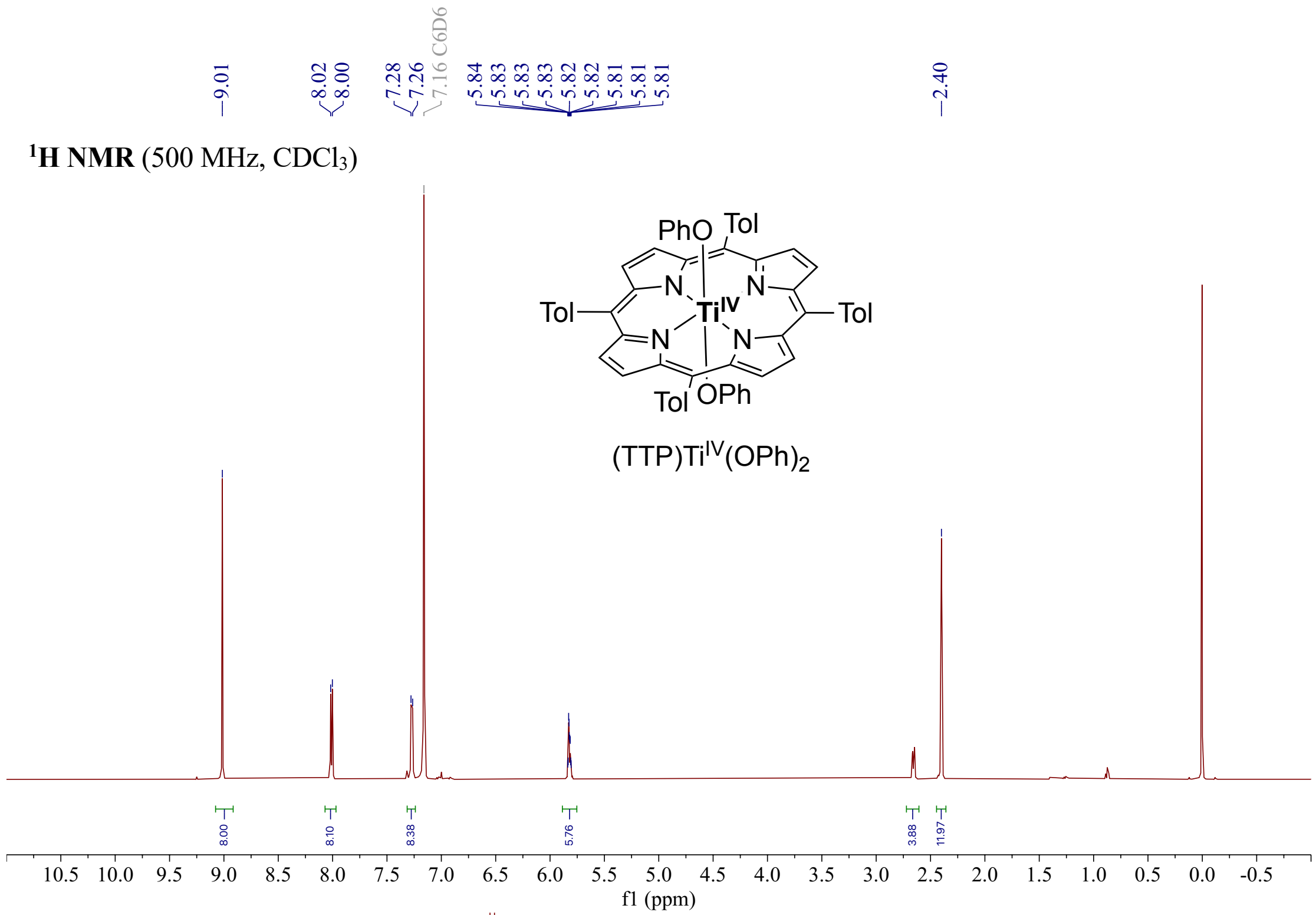

^{13}C NMR (126 MHz, $CDCl_3$)

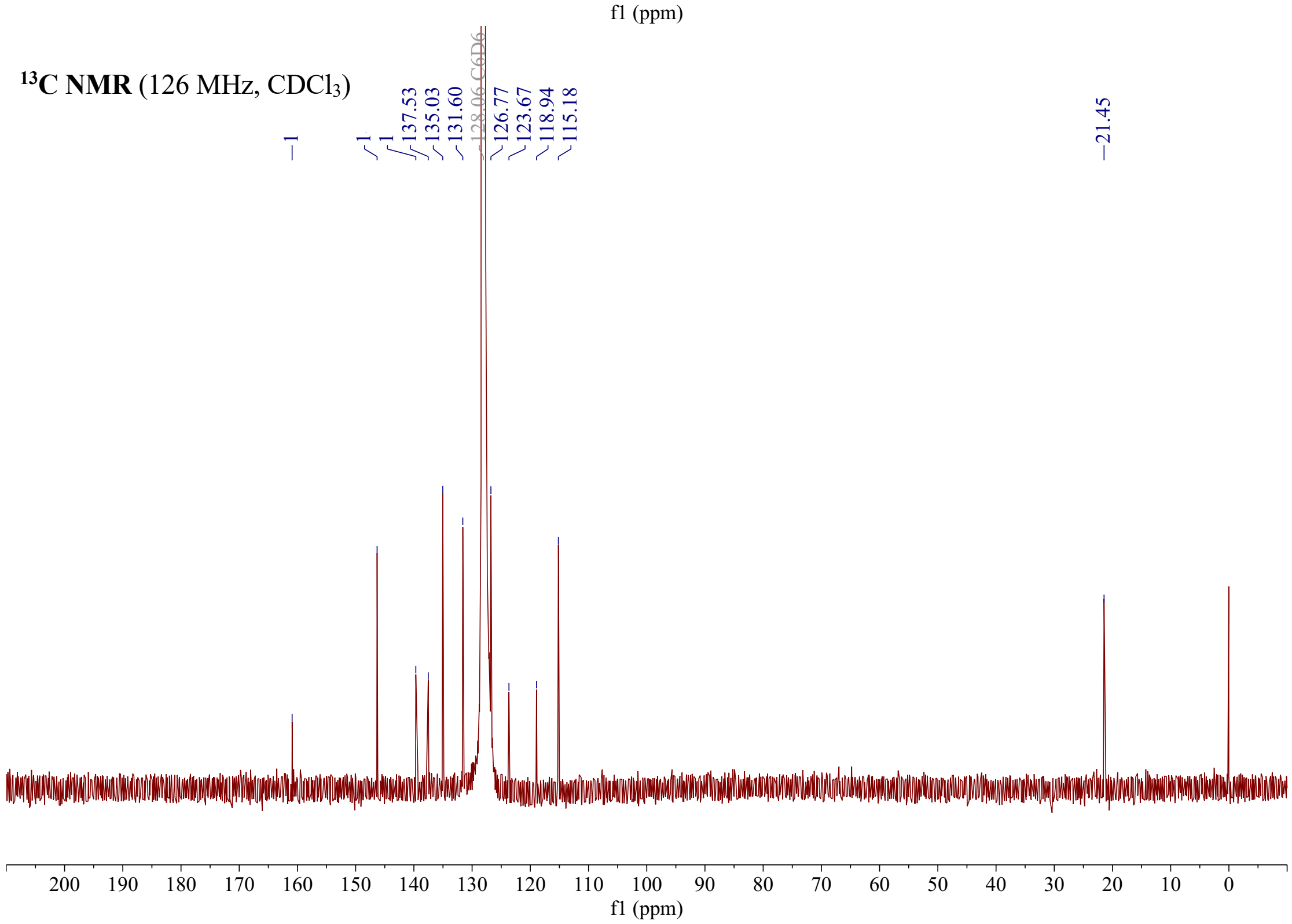

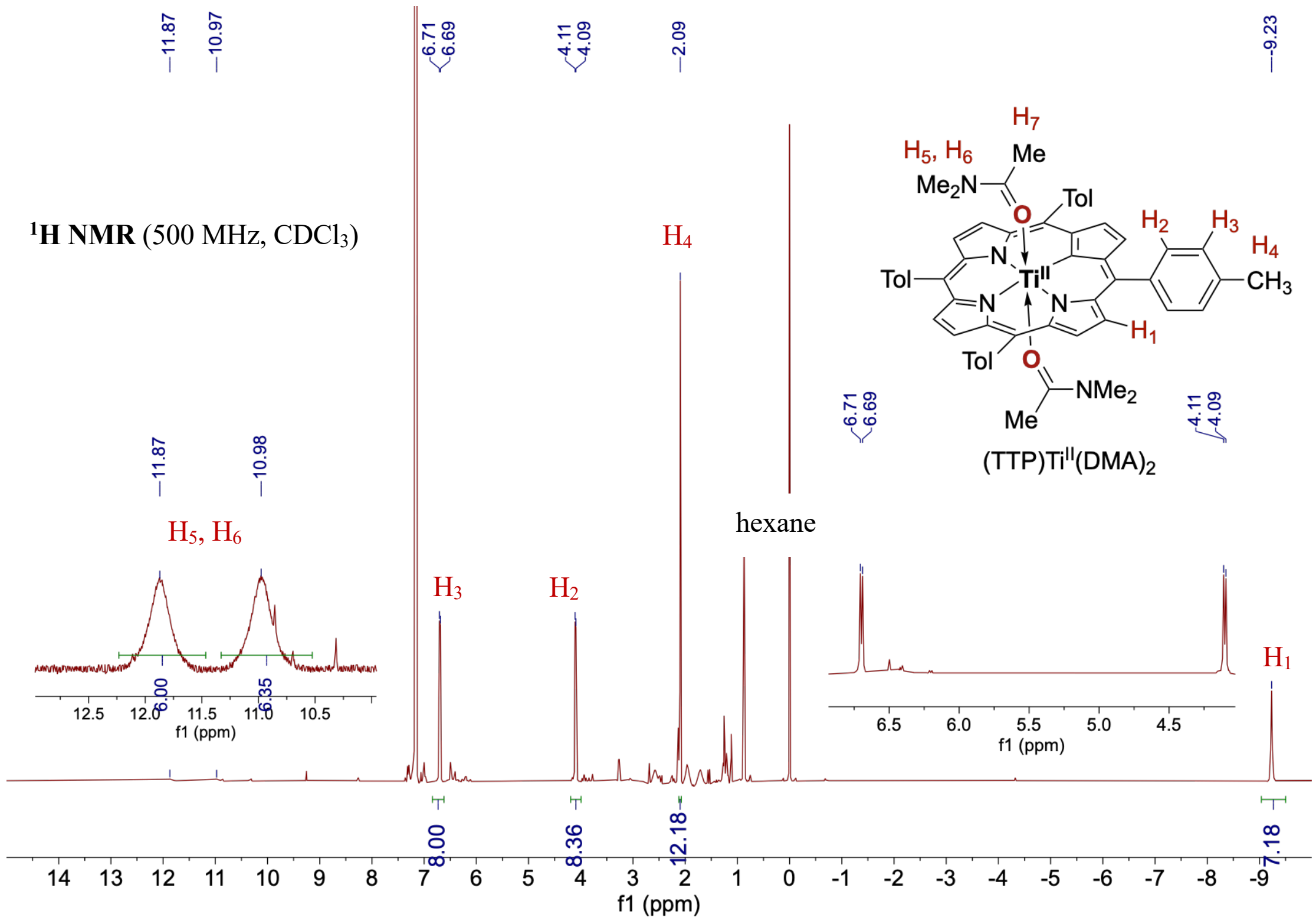

^{1}H NMR (500 MHz, $CDCl_3$)
H5, H6
H7
Me
Me2N
Tol
H2
H3
H4
CH3
H1
(TTP)TiII(DMA)2
hexane
f1 (ppm)

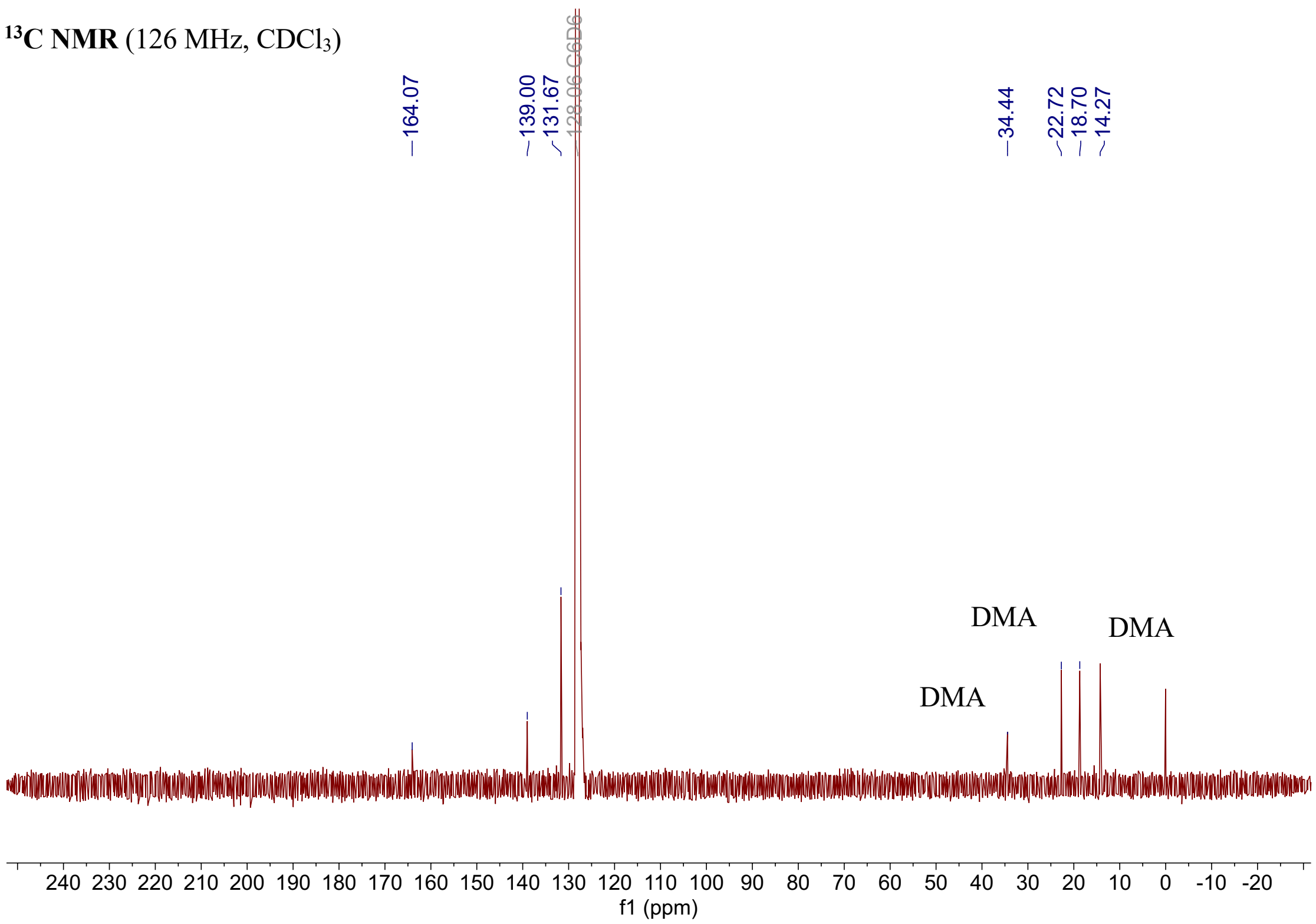

^{13}C NMR (126 MHz, $CDCl_3$)
DMA
DMA
DMA
f1 (ppm)

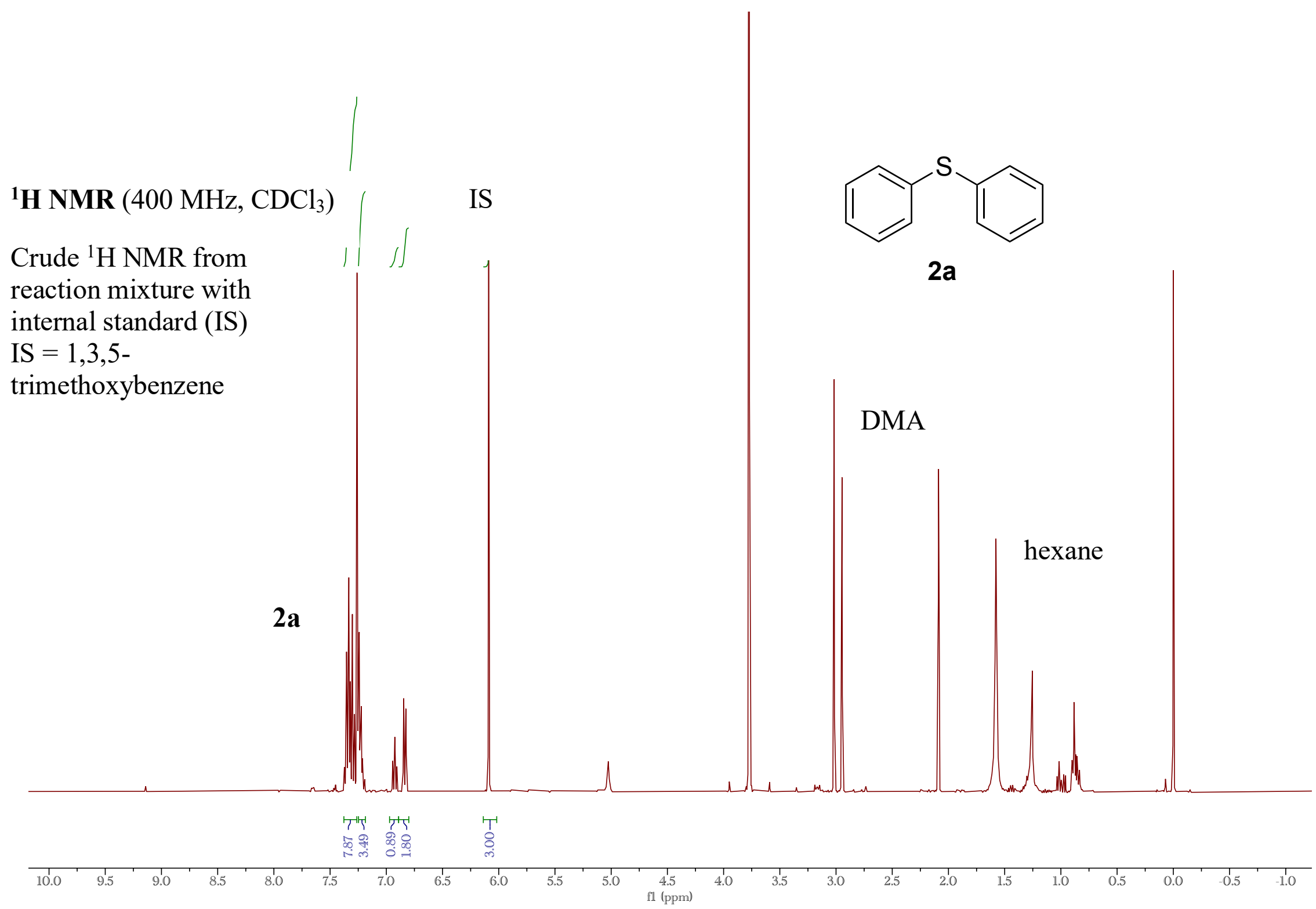
^{1}H NMR (400 MHz, CDCl$_3$)
Crude ^{1}H NMR from reaction mixture with internal standard (IS)
IS = 1,3,5-trimethoxybenzene
IS
2a
DMA
hexane
f1 (ppm)

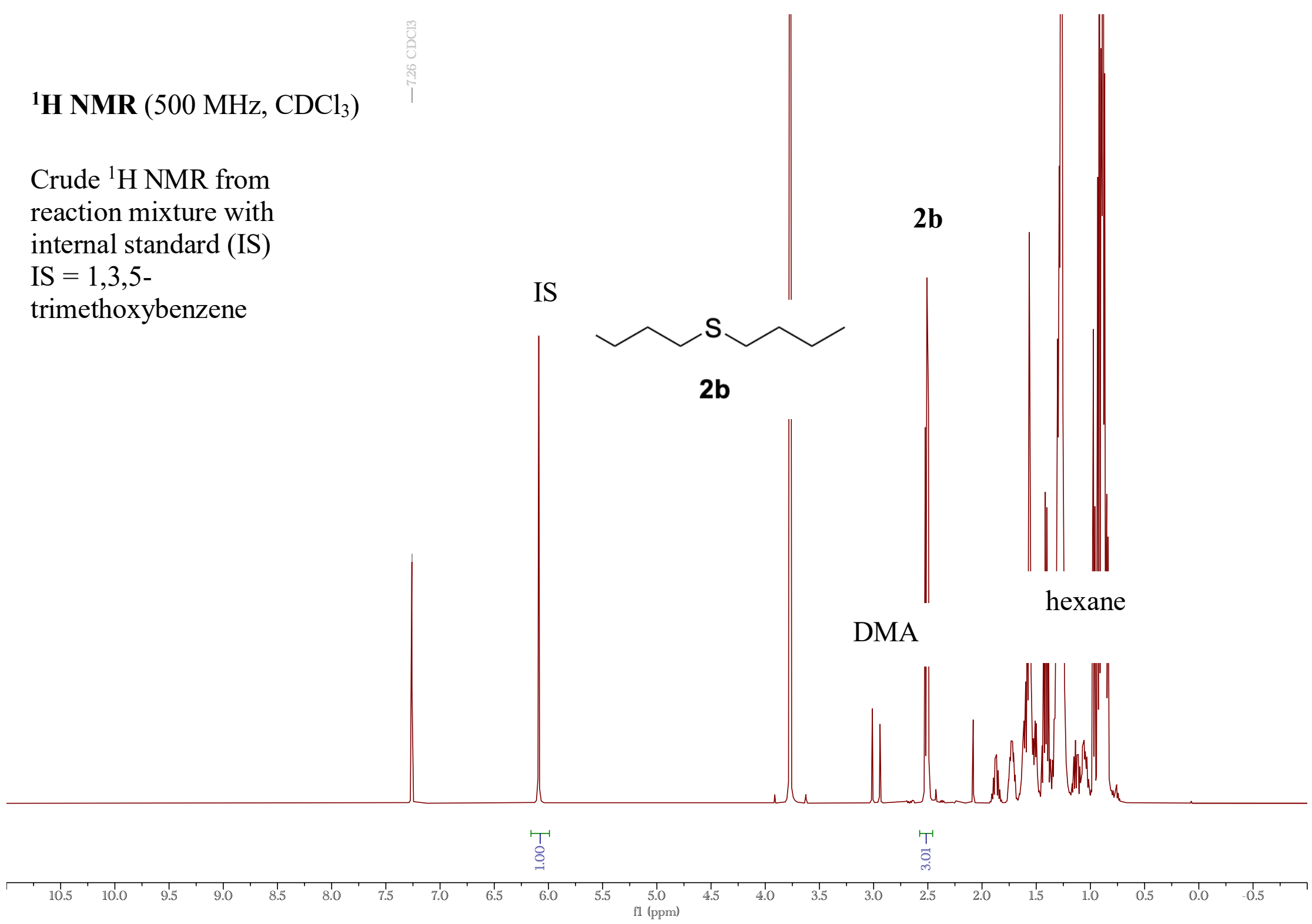
^{1}H NMR (500 MHz, CDCl$_3$)
Crude ^{1}H NMR from reaction mixture with internal standard (IS)
IS = 1,3,5-trimethoxybenzene
IS
2b
DMA
hexane
f1 (ppm)

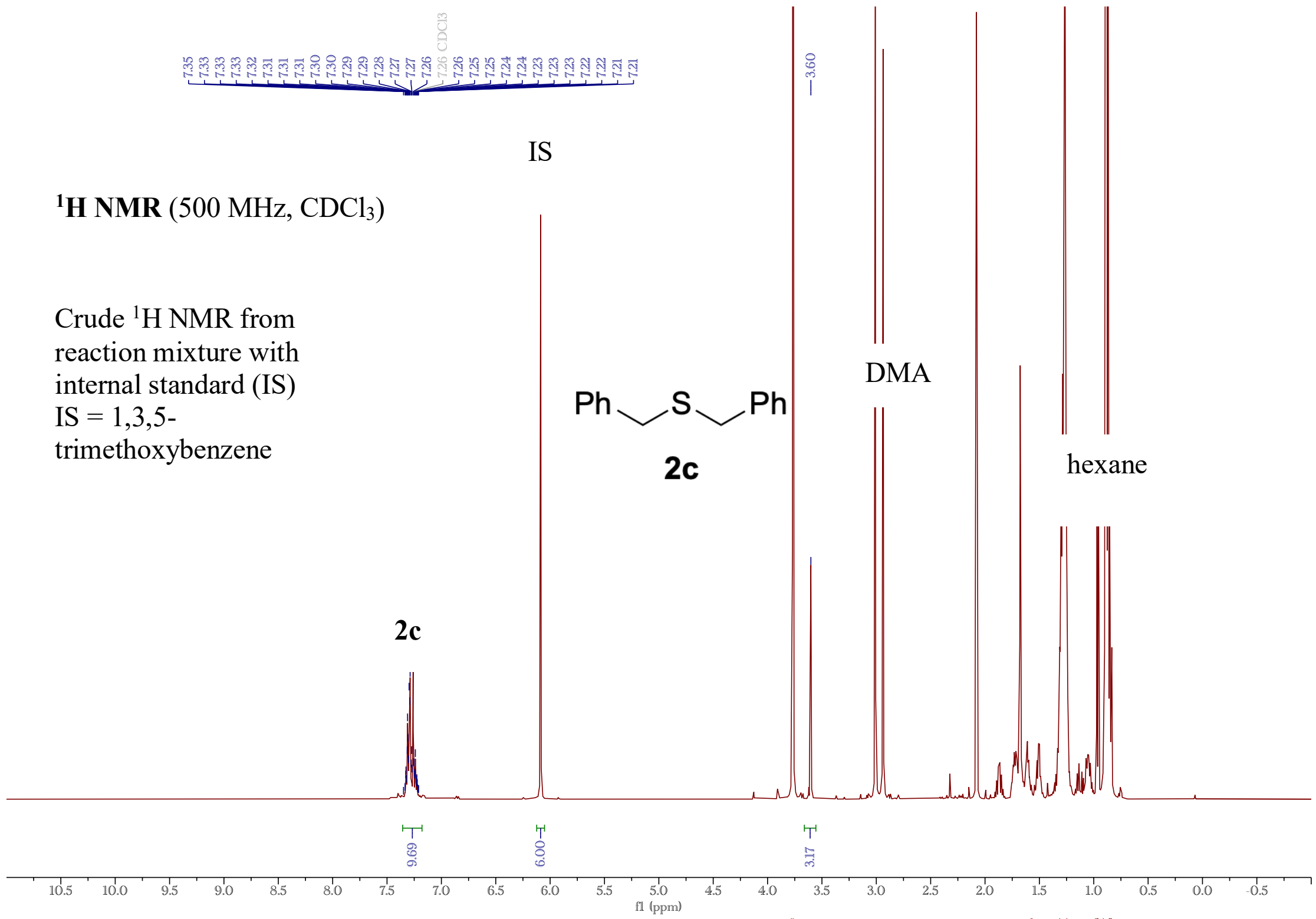

¹H NMR (500 MHz, CDCl3)
Crude ¹H NMR from reaction mixture with internal standard (IS)
IS = 1,3,5-trimethoxybenzene
IS
DMA
hexane
Ph S Ph
2c
2c
f1 (ppm)

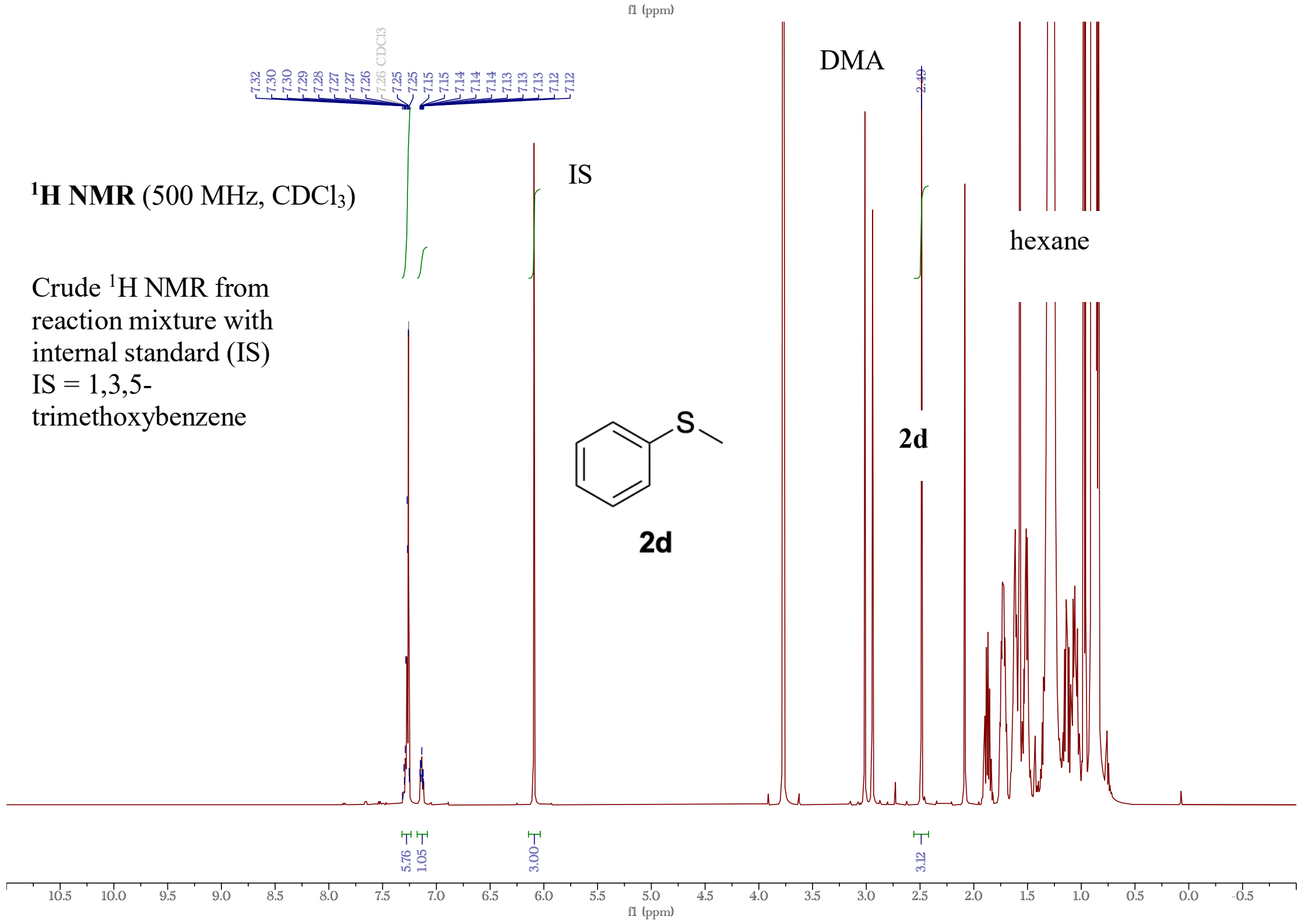

¹H NMR (500 MHz, CDCl3)
Crude ¹H NMR from reaction mixture with internal standard (IS)
IS = 1,3,5-trimethoxybenzene
IS
DMA
hexane
S
2d
2d
f1 (ppm)

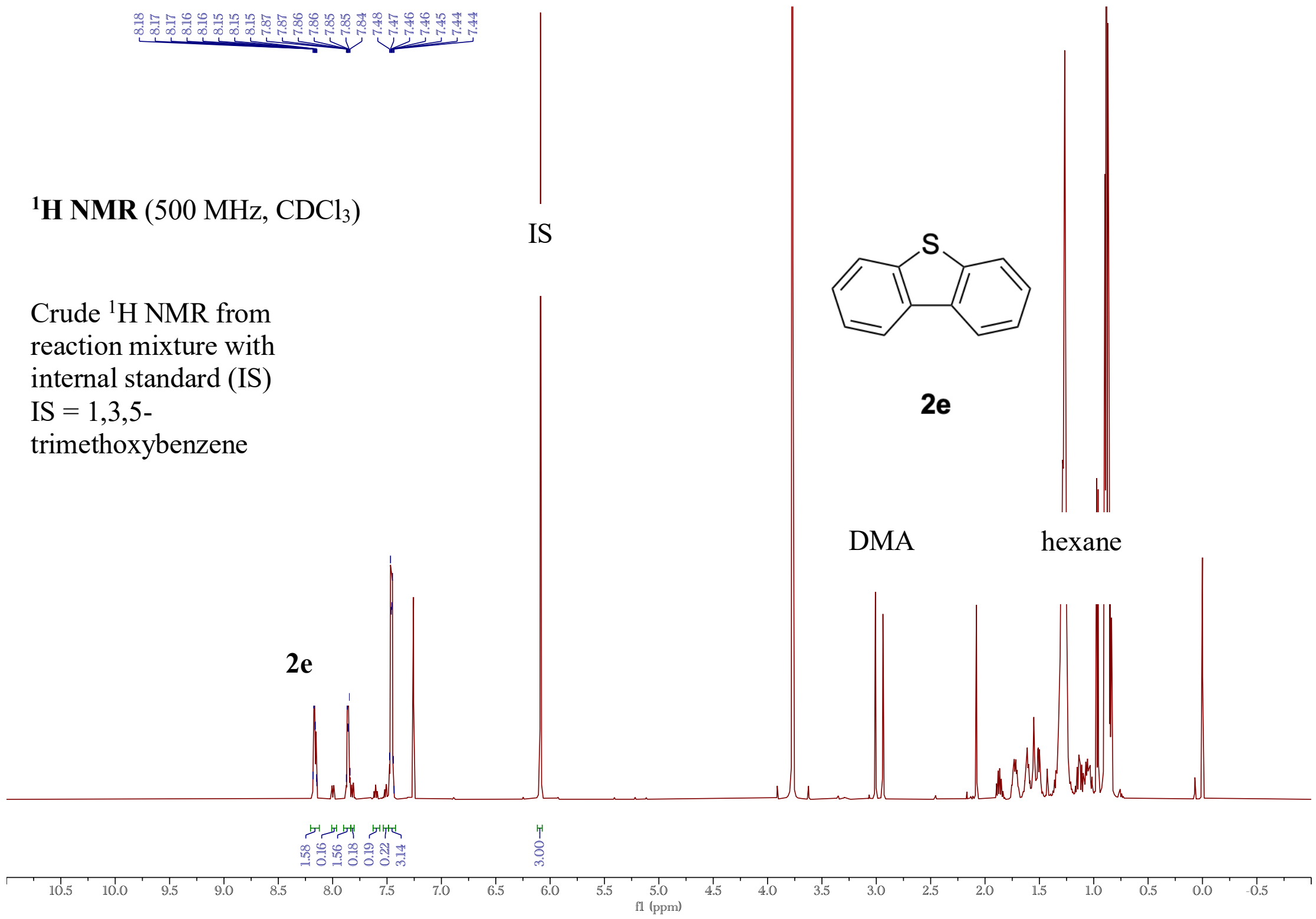

8.18
8.17
8.17
8.16
8.16
8.15
8.15
8.15
7.87
7.87
7.86
7.86
7.85
7.85
7.84
7.48
7.47
7.46
7.46
7.45
7.44
7.44
^{1}H NMR (500 MHz, CDCl3)
Crude ^{1}H NMR from
reaction mixture with
internal standard (IS)
IS = 1,3,5-
trimethoxybenzene
IS
S
2e
DMA
hexane
2e
1.58
0.16
1.56
0.18
0.19
0.22
3.14
3.00
10.5
10.0
9.5
9.0
8.5
8.0
7.5
7.0
6.5
6.0
5.5
5.0
4.5
4.0
3.5
3.0
2.5
2.0
1.5
1.0
0.5
0.0
-0.5
f1 (ppm)

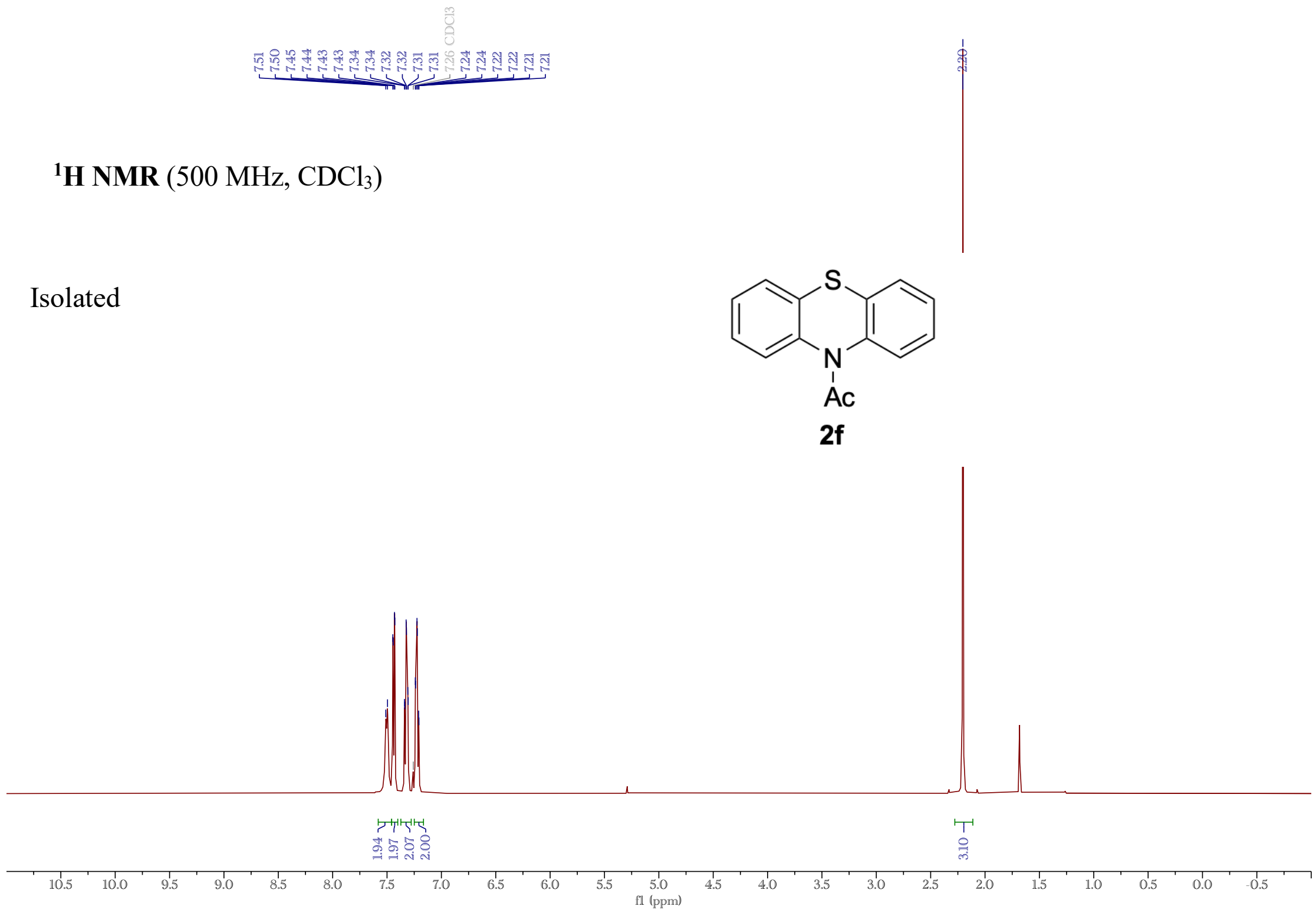

7.51
7.50
7.45
7.44
7.43
7.43
7.34
7.34
7.32
7.32
7.31
7.31
7.26 CDCl3
7.24
7.24
7.22
7.22
7.21
7.21
2.20
¹H NMR (500 MHz, CDCl3)
Isolated
S
N
Ac
2f
1.94
1.97
2.07
2.00
3.10
f1 (ppm)

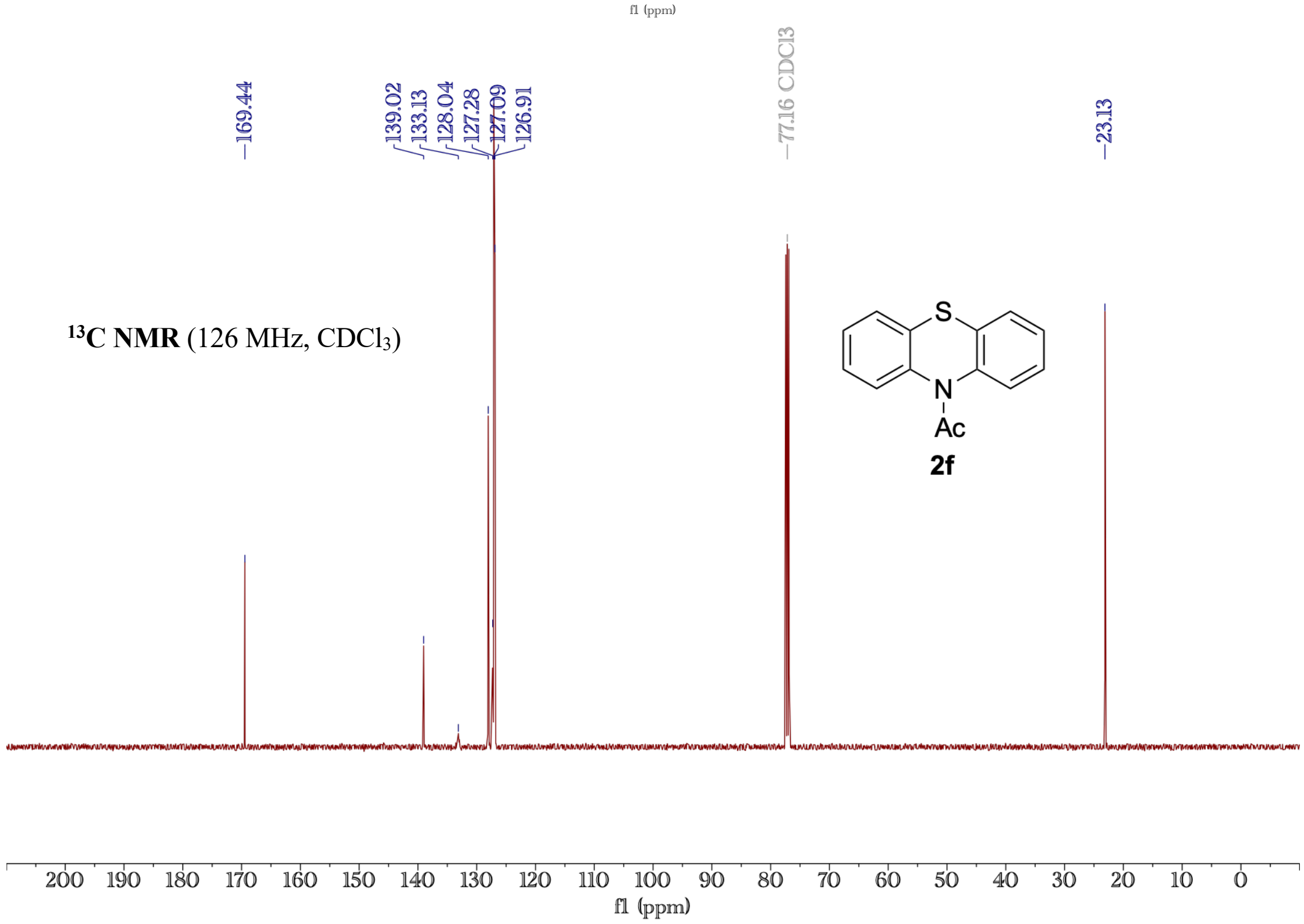

169.44
139.02
133.13
128.04
127.28
127.09
126.91
77.16 CDCl3
23.13
¹³C NMR (126 MHz, CDCl3)
S
N
Ac
2f
f1 (ppm)

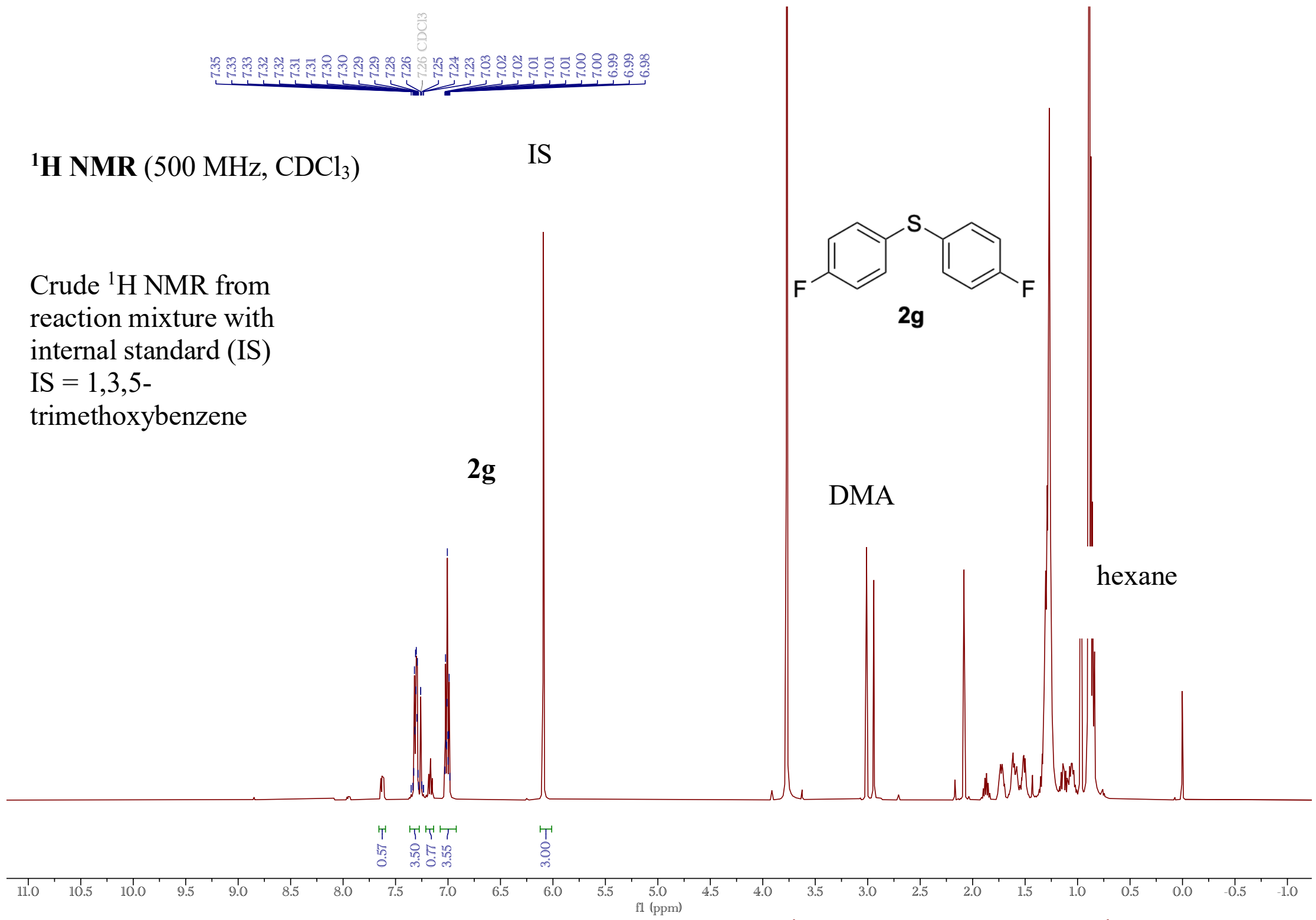

¹H NMR (500 MHz, CDCl3)
Crude ¹H NMR from reaction mixture with internal standard (IS)
IS = 1,3,5-trimethoxybenzene
IS
2g
DMA
hexane
F
S
F
2g
0.57
3.50
0.77
3.55
3.00
f1 (ppm)

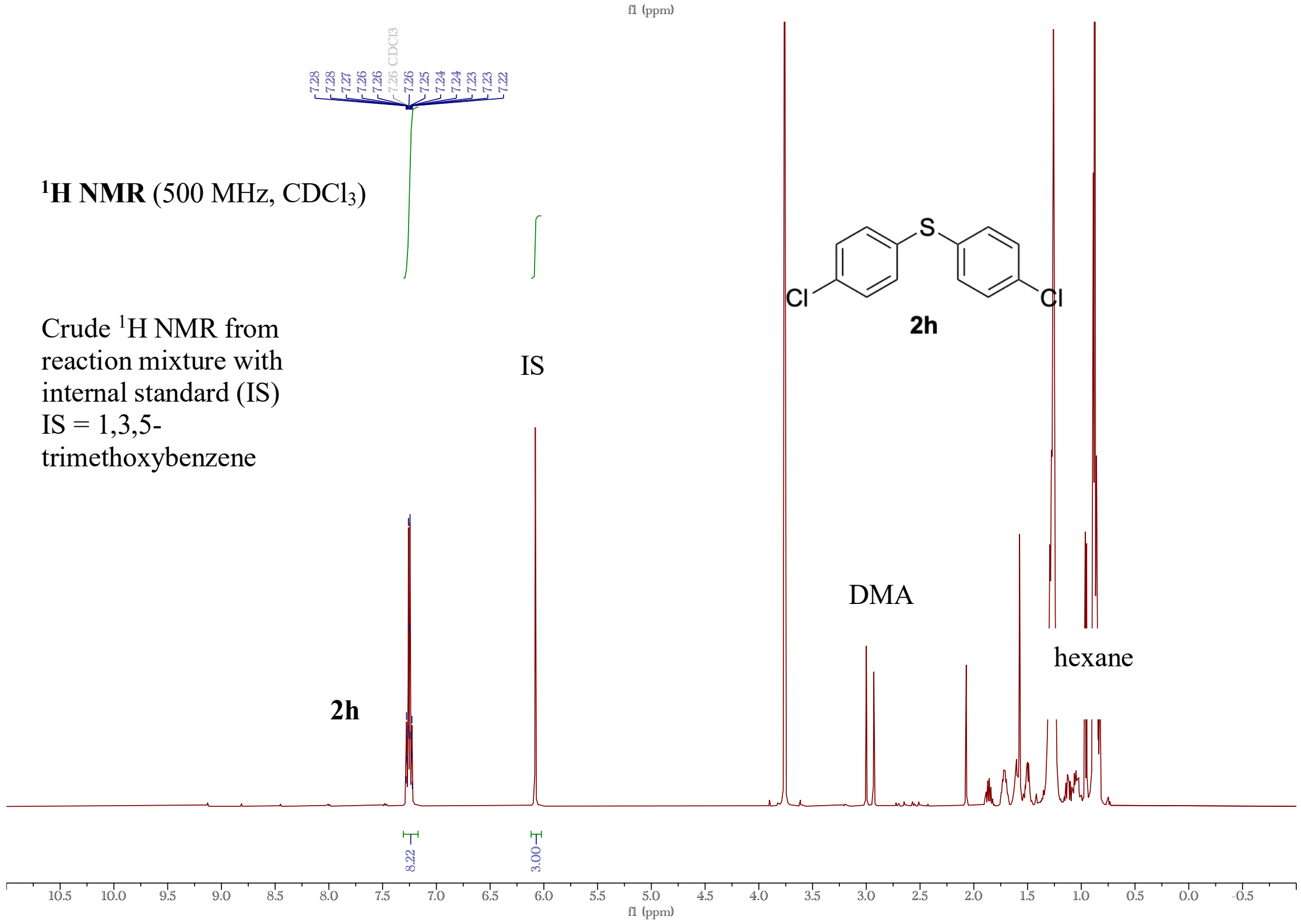

¹H NMR (500 MHz, CDCl3)
Crude ¹H NMR from reaction mixture with internal standard (IS)
IS = 1,3,5-trimethoxybenzene
IS
2h
DMA
hexane
Cl
S
Cl
2h
8.22
3.00
f1 (ppm)

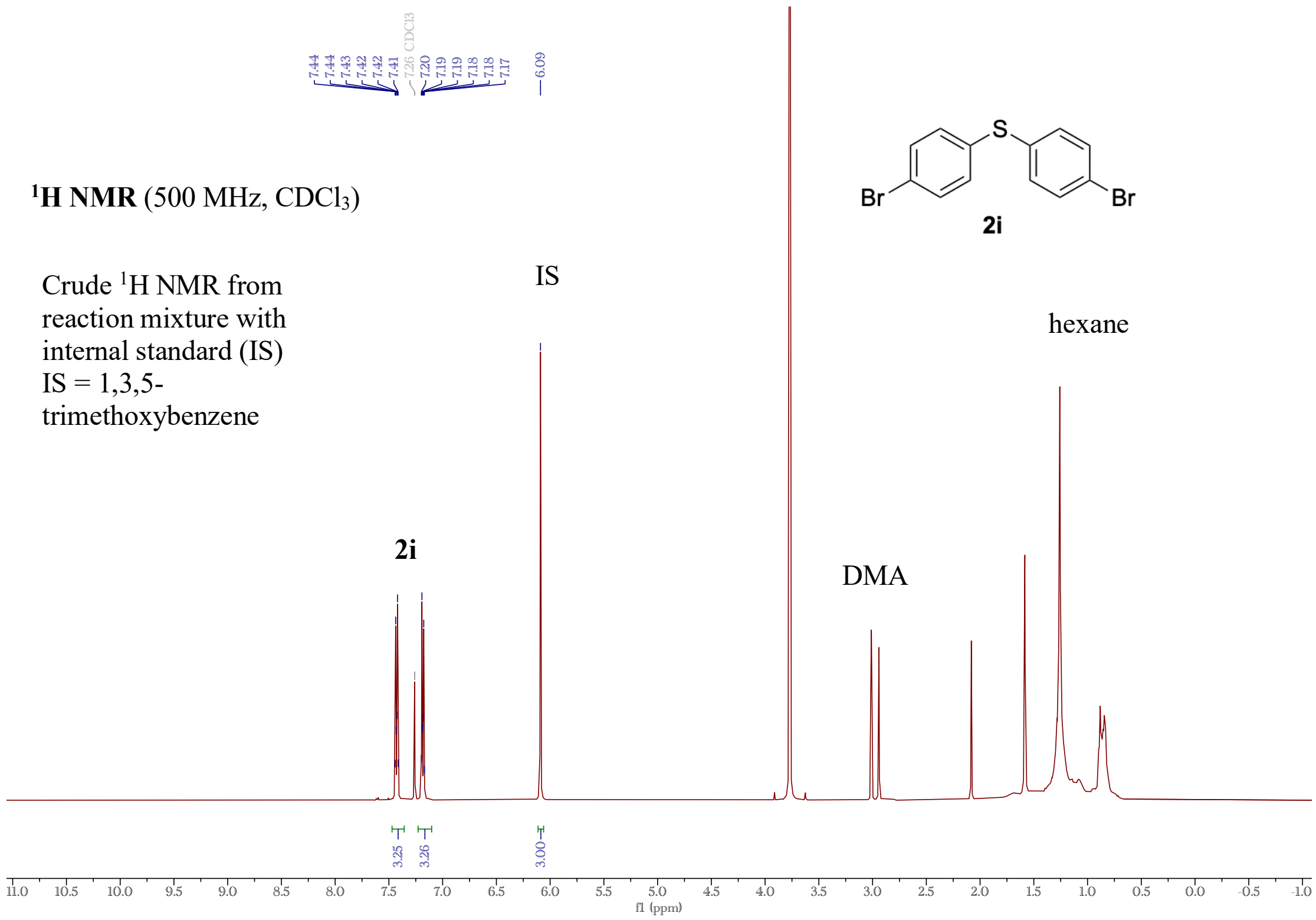

^{1}H NMR (500 MHz, CDCl$_3$)
Crude ^{1}H NMR from reaction mixture with internal standard (IS)
IS = 1,3,5-trimethoxybenzene
IS
2i
DMA
hexane
Br
S
Br
2i
f1 (ppm)

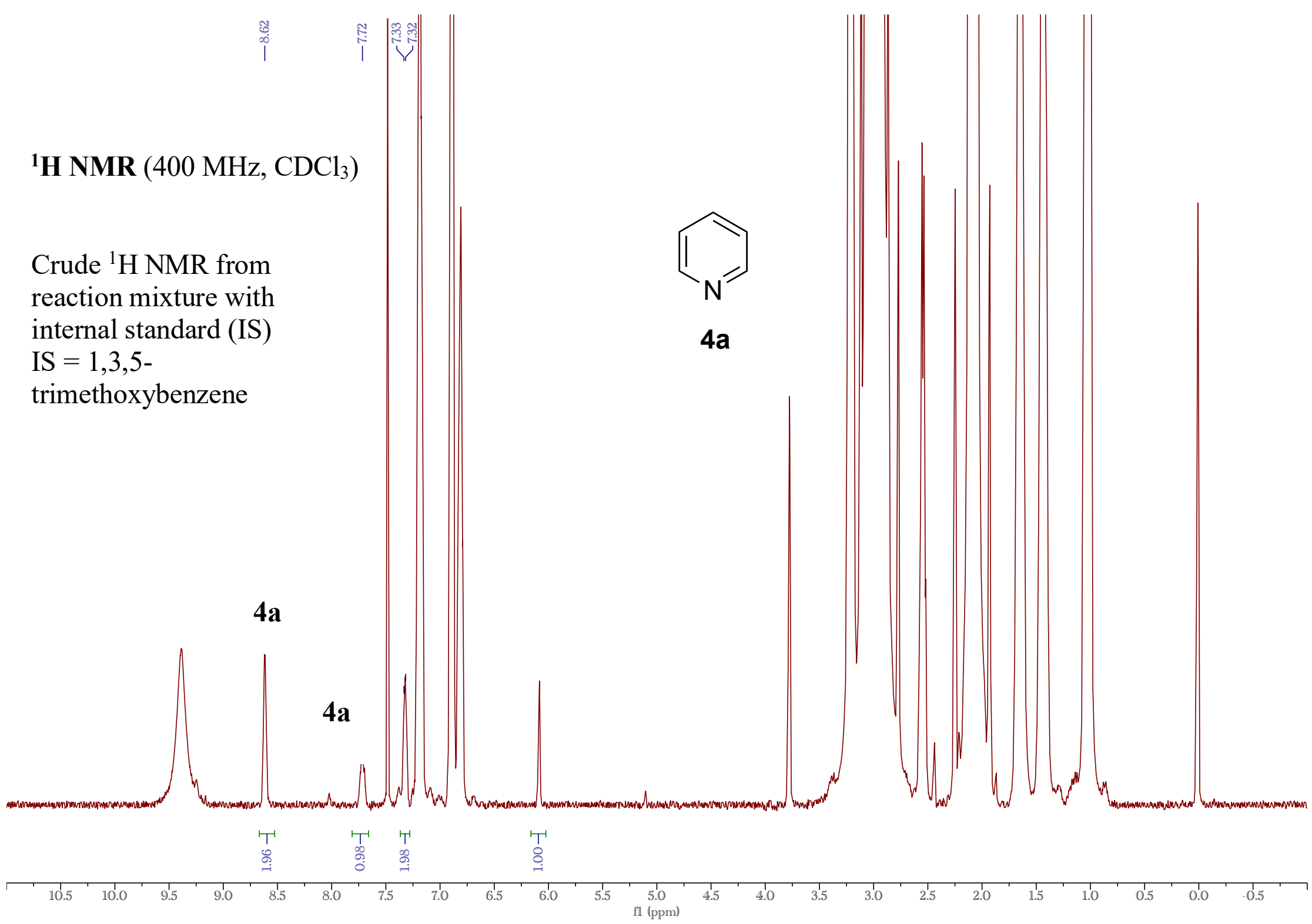

^{1}H NMR (400 MHz, CDCl$_3$)
Crude ^{1}H NMR from reaction mixture with internal standard (IS)
IS = 1,3,5-trimethoxybenzene
N
4a
4a
4a
f1 (ppm)

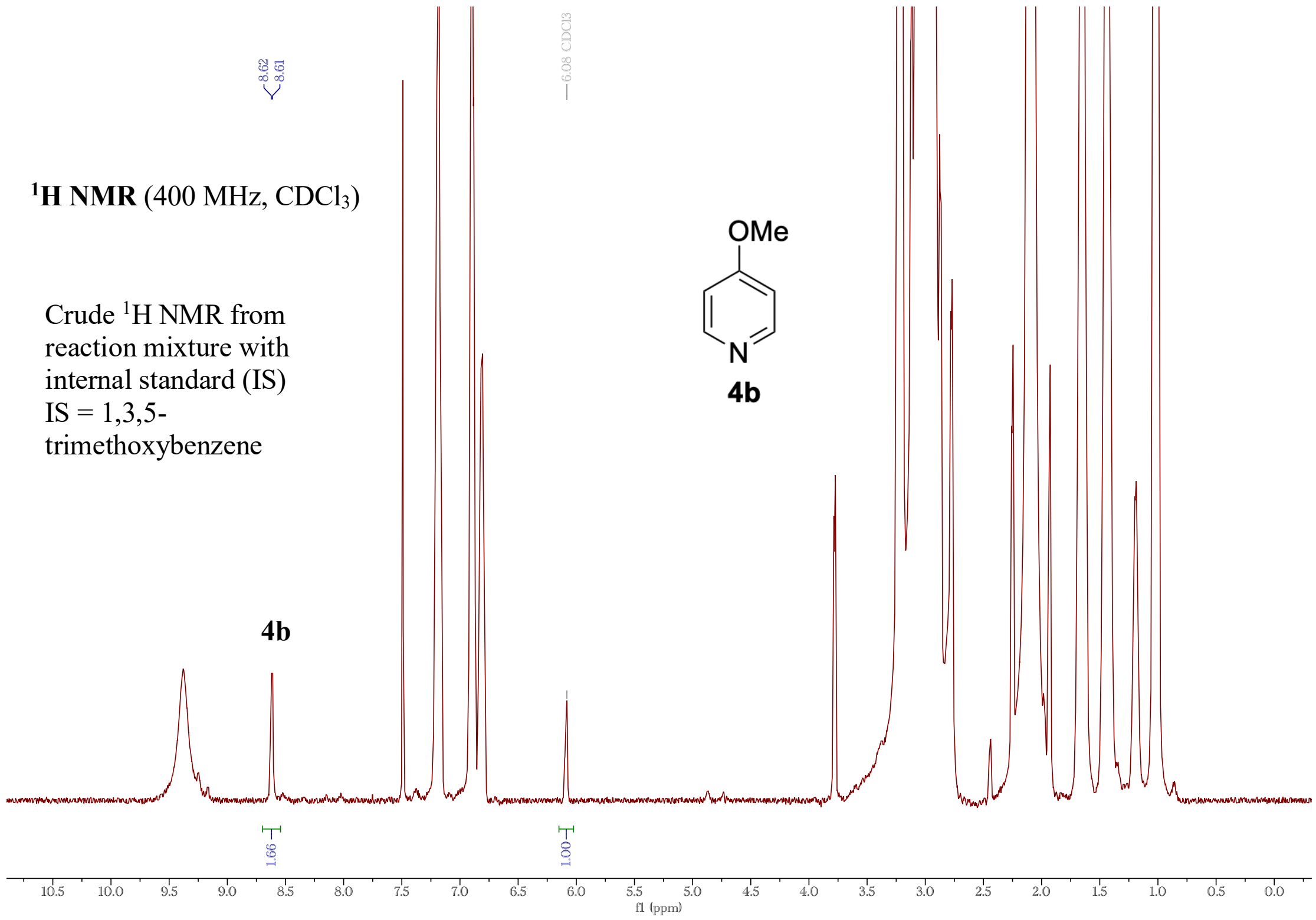

1H NMR (400 MHz, CDCl3)
Crude 1H NMR from reaction mixture with internal standard (IS)
IS = 1,3,5-trimethoxybenzene
OMe
N
4b
4b
8.62
8.61
6.08 CDCl3
1.66
1.00
10.5 10.0 9.5 9.0 8.5 8.0 7.5 7.0 6.5 6.0 5.5 5.0 4.5 4.0 3.5 3.0 2.5 2.0 1.5 1.0 0.5 0.0
f1 (ppm)

^{1}H NMR (500 MHz, $CDCl_3$)

Isolated

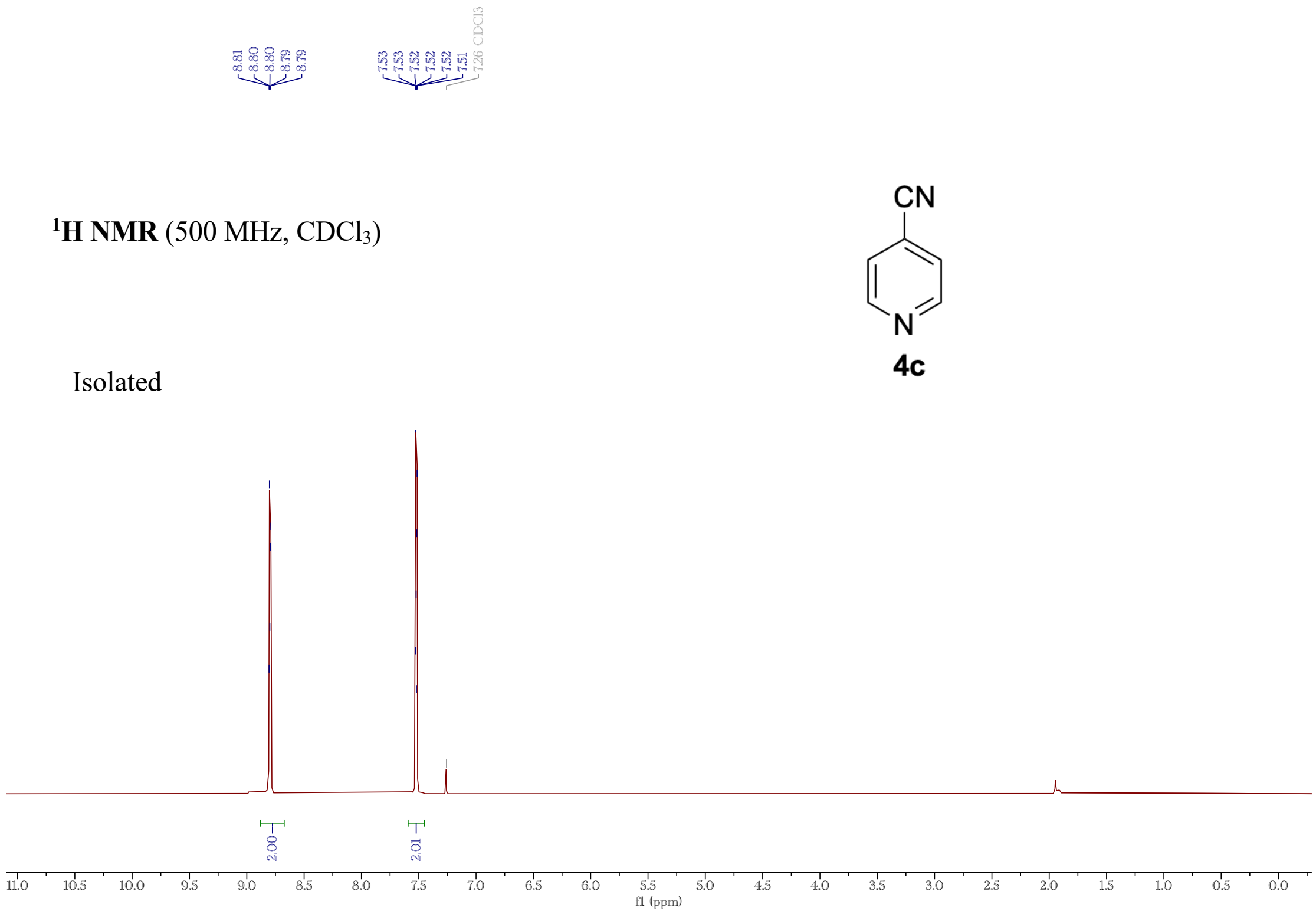

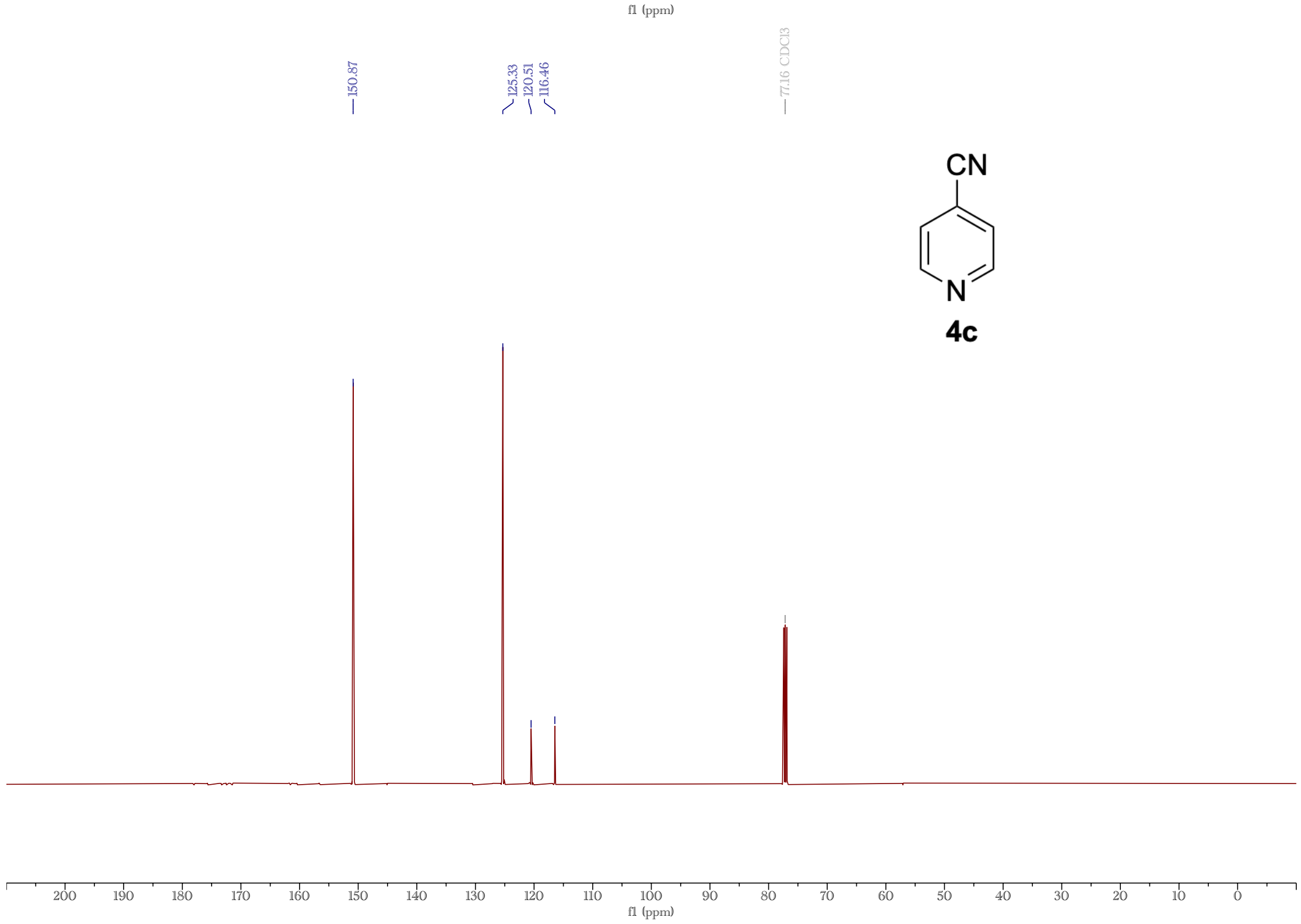

^{1}H NMR (500 MHz, $CDCl_3$)

Isolated

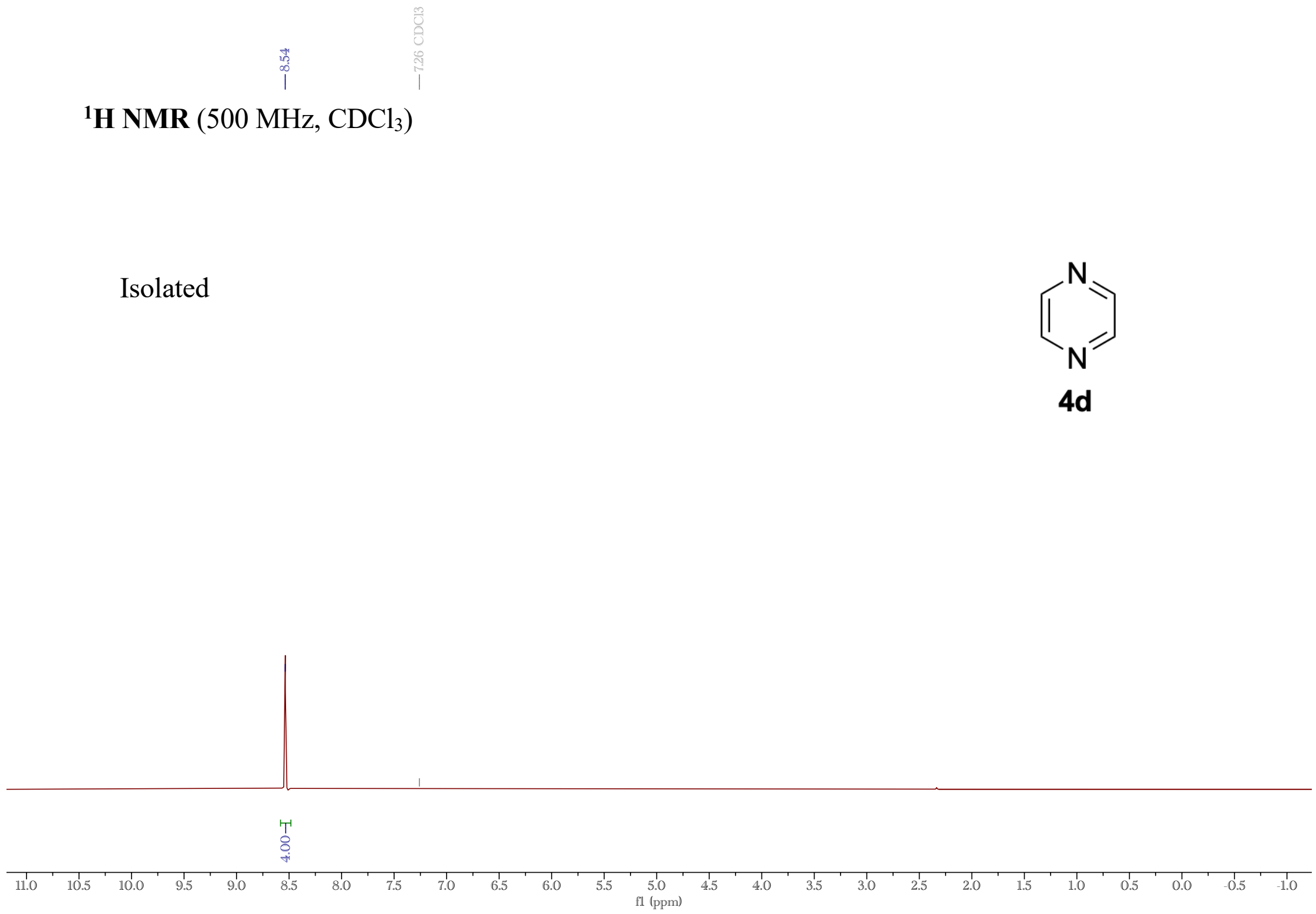

4d

^{13}C NMR (126 MHz, $CDCl_3$)

Isolated

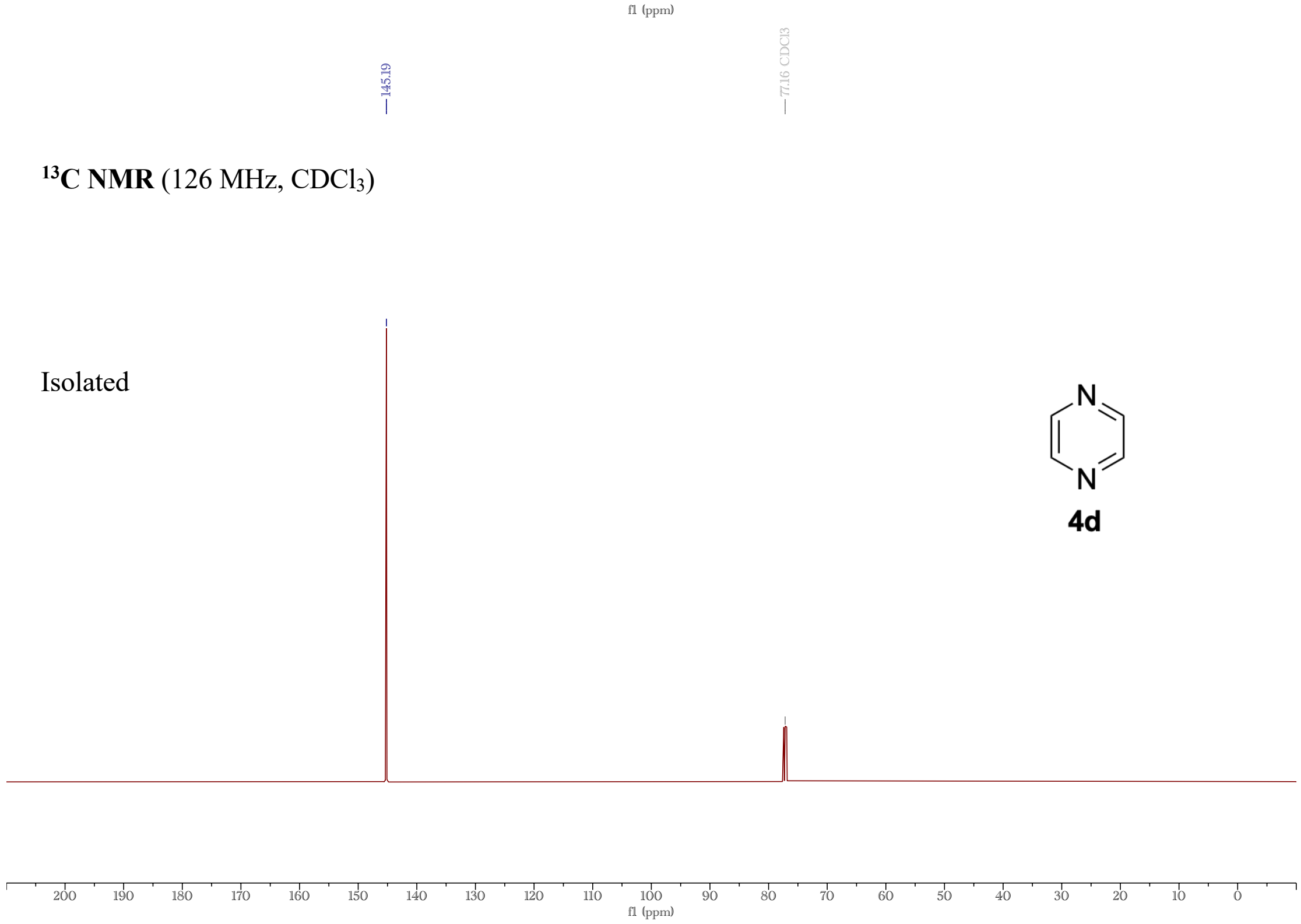

4d

^{1}H NMR (500 MHz, $CDCl_3$)

Isolated

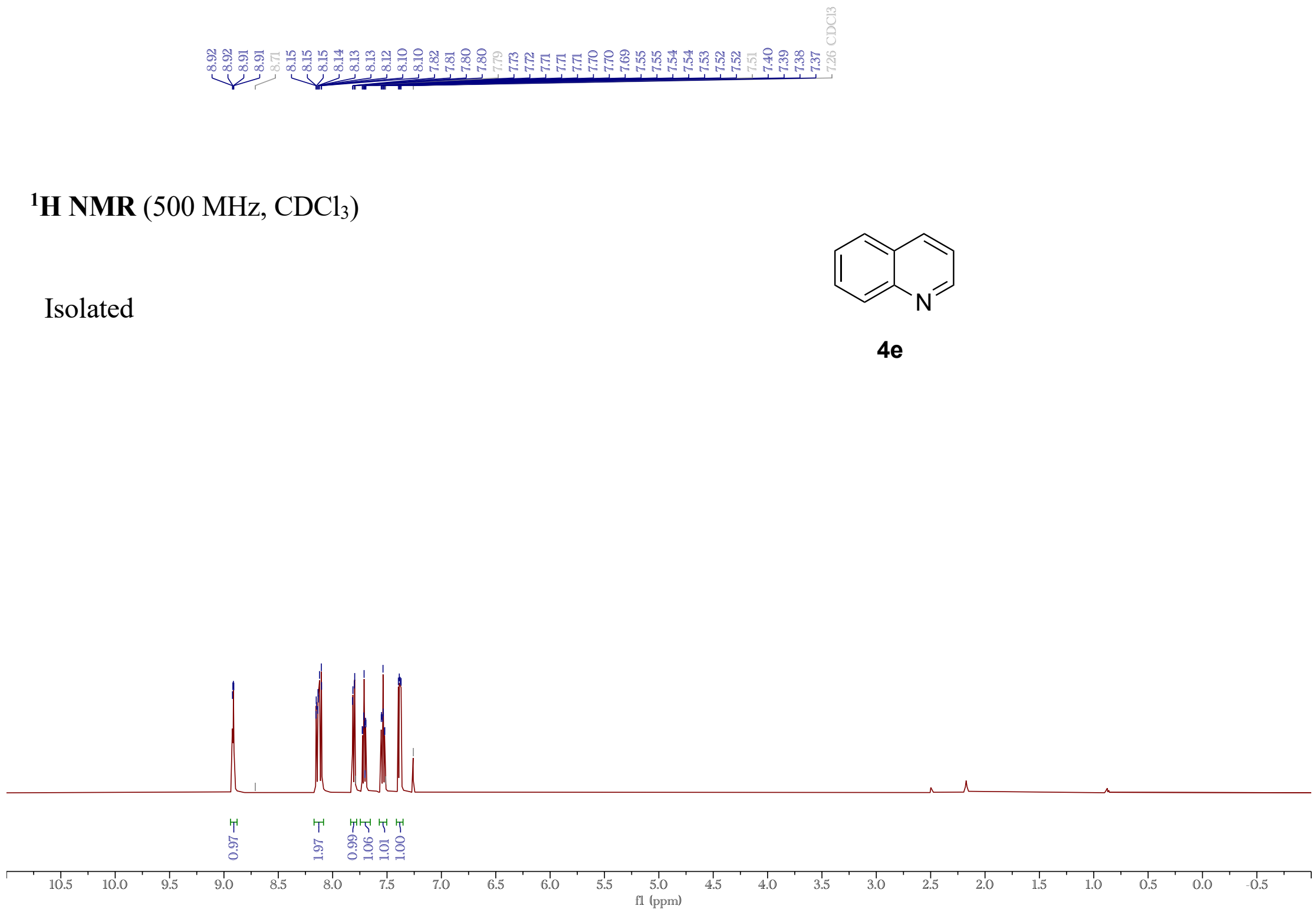

4e

^{13}C NMR (126 MHz, $CDCl_3$)

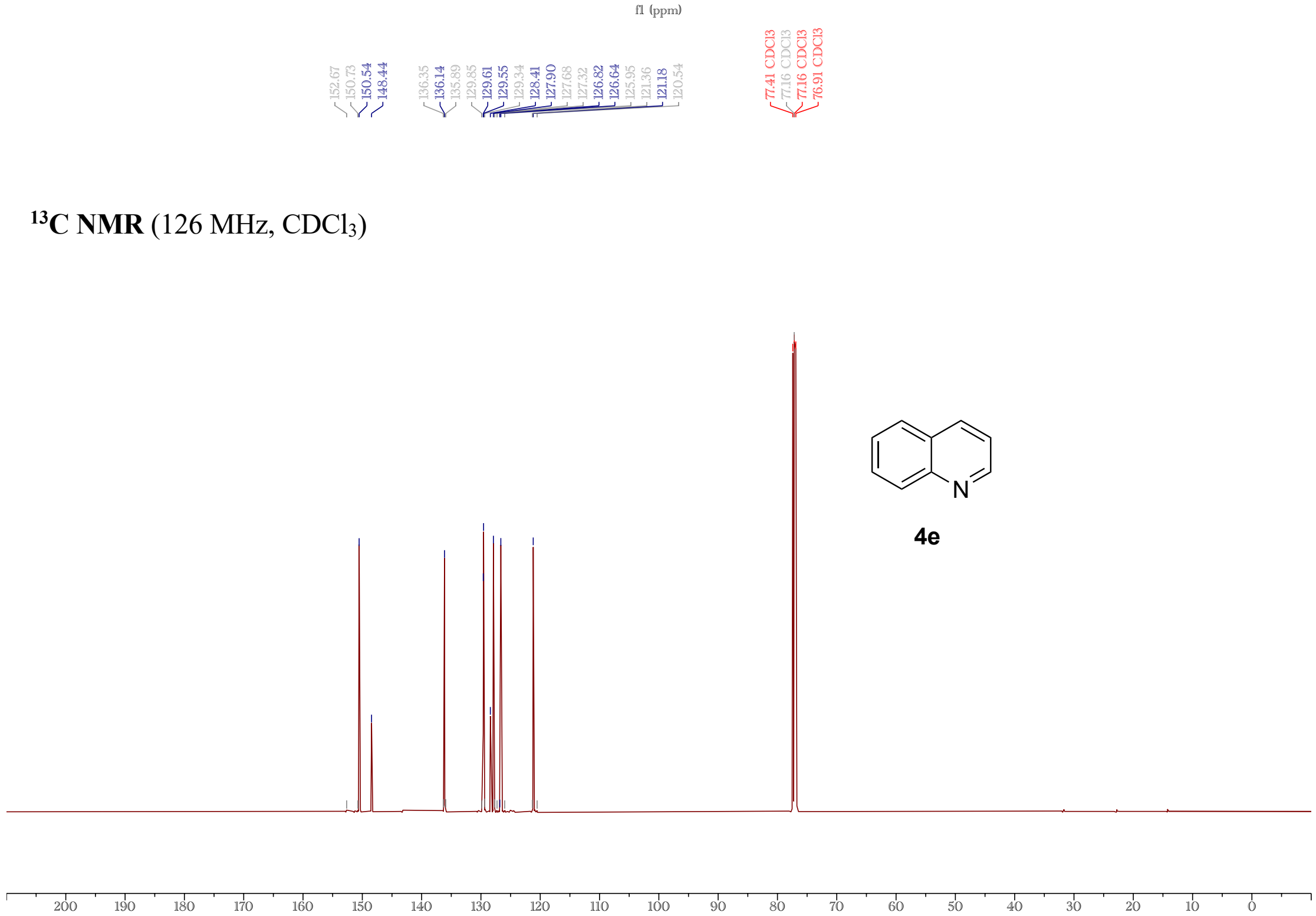

4e

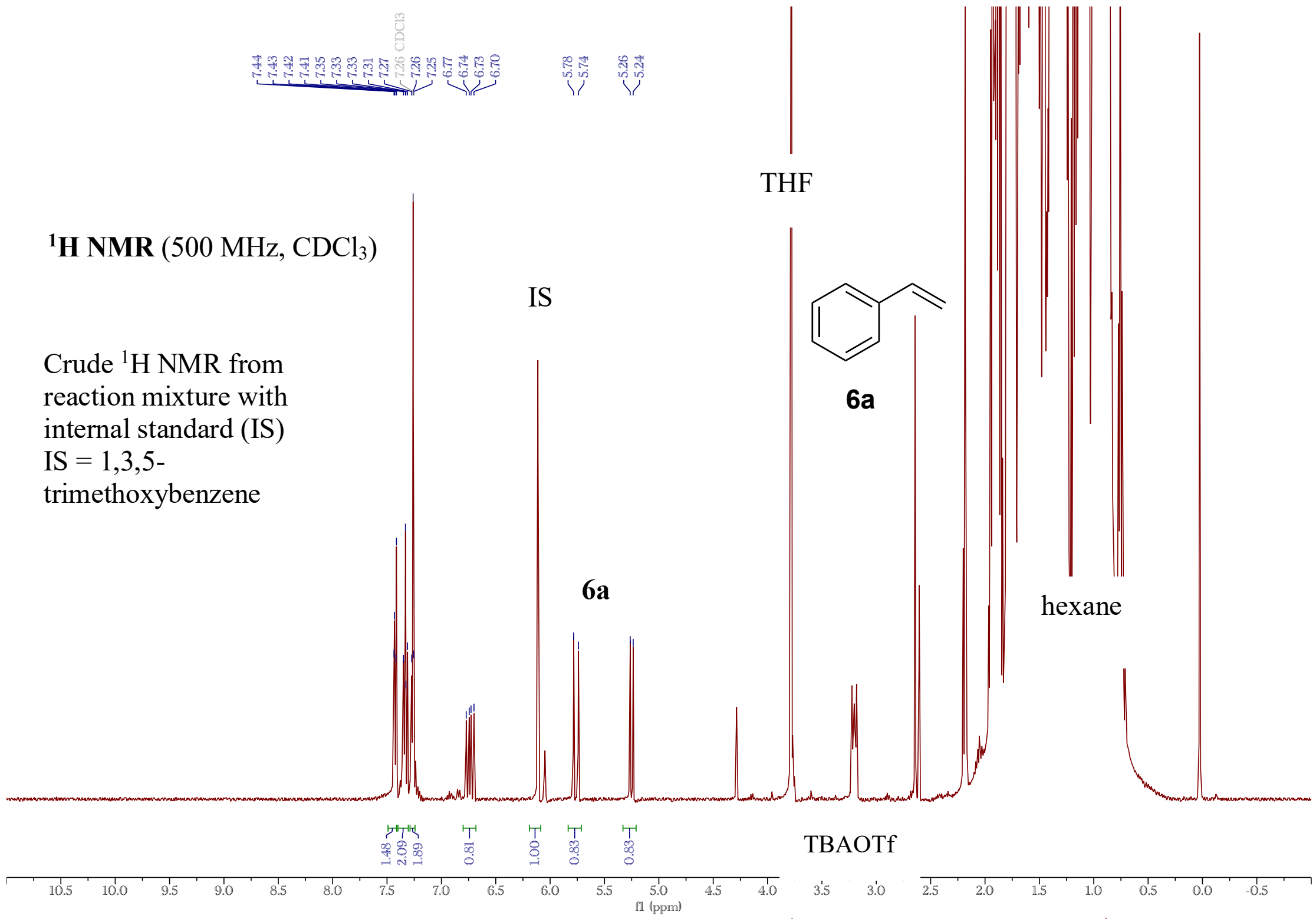

¹H NMR (500 MHz, $CDCl_3$)
Crude ¹H NMR from reaction mixture with internal standard (IS)
IS = 1,3,5-trimethoxybenzene
IS
THF
6a
6a
hexane
TBAOTf
f1 (ppm)

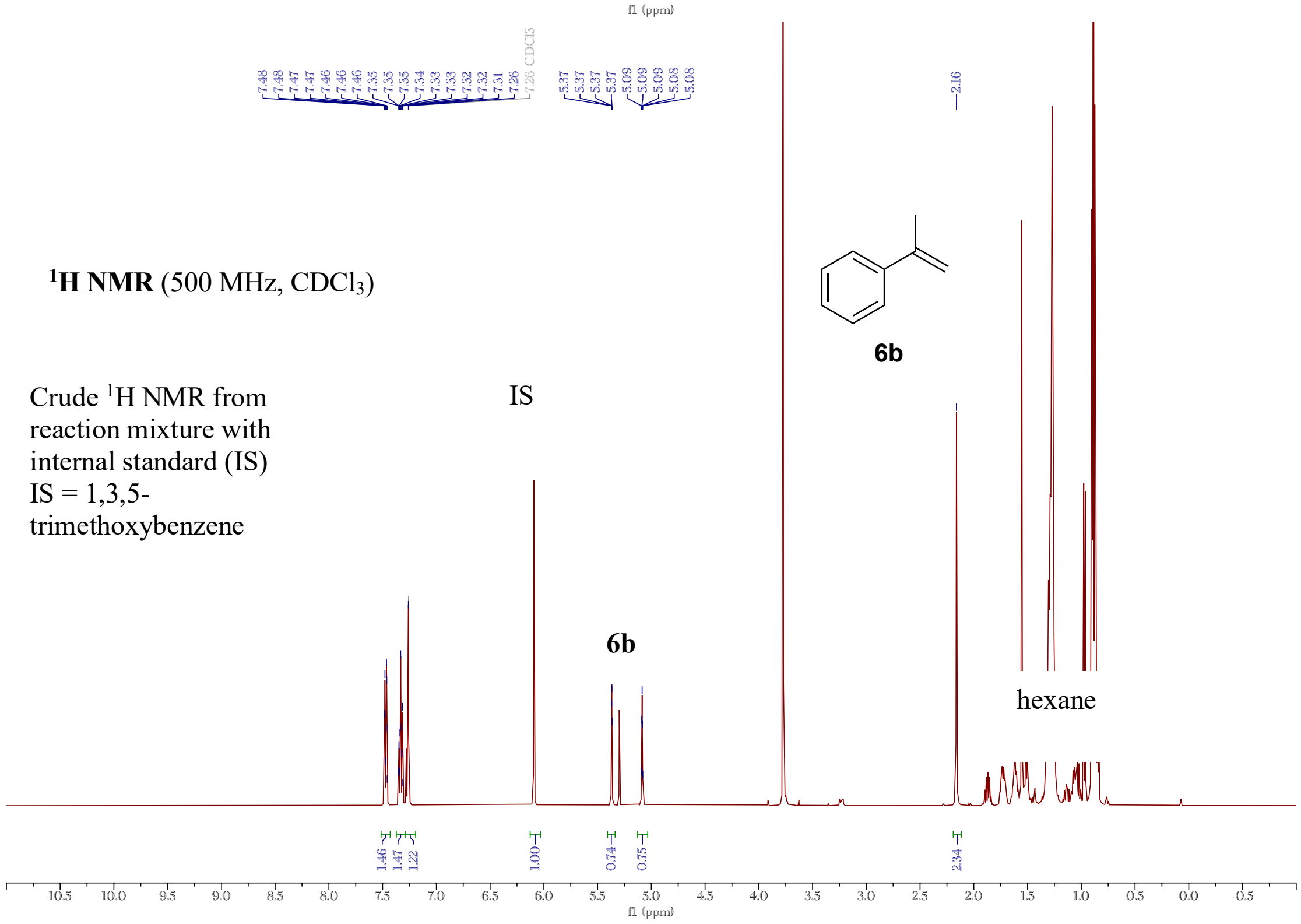

¹H NMR (500 MHz, $CDCl_3$)
Crude ¹H NMR from reaction mixture with internal standard (IS)
IS = 1,3,5-trimethoxybenzene
IS
6b
6b
hexane
f1 (ppm)

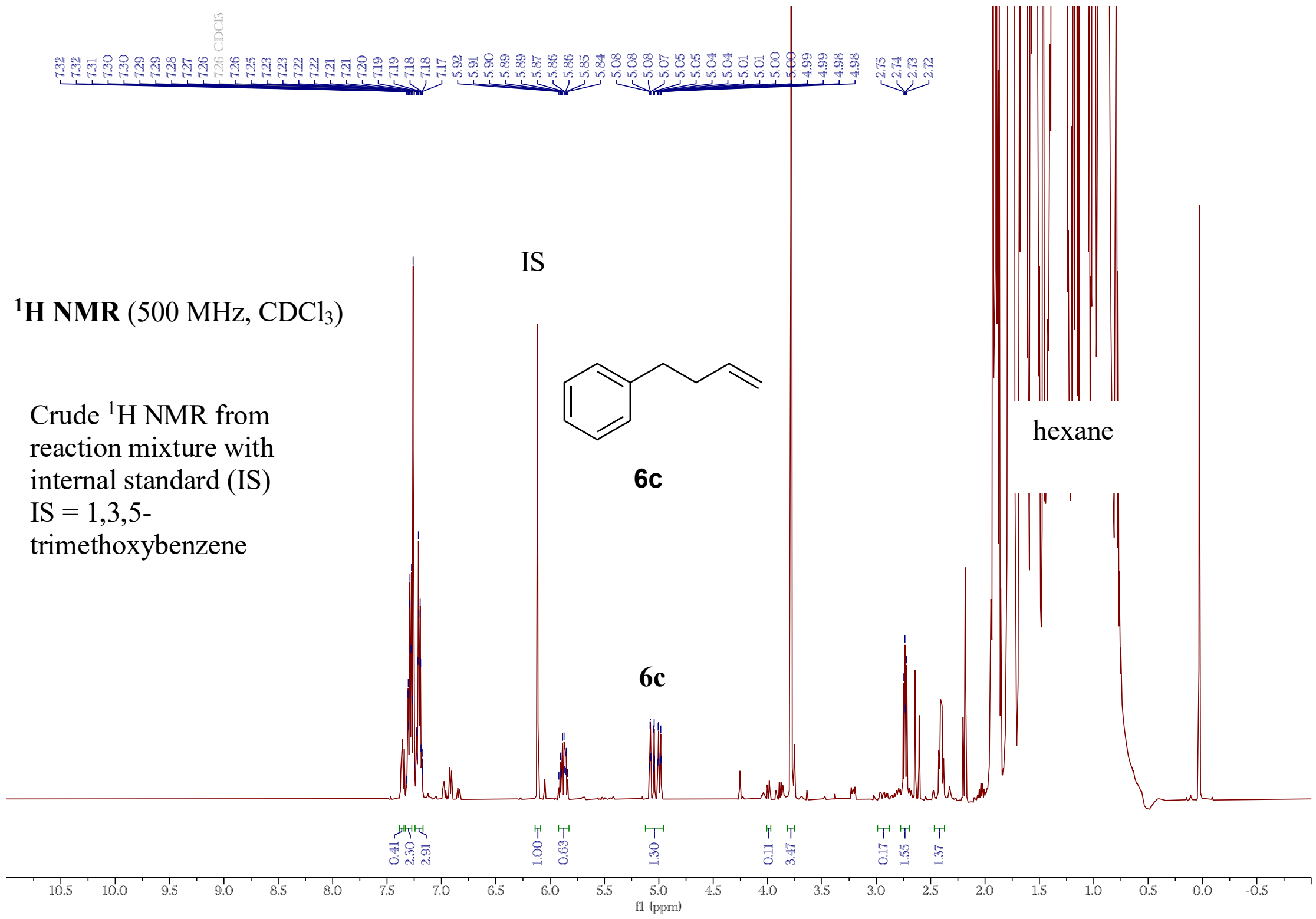
1H NMR (500 MHz, CDCl3)
Crude 1H NMR from reaction mixture with internal standard (IS)
IS = 1,3,5-trimethoxybenzene
IS
6c
6c
hexane
f1 (ppm)

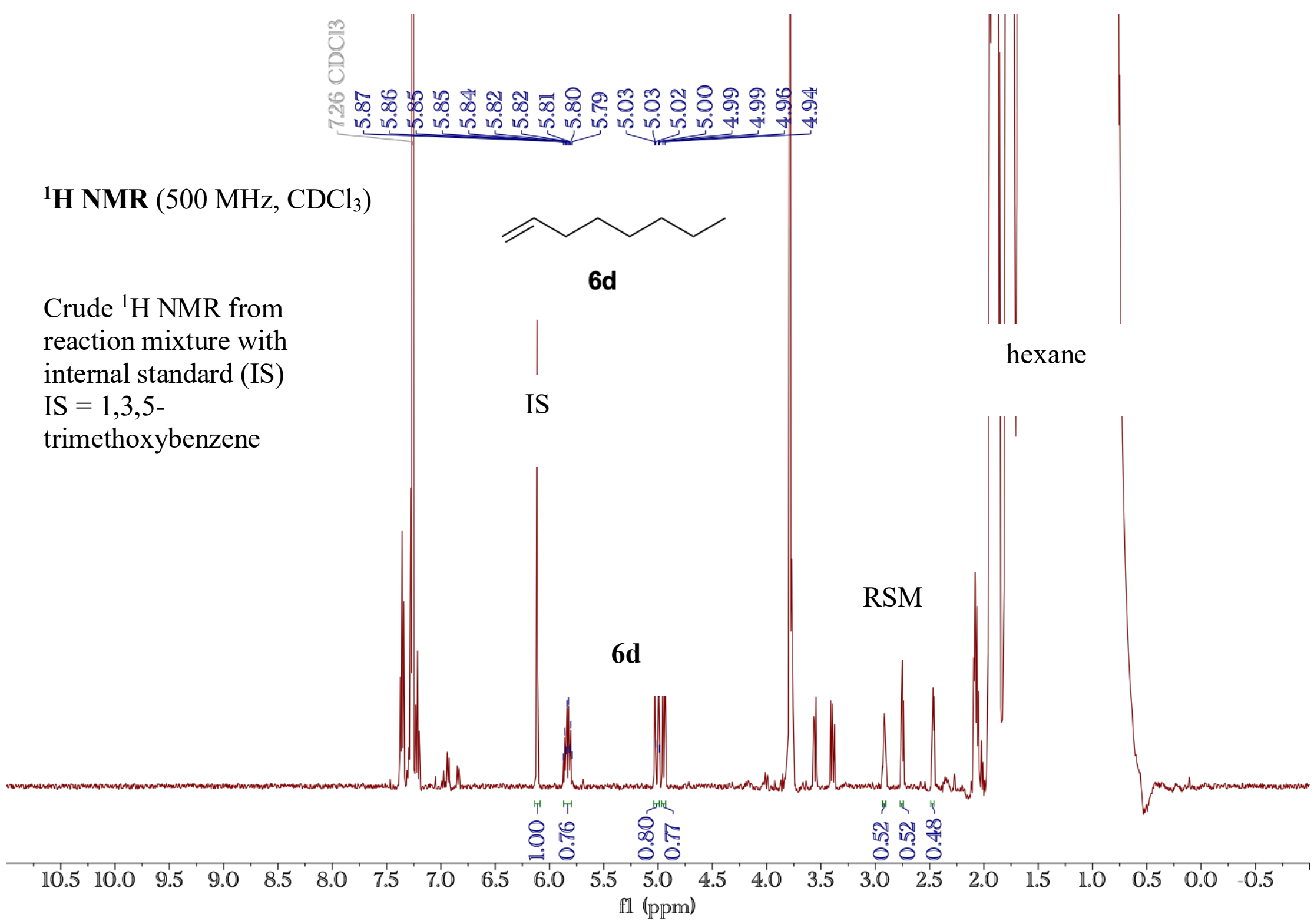
1H NMR (500 MHz, CDCl3)
Crude 1H NMR from reaction mixture with internal standard (IS)
IS = 1,3,5-trimethoxybenzene
6d
IS
6d
hexane
RSM
f1 (ppm)

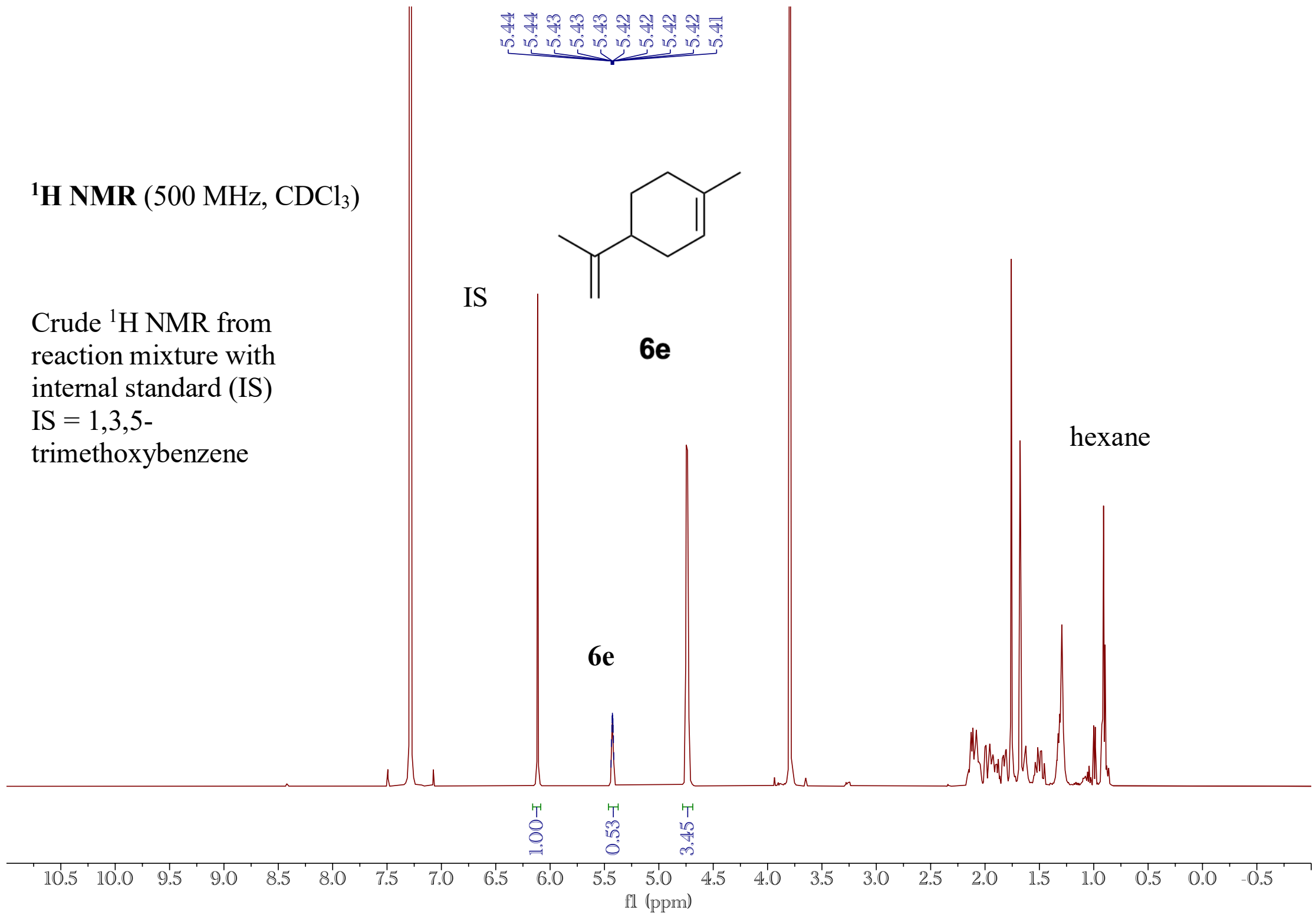

5.44
5.44
5.43
5.43
5.43
5.42
5.42
5.42
5.42
5.41
¹H NMR (500 MHz, $CDCl_3$)
Crude ¹H NMR from reaction mixture with internal standard (IS)
IS = 1,3,5-trimethoxybenzene
IS
6e
6e
hexane
1.00
0.53
3.45
10.5 10.0 9.5 9.0 8.5 8.0 7.5 7.0 6.5 6.0 5.5 5.0 4.5 4.0 3.5 3.0 2.5 2.0 1.5 1.0 0.5 0.0 -0.5
f1 (ppm)
